# Singlet oxygen-mediated selective C–H bond hydroperoxidation of ethereal hydrocarbons

**DOI:** 10.1038/s41467-017-01906-5

**Published:** 2017-11-27

**Authors:** Arunachalam Sagadevan, Kuo Chu Hwang, Ming-Der Su

**Affiliations:** 1 Department of Chemistry, National Tsing Hua University, Hsinchu, 30013 Taiwan; 2 Department of Applied Chemistry, National Chiayi University, Chiayi, 60004, Taiwan; 3 Department of Medicinal and Applied Chemistry, Kaohsiung Medical University, Kaohsiung, 80708 Taiwan

## Abstract

Singlet O_2_ is a key reactive oxygen species responsible for photodynamic therapy and is generally recognized to be chemically reactive towards C=C double bonds. Herein, we report the hydroperoxidation/lactonization of α-ethereal C–H bonds by singlet O_2_ (^1^Δ_g_) under exceptionally mild conditions, i.e., room temperature and ambient pressure, with modest to high yields (38~90%) and excellent site selectivity. Singlet O_2_ has been known for > 90 years, but was never reported to be able to react with weakly activated C–H bonds in saturated hydrocarbons. Theoretical calculations indicate that singlet O_2_ directly inserts into the α-ethereal C–H bond in one step with conservation of steric configuration in products. The current discovery of chemical reaction of singlet oxygen with weakly activated solvent C–H bonds, in addition to physical relaxation pathway, provides an important clue to a 35-year-old unresolved mystery regarding huge variations of solvent dependent lifetime of singlet O_2_.

## Abstract (web summary)

Singlet oxygen is known to react with carbon–carbon double bonds. Here, the authors show the unusual functionalization of α-ethereal C–H bonds mediated by singlet oxygen under mild conditions to afford lactones and hydroperoxide products.

## Introduction

Singlet oxygen is widely recognized owing to its reactivity towards alkenes^1, 2^, such as ene reactions, cycloaddition, and heteroatom oxidation^1, 2^, as well as its involvement in clinical photodynamic therapy treatments of tumors^3^. However, singlet O_2_ was never reported to be able to react with C–H bonds in saturated hydrocarbons. In the literature, the lifetimes of singlet oxygen were reported to be strongly dependent on solvents, ranging from 900 μs in tetrachloromethane (CCl_4_), 20 μs in tetrahydrofuran (THF) to 2 μs in H_2_O^4–7^. The large solvent dependence of singlet O_2_ lifetimes was once attributed to significant overlap between the singlet O_2_ phosphorescence emission bands and solvent infrared absorptions at ~ 1269 and ~ 1592 nm, as well as solvent perturbation-induced physical deactivation via electronic vibrational coupling^4, 5^. However, large d-solvent effects (K_H_/K_D_ ~ 5.8–19.9) on the lifetime of singlet O_2_ were also observed^6, 8^, which cannot be well explained by the solvent perturbation model^7, 9^. When strained hydrocarbons (such as cubanes) were used to quench the excited state of singlet oxygen, the quenching rate is one order faster than that predicted by the solvent C–H bond vibration-induced perturbation model^9^. There is a large variation and inconsistency between the experimentally measured singlet oxygen lifetimes and theoretically calculated values derived from solvent C–H bond vibration-induced physical perturbation model^7–9^. It was once found that the short lifetime of singlet oxygen is related to the presence of olefinic impurities in methyl tert-butyl ether (MTBE)^10^. Detailed analysis shows that in addition to the olefinic impurity quenching process, pure MTBE itself can also deactivate the singlet O_2_, via physical quenching, with a rate of 2.9 × 10^3^ M^−1^s^−1^ ^11^. The impurity quenching mechanism cannot account for the short lifetimes of singlet oxygen in various organic solvents, since not all solvents contain olefinic impurities. To-date, the origin of the huge lifetime variations of singlet O_2_ in different solvents is still a mystery^6–9, 12^.

Here, we report that direct oxidative hydroperoxidation of weakly activated α-ethereal C–H bond of aliphatic ether compounds could be achieved with excellent site selectivity and good yields via direct C–H bond insertion and conservation of steric configuration using singlet O_2_, in situ generated either by photo-sensitization of triplet sensitizers (such as Ir(ppy)_2_acac, rose bengal or *meso*-tetraphenylporphyrin, *meso*-TPP), direct photoexcitation of molecular oxygen using 760 nm or 1270 nm light, or by dark reaction of H_2_O_2_ with Na_2_MoO_2_ (or HClO). We also demonstrate that the singlet O_2_-mediated C–H bond functionalization can also be applied to molecules having complicated structures for hydroperoxidation/lactonization at the α-ethereal C–H site. Such a chemical reaction between singlet oxygen and solvent C–H bonds was never considered as a deactivation channel for singlet oxygen and might provide an important clue or explanation for the huge variation of singlet oxygen lifetimes in different solvents.

## Results

### Chemical reaction of THF with singlet oxygen

In this study, we demonstrate that singlet oxygen can chemically react with C–H bonds of various ethereal solvent molecules, leading to the formation of hydroperoxide and lactone products (Tables 1 and 2). We isolated singlet O_2_-THF chemical reaction products after photo irradiation of a THF solution in the presence of a singlet O_2_ photosensitizer, e.g., *meso*-TPP. THF was freshly distillated prior to all experiments to avoid interference from any possible pre-existing hydroperoxides. Surprisingly, after 2 h of photo irradiation by blue LEDs (460 nm, 40 mW cm^−2^) at room temperature, formation of 2-hydroperoxyl-THF (**2a**) and tetrahydrofuran-2(3 H)-one (**3a**) were observed with a total yield of 27%. The yield was increased to 45% in the presence of a Lewis acid, such as, γ-Al_2_O_3_ or Et_3_B, in the solution  (Table 1, entry 1). The hydroperoxidation and lactonization occur exclusively and selectively at the α-ethereal position, and no β-ethereal product was observed. The involvement of singlet O_2_ in the formation of **2a** and **3a** was confirmed by several control experiments listed below. Phosphorescence of singlet O_2_ at ~ 1270 nm was observed. In the absence of photosensitizers, no products and singlet O_2_ phosphorescence were observed under the same 460 nm LED light irradiation condition. The presence of NaN_3_, a well-known singlet O_2_ quencher, markedly suppresses the yields of **2a** and **3a**. Generation of singlet O_2_ via a dark reaction of H_2_O_2_ with strong oxidants (such as, HOCl or NaMoO_4_)^13^ in THF also leads to the formation of the products, **2a** and **3a**. Addition of olefins to the THF- *meso*-TPP solution results in the formation of expected singlet O_2_-ene reaction products and decreases the product yields of **2a** and **3a**.

**Table 1:** Substrate scope of aliphatic ethers

Selective α-hydroperoxidation/lactonization of aliphatic cyclic and linear ethers by ^1^O_2_. All neat substrates were irradiated by blue LEDs (40 mW cm^−2^) for 8 h at room temperature in the presence of 1 × 10^−5^ M of *meso*-TPP, ~ 1 atm O_2_ balloon, and the presence of γ-Al_2_O_3_. All values reported were based on isolated yields (after removal of unreacted starting substrate)

^[a]^ % of yield was expressed with respect to the amount of starting materials. Beside the listed products, the rest of material is unreacted starting substrate

^[b]^ Conv % = 100%−(% recovered starting substrate)

^[c]^ Yields were not reported due to the high volatility of the products

**Table 2:** Substrate scope of aromatic ring containing compounds

Selective α-hydroperoxidation/lactonization of aromatic cyclic/linear ethers by ^1^O_2_. All substrates (0.35 M, in DCM-ACN (9:1)) were irradiated by blue LEDs (40 mW cm^−2^) for 12 h at room temperature in the presence of 1 × 10^−5^ M of *meso*-TPP, ~ 1 atm O_2_ balloon, and in the presence of γ-Al_2_O_3_. All yields were reported based isolated yields (after removal of the unreacted starting substrate)

^[a]^ % of yield was expressed with respect to the amount of starting materials. Beside the listed products, the rest of material is unreacted starting substrate

^[b]^ Conv % = 100%−(% recovered starting substrate)

**Fig. 1:**
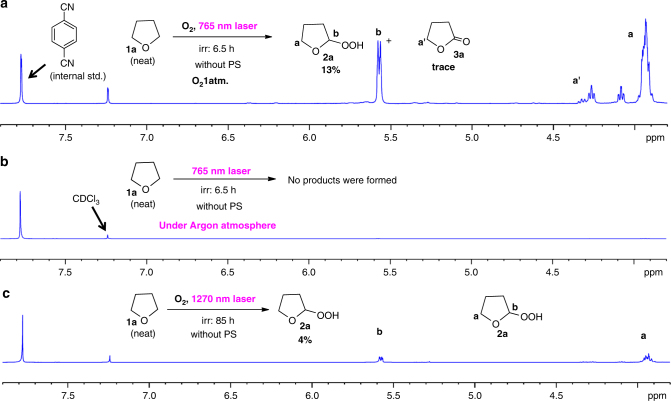
^1^H-NMR spectra of photo irradiation products. Products were obtained from direct photoexcitation of molecular oxygen in a neat THF solution under different conditions: **a** O_2_ atmosphere, 765 nm, 477 mW cm^−2^, continuous wave (CW mode), 6.5 h; **b** Ar atmosphere, 765 nm, 477 mW cm^−2^, CW mode, 6.5 h; and **c** O_2_ atmosphere, 1270 nm, 3.2 mW cm^−2^, CW mode, 85 h.  PS = photosensitizer

It is reported that singlet oxygen can be generated via direct photo irradiation of molecular oxygen by either 765 nm light^14^ or 1270 nm NIR light^15^. In the current system, freshly distillated neat THF was irradiated by 765 nm light (output from a Ti:sapphire laser, 477 mW cm^−2^, CW mode, 6.5 h) under 1 atm oxygen atmosphere. Figure 1a shows that THF-hydroperoxide and a subsequent lactone decomposition product were observed. These two products were not formed in an argon atmosphere under the same irradiation condition (Fig. 1b). When the THF-O_2_ solution was irradiated by 1270 nm (output from a diode laser, 3.2 mW cm^−2^, CW mode, 85 h), the THF-hydroperoxide was formed without co-formation of the lactone product (Fig. 1c). These experiments unambiguously demonstrate that it is singlet oxygen, that reacts with THF to form the THF-hydroperoxide (and/or lactone) product.

The O–O bond strength (34~45 kcal/mol) of hydroperoxides is very weak, and thermal decomposition occurs easily to form hydroxyl radical and O-centered alkoxyl radical^16^. The hydroxyl radical can abstract a proton from the weakest geminal C–H site, via breakage of a C–H bond (~ 98 kcal/mol), to form a strong O–H bond (~ 110 kcal/mol) and a C=O bond (~ 88 kcal/mol), leading to the formation of corresponding lactone. Overall, such an O–O bond cleavage in hydroperoxide or hydroperoxy acetals is exothermic and thermodynamically favored. Rearrangement of hydroperoxides (via the Hock rearrangement mechanism) or hydroperoxy acetals to form corresponding ketones, esters, or lactones are well documented in the literature^11, 17^. These rearrangements can be catalyzed by metal ions and Lewis acids^18^. Rapid explosion often occurs by mistakenly adding metal ions-containing waste water into hydroperoxides-containing organic wastes.

The chemical reaction rate of THF with singlet O_2_ was determined to be 3.8 × 10^3^ M^−1^s^−1^ (see Supplementary Disscussion and Supplementary Fig. 36) by using 1-methylcyclohexene as a competing reactant, of which a reaction rate of 0.16 × 10^6^ M^−1^s^−1^ with singlet O_2_ is known^19^. The THF reaction rate is compatible with those for poorly reactive olefins, such as, 2-methyl propene (4.4 × 10^3^ M^−1^s^−1^)^20^, and bimolecular quenching rates of singlet O_2_ by cubanes (10^3^~10^4^ M^−1^s^−1^)^9^. Such a chemical reaction rate corresponds to a singlet O_2_ lifetime of ~ 21 µs in neat THF, which is also in good agreement with the range of 20~30 µs lifetime of singlet O_2_ in THF as reported in the literature^4–7, 21^. In this study, we explicitly demonstrate that chemical reaction of singlet O_2_ with THF solvent molecules has a quantum yield of 0.93 (see Supplementary Equations 4, 5, and 6), and the chemical reaction rate is one order faster than the solvent collision induced physical relaxation. Therefore, the chemical reaction of singlet oxygen with THF solvent molecule is the dominant deactivation channel responsible for the short lifetime (20 μs) of singlet O_2_ in THF, when compared to the very long lifetime (900 μs) of singlet O_2_ in CCl_4_. Such a chemical deactivation channel was not considered or recognized in the past 35 years to account for short lifetimes of singlet oxygen in various solvents. The observation of the chemical reaction between singlet O_2_ and α-ethereal C–H bonds of THF is consistent with some previously observed clues that the inverse singlet O_2_ lifetime (1/*τ*) is linearly proportional to molar concentrations of solvent C–H bonds^5, 6^, and negative activation volume observed in high pressure lifetime measurements^22^.

### Synthetic applications

**Fig. 2:**
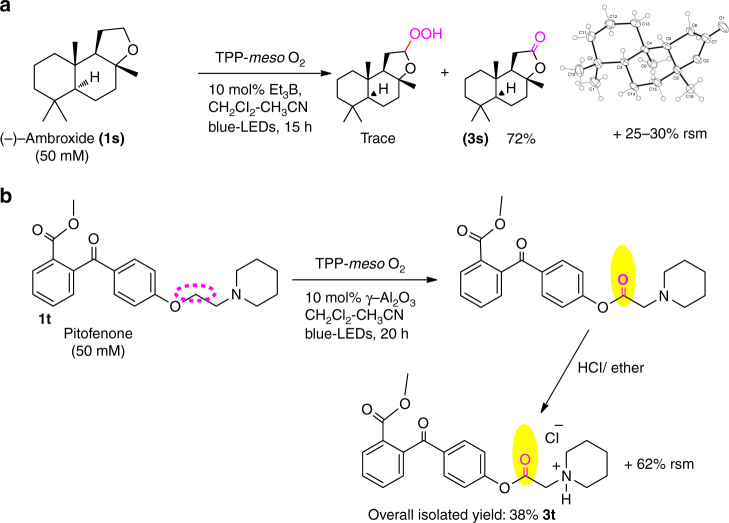
Singlet oxygen reaction with structurally complex substrates. Singlet O_2_-mediated direct aliphatic α-ethereal C–H oxidative functionalization of substrates: (**a**) (-)-ambroxide, and (**b**) pitofenone. Rsm: recovered starting material. The yield refers to the isolated product after workup

To explore the synthetic application of such a unique singlet O_2_-mediated α-ethereal C–H bond functionalization, many cyclic/linear aliphatic ether-containing substrates were subjected to photo irradiation in the presence of air and a photosensitizer. Tables 1 and 2 show many aliphatic cyclic and linear ether substrates can react with singlet O_2_ to form hydroperoxides and/or lactones with yields ranging from 24 to 90% after 8-12 h blue LED light irradiation at room temperature in the presence of a Lewis acid, γ-Al_2_O_3_ under 1 atm O_2_ atmosphere. In the absence of γ-Al_2_O_3_, the same products were obtained in lower yields. When the substrates contain both α-methylene C–H and α-methine C–H bonds, the hydroperoxidation occurs selectively and exclusively at the methine C–H bond (Table 1, entries 6–8). Upon co-existence of α-benzylic C–H and α-methylene C–H bonds, the hydroperoxidation occurs selectively and exclusively at the α-benzylic C–H bond (Table 2, entries 14 and 15). To further explore the possibility of using singlet O_2_ for α-ethereal C–H bond functionalization of ethereal molecules with complex structures, (-)-ambroxide (**1s**) was photolyzed in a dichloromethane-acetonitrile (9:1) solution in the presence of *meso*-TPP (1 × 10^−5^ M) and 10 mol% triethyl borane (Et_3_B). After 15 h blue LED light irradiation, sclareolide (**3s**) was obtained with 72% yield (Fig. 2a). The product was confirmed by ^1^H/^13^C NMR spectroscopy, mass spectrometry and single crystal X-ray diffraction (see Supplementary Fig. 29 and 30). As expected, lactonization occurs selectively and exclusively at α-ethereal C–H bond of this macrocyclic ether, but not at methyl, methylene or methine C–H bonds. Only trace amounts of hydroperoxide product were observed, which were characterized by ^1^H-NMR. When pitofenone was subjected to a reaction with singlet O_2_, an ester was formed selectively at the methylene site adjacent to the ethereal oxygen, but not at those adjacent to tertiary amine site (Fig. 2b).

### Reaction mechanism

**Fig. 3:**
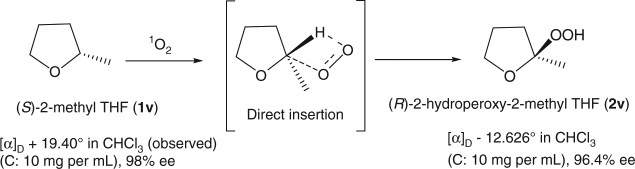
Direct C–H bond insertion and retention of configuration. Mechanistic study for singlet O_2_-mediated direct aliphatic α-ethereal C–H oxidative functionalization of (*S*)-2-methyl THF. The optical rotation and ee values are based on experimental measurements (see Supplementary Fig. 32–35)

To examine whether the hydroperoxidation of α-ethereal C–H bond by singlet O_2_ occurs via a single-step direct insertion process or two steps hydrogen atom abstraction followed by radical recombination, chiral (*S*)-2-methyl THF (**1v**, ((α)_D_ + 19.40°, 98% ee, see Supplementary Figs. 32–35) was synthesized according the procedure from the literature^23^, and then subjected to singlet O_2_ oxidation under standard condition. As expected, hydroperoxidation reaction occurs selectively at the α-methine C–H site. Chiral column separation data (see Supplementary Fig. 32–35) show that the hydroperoxidation products ((α)_D_ −12.626°) contain 98.22% (*R*)-2-hydroperoxyl-2-methyl THF and 1.78% (*S*)-2-hydroperoxyl-2-methyl THF, that is, 96.4% ee excess of (*R*)-2-hydroperoxyl-2-methyl THF, which is close to the 98% ee of the starting chiral (*S*)-2-methyl THF. The chiral (*R*)-2-hydroperoxyl-2-methyl THF has the same steric configuration as the starting substrate ((α)_D_ + 19.40°, see Supplementary Figures 32~35)^23^. The oxidation of C–H to COOH proceeds with at least nearly complete steric retention. The results unambiguously indicate that the hydroperoxidation of α-ethereal C–H bond by singlet O_2_ occurs via a single-step direct insertion process (Fig. 3).

In addition to the above singlet oxygen direction C–H bond insertion mechanism, the involvements of other species, such as superoxides (via electron transfer from electronically excited photosensitizers to molecular oxygen) or radicals, was also examined. The addition of 25 mol% of KO_2_, a stable source of superoxide, to THF for 36 h in the dark did not result in the formation of any trace amounts of THF-hydroperoxide or lactone. Therefore, the participation of superoxide in the reaction with THF to form the oxidation products, **2a** and **3a**, is unambiguously excluded. We have also added 5 mol% (relative to photosensitizer) of a radical trapping reagent, namely, DMPO (5,5-Dimethyl-1-Pyrroline-*N*-Oxide), into a *meso*-TPP -THF solution. The total yield of THF-hydroperoxide was reduced slightly from 27% (in the absence of DMPO) to 22% (in the presence of DMPO) after 8 h photo irradiation using 100 W Hg lamp, suggesting that very little (or no) amount of THF-hydroperoxide/lactone was produced via a radical-associated pathway.

### Theoretical calculations

**Fig. 4:**
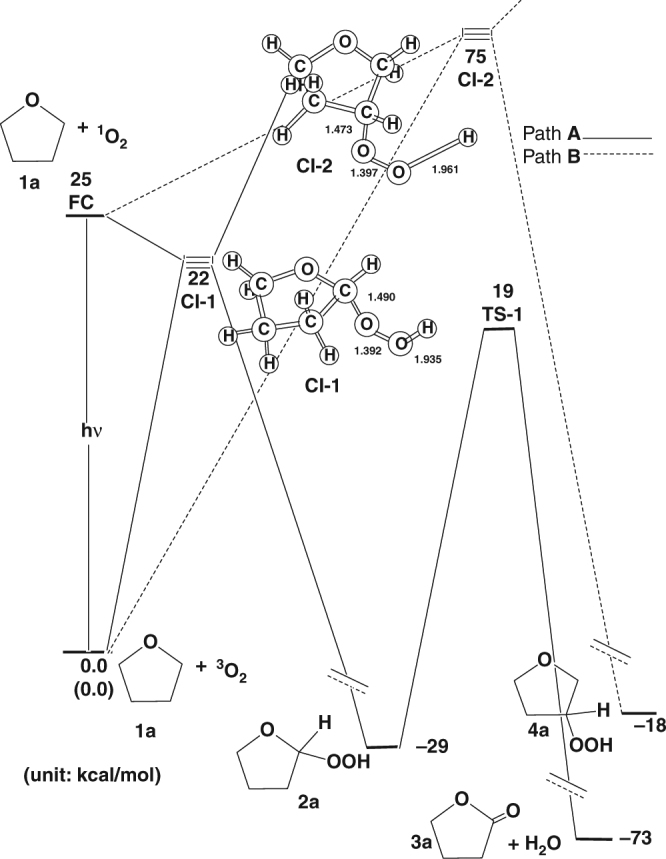
Theoretical calculated energy diagram. Theoretical calculations of direct singlet oxygen insertion into α and β C–H bonds of THF adjacent to the ethereal oxygen were carried out using the CAS-MP2(20,14)/6-311 G(p)//CAS(20,14)/6-31 G(p) method

To look for theoretical support to the above observed one-step direct insertion process of singlet O_2_ to the α-ethereal C–H bond, we carried out theoretical calculations at the CAS-MP2(20,14)/6-311 G(p)//CAS(20,14)/6-31 G(p) level of theory^24^. Figure 4 shows that the excited state energy of singlet O_2_ at the Frank-Condon geometry (FC) is 25 kcal/mol above the ground state configuration. Two minimum-energy pathways on the singlet excited potential energy surface of oxygen were characterized by optimizing the geometries along path A (the direct insertion of singlet O_2_ at the α-ethereal C–H bond) and path B (the direct insertion of singlet O_2_ at the β-ethereal C–H bond), which can lead to the different products (**2a**, **3a** + H_2_O, and **4a**, respectively). The results of our theoretical calculations indicate that **CI-1** is 3.0 kcal mol^−1^ lower in energy than FC, whereas **CI-2** is 50 kcal mol^−1^ above FC. The theoretical evidences demonstrate that the path A is strongly preferred over the path B. The THF-hydroperoxide product **2a** can be easily produced during the irradiation of reactants (**1a** and triplet ground state molecular O_2_) with light. Moreover, tunneling through the **CI-1** point can lead to either the direct insertion product (**2a**) or the initial ground state reactants. It has to be pointed out that the singlet **2a** can revert to the starting materials (**1a** and triplet ground state molecular O_2_) in a thermal process on the ground state (Fig. 4). After the minimum **2a** on the ground state energy surface is produced, there is another reaction pathway (**TS-1**), from which one may obtain the final rearrangement products (**3a** and H_2_O). The theoretical findings reveal that due to the high excess energy of 51 kcal/mol, resulting from the

**CI-1** to THF-hydroperoxide **2a**, the barrier of 48 kcal mol^−1^ from **2a** to a more stable lactone product, **3a**, that can be easily overcome (in the absence of any catalysts). Accordingly, the theoretical investigations suggest that the final lactone product, **3a**, can be generated either through the direct irradiation of the initial reactants (**1a** and triplet ground state molecular O_2_). The above calculation for conversion of hydroperoxide **2a** to lactone **3a** is based on the condition in the absence of any catalysts. It has been reported in the literature that metal ions/Lewis acid can catalyze dehydration of hydroperoxide to lactone^18^. The presence of catalysts may find other pathways with lower activation energies. However, the involvement of catalysts in theoretical calculations is too complicated and beyond the scope of this study.

## Discussion

In the current study, the site selectivity of singlet O_2_ is most probably governed by the C–H bond strength of the α-ethereal C–H bond and the activation energy, rather than by its electrophilicity. From theoretical calculations, it is clear that the α-ethereal C–H bond strength and the energy barrier for direct singlet oxygen insertion at the α C–H bond are much lower than for the β-ethereal C–H bonds. Therefore, direct insertion of singlet oxygen occurs selectively at the α-ethereal C–H bonds, rather than at the more electron-rich β C–H site. The yield of C–H peroxidation products was enhanced in the presence of Lewis acids (such as, Et_3_B, γ-Al_2_O_3_, etc). Coordination of Lewis acids to the ethereal oxygen atom can help withdraw electrons and weaken adjacent α-ethereal C–H bond, favoring attack of α-ethereal C–H bonds by singlet oxygen and higher product yields. This type of site selectivity is unusual for electrophilic oxidants, such as singlet O_2_. This type of C–H bond site selectivity is similar to direct rhodium carbenoid insertion into C–H bond in alkanes/THF^25^, but is opposite to what was observed using electrophilic organometallic catalyst and H_2_O_2_ as an oxidant, where C–H functionalization occurs at the most remote position (or the most electron-rich site) away from the electron withdrawing directing group^26–28^. In organic chemistry laboratories, it is commonly known that 6~12 months exposure of THF cans to air and room light will result in the formation of THF-hydroperoxide (but not tetrahydrofuran-2(3 H)-one)^29^, which was once mistakenly attributed to photoexcitation of a O_2_-THF ground state charge transfer complex without involving singlet O_2_^29^. Our study shows that this type of hydroperoxide formation commonly observed in ethereal solvents might be due to the chemical reaction of singlet oxygen with solvent molecules, where singlet oxygen was formed via direct 6~12 months room light excitation of molecular oxygen or via photo-sensitization of trace amounts of light absorbing species existing in the solvents.

Overall, we have unambiguously demonstrated that α-ethereal C–H bonds of aliphatic ethers can be selectively functionalized by singlet O_2_ with moderate to good yields and excellent site selectivity via a one-step direct insertion process with conservation of steric configuration. The C–H bond insertion rate to α-ethereal C–H bond in THF was experimentally determined to be ~ 3.8 × 10^3^ M^−1^s^−1^ We believe that such a type of singlet O_2_ reaction will soon find its wide applications in synthetic chemistry and likely play a role in photodynamic therapy destruction of cancer cells. This study provides important clues to the literature mystery regarding the lifetime variation of singlet oxygen in various solvents by considering the chemical reaction pathway of singlet oxygen with weakly activated solvent C–H bonds.

## Methods

### General procedure

All reactions were conducted under an oxygen atmosphere and oven-dried glass wares were used. All reactions were conducted using blue LED lights (30 lamps, power density: 40 mW cm^−2^) or 100 W Hg lamp as a light source without any filter. All solvents were dried according to known methods, and distilled prior to use. Starting materials including photosensitizers were commercially available and used as purchased. NMR spectra were recorded in CDCl_3_, ^1^H-NMR at 400 and 600 MHz, ^13^C NMR at 100 and 150 MHz. Data reported as: s = singlet, d = doublet, t = triplet, q = quartet, m = multiplet, and b = broad. Specific optical rotation was obtained by JASCO p-2000 polarimeter (serial no: A060361232) with a sodium lamp and are reported as follows: (*α*)_D_ (c = 10 mg/mL, solvent: CHCl_3_). For other details of experimental procedures, please refer to the Supplementary Methods.

### Data availability

The authors declare that data supporting the findings of this study are available within the paper and the supplementary information file. Supplementary Figs. 29–30 for **3p** and **3s** can be obtained from the Cambridge Crystallographic Data Centre www.ccdc.cam.ac.uk/data_request/cif on quoting registry no. CCDC 1436061 (**3p**) and CCDC 1436060 (**3s**).

## Electronic supplementary material

Supplementary Information

Peer Review File
