# Supplementary material for: Singlet oxygen-mediated selective C–H bond hydroperoxidation of ethereal hydrocarbons
Source: Nat Commun. 2017 Nov 27;8:1812. doi: 10.1038/s41467-017-01906-5 (PMC5703888; doi:10.1038/s41467-017-01906-5)
Supplement: Supplementary file 1 — Supplementary Information [file 41467_2017_1906_MOESM1_ESM.pdf]

Supplementary Methods.

All reactions were conducted under an oxygen atmosphere and used oven-dried glass wares. All reactions were conducted using 100 W Hg lamp as a light source without any filter or blue-LED lights (30 lamps, power density: 40 mW cm^{-2}). All solvents were dried according to known methods and distilled prior to use. Starting materials at the highest commercial quality were purchased and purified by distillation or passing through an activated alumina column. Photosensitizers were used as received without further purification. NMR spectra were recorded using $CDCl_3$. ^{1}H NMR were recorded at 400 or 600 MHz, and ^{13}C NMR at 100 or 150 MHz. Data reported as: s= singlet, d= doublet, t= triplet, q= quartet, m= multiplet, and b= broad. Specific optical rotation was obtained using JASCO p-2000 polarimeter (serial no: A060361232) with a sodium lamp and is reported as follows: $[\alpha]_D$ (c= 10 mg/1mL, solvent: $CHCl_3$). Chiral Gas Chromatography analysis were carried out using a GC-2014 SHIMADZU gas-chromatograph (serial no: C11484301285SA) equipped with a FID detector and suitable for operation with fused silica capillary column. The carrier gas was high purity grade nitrogen (N_2).

General procedure for C-H bond functionalization of cyclic and linear ether: A dry test tube (20 mL) with rubber septum and magnetic stirrer bar was charged with aliphatic ether (1 mL). The test tube was purged with dry oxygen for 10 minutes and then added 1 x 10^{-5} M of photosensitizer (typically *meso*-TPP or rosebengal) and again purged with dry oxygen for 5 minutes. Finally, it was added 10 mol% of Lewis acid (γ-Al_2O_3). The solution was then irradiated using100 W Hg lamp or a blue-LEDs array for 8 h at room temperature under an 1 atm oxygen atmosphere. The unreacted starting material was evaporated using a rotary evaporator, and the residue products were submitted for NMR analysis.

General procedure for Aromatic ethers: A dry test tube (20 mL) with rubber septum and magnetic stirrer bar was charged with aromatic ether (1 mmol). The test tube was purged with dry oxygen for 10 minutes, added 1 x 10^{-5} M of photosensitizer (typically *meso*-TPP or rose bengal) and again purged with dry oxygen for 5 minutes. Finally, it was added 10 mol% of Lewis acid (γ-Al_2O_3). The solution was irradiated using a 100 W Hg lamp or a blue-LEDs array for 12 h at room temperature under an 1 atm oxygen atmosphere. The unreacted starting material was removed via a silica column using hexane and ethyl acetate as eluent.

Selective oxidative alpha ethereal C-H functionalization of pitofenone. A dry test tube (20 mL) with rubber septum and magnetic stirrer bar was charged with pitofenone **1t** (1 mmol), and then bubbled with dry oxygen for 10 minutes before addition of 1 x 10^{-5} M of photosensitizer (*meso*-TPP) and 10 mol% of Lewis acid (γ-Al_2O_3). The solution was then irradiated using a blue-LEDs

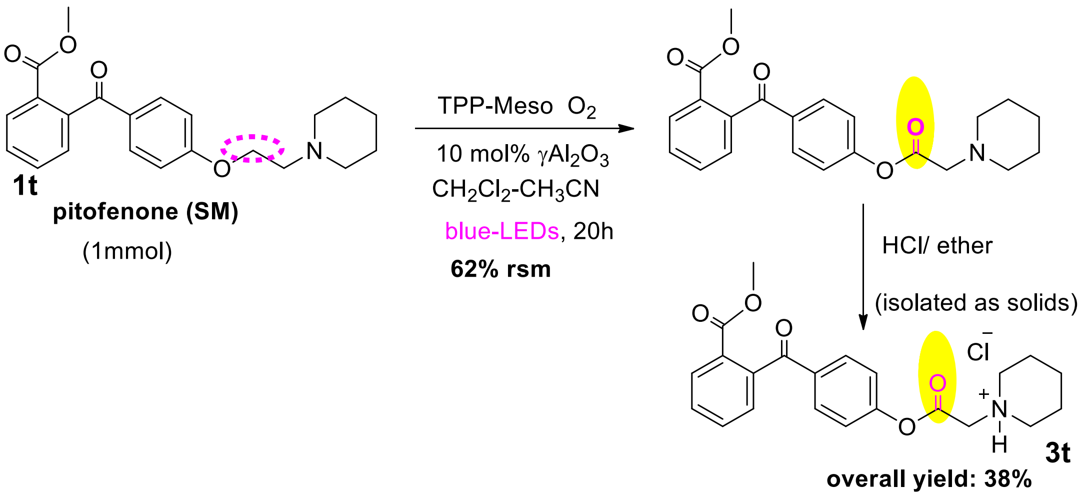

array at room temperature (25-28 ^{o}C) under an 1 atm oxygen atmosphere for 20 h. The reaction mixture was diluted with ethyl acetate-hexane (4:6 volume ratio), and stirred for 10 min. The mixture was filtered through celite, silica gel pads, and washed with ethyl acetate. The filtrate was concentrated using a rotary evaporator. The residue products were purified by column chromatography on silica gel and collected as pasty form. The collected product was dissolved in ether, followed by slow addition of HCl in ether under stirring for 2 h at room temperature. Finally, the solid product (**3t**) was collected by filtration.

Kinetic isotope labeling experiments by THF-d$_8$. THF-d_8 was used as a starting material to react with singlet oxygen, which leads to the production of THF-d_8 peroxide in 8% yield after 15 h irradiation using a 100 W Hg lamp. The yield (8%) of THF-d_8 hydroperoxide is significant lower than that (35%) of the THF-H_8 hydroperoxide under the same condition, which is due to the stronger C-D bond than the C-H bond and is consistent with the expected kinetic isotope effect.

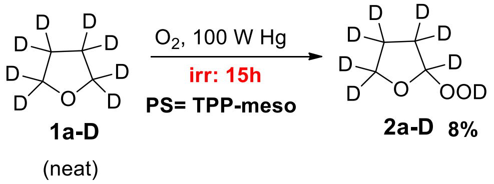

Spectroscopic Data.

Hydroperoxy-tetrahydrofuran (2a)[1,2] (CAS Registry No: 4676-82-8)

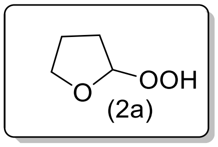

Colorless oil; **^{1}H NMR** (400 MHz, $CDCl_3$): δ 9.1 (s, 1H), 5.58-5.56 (m, 1H), 3.95-3.93 (m, 2H), 2.04-1.82 (m, 2H); **^{13}C NMR** (100 MHz, $CDCl_3$): δ 107.9, 67.6, 29.0, and 23.8.

NMR data of (2a) was identical with that in the literature[1,2].

Dihydrofuran-2(3H)-one (3a) (CAS NO: 96-48-0)

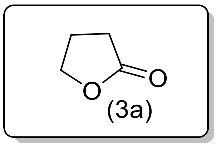

Colorless oil; **^{1}H NMR** (400 MHz, $CDCl_3$): δ 4.33-4.30 (t, 2H), 2.48-2.45 (t, 2 H), 2.26-2.22 (m, 2 H); **^{13}C NMR** (100 MHz, $CDCl_3$): δ 178.1, 68.8, 27.9, and 22.5.

*NMR data (***3a***) is in agreement with authentic commercially available sample.*

2-hydroperoxytetrahydro-2H-pyran (2b)[3] (CAS Registry No: 4676-84-0)

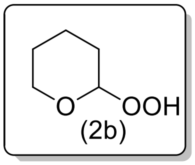

Colorless oil; **^{1}H NMR** (400 MHz, $CDCl_3$): δ 8.16 (s, 1H), 5.10-5.08 (t, 1H), 3.99-3.94 (m, 2H), 3.66-3.62 (m, 1H), 1.77-1.54 (m, 6H); **^{13}C NMR** (100 MHz, $CDCl_3$): δ 102.4, 62.7, 27.2, 25.0 and 19.4. NMR data of (**2b**) was identical with that in the literature[3].

2-tetrahydro-2H-pyran-2-one (3b) (CAS NO: 542-28-9)

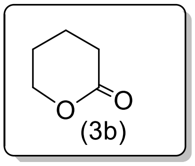

Colorless oil; ^{1}**H NMR** (400 MHz, $CDCl_3$): δ 8.16 (s, 1 H), 4.34-4.31 (t, 2H), 2.58-2.48 (t, 2H), 1.89-1.56 (m, 4H); ^{13}**C NMR** (100 MHz, $CDCl_3$): δ 172.2, 69.8, 30.1, 22.8, and 19.2.
NMR data is in agreement with authentic commercially available sample.

2-hydroperoxyoxepane (2c)[3] (CAS Registry No: 366817-95-0)

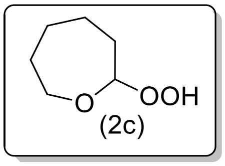

Colorless oil; ^{1}**H NMR** (400 MHz, $CDCl_3$): δ 8.74 (s, 1H), 5.21-5.17 (m, 1H), 3.81-3.62 (m, 2H), 2.04-1.61 (m, 8H); ^{13}**C NMR** (100 MHz, $CDCl_3$): δ 106.2, 62.8, 30.7, 30.6, 29.2 and 22.8.
NMR data of (2c) was identical with that in the literature[3].

Oxepan-2-one (3c) (CAS NO: 502-44-3)

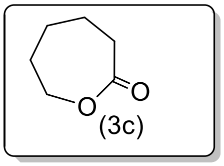

Colorless oil; ^{1}**H NMR** (400 MHz, $CDCl_3$): δ 4.17-4.12 (m, 2H), 2.57-2.53 (q, 2H), 1.78-1.67 (m, 6H); ^{13}**C NMR** (100 MHz, $CDCl_3$): δ 176.0, 69.0, 34.3, 29.0, 28.6, and 22.7.
NMR data is in agreement with authentic commercially available sample.

2-hydroperoxy-1, 3-dioxolane (2d)[4] (CAS Registry No: 5771-94-8)

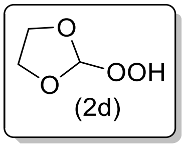

Colorless oil; ^{1}**H NMR** (400 MHz, $CDCl_3$): δ 9.13 (s, 1H), 6.09-6.01 (s, 1H), 4.17-4.09 (m, 2H), 3.98-3.95 (m, 2H); ^{13}**C NMR** (100 MHz, $CDCl_3$): δ 116.7 and 65.0.
NMR data of (2d) was identical with that in the literature[4].

1, 3-dioxolan-2-one (3d) (CAS NO: 96-49-1)

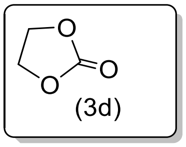

Colorless oil; **^{1}H NMR** (400 MHz, $CDCl_3$): δ4.5(s, 4H); **^{13}C NMR** (100 MHz, $CDCl_3$): δ 155.4 and 64.5. NMR data is in agreement with authentic commercially available sample.

2-hydroperoxy-1, 4-dioxane (2e) (CAS Registry No: 4722-59-2)

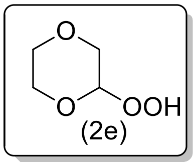

Colorless oil; **^{1}H NMR** (400 MHz, $CDCl_3$): δ 9.41 (s, 1H), 5.02 (s, 1H), 4.18-4.11 (m, 1H), 3.80-3.60 (m, 5H); **^{13}C NMR** (100 MHz, $CDCl_3$): δ 98.9, 66.1, 65.9 and 61.15. **IR (neat, cm^{-1})** 3450, 1265, 1184; **HRMS** calcd for $C_4H_8O_4$: (M+H): 105.0.

1,4-dioxan-2-one (3e)[5] (CAS NO: 3041-16-5)

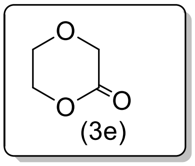

Colorless oil; **^{1}H NMR** (400 MHz, $CDCl_3$): δ 9.41 (s, 1H), 5.02 (s, 1H), 4.18-4.11 (m, 1H), 3.80-3.60 (m, 5H); **^{13}C NMR** (100 MHz, $CDCl_3$): δ 98.9, 66.1, 65.9 and 61.15.

NMR data of (**3e**) was identical with that in the literature[5].

2-hydroperoxy-2-methyltetrahydrofuran (2f) (CAS Registry No: 23277-12-5)

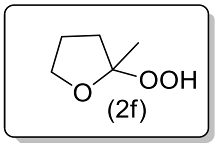

Colorless oil; **^{1}H NMR** (400 MHz, $CDCl_3$): δ 8.0 (s, 1H), 3.97-3.94 (m, 2H), 2.04-1.84 (m, 4H), 1.51 (s, 3H); **^{13}C NMR** (100 MHz, $CDCl_3$): δ 113.1, 68.7, 34.7, 25.0, and 21.6. **IR (neat, cm^{-1})** 3342, 1119; **HRMS** calcd for $C_5H_{10}O_3$: (M+H): 119.0.

2-hydroperoxy-2-(methoxymethyl)tetrahydrofuran (2g)

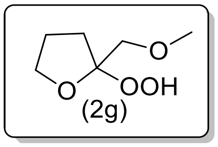

Colorless oil; ^{1}**H NMR** (400 MHz, $CDCl_3$): δ 4.21-4.20 (m, 1H), 3.65-3.64 (m, 1H), 3.38-3.36 (m, 5H), 2.48-2.44 (m, 1H), 1.91-1.78 (m, 3H); ^{13}**C NMR** (100 MHz, $CDCl_3$): δ 108.25, 75.0, 66.2, 59.2, 31.8, and 28.7.

2-(chloromethyl)-2-hydroperoxytetrahydrofuran (2h)

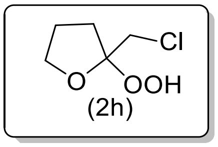

Colorless oil; ^{1}**H NMR** (400 MHz, $CDCl_3$): δ 4.05-3.90 (m, 2H), 3.83-3.55 (m, 3H), 2.09-1.87 (m, 4H), 1.45; ^{13}**C NMR** (100 MHz, $CDCl_3$): δ 112.3, 69.8, 69.4, 44.6, 44.4, 32.5, 32.3, 24.9 and 24.6.

2-hydroperoxy-2,5-dimethyltetrahydrofuran (2i) (CAS Registry No: 25258-70-2)

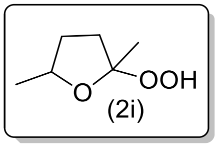

Colorless oil; ^{1}**H NMR** (400 MHz, $CDCl_3$): δ 9.11, 9.07 (s, 1H), 4.26-4.16 (m, 1H), 2.16-1.64 (m, 4H), 1.45, 1.417 (s, 3H), 1.27-1.19 (dd 3H); ^{13}**C NMR** (100 MHz, $CDCl_3$): δ 112.9, 112.8, 77.4, 76.1, 35.9, 34.7, 32.6, 32.5, 22.4, 22.1, 21.7 and 20.5.

2-hydroperoxy-2, 5-dimethoxytetrahydrofuran (2j)

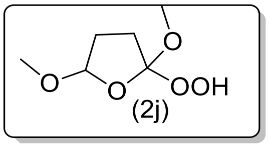

Colorless oil; ^{1}**H NMR** (400 MHz, $CDCl_3$): δ 5.80-5.77(m, 1H), 3.64(s, 3H), 3.37(s, 3 H), 2.64-2.61(m, 1H) 2.40-2.37 (m, 2H), 2.06-1.97 (m, 1H); ^{13}**C NMR** (100 MHz, $CDCl_3$): δ 130.8, 128.7, 107.4, 106.8, 56.84, 56.82, 29.43, 28.96, 28.94, 28.3.

2-hydroperoxy-2,-isopropoxypropane[6,7] (2k)

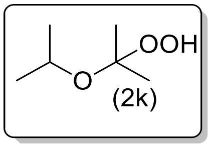

Colorless oil; **^{1}H NMR** (400 MHz, $CDCl_3$): δ 8.85(s, 1H), 4.09-3.98(m, 2H), 1.35(s, 6H), 1.97-1.81(d, 6H); **^{13}C NMR** (100 MHz, $CDCl_3$): δ 120.3, 67.3, 23.0, and 22.7.
NMR data of (2k) was identical with that in the literature[6,7].

2-phenyl butyrate (3l)[8]

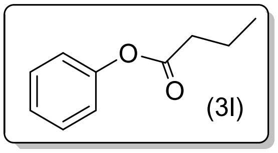

Colorless oil; **^{1}H NMR** (400 MHz, $CDCl_3$): δ 7.39-7.36 (m, 2H), 7.24-7.22 (m, 1H), 7.09-7.07(d, 2H), 2.56-2.53 (t, 2H), 1.82-1.66 (q, 2H), 1.09-1.05 (t, 3H); **^{13}C NMR** (100 MHz, $CDCl_3$): δ 173.3, 152.3, 130.2, 125.0, 122.0, 36.3, 19.0, 14.2.
NMR data of (**3l**) was identical with that in the literature[8].

2-hydroperoxy-2,-methoxymethyl)tetrahydrofuran[9] (3m) (CAS NO: 2315-68-6)

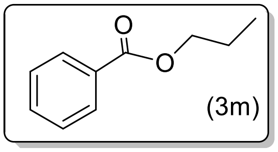

Colorless oil; **^{1}H NMR** (400 MHz, $CDCl_3$): δ 8.05-8.03 (t, 2 H), 7.55-7.43 (m, 1 H), 7.41-7.33 (m, 2 H), 4.34-4.31 (t, 2 H), 1.77-1.72 (t, 2 H), 1.58-1.47 (m, 2 H), 1.04-0.83 (t, 3 H); **^{13}C NMR** (100 MHz, $CDCl_3$): δ 166.9, 134.0, 130.5, 129.4, 128.5, 65.0, 31.3, 18.6, 14.0.
NMR data of (**3m**) was identical with commercially available authentic material and with that in the literature[9].

1-hydroperoxy-1-hydroperoxyisochroman (2n)[S10] (CAS Registry No: 2734-00-1)

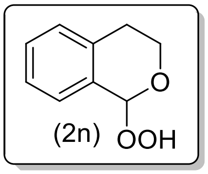

(2n)

Colorless oil; **^{1}H NMR** (400 MHz, $CDCl_3$): δ 9.35 (s, 1H), 7.44-7.36 (m, 1H), 7.30-7.21 (m, 2H), 7.15-7.13 (d, 1H), 6.36, 6.20 (s, 1H), 4.46-4.28 (t, 1H), 4.06-3.98 (m, 1H), 3.11-3.02 (m, 1H), 2.65-2.60 (m, 1H); **^{13}C NMR** (100 MHz, $CDCl_3$): δ 135.6, 135.2, 129.6, 129.3, 128.95, 128.93, 128.6, 128.5, 128.4, 128.3, 126.28, 126.23, 100.9, 99.0, 58.3, 58.0, 27.7 and 27.5.
NMR data of (2n) was identical with that in the literature[10].

Isochroman-1-one (3n)[11] (CAS NO: 4702-34-5)

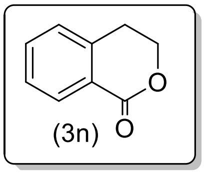

(3n)

Pale white pasty oil; **^{1}H NMR** (400 MHz, $CDCl_3$): δ 8.05-8.03 (d, 1H), 7.51-7.47 (m, 1H), 7.36-7.32 (m, 1H), 7.23-7.21 (d, 1H), 4.50-4.47 (t, 2H), 3.03-3.00 (t, 2H); **^{13}C NMR** (100 MHz, $CDCl_3$): δ 165.0, 139.4, 133.5, 130.2, 127.5, 127.1, 125.1, 67.1 and 27.6.
NMR data of (**3n**) was identical with commercially available authentic material and with that in the literature[11].

Benzofuran-3(2H)-one (3o)[12] (CAS NO: 7169-34-8)

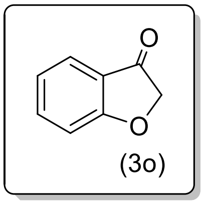

(3o)

Pale white solid; **^{1}H NMR** (400 MHz, $CDCl_3$): δ 7.66-7.64 (m, 1H), 7.61-7.57 (t, 1H), 4.60 (s, 1H); **^{13}C NMR** (100 MHz, $CDCl_3$): δ 199.8, 173.9, 137.8, 124.0, 121.9, 121.1, 113.6, and 74.6.
NMR data of (**3o**) was identical with commercially available authentic material and with that in the literature[12].

1-hydroperoxy-1,3-dihydroisobenzofuran (2p)[13] (CAS Registry No: 4676-81-7)

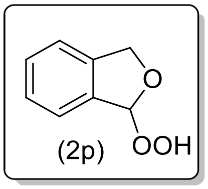

Colorless oil; **^{1}H NMR** (400 MHz, $CDCl_3$): δ 9.52 (s, 1H), 7.44-7.22 (m, 4H), 6.62, 6.21(ss, 1H), 5.28-5.04 (m, 2H); **^{13}C NMR** (100 MHz, $CDCl_3$): δ 140.3, 133.4, 129.9, 127.7, 123.5, 120.9, 110.3, and 72.8.

NMR data of (2p) was identical with that in the literature[13].

Isobenzofuran-1(3H)-one (3p)[11] (CAS NO: 87-41-2)

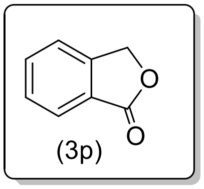

White solid; **^{1}H NMR** (400 MHz, $CDCl_3$): δ 7.87-7.85 (d, 1H), 7.86-7.62(t, 1H), 7.51-7.45 (m, 2H); **^{13}C NMR** (100 MHz, $CDCl_3$): δ 171.0, 146.4, 133.9, 128.9, 125.5, 122.06, 122.05, and 69.5. NMR data of (**3p**) was identical with commercially available authentic material and with that in the literature[11].

4-bromoisobenzofuran-1(3H)-one (3q)[14] (CAS NO: 102308-43-0)

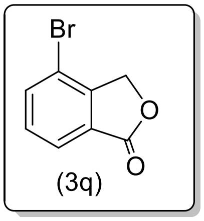

White solid; **^{1}H NMR** (400 MHz, $CDCl_3$): δ 7.78-7.76 (d, 1 H), 7.73-7.71 (d, 1H), 7.40-7.36 (t, 1H), 5.13 (s, 2H); **^{13}C NMR** (100 MHz, $CDCl_3$): δ 169.7, 146.4, 136.5, 130.7, 127.7, 124.3, 116.3, and 69.6. NMR data of (**3q**) was identical with commercially available authentic material and with that in the literature[14].

4-hydroperoxy-2,3-dihydrobenzo[b][1,4]dioxine (2r)

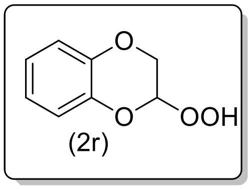

Colorless oil; ^{1}**H NMR** (400 MHz, $CDCl_3$): δ 6.89-6.87 (m, 4H), 5.54, 5.53 (s, 1H), 4.13-4.04 (m, 2H), 3.56 **(**b, 1H); ^{13}**C NMR** (100 MHz, $CDCl_3$): δ 142.72, 140.93, 122.38, 121.9, 117.71, 117.18, 88.9, and 66.6.

(3aR,5aS,9aS)-3a,6,6,9a-tetramethyldecahydronaphtho[2,1-b]furan-2(3aH)-one (3s)[15] **(**CAS NO: 102308-43-0)

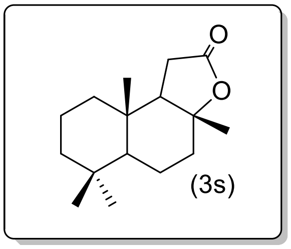

White solid; ^{1}**H NMR** (400 MHz, $CDCl_3$): δ 2.40-2.32 (q, 1H), 2.21-2.15(q, 1H), 2.04-2.00 (m, 1H), (1.95-1.89 (d, 1H), 1.85-1.81 (m, 1H), 1.67-1.59 (m, 2H), 1.43-1.40 (m, 3H),1.28 (s, 3H), 1.209-1.13 (m, 2H), 1.03-0.97 (m, 2H), 0.87 (s, 3H), 0.81(s, 3H), 0.77 (s, 3H) ; ^{13}**C NMR** (100 MHz, $CDCl_3$): δ 176.7, 86.2, 59.0, 56.5, 42.0, 39.4, 36.2, 38.6, 35.9, 33.0, 33.01, 29.5, 28.6,21.4, 20.8, 20.4, 17.9, 14.9. **IR (KBr, cm^{-1})** 2997, 2867, 2845, 1776, 1456, 1198, 1126, 950; **HRMS** calcd for $C_{16}H_{26}O_2$: 250.1933, found: 250.1930.

1-(2-(4-(2-(methoxycarbonyl)benzoyl)phenoxy)-2-oxoethyl)piperidin-1-ium chloride (3t)

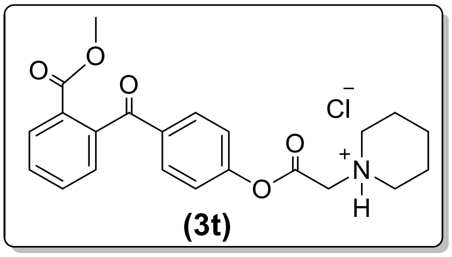

Brown solid; ^{1}**H NMR** (600 MHz, $CDCl_3$): δ 8.01 (d, J= 12.0 Hz, 1H) 7.73 (d, J= 12.0 Hz, 2H), 7.64-7.53 (m, 2 H), 7.33 (d, J= 12.0 Hz, 1H), 7.27 (d, J= 6.0 Hz, 2H), 4.30 (s, 2H), 3.74 (b, 2H), 3.62 (s, 3H), 3.38 (broad, 2H), 2.18 (broad, 2H), 1.90 (broad, 3H), 1.54 (b, 1H) ; ^{13}**C NMR** (150 MHz, $CDCl_3$): δ 195.6, 166.1, 162.7, 152.8, 141.1, 135.5, 132.5, 130.8, 130.1, 129.8, 128.8, 127.4, 121.6, 55.7, 53.8, 52.3, 22.8 and 21.0; **EI-MS** calcd for $C_{22}H_{24}NO_5^+$ (M+): 382.1649, found: 382.1650.

Preparation of pitofenone starting material (1t)[16] The synthetic route for prepration of pitofenone starting material (1t) was listed below..

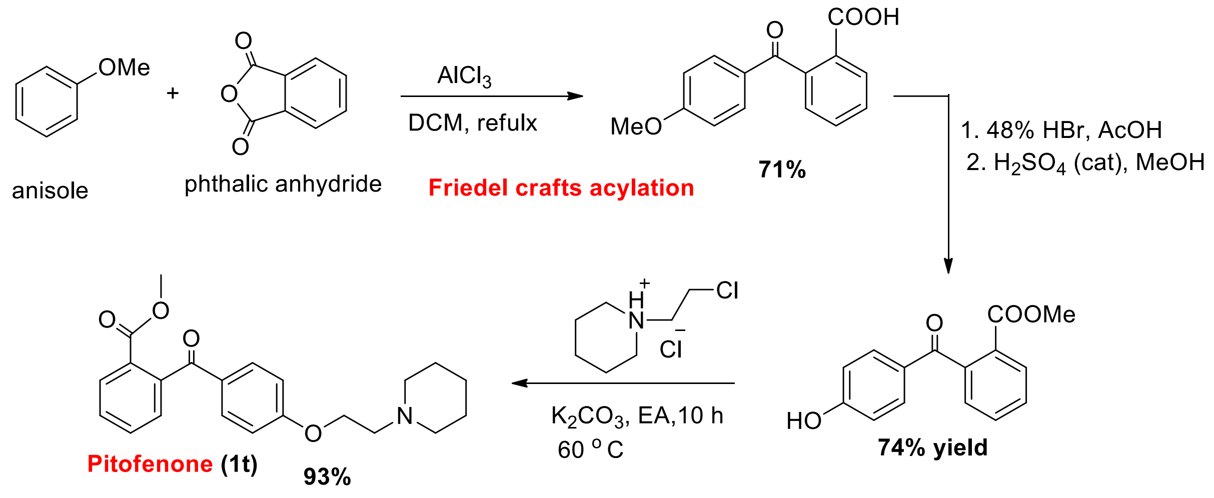

1methyl 2-(4-(2-(piperidin-1-yl)ethoxy)benzoyl)benzoate (1t)[16]

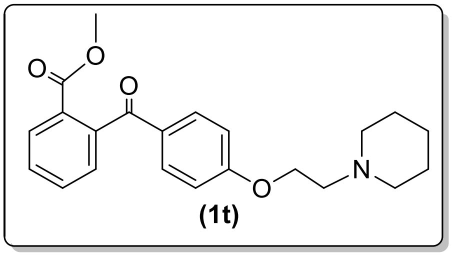

Brown solid; ^{1}**H NMR** (600 MHz, $CDCl_3$): δ 7.97 (d, J=6.0 Hz, 1H), 7.65 (d, J=6.0 Hz, 2H), 7.57-7.47 (m, 2H), 7.32 (d, J=6.0 Hz, 1H), 6.85 (d, J=6.0 Hz, 2H), 4.10 (t, J=6.0 Hz, 2H), 3.59 (s, 3H), 2.73 (t, J=6.0 Hz, 2H), 2.45 (s, 4H), 1.57-1.53 (m, 4H), 1.40 (t, J=6.0 Hz, 2H); ^{13}**C NMR**

(150 MHz, $CDCl_3$): δ 195.5, 166.3, 162.7, 141.9, 132.1, 131.4, 130.1, 129.9, 129.0, 127.5, 114.2, 66.1, 57.5, 54.9, 52.0, 25.7 and 24.0.

Preparation method of (S)-2-methyltetrahydrofuran (1v)[17,18]. 80 Miligram of CBS catalyst was combined with 1.5 mL of THF-BH_3 at 15-20 degree, to this of 2.0 g of 4-chloro-2-pentanone in 7 mL of dry THF solution and 7 mL of THF-BH_3 solution were added simultaneously via syringe. The reaction was stirred for 10 minutes at 15-20 degree and the reaction mixture was quenched by dilute acetic water, the product (**1u**) was separated by layer separation.

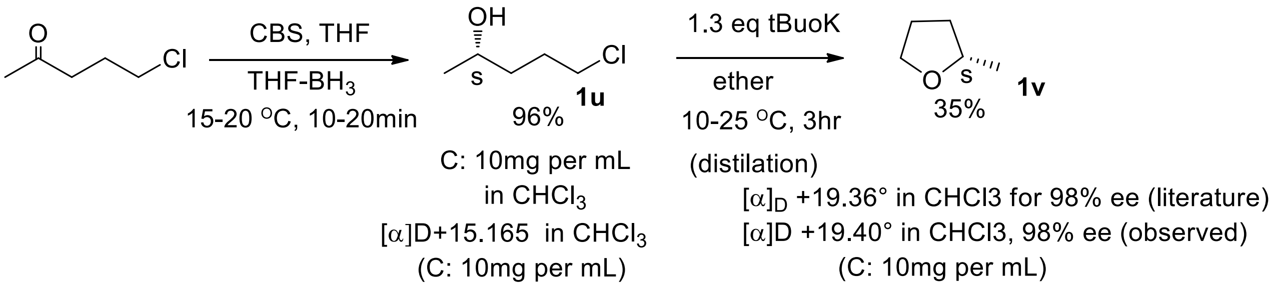

2.0 Gram of (S)-5-chloropentan-2-ol (**1u**) was dissolved in diethyl ether and cooled to 10 degree, slowly added 1.3 eqv. of t-BuOK at 10 degree. And then the reaction mixture was maintained at 20-25 degree for 3 hrs. After 3 hrs, the (S)-2-methyltetrahydrofuran (**1v**) was collected under atmospheric distillation.

(*S*)-5-chloropentan-2-ol (1u)[17,18] (CAS NO: 99212-19-8)

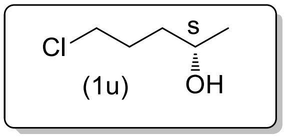

Pale brown oil; **^{1}H NMR** (400 MHz, $CDCl_3$): δ 3.83-3.78 (m, 1 H), 3.56-3.52 (t, 2 H), 1.91-1.76 (m, 2 H), 1.61-1.50 (m, 3 H), 1.19-1.17 (d, 3 H); **^{13}C NMR** (100 MHz, $CDCl_3$): δ 67.4, 45.1, 36.2, 28.8, and 23.6. SOR: $[\alpha]_D$ +15.165° in $CHCl_3$ (10 mg/1 mL $CHCl_3$).

Spectroscopic data of (1u) was identical with that in the literature[17,18]

(*S*)-2-methyltetrahydrofuran (1v)[18]

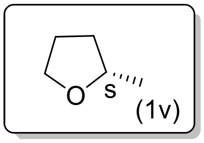

Colorless oil; ^{1}**H NMR** (400 MHz, $CDCl_3$): δ 3.92-3.82 (m, 2 H), 3.69-3.65 (m, 1 H), 1.96-1.81 (m, 3 H), 1.39-1.34 (m, 1 H), 1.19-1.18 (d, 3 H); ^{13}**C NMR** (100 MHz, $CDCl_3$): δ 75.1, 67.6, 33.0, 25.8, and 20.9. SOR: $[\alpha]_D$ +19.400° in $CHCl_3$ (10 mg/1ml $CHCl_3$) Spectroscopic data of (1v) was identical with that in the literature[18].

(*R*)-2-hydroperoxy-2-methyltetrahydrofuran (2v)

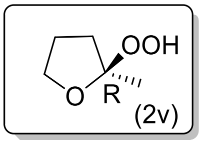

Colorless oil; ^{1}**H NMR** (400 MHz, $CDCl_3$): δ 8.1(s, 1 H), 3.97-3.94 (t, 3 H), 2.04-1.84 (m, 4 H), 1.52 (s, 3 H); ^{13}**C NMR** (100 MHz, $CDCl_3$): δ 113.1, 68.7, 34.7, 25.0, and 21.6.
SOR: $[\alpha]_D$ -12.626° in $CHCl_3$ (10 mg/1 mL $CHCl_3$).

Measurements of enantiomeric excess (ee) by chiral gas chromatography.

Instrumentation: Gas Chromatography analysis were carried out using GC-2014 SHIMADZU gas-chromatograph (serial no: C11484301285SA) equipped with a FID detector and a fused silica capillary column. The carrier gas was high purity grade nitrogen (N_2).

Column: Fused silica tubing (undeactivated, untreated) with 60 m x 0.53 mm (serial no: 1406465) was purchased from RESTEK GC Columns. Prior to use, the column was washed with n-hexane, chloroform (purified by basic alumina), acetone, and water. The cleaning procedure was repeated once in a reverse order.

***Coating of column*:** In a typical coating procedure, 15.1 mg of nickel(II) bis[(1*R*)-3-(hepta-fluorobutyryl)camphorates] and 200 mg of squalane (purchased from Alfa Aesar) were dissolved in 3.5 mL acid-free, high purity grade chloroform, and mixed well by sonication A 60 m x 0.53 mm fused silica capillary column was coated with this solution at 0.6 atm. of N_2 (over pressure) at 30 ^{O}C. The N_2 pressure was maintained for 5 hr after the coating solution was injected through the column. The column was connected to the gas chromatograph and conditioned at

0.3 atm. (overpressure) N_2 with the column temperature being raised from 30 to 100 ^{O}C and then maintained there for 12 h with the exit end left open.

Analysis condition: Column temperature: 80 ^{O}C, injection temperature: 150 ^{O}C, injection split ratio was 1:50, injection volume: 1.0 μL, column flow: the over pressure of the carrier gas was 0.3-0.5 atm. n-Octane was used as a non-coordinating reference standard.

Preparation of chiral Nickel(II) bis[(1R)-3-(hepta-fluorobutyryl)camphorates]-complex[19]: sodium (1R)-3-(Heptafluorobutyryl)camphorate. 0.3 Gram sample of 80% sodium hydride suspension in paraffin (1 0 mmol of NaH) was washed under nitrogen with dry benzene until the paraffin was completely removed. The residue was suspended in benzene and transferred into the reaction flask. 2.5 Gram (7.2 mmol) of (l*R*)-3-(heptafluorobutyryl) camphor was dissolved in 80 mL of dry benzene, and then added to the suspension of sodium hydride in benzene. The mixture was stirred for 3 h under nitrogen at 30-35 ^{o}C, and then concentrated in vacuum using rotary evaporator. The residue was dissolved in warm chloroform, and the solution was filtered. The filtrate was diluted (1:1) with dry ether with vigorous mixing by stirring. The mixture was allowed to cool down to 5 ^{o}C, leading to gradual formation of precipitates. The solid was isolated by filtration and re-precipitated from chloroform/ether. The final product was dried at high vacuum, yielding 2.45 g of product which is equivalent to a 92% yield.

Nickel(II) bis[(1*R*)-3-(heptafluorobutyryl)camphorate]. 2 Gram (5.4 mmol) sample of sodium (1R)-3-(heptafluorobutyryl)camphorate was dissolved in 50 mL of dry ethanol. Then 0.37 g (2.85 mmol) of anhydrous powdered nickel(II) chloride was added, followed by refluxing the mixture for 12 h. The green solution was filtered to remove sodium chloride as a by-product, then concentrated the filtrate, and the residue was dried at high vacuum. Yield: 1.3 g (32%) of a pale green glassy powder.

Since the molar optical rotational angle value of the as-produced chiral 2-hydroperoxyl-2-methyl-THF is not available. We have followed a procedure from the literature[*S19*] to determine the enantiomeric excess (ee) of the chiral 2-hydroperoxyl-2-methyl-THF (**2v**) by using a chiral gas chromatography (GC) (chiral Ni-complex coated with column). From the enantiomeric resolution by chiral GC, we have calculated the enantiomeric excess (ee) for (*R*)-1-methyl-THF-hydroperoxide is to be a 96.44%.

(a)

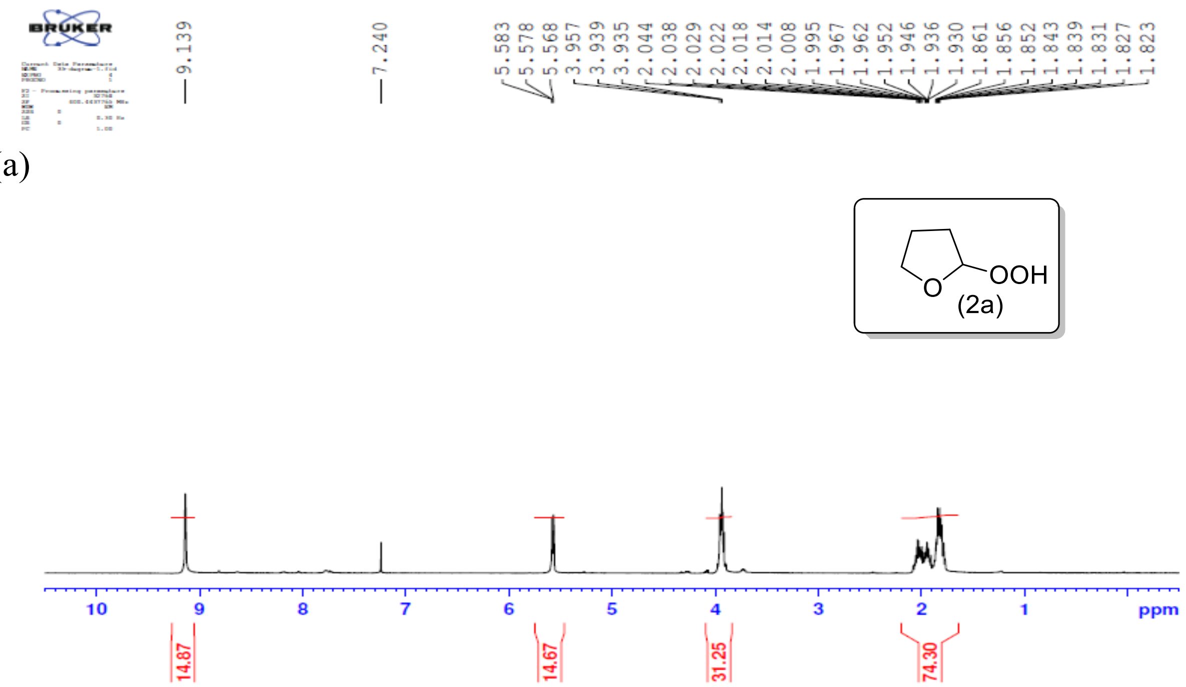

(b)

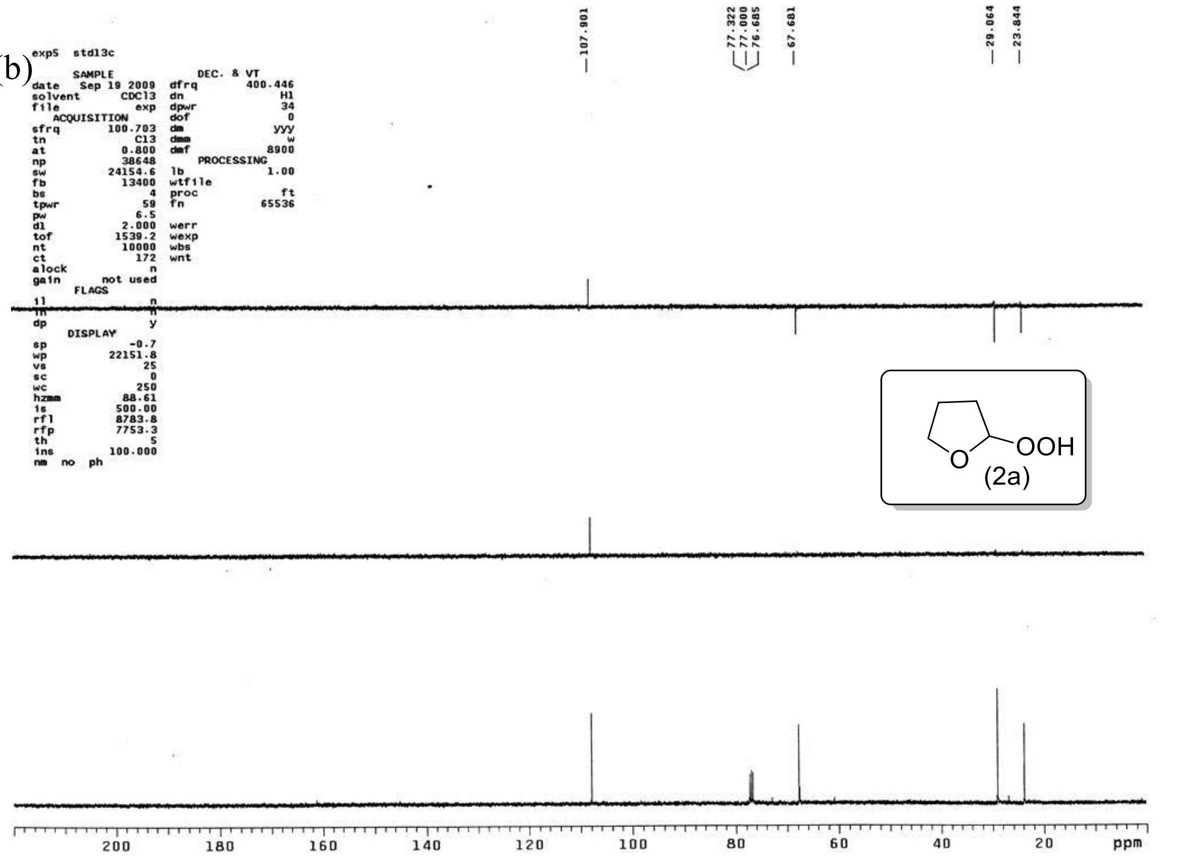

Supplementary Figure 1. NMR spectra of compound **2a**, (a) ^{1}H NMR, and (b) ^{13}C NMR spectra.

(a)

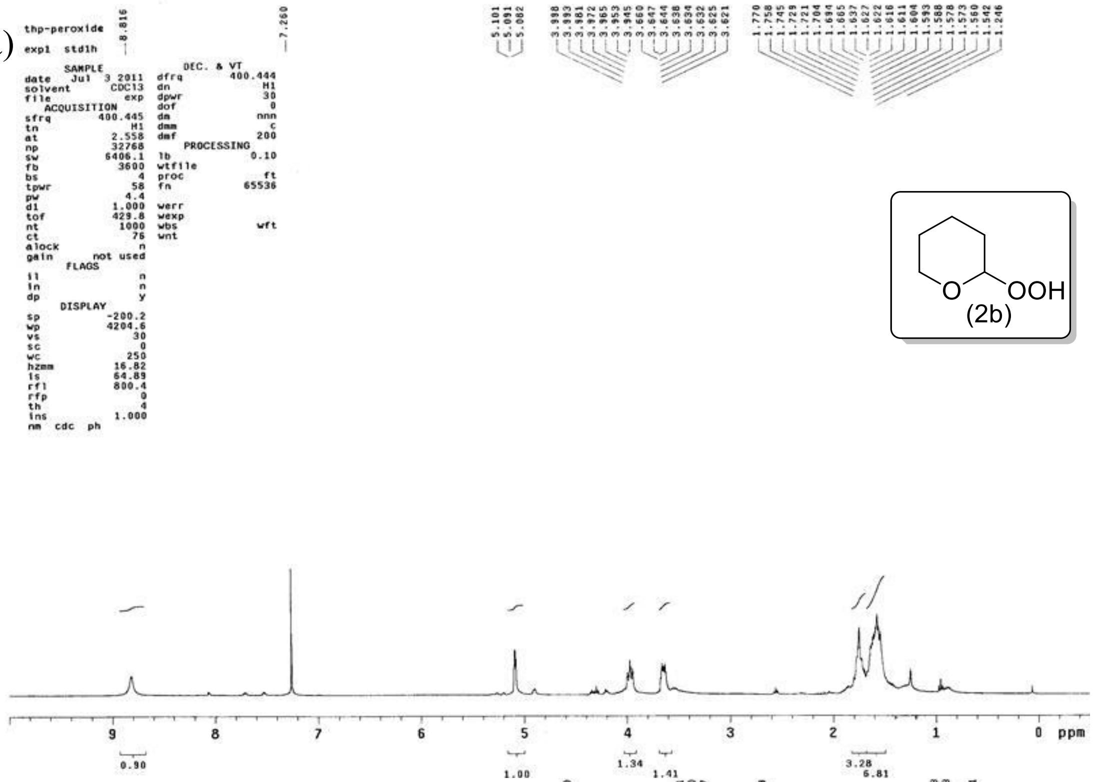

(b)

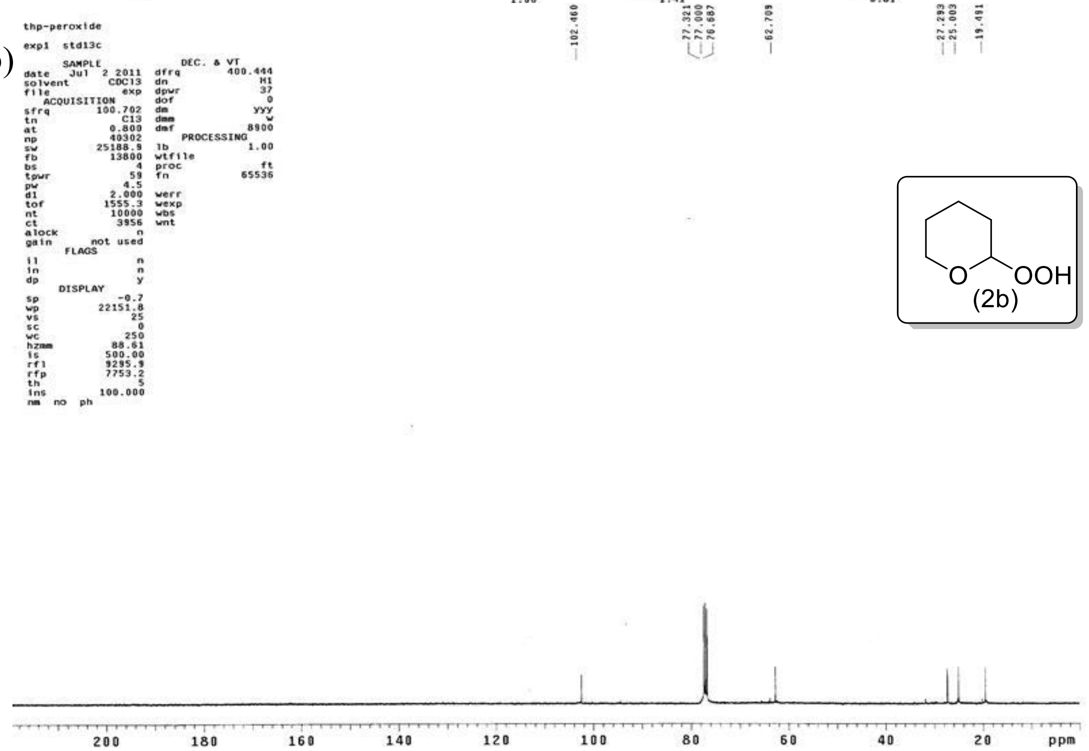

Supplementary Figure 2. NMR spectra of compound **2b**, (a) ^{1}H NMR, and (b) ^{13}C NMR spectra.

(a)

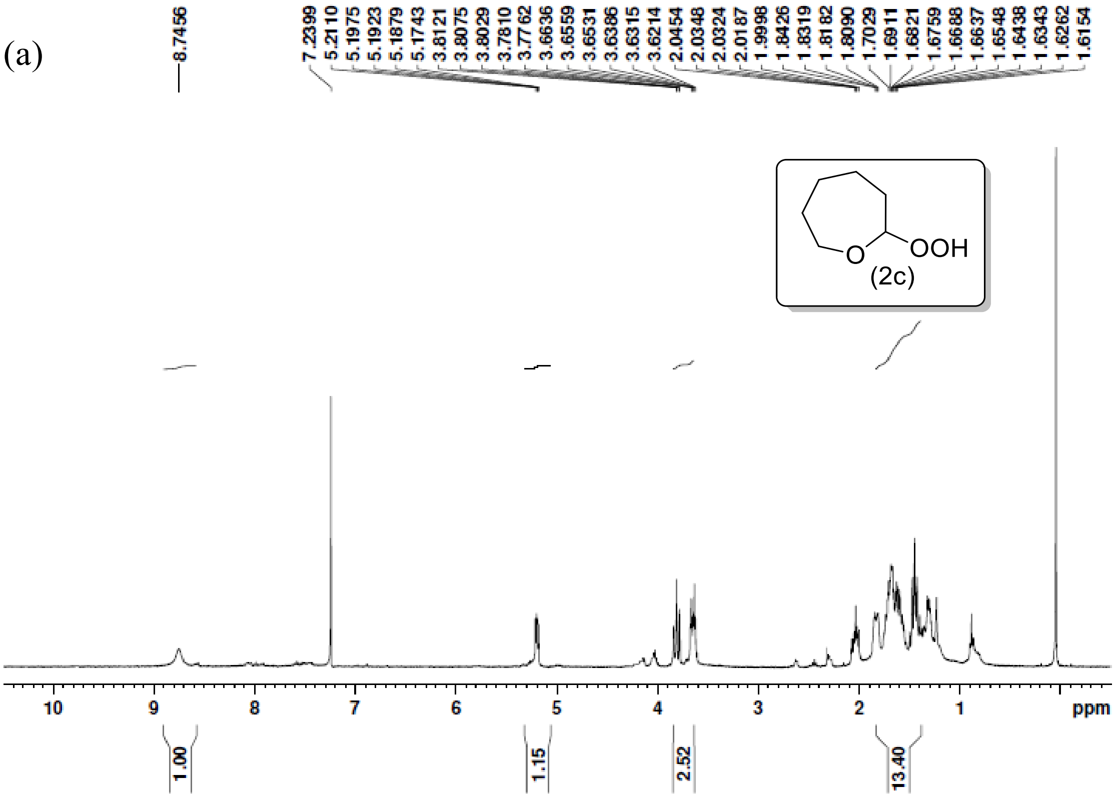

(b)

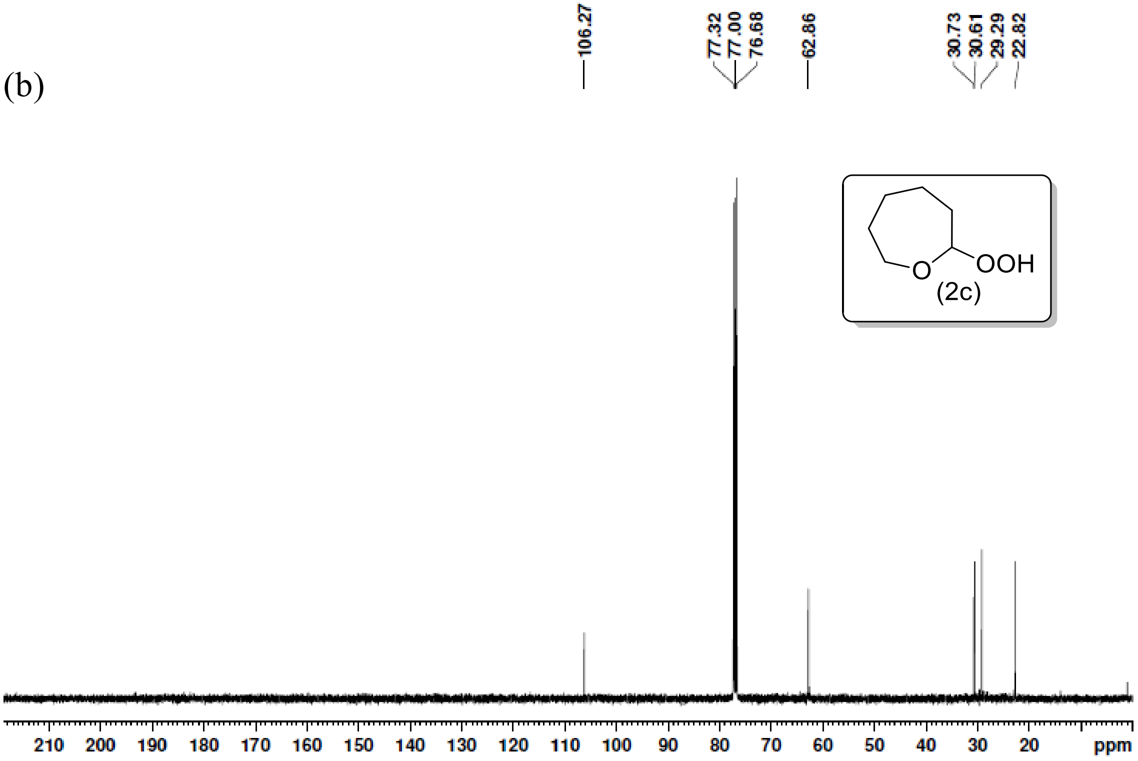

Supplementary Figure 3. NMR spectra of compound **2c**, (a) ^{1}H NMR, and (b) ^{13}C NMR spectra.

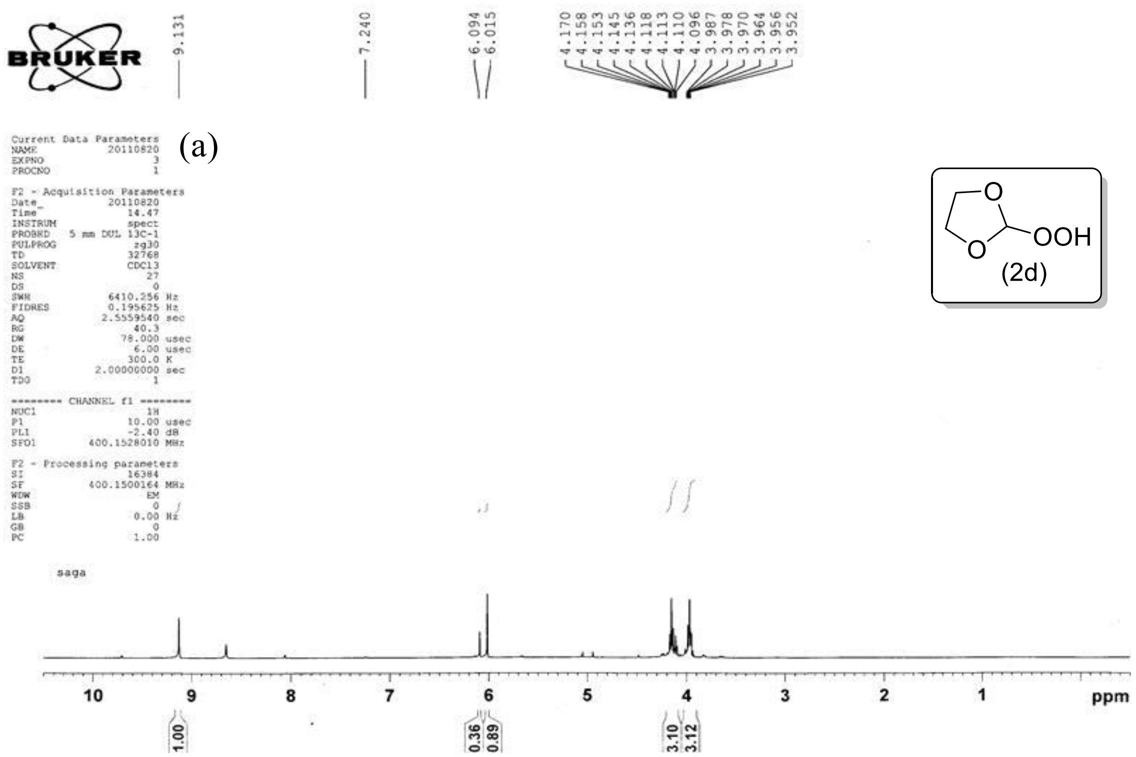

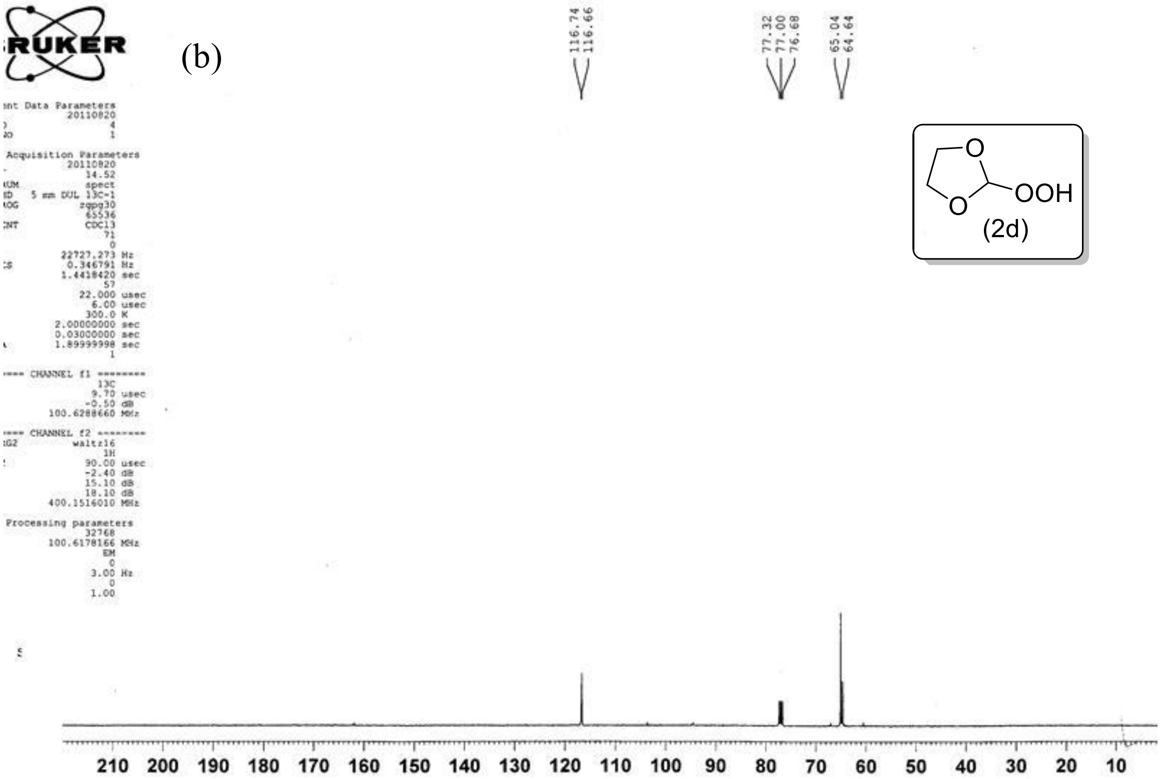

Supplementary Figure 4. NMR spectra of compound **2d**, (a) ^{1}H NMR, and (b) ^{13}C NMR spectra.

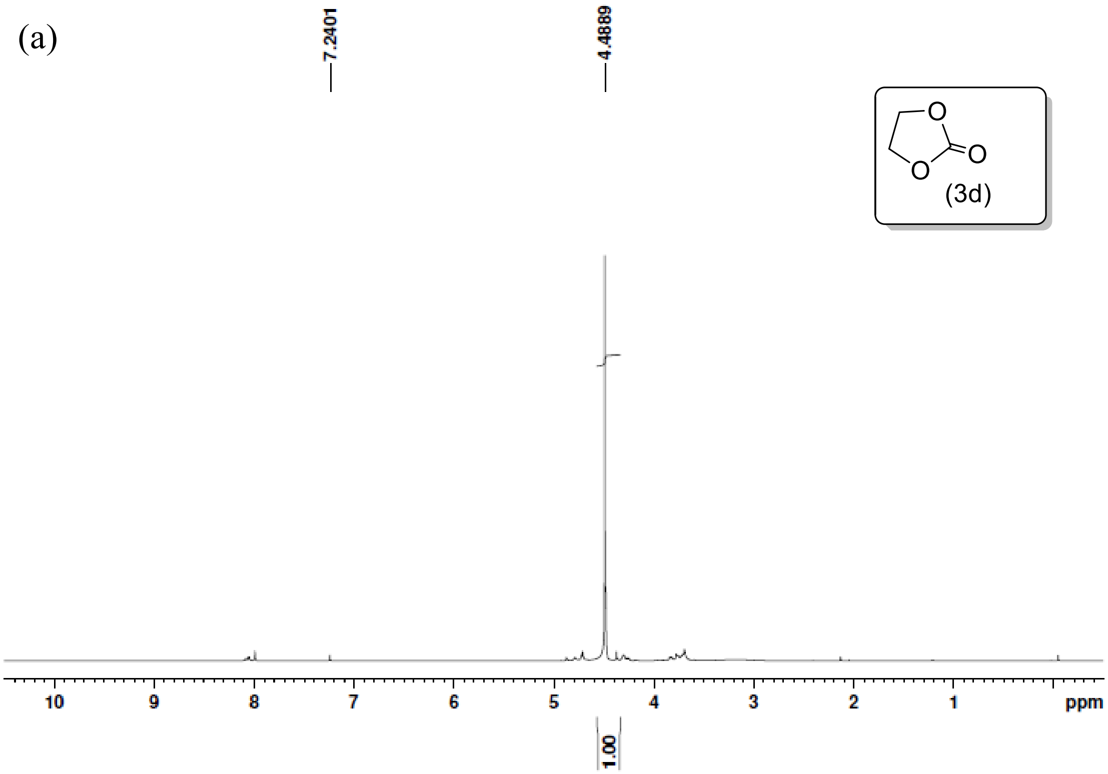

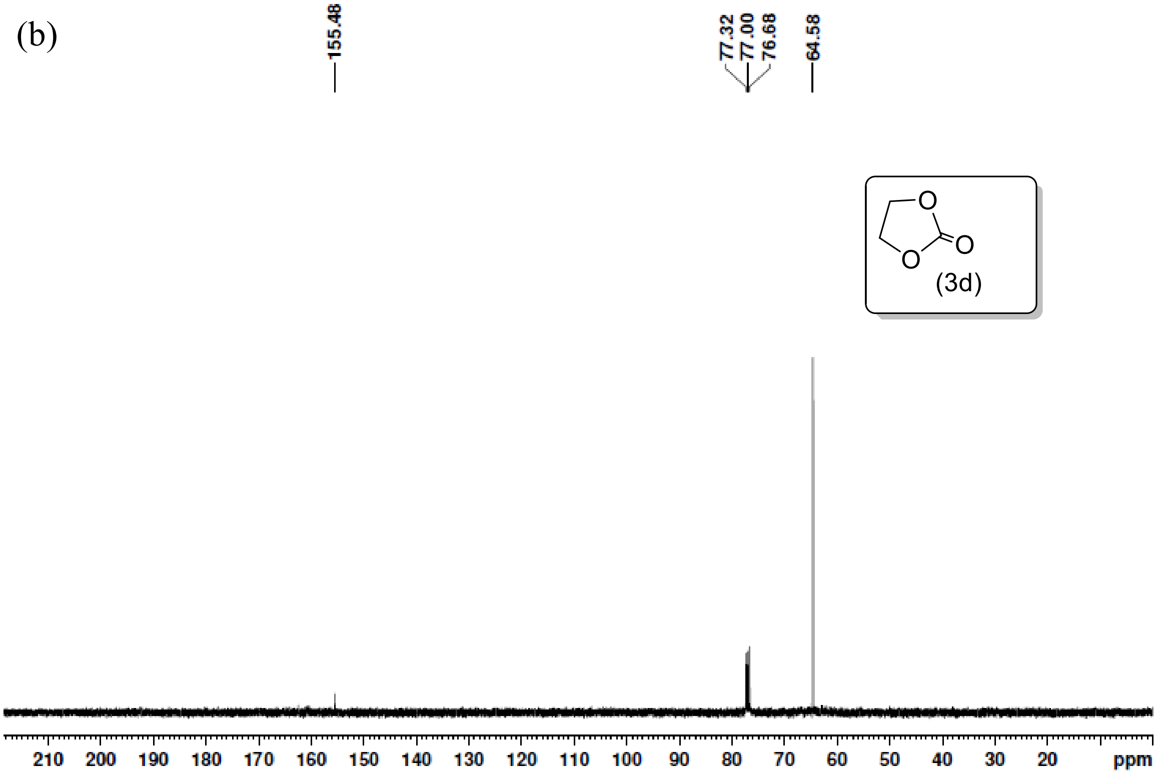

Supplementary Figure 5. NMR spectra of compound **3d**, (a) ^{1}H NMR, and (b) ^{13}C NMR spectra.

(a)

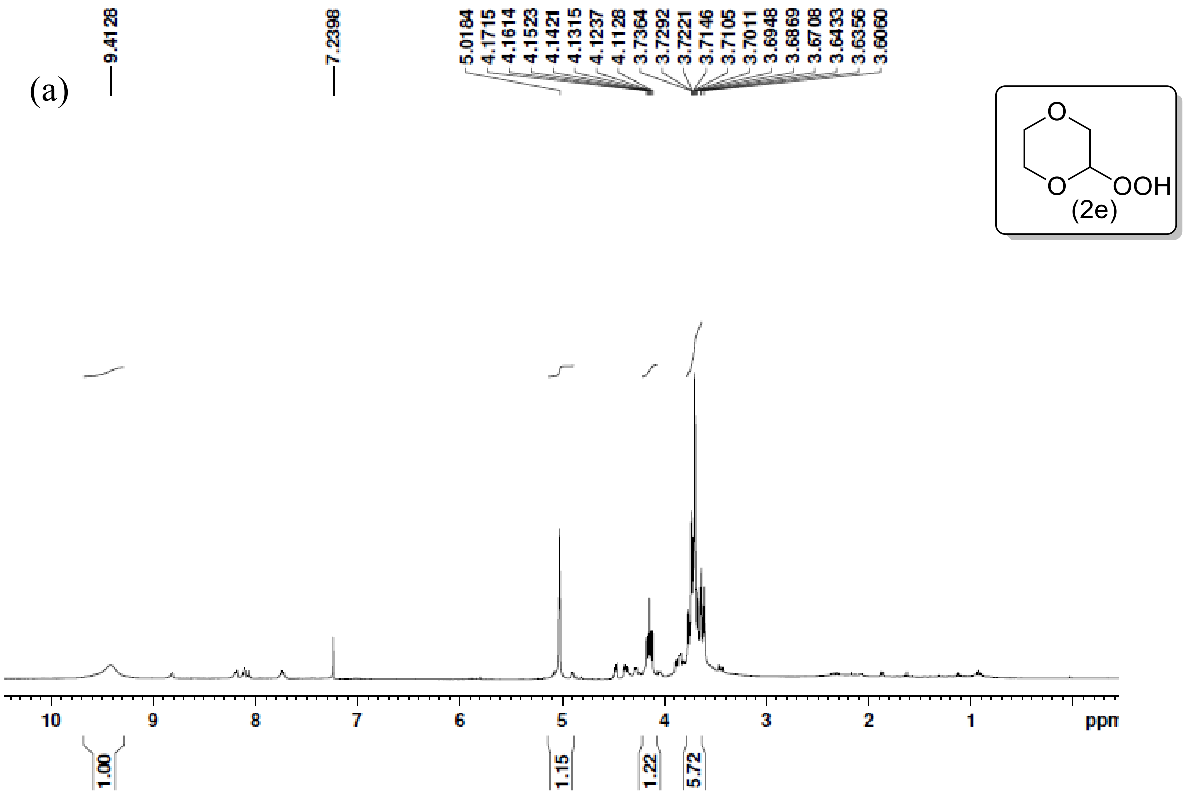

(b)

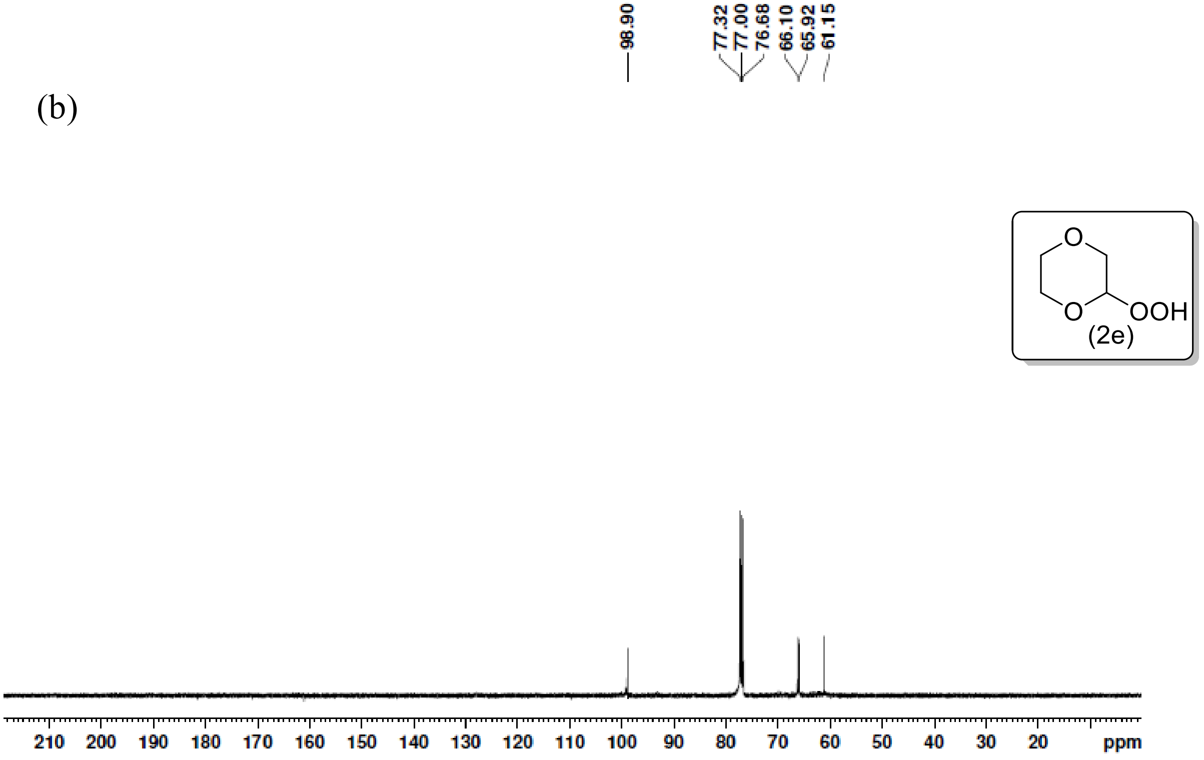

Supplementary Figure 6. NMR spectra of compound **2e**, (a) ^{1}H NMR, and (b) ^{13}C NMR spectra.

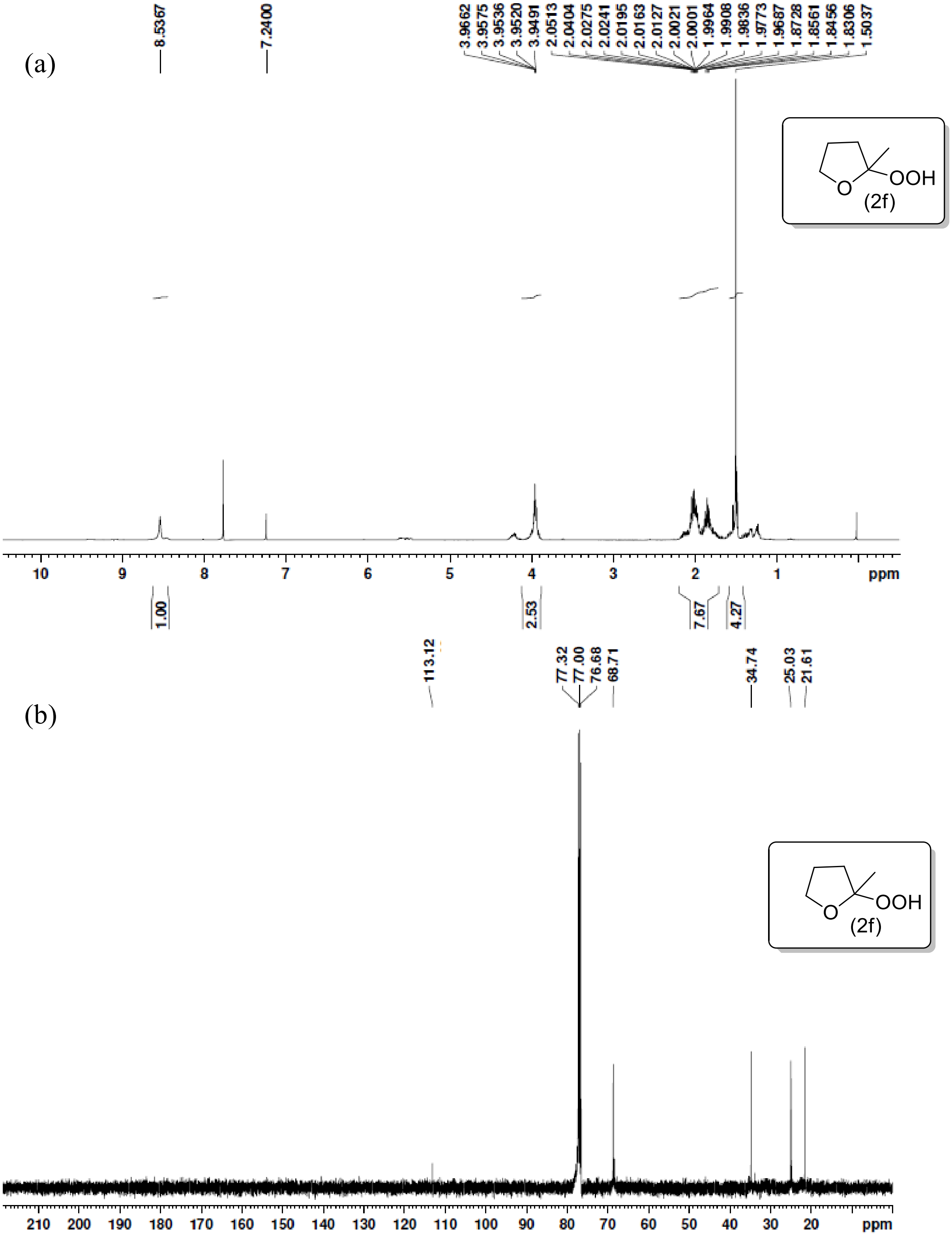

Supplementary Figure 7. NMR spectra of compound **2f**, (a) ^{1}H NMR, and (b) ^{13}C NMR spectra.

(a)

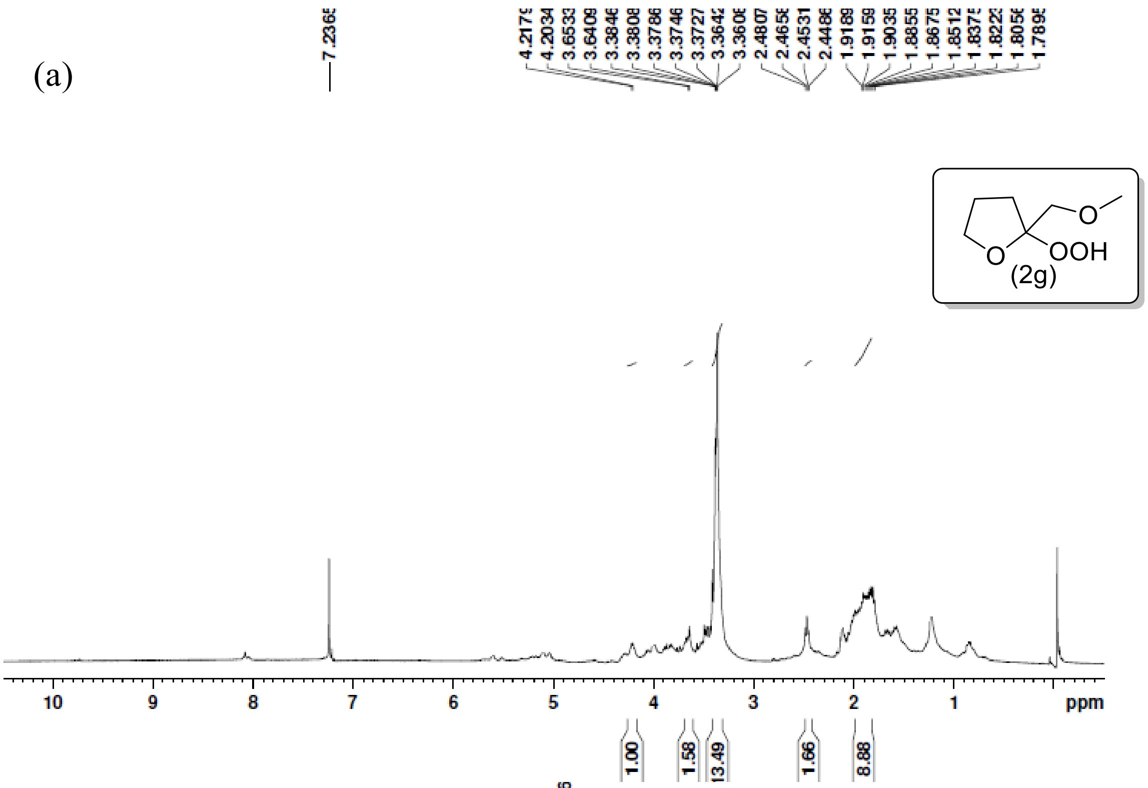

(b)

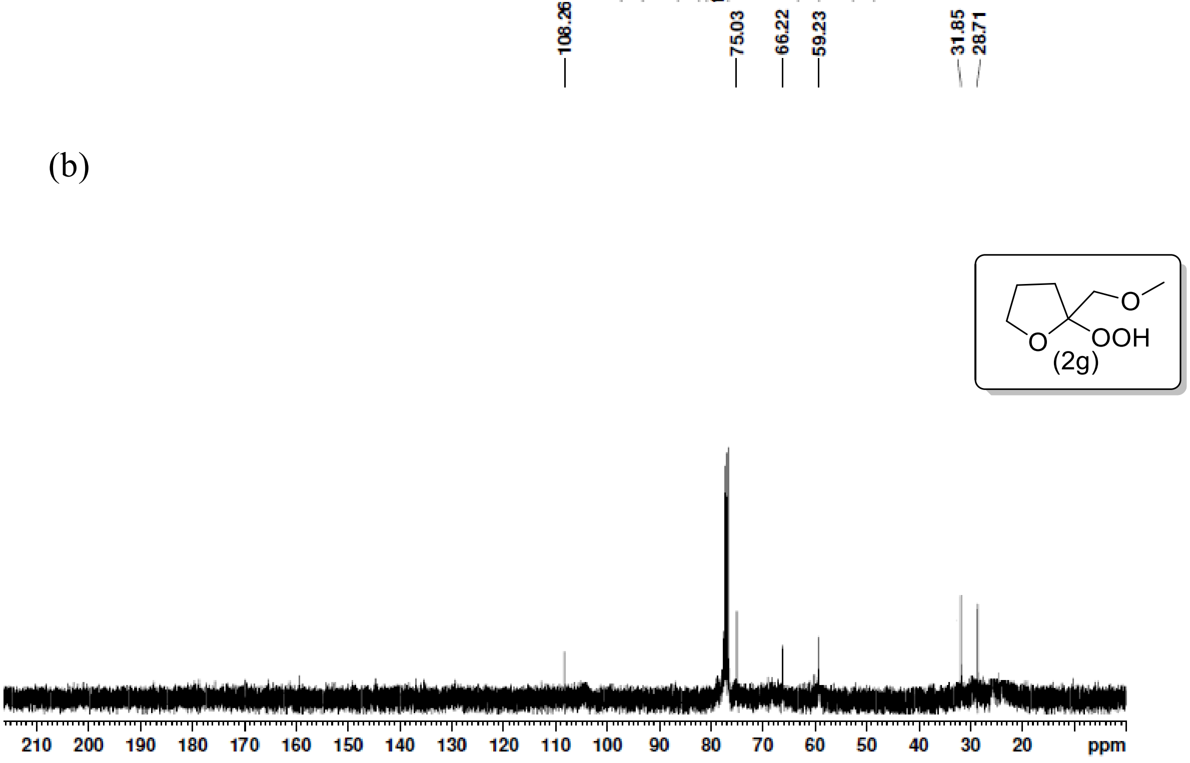

Supplementary Figure 8. NMR spectra of compound **2g**, (a) ^{1}H NMR, and (b) ^{13}C NMR spectra.

(a)

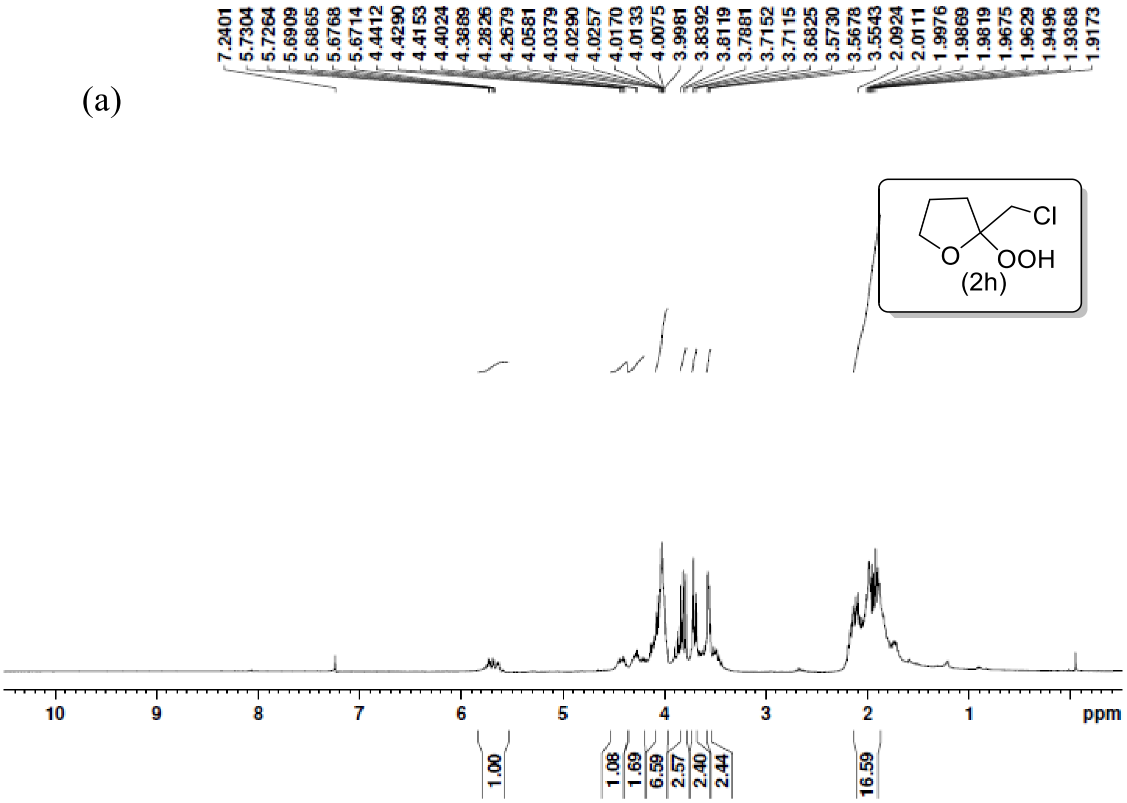

(b)

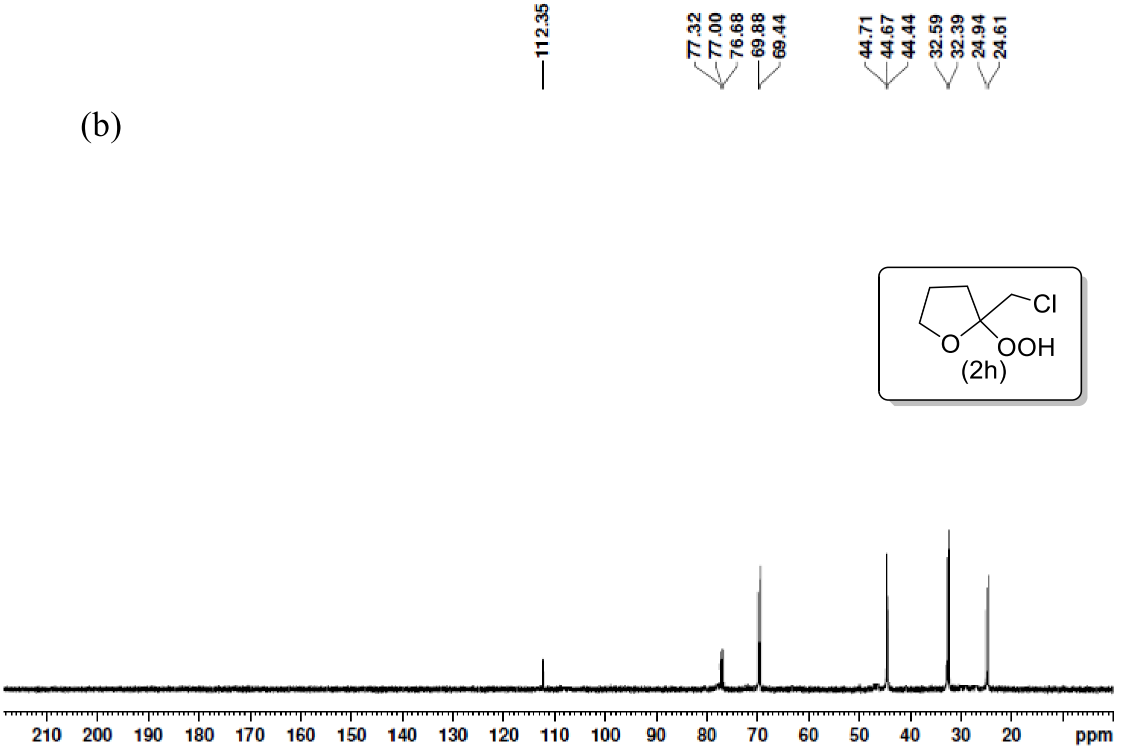

Supplementary Figure 9. NMR spectra of compound **2h**, (a) ^{1}H NMR, and (b) ^{13}C NMR spectra.

(a)

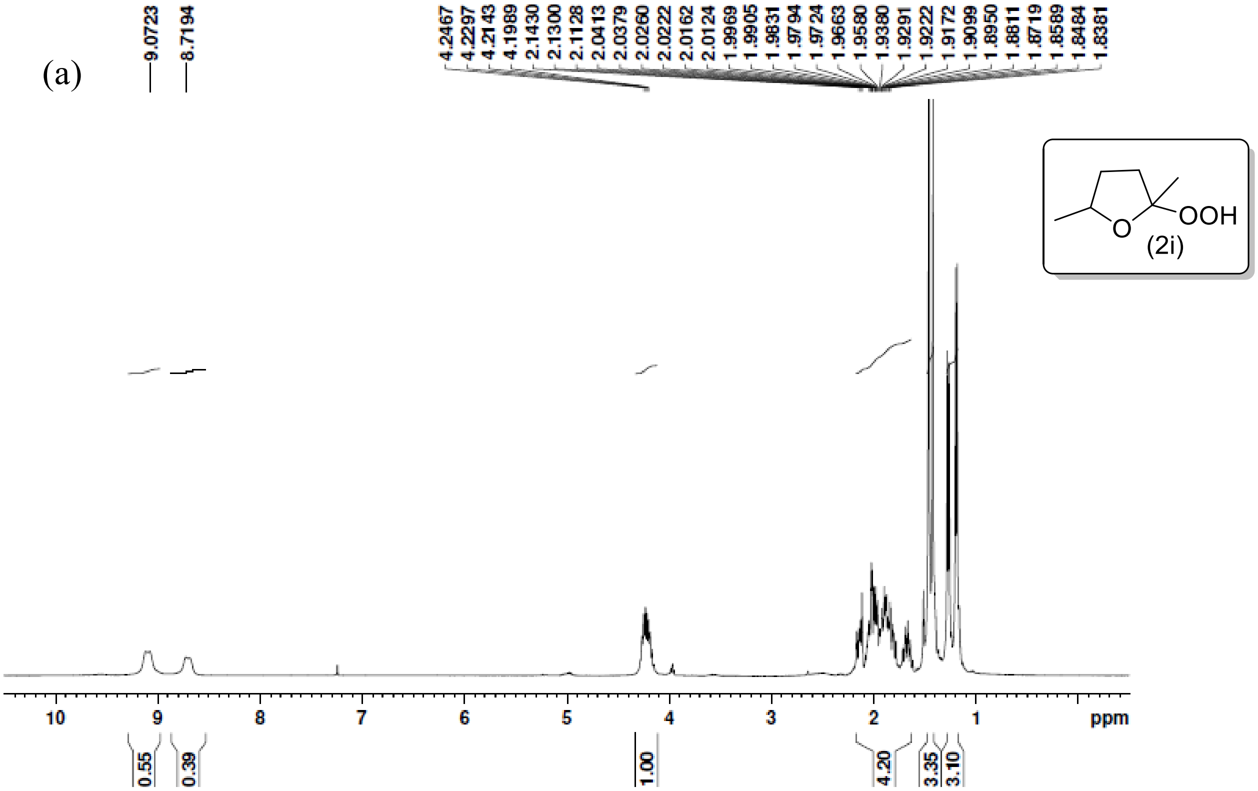

(b)

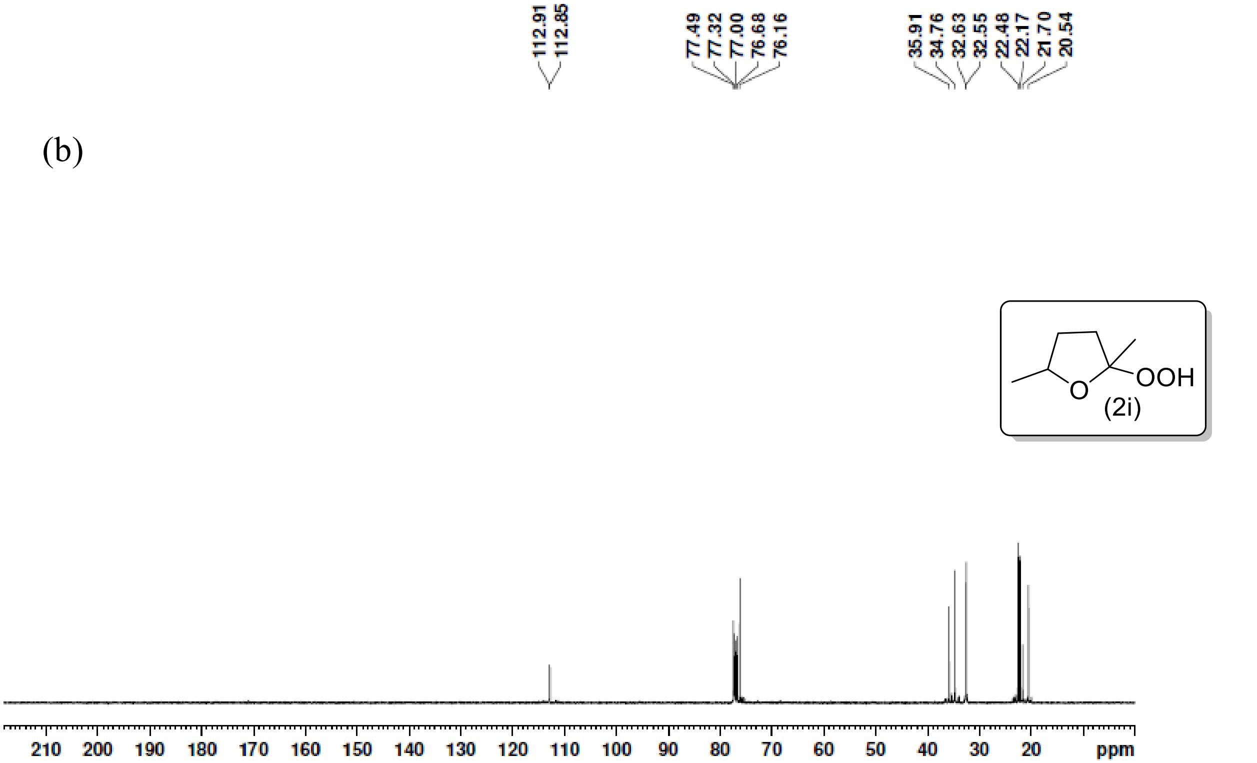

Supplementary Figure 10. NMR spectra of compound **2i**, (a) ^{1}H NMR, and (b) ^{13}C NMR spectra.

(a)

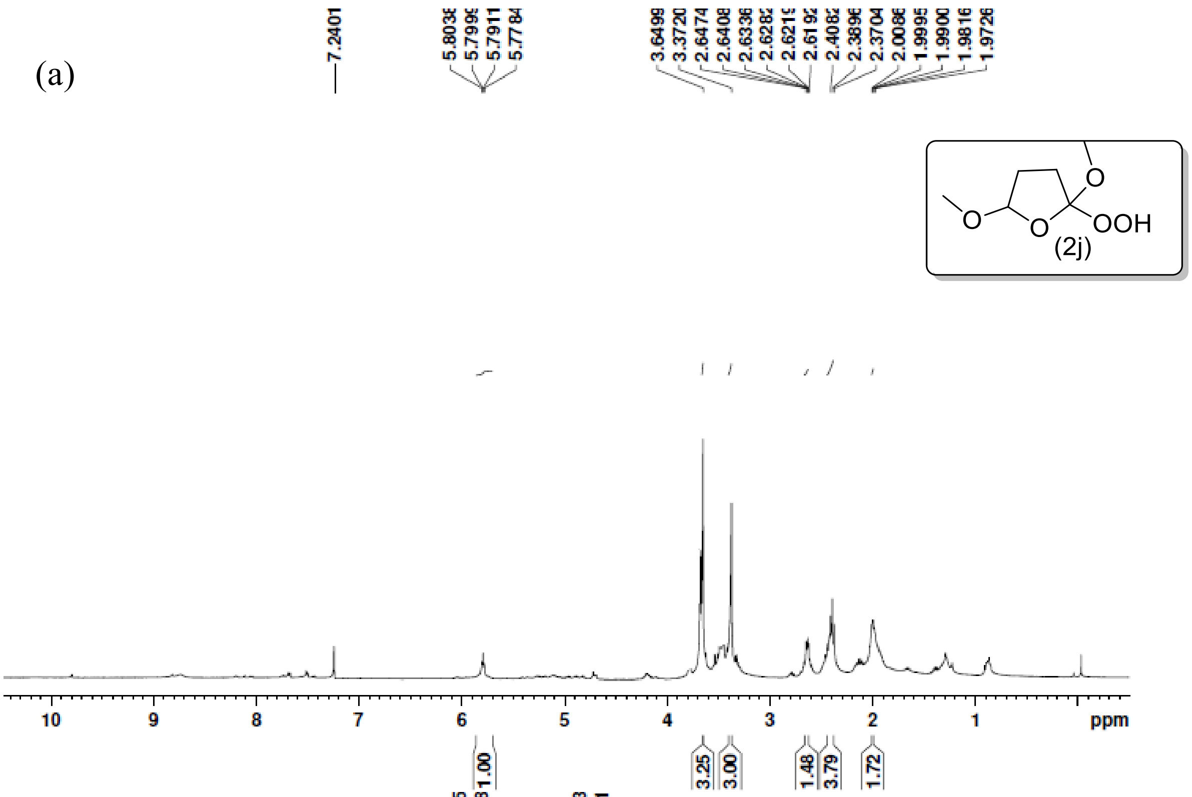

(b)

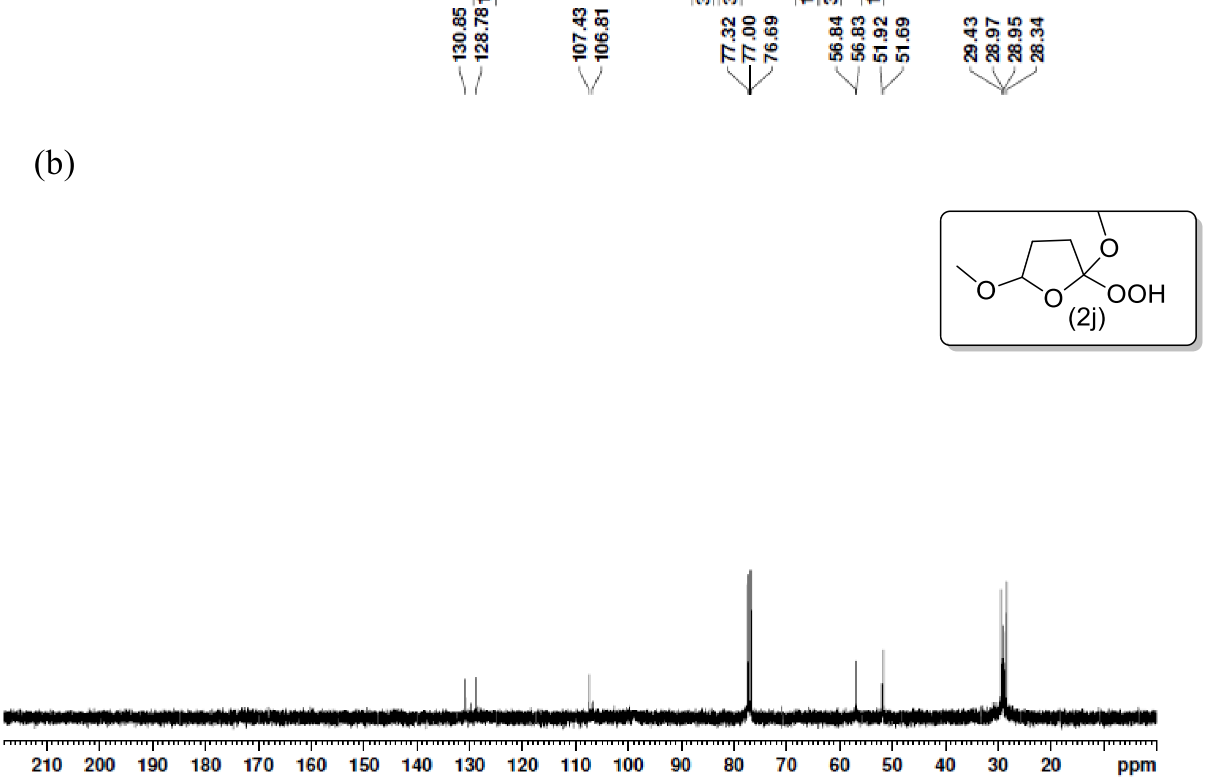

Supplementary Figure 11. NMR spectra of compound **2j**, (a) ^{1}H NMR, and (b) ^{13}C NMR spectra

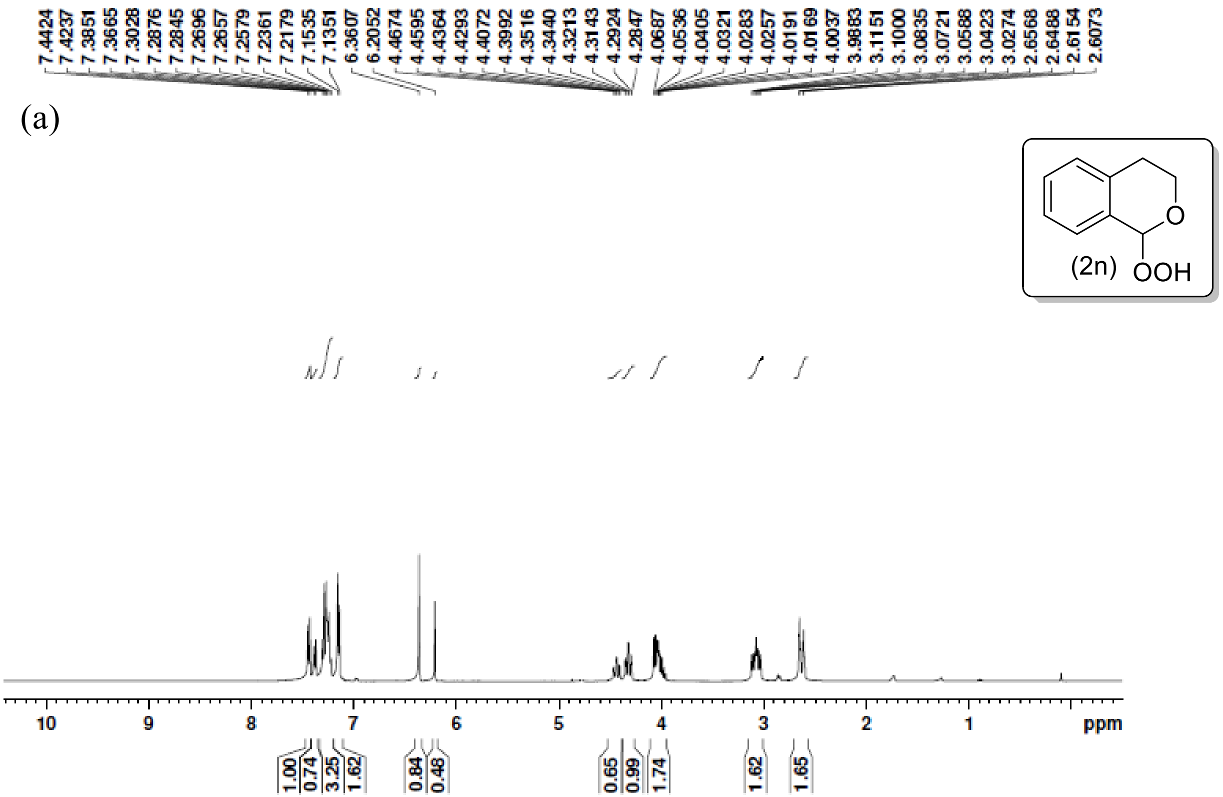

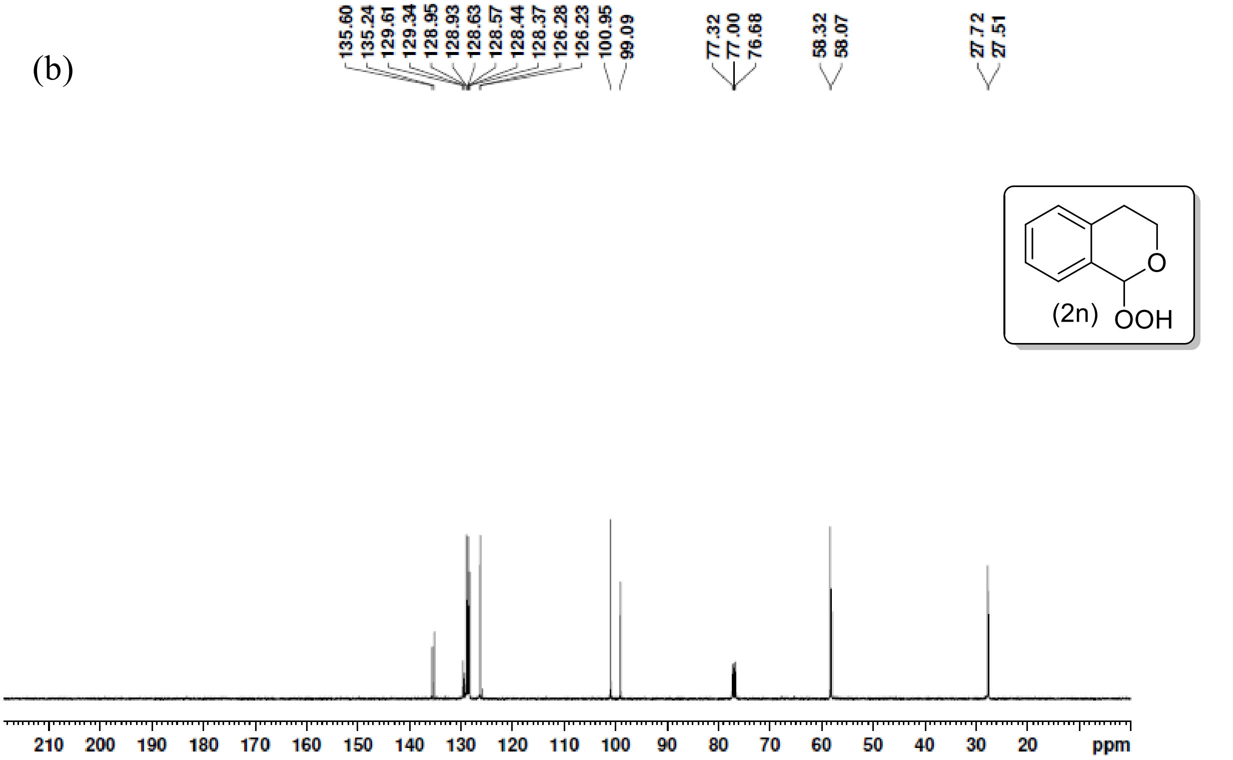

Supplementary Figure 12. NMR spectra of compound **2n**, (a) ^{1}H NMR, and (b) ^{13}C NMR spectra

(a)

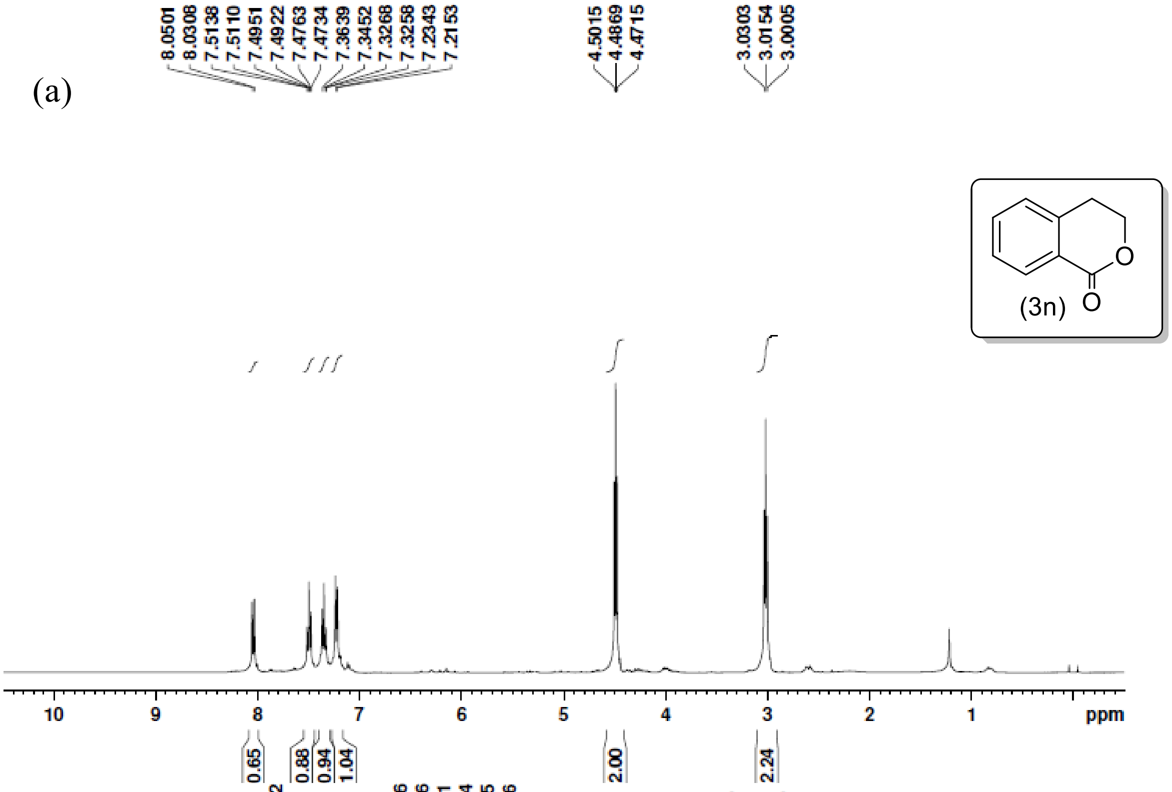

(b)

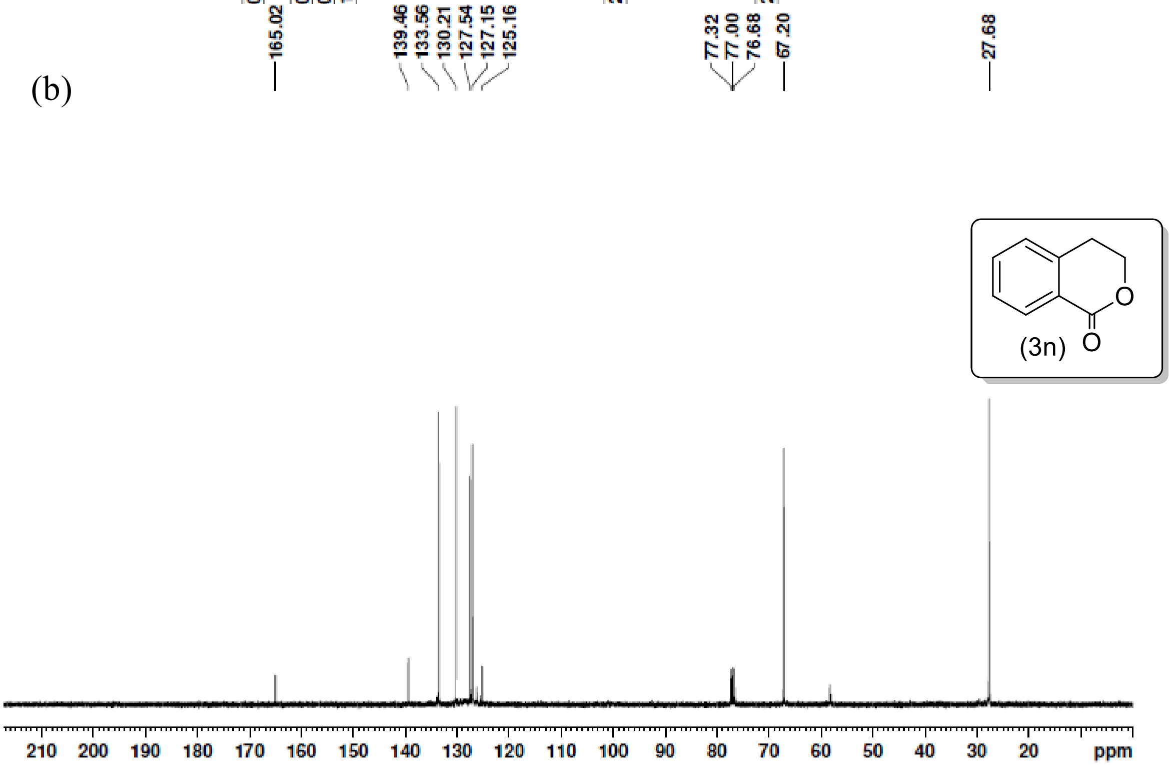

Supplementary Figure 13. NMR spectra of compound **3n**, (a) ^{1}H NMR of compound **3n**, and (b) ^{13}C NMR of compound **3n**.

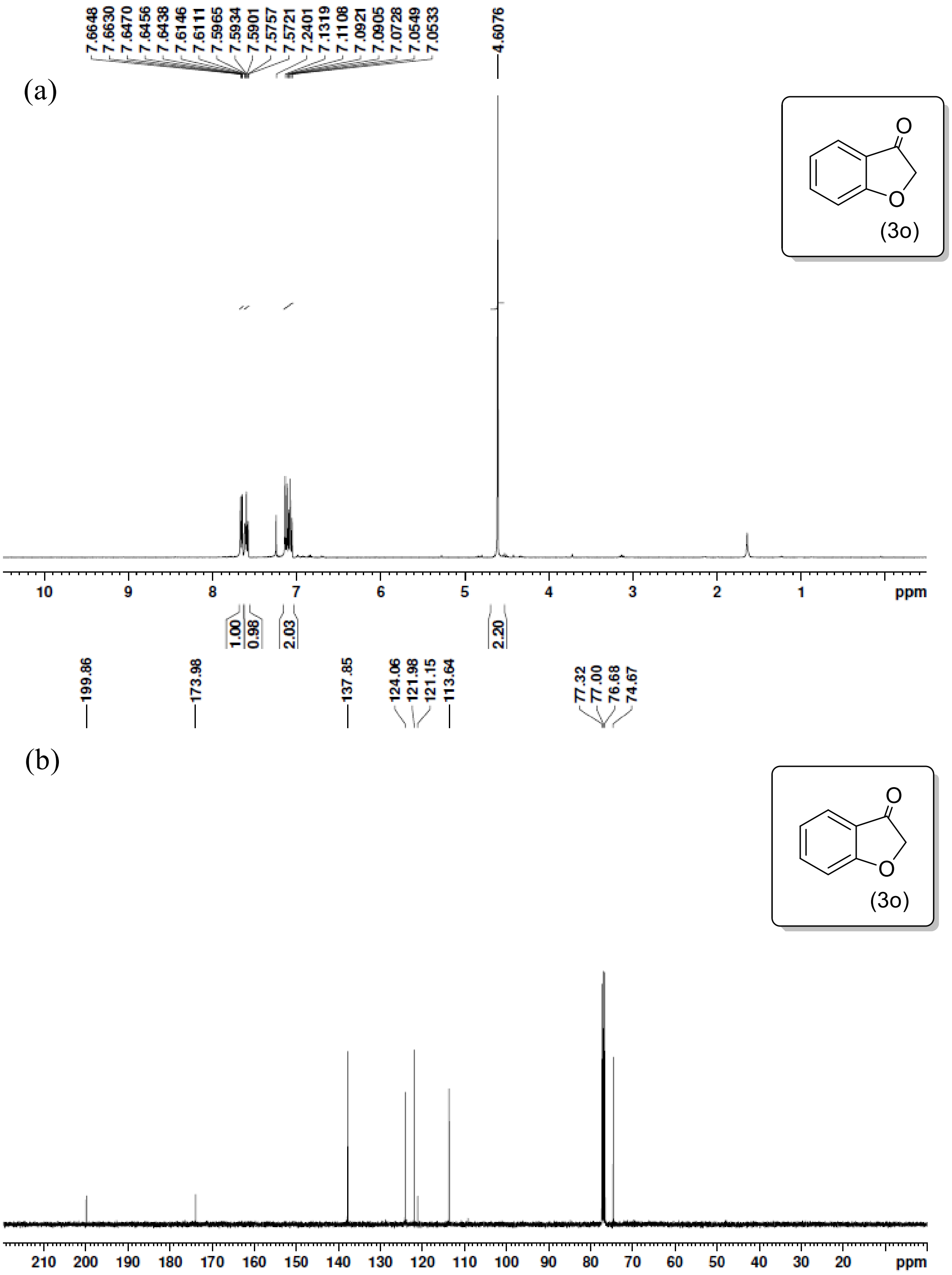

Supplementary Figure 14. . NMR spectra of compounds **3o**, (a) ^{1}H NMR of compound **3o**, and (b) ^{13}C NMR of compound **3o**.

(a)

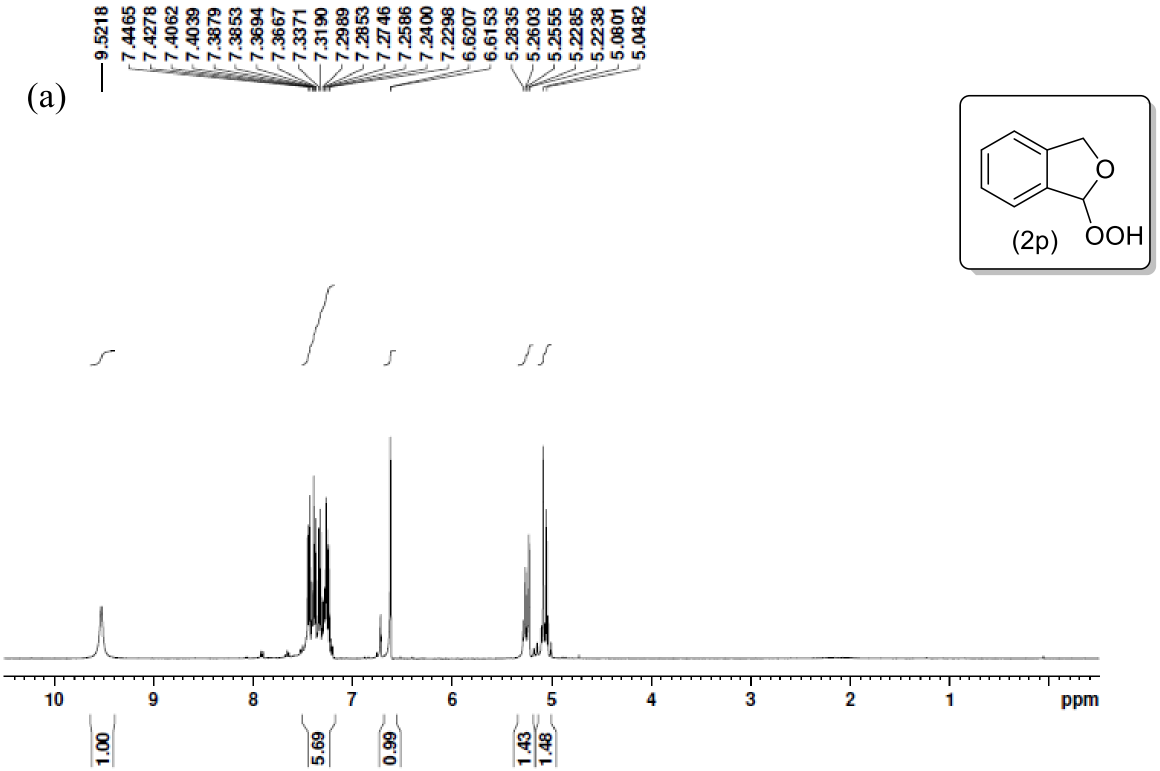

(b)

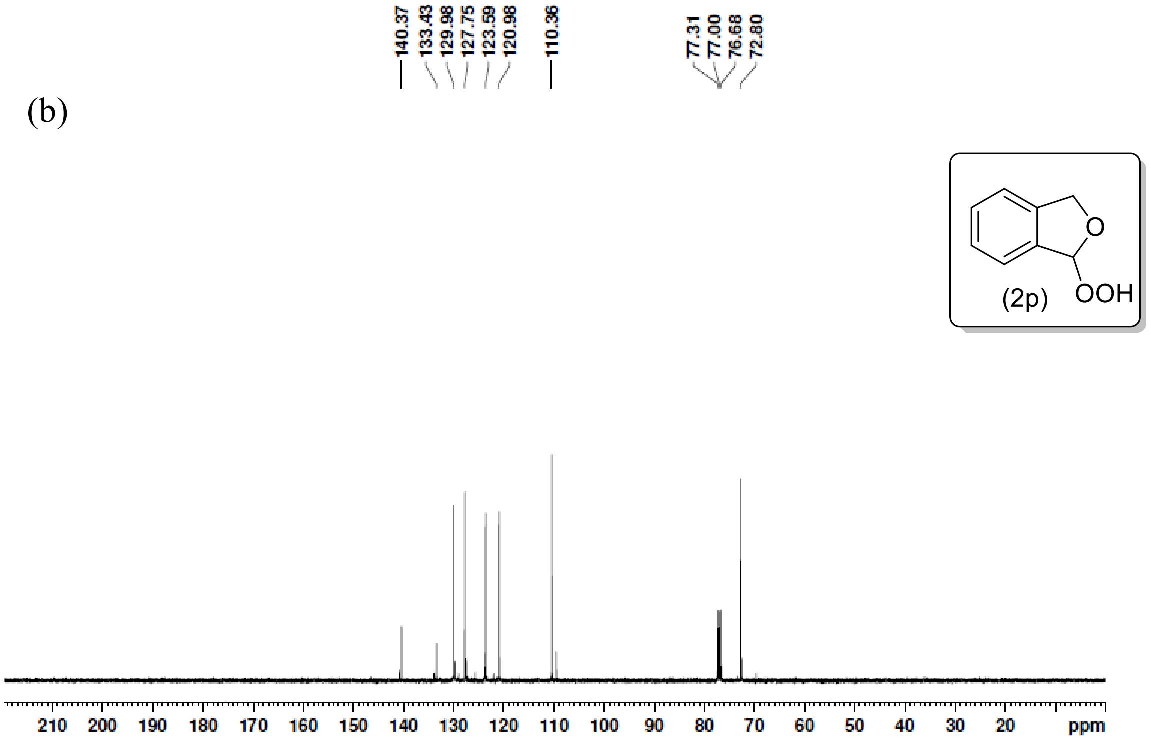

Supplementary Figure 15. NMR spectra of compound **2p**, (a) ^{1}H NMR, and (b) ^{13}C NMR spectra

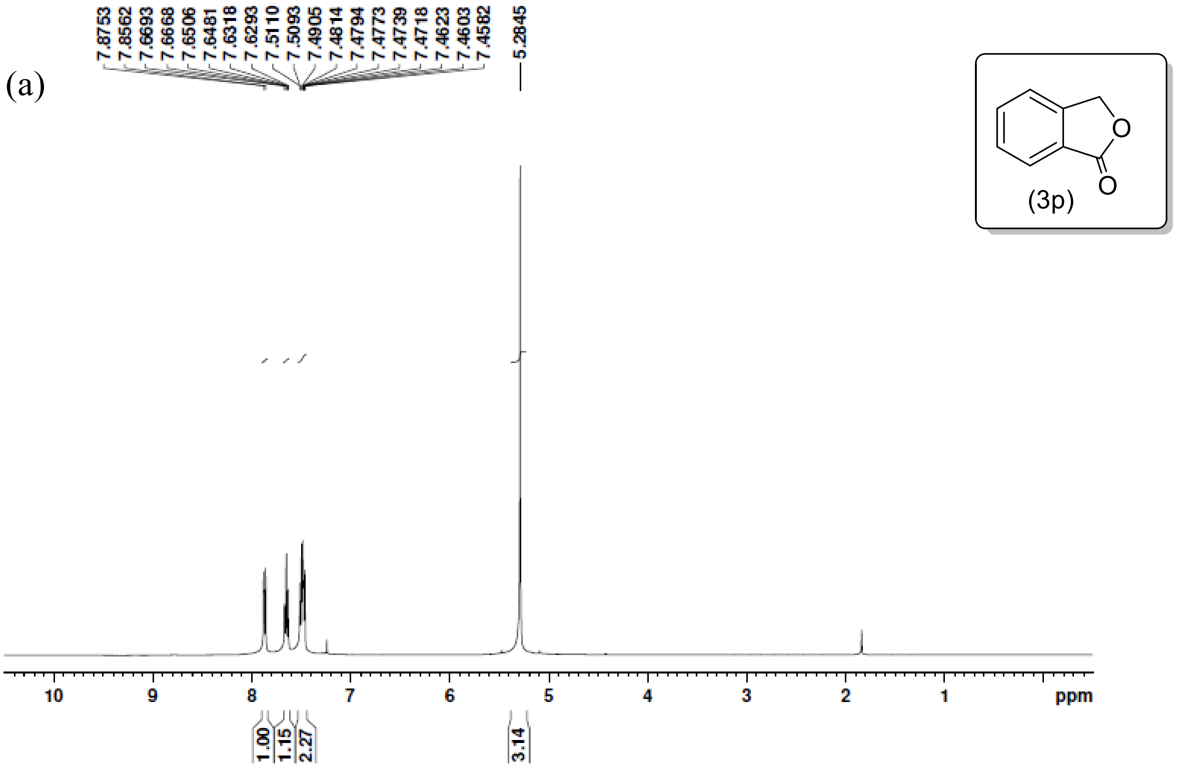

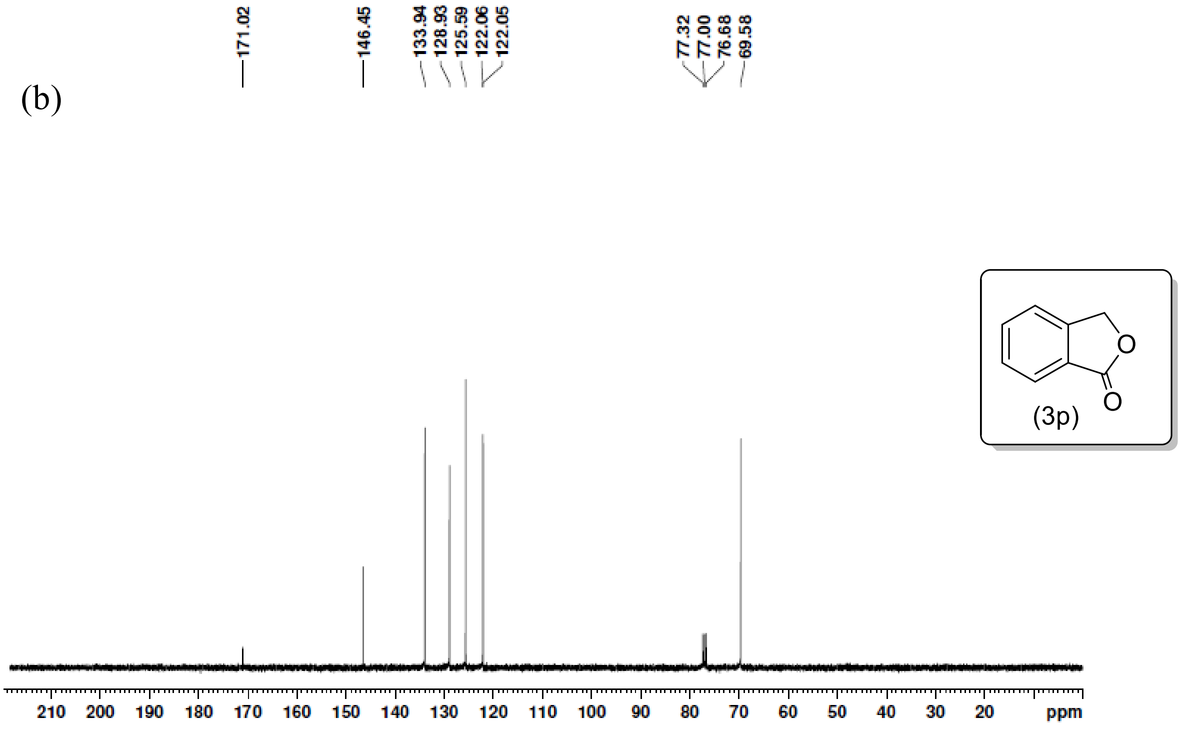

Supplementary Figure 16. NMR spectra of compound **3p**, (a) ^{1}H NMR, and (b) ^{13}C NMR spectra

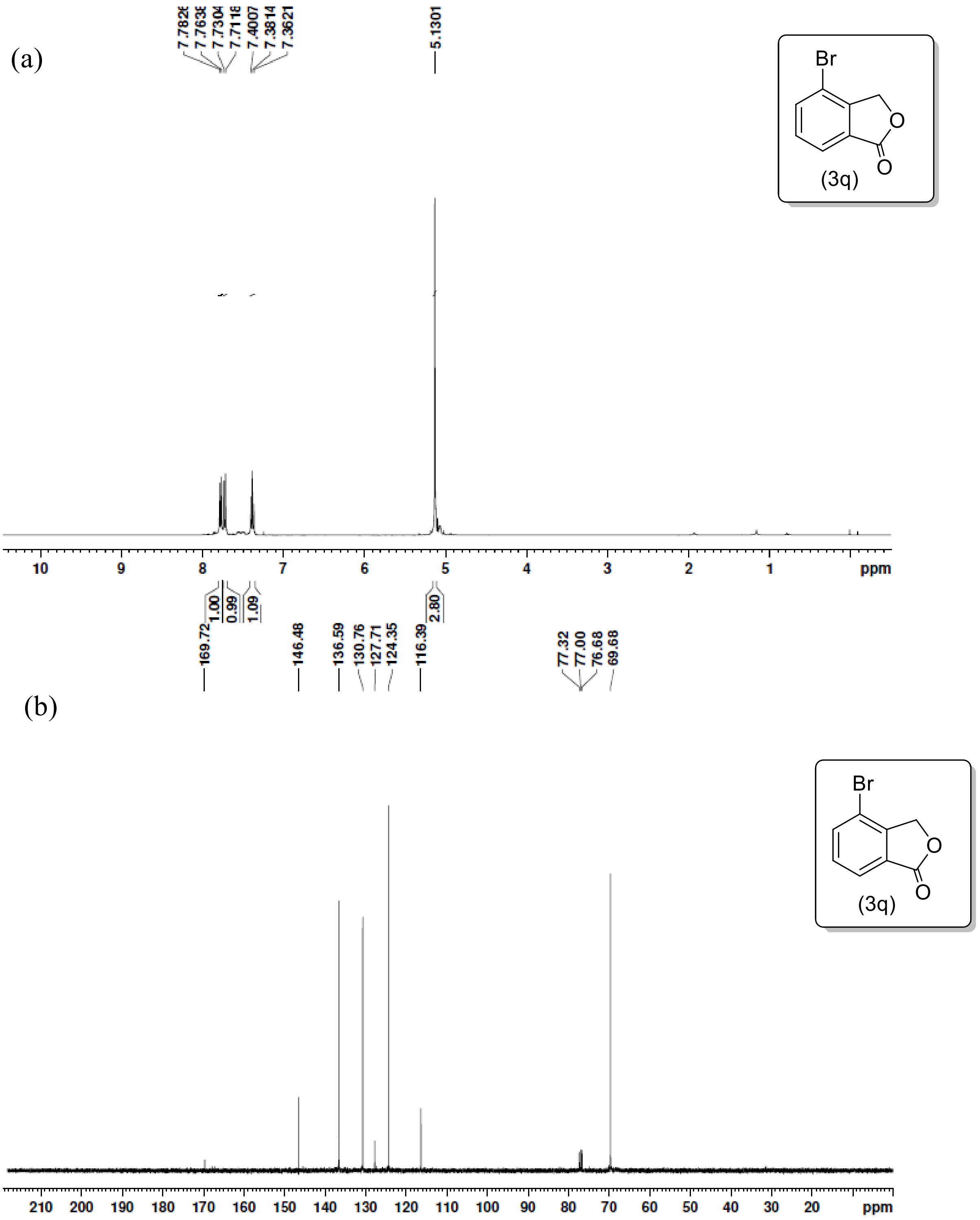

Supplementary Figure 17. NMR spectra of compound **3q**, (a) ^{1}H NMR, and (b) ^{13}C NMR spectra

(a)

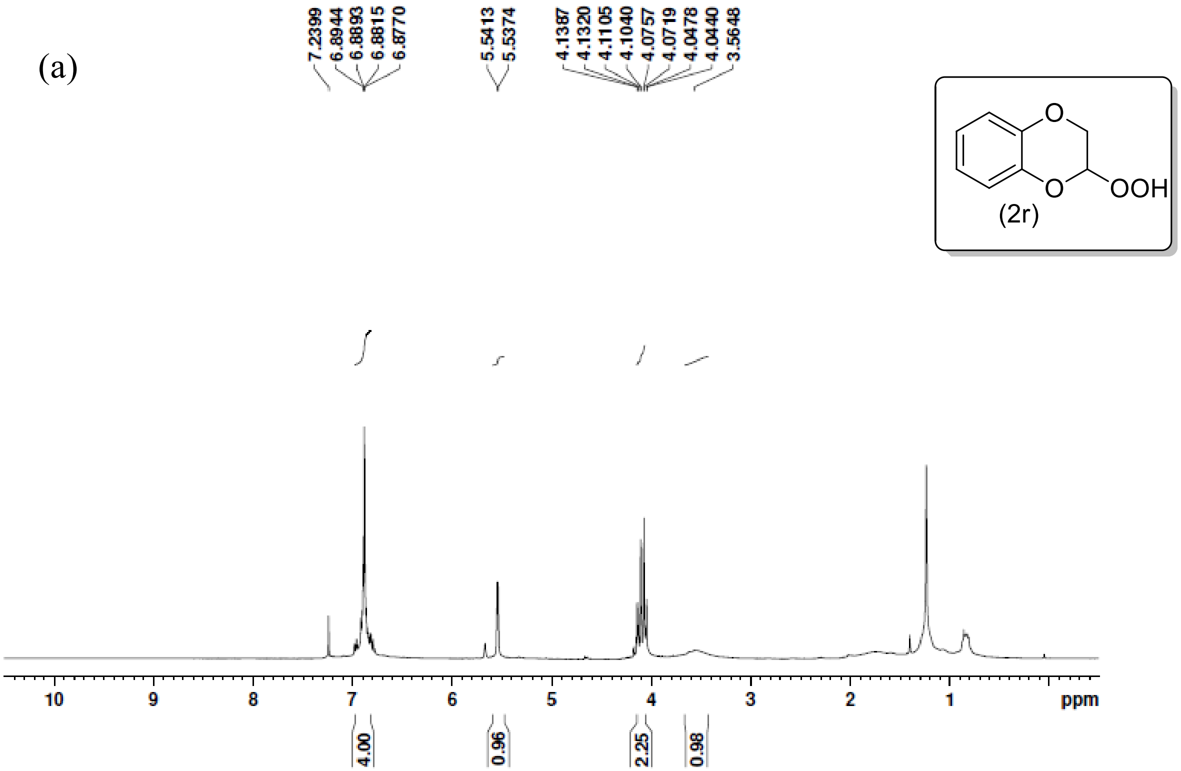

(b)

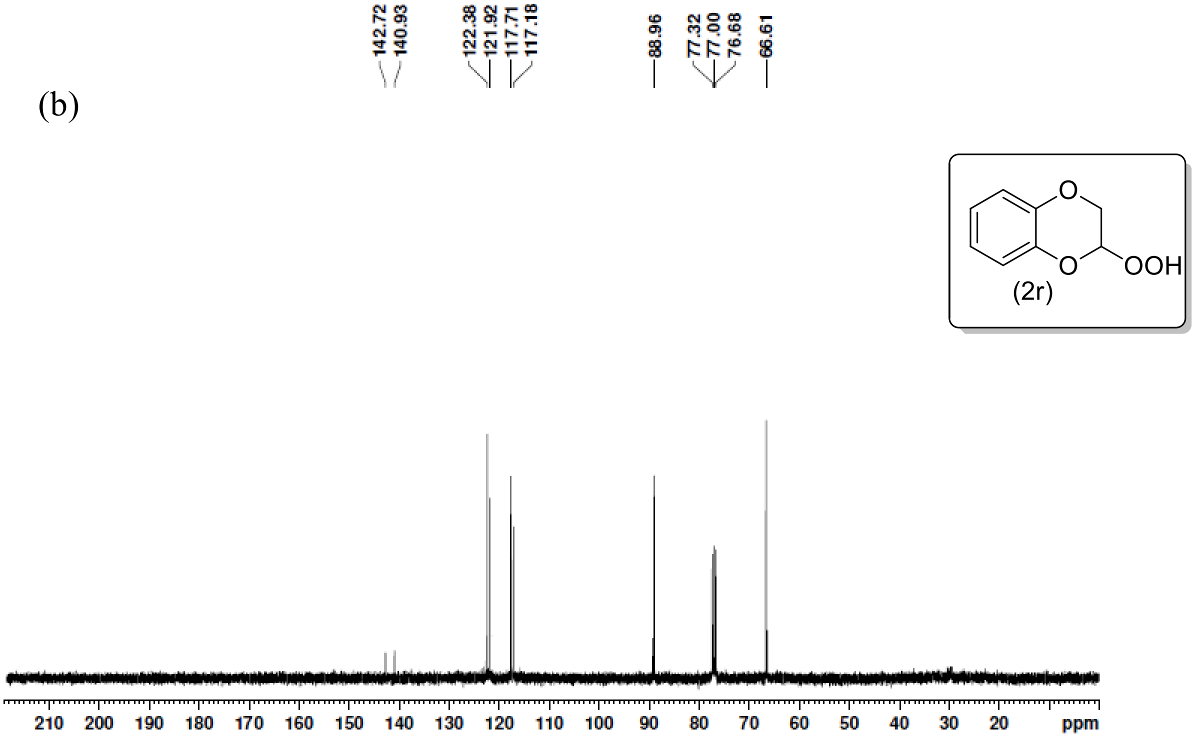

Supplementary Figure 18. NMR spectra of compound **2r**, (a) ^{1}H NMR, and (b) ^{13}C NMR spectra.

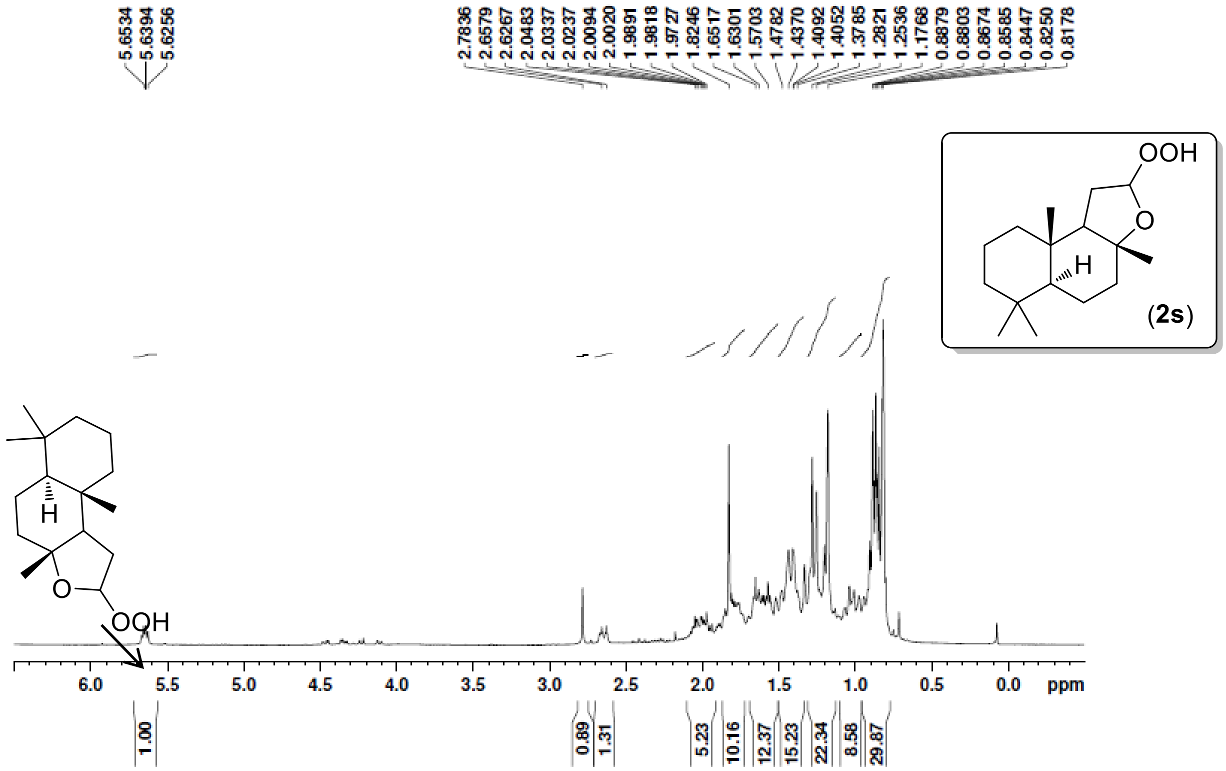

Supplementary Figure 19. ^{1}H NMR spectrum of compound **2s.**

(a)

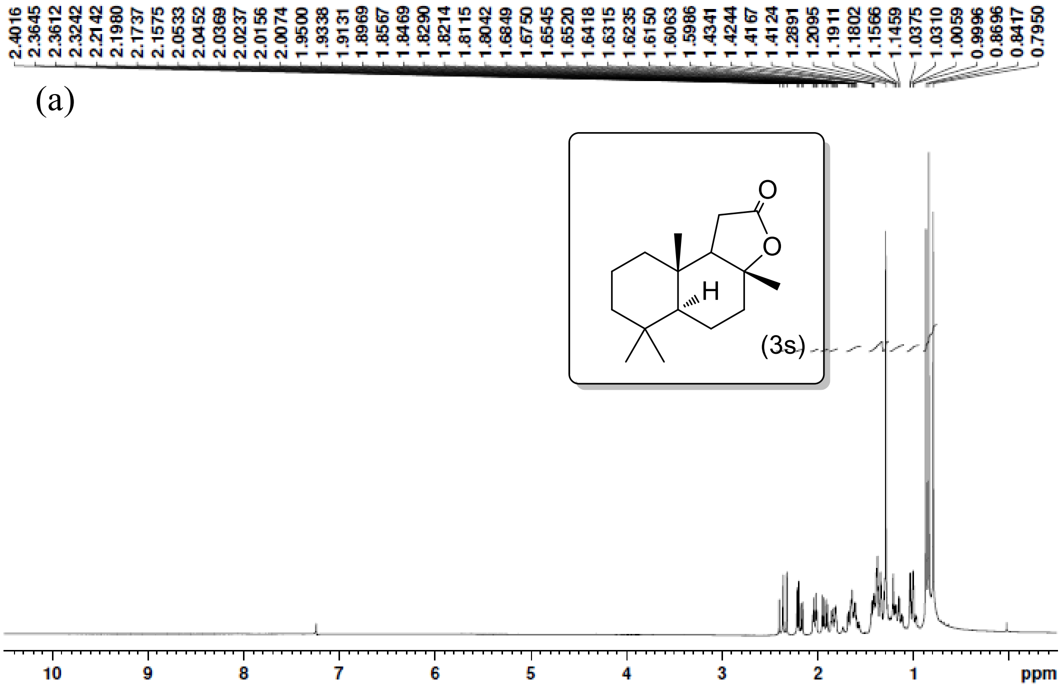

(b)

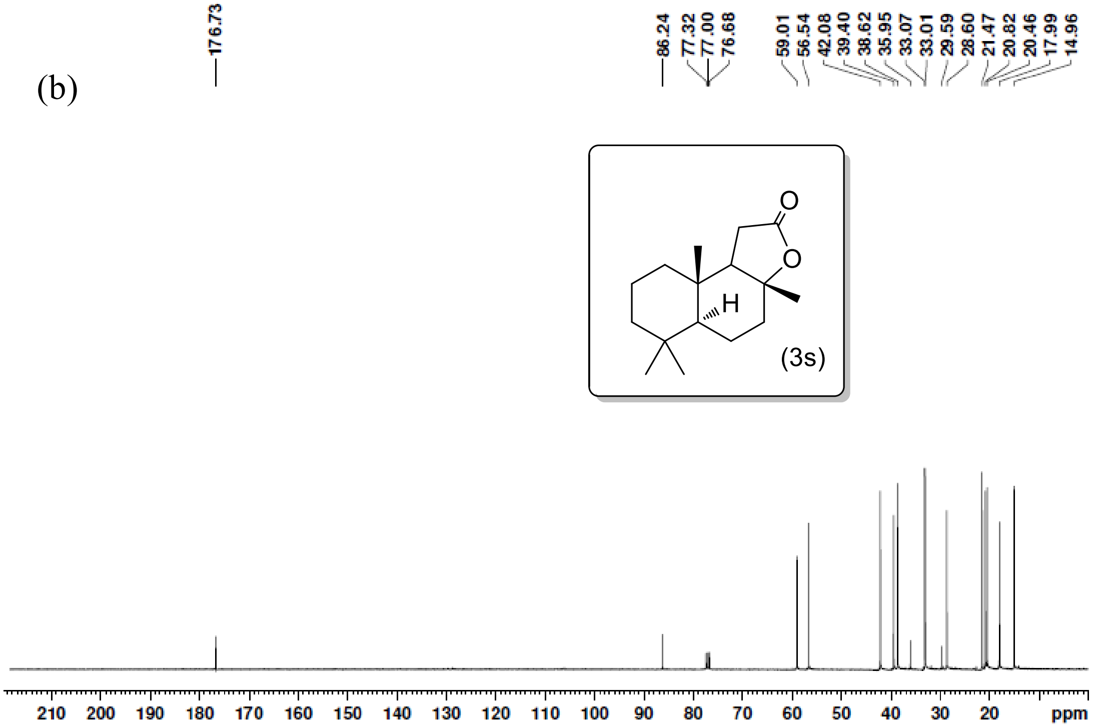

Supplementary Figure 20. NMR spectra of compound **3s**, (a) ^{1}H NMR, and (b) ^{13}C NMR spectra.

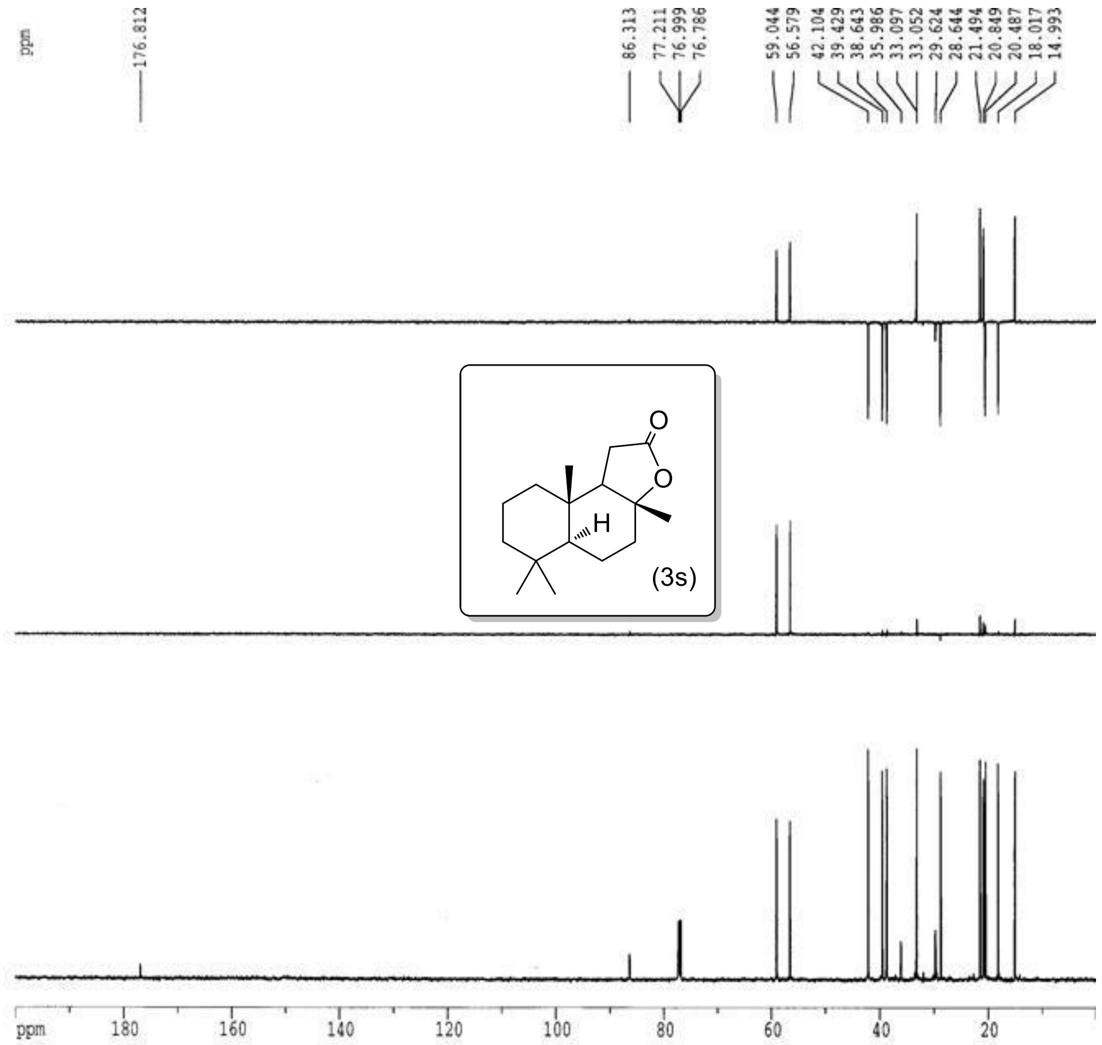

Supplementary Figure 21. ^{13}C NMR spectra of compound **3s**.

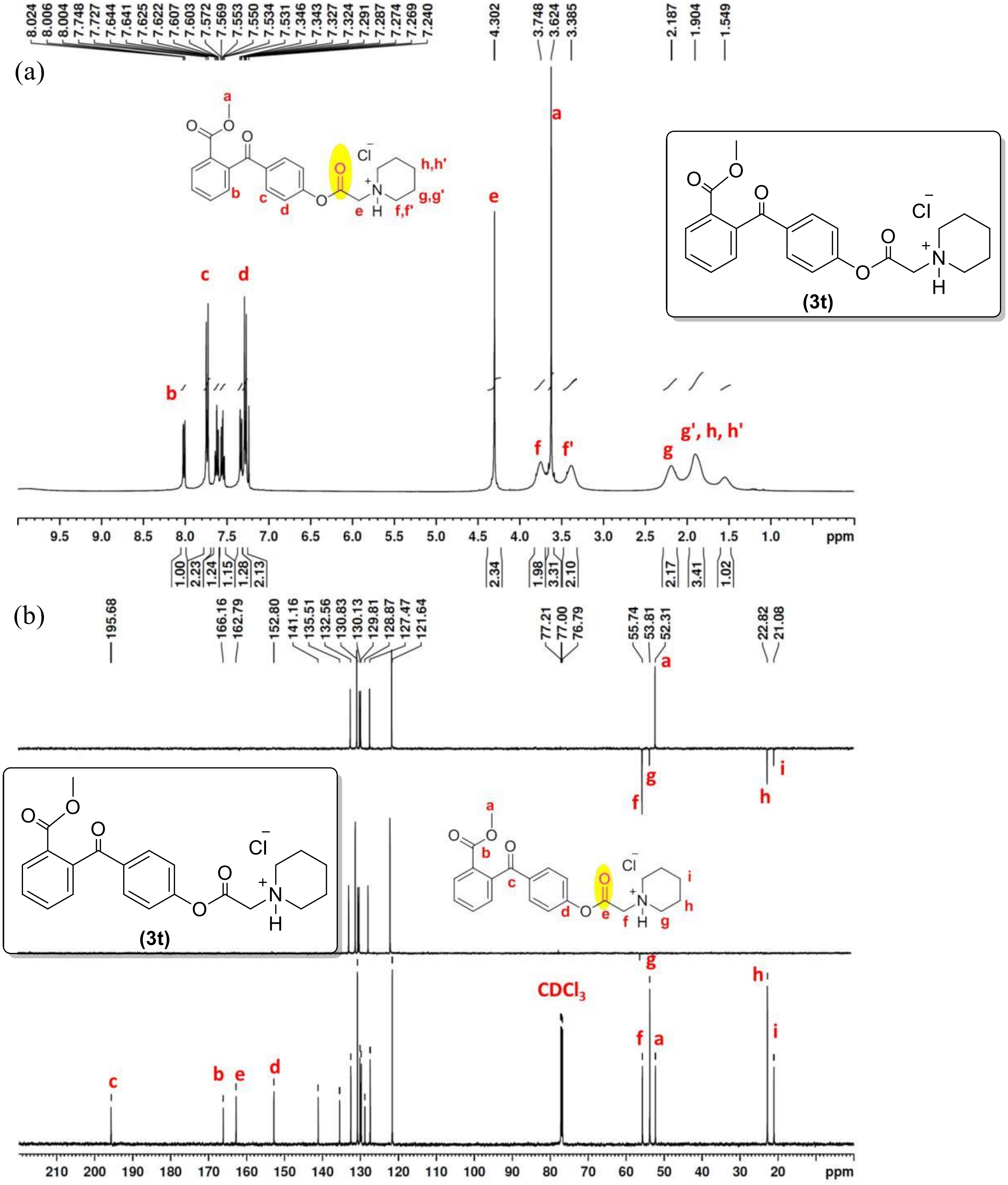

Supplementary Figure 22. NMR spectra of compound **3t**, (a) ^{1}H NMR, and (b) ^{13}C NMR spectra.

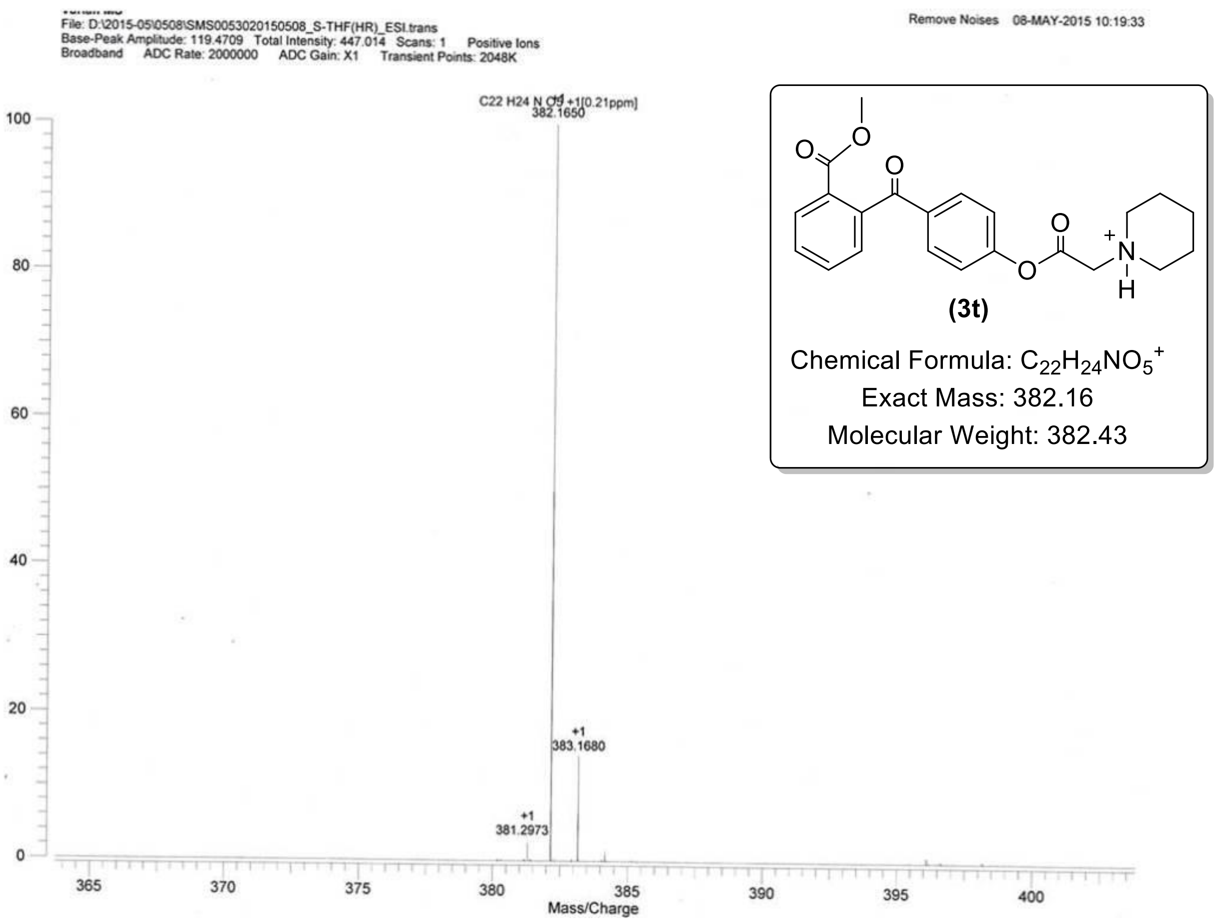

Supplementary Figure 23. HRMS spectrum of compound **3t.**

(a)

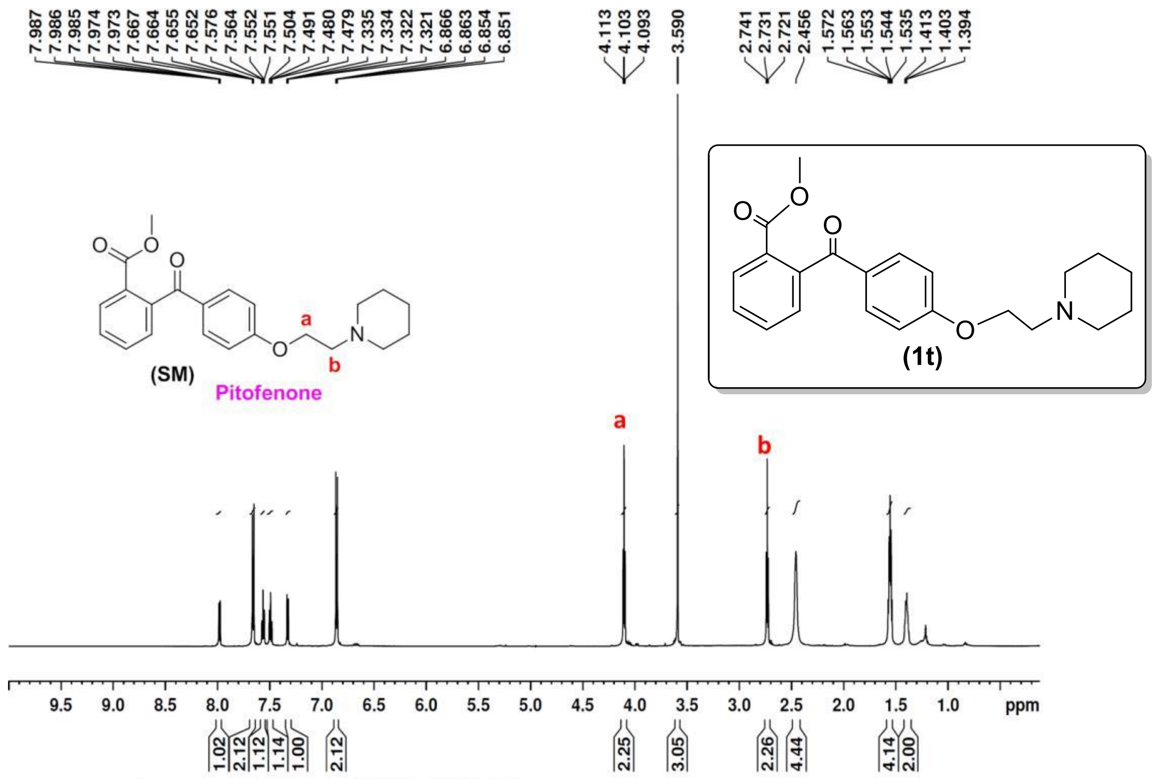

(b)

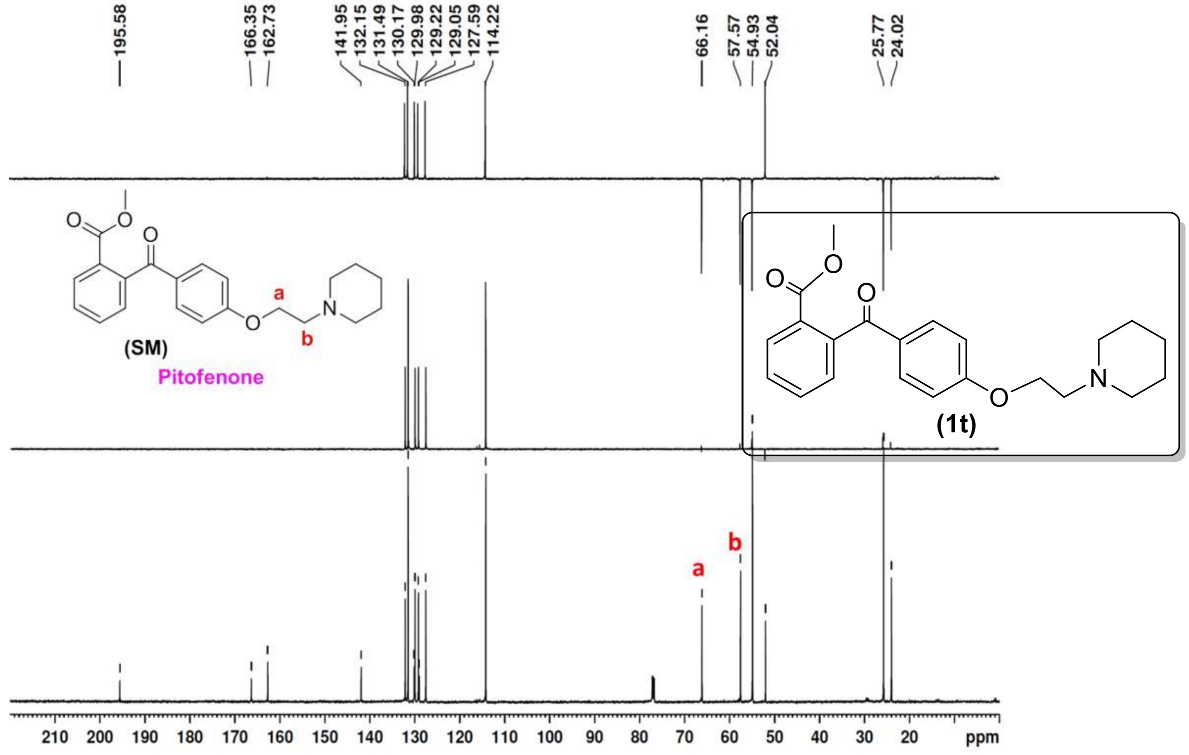

Supplementary Figure 24. NMR spectra of compound **1t**, (a) ^{1}H NMR, and (b) ^{13}C NMR spectra.

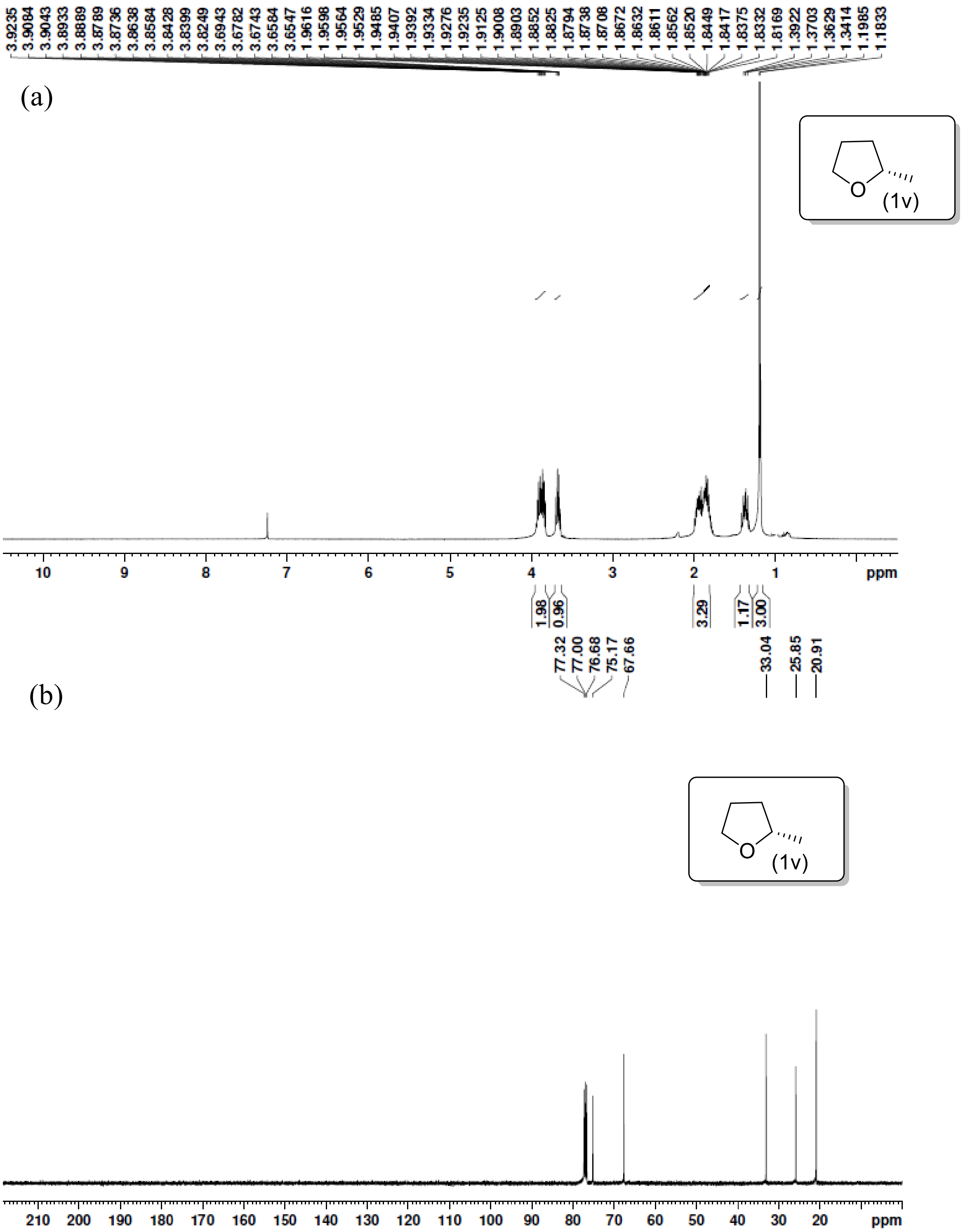

Supplementary Figure 25. NMR spectra of compound **1v**, (a) ^{1}H NMR, and (b) ^{13}C NMR spectra.

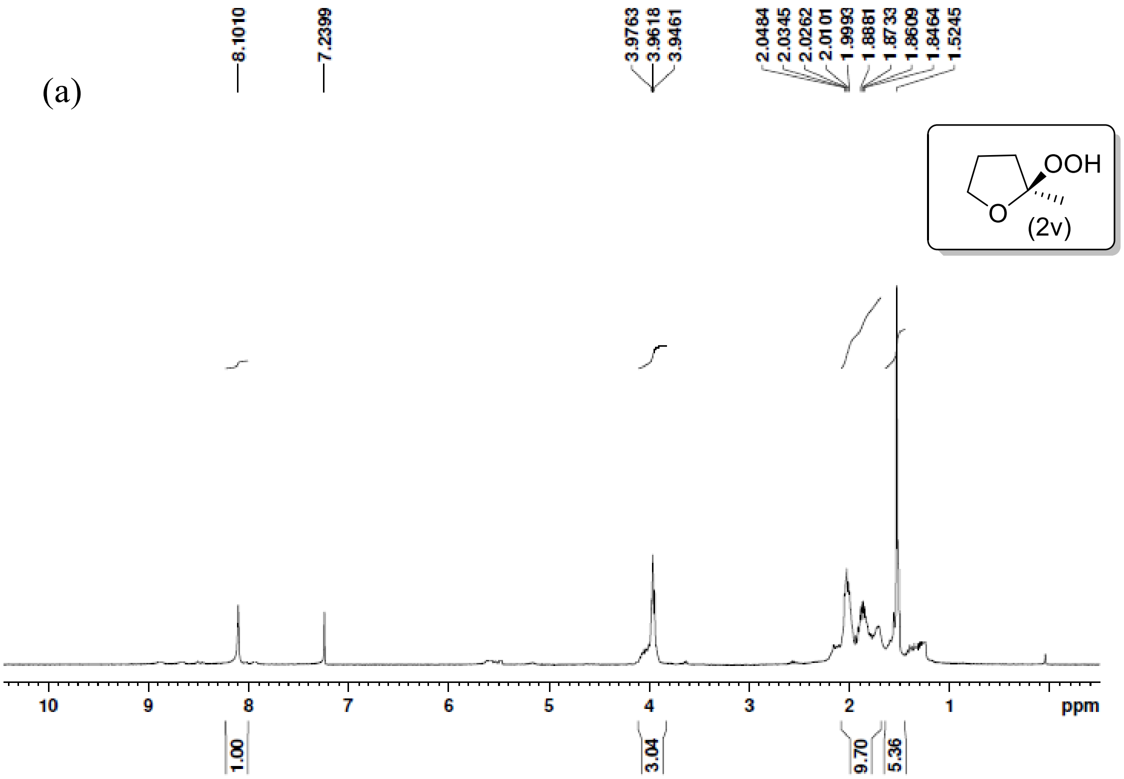

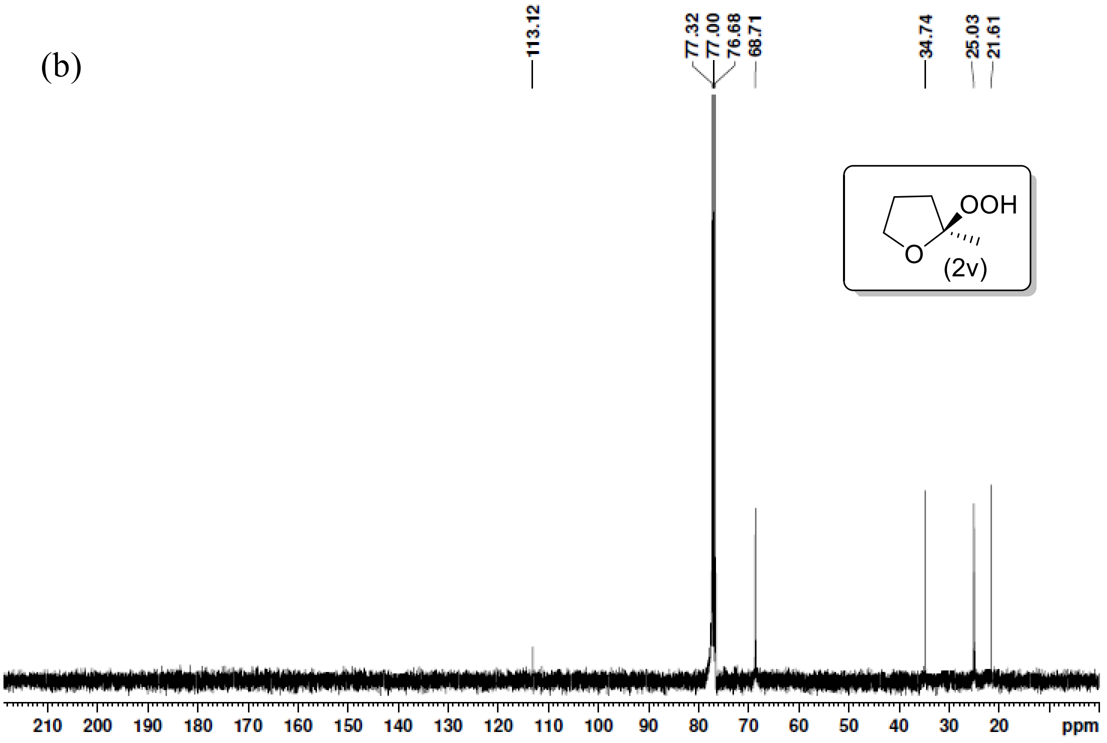

Supplementary Figure 26. NMR spectra of compound **2v**, (a) ^{1}H NMR, and (b) ^{13}C NMR spectra.

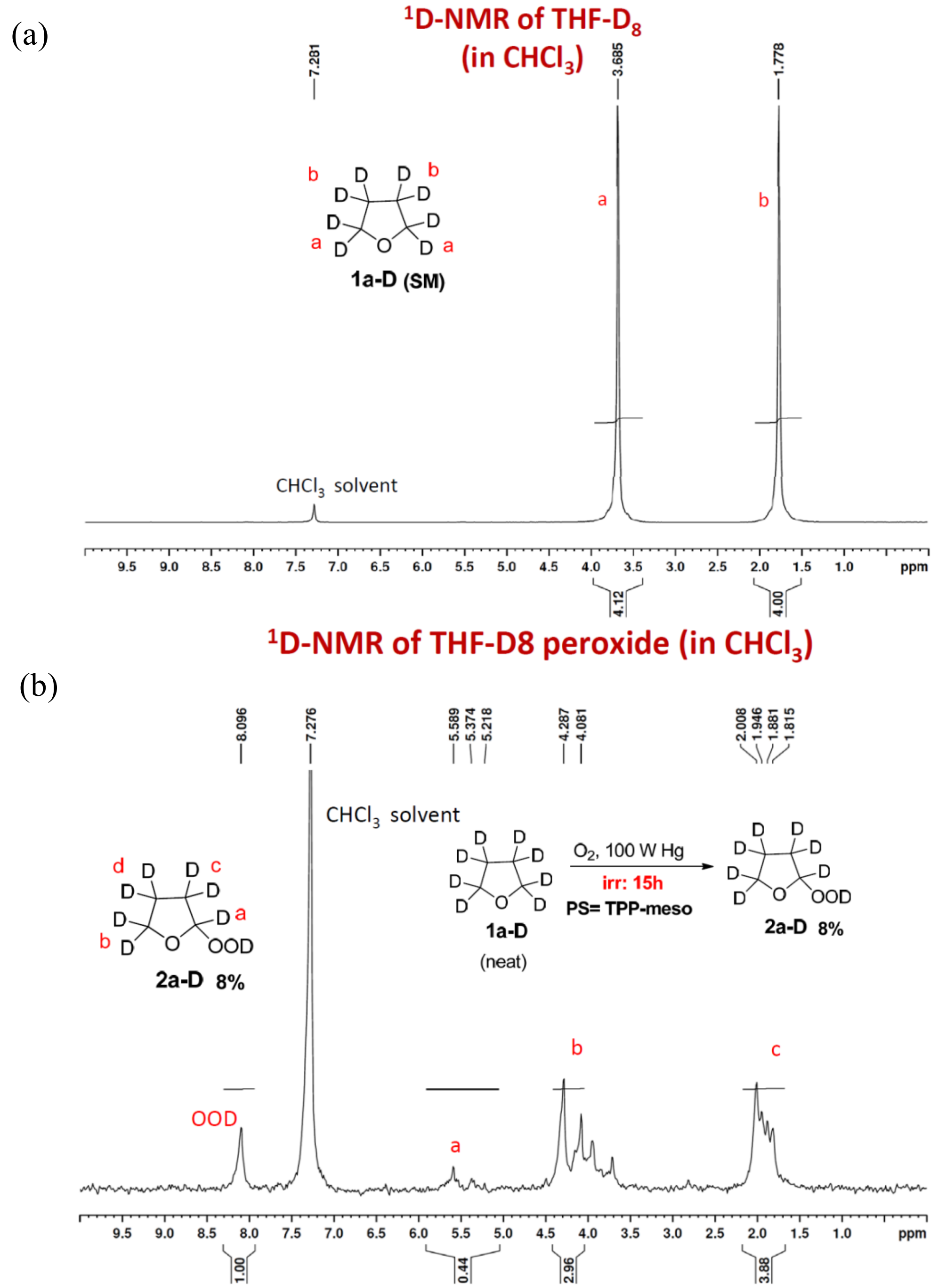

Supplementary Figure 27. ^{1}H NMR spectra of (a) THF-d_8, and (b) THF-d_8-hydroperoxide.

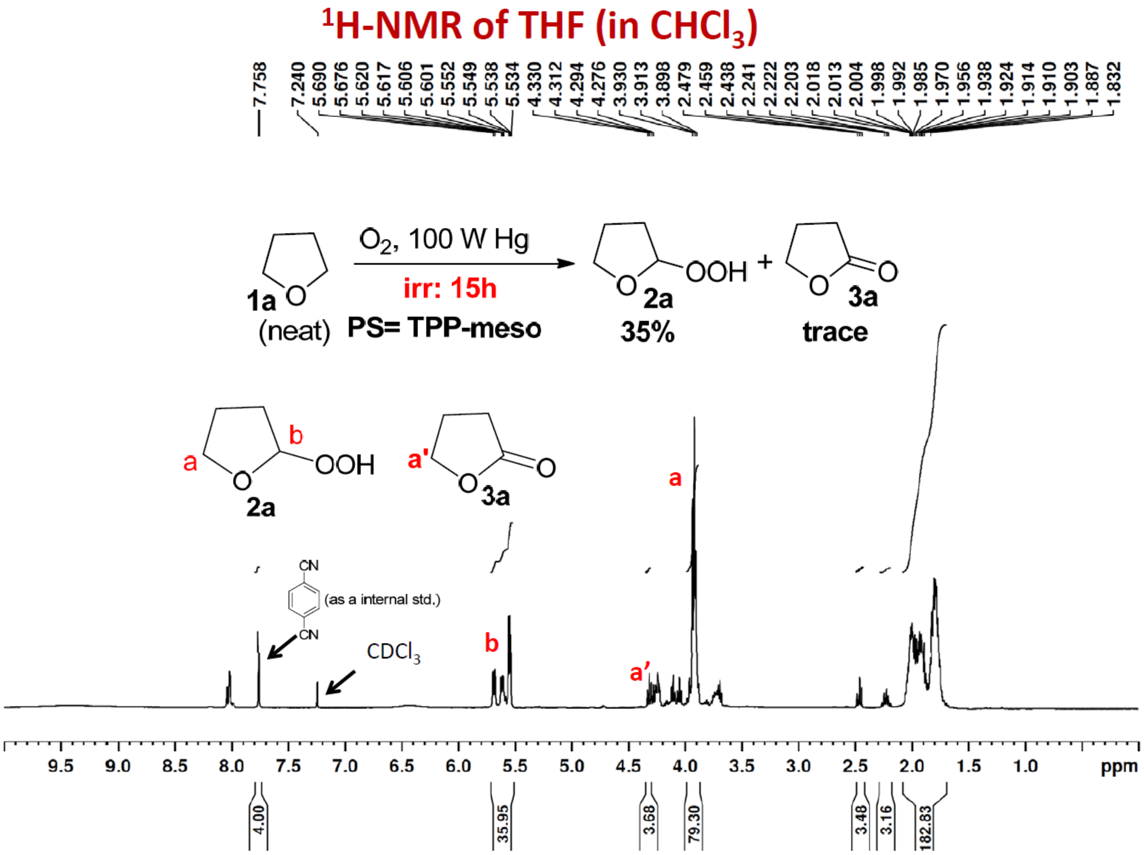

Supplementary Figure 28. ^{1}H NMR spectrum of compound THF-hydroperoxide and THF-lactone.

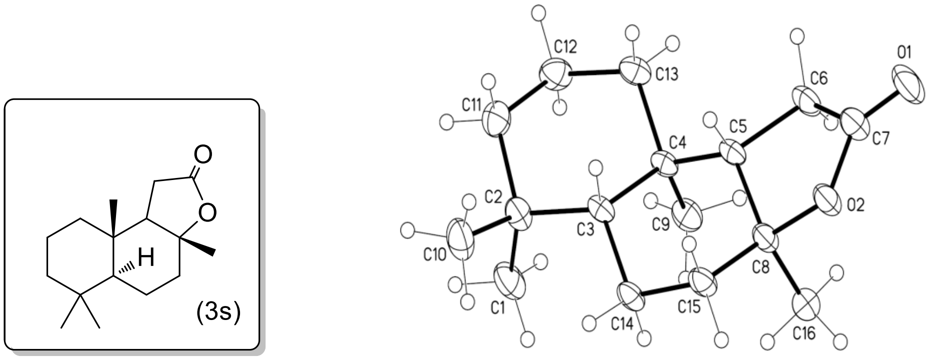

Supplementary Figure 29. ORTEP diagram of compound (3a*R*,5a*S*,9a*S*)-3a,6,6,9a tetramethyl decahydronaphtho[2,1-b]furan-2(3aH)-one (3s).

Supplementary Table 1. Crystal data and structure refinement for 120421LT_0m.

Identification code	120421lt_0m	
Empirical formula	C16 H26 O2	
Formula weight	250.37	
Temperature	100(2) K	
Wavelength	0.71073 Å	
Crystal system	Monoclinic	
Space group	P 1 21 1	
Unit cell dimensions	a = 7.4988(5) Å	α= 90°.
	b = 10.6861(7) Å	β= 110.507(3)°.
	c = 9.4162(7) Å	γ = 90°.
Volume	706.73(8) Å ^{3}	
Z	2	
Density (calculated)	1.177 Mg/m^3	
Absorption coefficient	0.075 mm^{-1}	
F(000)	276	
Crystal size	0.15 x 0.03 x 0.03 mm^3	
Theta range for data collection	2.31 to 26.40°.	
Index ranges	-9<=h<=9, -8<=k<=13, -11<=l<=11	
Reflections collected	6262	
Independent reflections	2329 [R(int) = 0.0307]	
Completeness to theta = 26.40°	99.8 %	
Absorption correction	Semi-empirical from equivalents	
Max. and min. transmission	0.9486 and 0.7138	
Refinement method	Full-matrix least-squares on F^2	
Data / restraints / parameters	2329 / 1 / 167	
Goodness-of-fit on F^2	1.056	
Final R indices [I>2sigma(I)]	R1 = 0.0340, wR2 = 0.0771	
R indices (all data)	R1 = 0.0399, wR2 = 0.0801	
Absolute structure parameter	-1.5(12)	
Largest diff. peak and hole	0.143 and -0.191 e.Å $^{-3}$	

Supplementary Table 2. Atomic coordinates (x 10^4) and equivalent isotropic displacement parameters (Å ^{2}x 10^3) for 120421LT_0m. U(eq) is defined as one third of the trace of the orthogonalized U^{ij} tensor.

	x	y	z	U(eq)
O(1)	-4674(2)	6625(1)	4264(2)	41(1)
O(2)	-1614(2)	6088(1)	4919(1)	26(1)
C(1)	3520(3)	2213(2)	10056(2)	34(1)
C(2)	2873(2)	3507(2)	10358(2)	25(1)
C(3)	1671(2)	4230(2)	8910(2)	19(1)
C(4)	-413(2)	3740(2)	8087(2)	20(1)
C(5)	-1320(2)	4777(2)	6945(2)	20(1)
C(6)	-3435(2)	4844(2)	5971(2)	27(1)
C(7)	-3410(2)	5924(2)	4950(2)	30(1)
C(8)	-385(2)	5050(2)	5765(2)	22(1)
C(9)	-577(2)	2456(2)	7331(2)	26(1)
C(10)	4671(3)	4269(2)	11222(2)	35(1)
C(11)	1690(3)	3433(2)	11408(2)	29(1)
C(12)	-321(2)	2935(2)	10654(2)	29(1)
C(13)	-1423(2)	3695(2)	9257(2)	26(1)
C(14)	2685(2)	4492(2)	7772(2)	22(1)
C(15)	1648(2)	5486(2)	6583(2)	24(1)
C(16)	-475(3)	4070(2)	4580(2)	29(1)

Supplementary Table 3. Bond lengths [Å] and angles [°] for 120421LT_0m.

O(1)-C(7)	1.203(2)
O(2)-C(7)	1.3680(19)
O(2)-C(8)	1.483(2)
C(1)-C(2)	1.524(3)
C(1)-H(1A)	0.9800
C(1)-H(1B)	0.9800
C(1)-H(1C)	0.9800
C(2)-C(10)	1.541(2)
C(2)-C(11)	1.545(2)
C(2)-C(3)	1.552(2)
C(3)-C(14)	1.541(2)
C(3)-C(4)	1.571(2)
C(3)-H(3)	1.0000
C(4)-C(5)	1.529(2)
C(4)-C(9)	1.531(3)
C(4)-C(13)	1.540(2)
C(5)-C(6)	1.531(2)
C(5)-C(8)	1.535(2)
C(5)-H(5)	1.0000
C(6)-C(7)	1.506(3)
C(6)-H(6A)	0.9900
C(6)-H(6B)	0.9900
C(8)-C(16)	1.515(3)
C(8)-C(15)	1.521(2)
C(9)-H(9A)	0.9800
C(9)-H(9B)	0.9800
C(9)-H(9C)	0.9800
C(10)-H(10A)	0.9800
C(10)-H(10B)	0.9800
C(10)-H(10C)	0.9800
C(11)-C(12)	1.520(3)
C(11)-H(11A)	0.9900
C(11)-H(11B)	0.9900
C(12)-C(13)	1.520(3)
C(12)-H(12A)	0.9900
C(12)-H(12B)	0.9900

C(13)-H(13A)	0.9900
C(13)-H(13B)	0.9900
C(14)-C(15)	1.542(2)
C(14)-H(14A)	0.9900
C(14)-H(14B)	0.9900
C(15)-H(15A)	0.9900
C(15)-H(15B)	0.9900
C(16)-H(16A)	0.9800
C(16)-H(16B)	0.9800
C(16)-H(16C)	0.9800
C(7)-O(2)-C(8)	108.90(13)
C(2)-C(1)-H(1A)	109.5
C(2)-C(1)-H(1B)	109.5
H(1A)-C(1)-H(1B)	109.5
C(2)-C(1)-H(1C)	109.5
H(1A)-C(1)-H(1C)	109.5
H(1B)-C(1)-H(1C)	109.5
C(1)-C(2)-C(10)	107.52(15)
C(1)-C(2)-C(11)	111.38(16)
C(10)-C(2)-C(11)	106.34(15)
C(1)-C(2)-C(3)	114.38(15)
C(10)-C(2)-C(3)	109.40(15)
C(11)-C(2)-C(3)	107.51(13)
C(14)-C(3)-C(2)	115.14(13)
C(14)-C(3)-C(4)	111.47(13)
C(2)-C(3)-C(4)	115.99(14)
C(14)-C(3)-H(3)	104.2
C(2)-C(3)-H(3)	104.2
C(4)-C(3)-H(3)	104.2
C(5)-C(4)-C(9)	112.29(14)
C(5)-C(4)-C(13)	108.47(13)
C(9)-C(4)-C(13)	108.99(14)
C(5)-C(4)-C(3)	103.03(13)
C(9)-C(4)-C(3)	115.59(13)
C(13)-C(4)-C(3)	108.14(13)
C(4)-C(5)-C(6)	124.59(14)
C(4)-C(5)-C(8)	116.36(13)

C(6)-C(5)-C(8)	101.41(13)
C(4)-C(5)-H(5)	104.1
C(6)-C(5)-H(5)	104.1
C(8)-C(5)-H(5)	104.1
C(7)-C(6)-C(5)	100.14(14)
C(7)-C(6)-H(6A)	111.7
C(5)-C(6)-H(6A)	111.7
C(7)-C(6)-H(6B)	111.7
C(5)-C(6)-H(6B)	111.7
H(6A)-C(6)-H(6B)	109.5
O(1)-C(7)-O(2)	120.35(18)
O(1)-C(7)-C(6)	129.46(16)
O(2)-C(7)-C(6)	110.14(14)
O(2)-C(8)-C(16)	105.22(13)
O(2)-C(8)-C(15)	111.58(14)
C(16)-C(8)-C(15)	111.25(14)
O(2)-C(8)-C(5)	100.12(12)
C(16)-C(8)-C(5)	119.20(15)
C(15)-C(8)-C(5)	108.83(14)
C(4)-C(9)-H(9A)	109.5
C(4)-C(9)-H(9B)	109.5
H(9A)-C(9)-H(9B)	109.5
C(4)-C(9)-H(9C)	109.5
H(9A)-C(9)-H(9C)	109.5
H(9B)-C(9)-H(9C)	109.5
C(2)-C(10)-H(10A)	109.5
C(2)-C(10)-H(10B)	109.5
H(10A)-C(10)-H(10B)	109.5
C(2)-C(10)-H(10C)	109.5
H(10A)-C(10)-H(10C)	109.5
H(10B)-C(10)-H(10C)	109.5
C(12)-C(11)-C(2)	114.66(14)
C(12)-C(11)-H(11A)	108.6
C(2)-C(11)-H(11A)	108.6
C(12)-C(11)-H(11B)	108.6
C(2)-C(11)-H(11B)	108.6
H(11A)-C(11)-H(11B)	107.6

C(13)-C(12)-C(11)	111.54(15)
C(13)-C(12)-H(12A)	109.3
C(11)-C(12)-H(12A)	109.3
C(13)-C(12)-H(12B)	109.3
C(11)-C(12)-H(12B)	109.3
H(12A)-C(12)-H(12B)	108.0
C(12)-C(13)-C(4)	112.38(14)
C(12)-C(13)-H(13A)	109.1
C(4)-C(13)-H(13A)	109.1
C(12)-C(13)-H(13B)	109.1
C(4)-C(13)-H(13B)	109.1
H(13A)-C(13)-H(13B)	107.9
C(3)-C(14)-C(15)	112.53(13)
C(3)-C(14)-H(14A)	109.1
C(15)-C(14)-H(14A)	109.1
C(3)-C(14)-H(14B)	109.1
C(15)-C(14)-H(14B)	109.1
H(14A)-C(14)-H(14B)	107.8
C(8)-C(15)-C(14)	108.57(14)
C(8)-C(15)-H(15A)	110.0
C(14)-C(15)-H(15A)	110.0
C(8)-C(15)-H(15B)	110.0
C(14)-C(15)-H(15B)	110.0
H(15A)-C(15)-H(15B)	108.4
C(8)-C(16)-H(16A)	109.5
C(8)-C(16)-H(16B)	109.5
H(16A)-C(16)-H(16B)	109.5
C(8)-C(16)-H(16C)	109.5
H(16A)-C(16)-H(16C)	109.5
H(16B)-C(16)-H(16C)	109.5

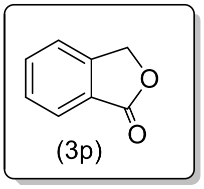

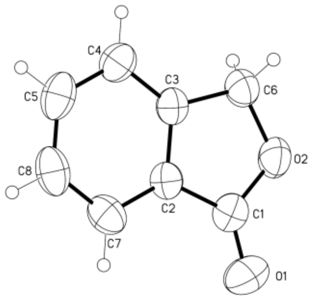

Supplementary Figure 30. ORTEP diagram of compound Isobenzofuran-1(3H)-one (**3p**)

Supplementary Table 4. Crystal data and structure refinement for 110813_0m.

Identification code	110813_0m	
Empirical formula	C8 H6 O2	
Formula weight	134.13	
Temperature	296(2) K	
Wavelength	0.71073 Å	
Crystal system	Monoclinic	
Space group	P 1 21/c 1	
Unit cell dimensions	a = 7.7471(7) Å	α= 90°.
	b = 10.8420(11) Å	β= 112.208(4)°.
	c = 8.1277(9) Å	γ = 90°.
Volume	632.04(11) Å ^{3}	
Z	4	
Density (calculated)	1.410 Mg/m^{3}	
Absorption coefficient	0.102 mm^{-1}	
F(000)	280	
Crystal size	0.20 x 0.17 x 0.15 mm^{3}	
Theta range for data collection	2.84 to 26.43°.	
Index ranges	-6<=h<=9, -13<=k<=12, -10<=l<=10	
Reflections collected	5345	
Independent reflections	1293 [R(int) = 0.0338]	
Completeness to theta = 26.43°	99.4 %	
Absorption correction	Semi-empirical from equivalents	
Max. and min. transmission	0.9486 and 0.8358	
Refinement method	Full-matrix least-squares on F^{2}	
Data / restraints / parameters	1293 / 0 / 91	
Goodness-of-fit on F^{2}	1.180	

Final R indices [I>2sigma(I)]	R1 = 0.0405, wR2 = 0.1125
R indices (all data)	R1 = 0.0662, wR2 = 0.1431
Largest diff. peak and hole	0.173 and -0.236 e.Å $^{-3}$

Supplementary Table 5. Atomic coordinates (x 10^4) and equivalent isotropic displacement parameters (Å ^{2}x 10^3) for 110813_0m. U(eq) is defined as one third of the trace of the orthogonalized U^{ij} tensor.

	x	y	z	U(eq)
O(1)	13257(2)	7538(1)	6052(2)	77(1)
O(2)	13760(2)	5928(1)	7876(2)	52(1)
C(1)	12631(2)	6742(2)	6691(2)	50(1)
C(2)	10699(2)	6454(2)	6429(2)	41(1)
C(3)	10699(2)	5478(2)	7500(2)	40(1)
C(4)	9038(2)	5029(2)	7531(2)	49(1)
C(5)	7412(2)	5593(2)	6465(2)	56(1)
C(6)	12676(2)	5102(2)	8496(2)	48(1)
C(7)	9051(2)	7013(2)	5329(2)	53(1)
C(8)	7407(2)	6572(2)	5368(2)	58(1)

Supplementary Table 6. Bond lengths [Å] and angles [°] for 110813_0m.

O(1)-C(1)	1.201(2)
O(2)-C(1)	1.354(2)
O(2)-C(6)	1.443(2)
C(1)-C(2)	1.463(2)
C(2)-C(3)	1.371(2)
C(2)-C(7)	1.391(2)
C(3)-C(4)	1.385(2)
C(3)-C(6)	1.494(2)
C(4)-C(5)	1.374(3)
C(4)-H(4)	0.9300
C(5)-C(8)	1.385(3)

C(5)-H(5)	0.9300
C(6)-H(6A)	0.9700
C(6)-H(6B)	0.9700
C(7)-C(8)	1.372(3)
C(7)-H(7)	0.9300
C(8)-H(8)	0.9300
C(1)-O(2)-C(6)	110.35(13)
O(1)-C(1)-O(2)	121.20(16)
O(1)-C(1)-C(2)	130.43(17)
O(2)-C(1)-C(2)	108.37(15)
C(3)-C(2)-C(7)	121.60(16)
C(3)-C(2)-C(1)	108.55(13)
C(7)-C(2)-C(1)	129.85(17)
C(2)-C(3)-C(4)	120.43(15)
C(2)-C(3)-C(6)	107.97(14)
C(4)-C(3)-C(6)	131.59(17)
C(5)-C(4)-C(3)	117.96(18)
C(5)-C(4)-H(4)	121.0
C(3)-C(4)-H(4)	121.0
C(4)-C(5)-C(8)	121.73(17)
C(4)-C(5)-H(5)	119.1
C(8)-C(5)-H(5)	119.1
O(2)-C(6)-C(3)	104.74(13)
O(2)-C(6)-H(6A)	110.8
C(3)-C(6)-H(6A)	110.8
O(2)-C(6)-H(6B)	110.8
C(3)-C(6)-H(6B)	110.8
H(6A)-C(6)-H(6B)	108.9
C(8)-C(7)-C(2)	117.89(18)
C(8)-C(7)-H(7)	121.1
C(2)-C(7)-H(7)	121.1
C(7)-C(8)-C(5)	120.37(16)
C(7)-C(8)-H(8)	119.8
C(5)-C(8)-H(8)	119.8

Symmetry transformations used to generate equivalent atoms:

Supplementary Table 7. Anisotropic displacement parameters ($Å^2 x 10^3$) for 110813_0m. The anisotropic displacement factor exponent takes the form: $-2\pi^2[h^2 a^{*2}U^{11} + ... + 2 h k a^* b^* U^{12}]$

	U^{11}	U^{22}	U^{33}	U^{23}	U^{13}	U^{12}
O(1)	66(1)	76(1)	96(1)	20(1)	41(1)	-8(1)
O(2)	36(1)	64(1)	58(1)	4(1)	18(1)	5(1)
C(1)	46(1)	54(1)	50(1)	-1(1)	20(1)	-1(1)
C(2)	37(1)	44(1)	39(1)	-5(1)	11(1)	1(1)
C(3)	38(1)	43(1)	37(1)	-6(1)	13(1)	1(1)
C(4)	45(1)	55(1)	48(1)	-6(1)	18(1)	-6(1)
C(5)	38(1)	71(1)	59(1)	-20(1)	19(1)	-9(1)
C(6)	42(1)	53(1)	49(1)	4(1)	16(1)	6(1)
C(7)	48(1)	52(1)	49(1)	4(1)	8(1)	6(1)
C(8)	37(1)	69(1)	56(1)	-10(1)	3(1)	10(1)

Supplementary Table 8. Hydrogen coordinates (x 10^4) and isotropic displacement parameters ($Å^2 x 10^3$) for 110813_0m.

	x	y	z	U(eq)
H(4)	9024	4366	8252	59
H(5)	6284	5310	6480	67
H(6A)	13008	5191	9767	58
H(6B)	12873	4251	8240	58
H(7)	9062	7666	4591	64
H(8)	6283	6932	4654	70

(*S*)-2-methyltetrahydrofuran (1v) (1.0 M in $CHCl_3$)

No.	Sample Name	Measurement Date	PMT Voltage[V]	Temperature[C]	Optical Rotation Monitor	Specific O.R.	Path Length[mm]
1	Blank				-0.0021		
2	Blank-1	2015/10/29 下午 02:48	281	30.82	-0.0025		
3	Blank-2	2015/10/29 下午 02:49	281	30.84	-0.0020		
4	Blank-3	2015/10/29 下午 02:49	282	30.87	-0.0017		
5	Blank				-0.0013		
6	Blank-1	2015/10/29 下午 02:50	281	30.91	-0.0014		
7	Blank-2	2015/10/29 下午 02:50	281	30.93	-0.0011		
8	Blank-3	2015/10/29 下午 02:51	281	30.95	-0.0014		
9	Blank				-0.0011		
10	Blank-1	2015/10/29 下午 02:52	281	30.98	-0.0013		
11	Blank-2	2015/10/29 下午 02:52	282	30.98	-0.0010		
12	Blank-3	2015/10/29 下午 02:53	282	30.99	-0.0010		
13	sm				0.0976	19.7333	50
14	sm-1	2015/10/29 下午 02:58	284	31.24	0.0979	19.8000	
15	sm-2	2015/10/29 下午 02:59	285	31.27	0.0974	19.7000	

No.	Concentration[w/v%]	S.D.	C.V.	Comment
1		0.0004	19.5554	
2				
3				
4				
5		0.0002	13.3235	
6				
7				
8				
9		0.0002	15.7459	
10				
11				
12				
13	1.0000	0.0577	0.2926	
14				
15				

(*S*)-2-methyltetrahydrofuran (1v) (1.0 M in $CHCl_3$)

No.	Sample Name	Measurement Date	PMT Voltage[V]	Temperature[C]	Optical Rotation Monitor	Specific O.R.	Path Length[mm]
16	sm-3	2015/10/29 下午 02:59	284	31.31	0.0974	19.7000	
17	sm				0.0968	19.5867	50
18	sm-1	2015/10/29 下午 03:00	284	31.36	0.0975	19.7200	
19	sm-2	2015/10/29 下午 03:00	284	31.39	0.0975	19.7200	
20	sm-3	2015/10/29 下午 03:01	284	31.42	0.0955	19.3200	
21	sm				0.0959	19.4000	50
22	sm-1	2015/10/29 下午 03:02	284	31.48	0.0959	19.4000	
23	sm-2	2015/10/29 下午 03:02	284	31.51	0.0960	19.4200	
24	sm-3	2015/10/29 下午 03:03	284	31.53	0.0958	19.3800	
25	sm				0.0962	19.4667	50
26	sm-1	2015/10/29 下午 03:05	284	31.64	0.0960	19.4200	
27	sm-2	2015/10/29 下午 03:06	284	31.66	0.0962	19.4600	
28	sm-3	2015/10/29 下午 03:06	284	31.68	0.0965	19.5200	

No.	Concentration[w/v%]	S.D.	C.V.	Comment
16				
17	1.0000	0.2309	1.1791	
18				
19				
20				
21	1.0000	0.0200	0.1031	
22				
23				
24				
25	1.0000	0.0503	0.2586	
26				
27				
28				

(*R*)-2-hydroperoxy-2-methyltetrahydrofuran (2v) (1.0 M in $CHCl_3$)

No.	Sample Name	Measurement Date	PMT Voltage[V]	Temperature[C]	Optical Rotation Monitor	Specific O.R.	Path Length[mm]
1	Blank				-0.0106		
2	Blank-1	2015/10/29 下午 05:29	293	34.27	-0.0110		
3	Blank-2	2015/10/29 下午 05:29	293	34.32	-0.0103		
4	Blank-3	2015/10/29 下午 05:30	292	34.35	-0.0105		
5	Blank				-0.0112		
6	Blank-1	2015/10/29 下午 05:30	292	34.38	-0.0109		
7	Blank-2	2015/10/29 下午 05:31	292	34.39	-0.0114		
8	Blank-3	2015/10/29 下午 05:31	292	34.41	-0.0114		
9	peroxideproduct 1.0M				-0.0734	-12.4267	50
10	peroxideproduct 1.0M-1	2015/10/29 下午 05:37	284	34.41	-0.0730	-12.3533	
11	peroxideproduct 1.0M-2	2015/10/29 下午 05:38	284	34.41	-0.0735	-12.4533	
12	peroxideproduct 1.0M-3	2015/10/29 下午 05:38	284	34.42	-0.0736	-12.4733	
13	PEROXIDEPRODUCT 1.0M				-0.0744	-12.6333	50
14	PEROXIDEPRODUCT 1.0M-1	2015/10/29 下午 05:39	284	34.41	-0.0743	-12.6133	
15	PEROXIDEPRODUCT 1.0M-2	2015/10/29 下午 05:40	284	34.42	-0.0743	-12.6133	

No.	Concentration[w/v%]	S.D.	C.V.	Comment
1		0.0004	3.4015	
2				
3				
4				
5		0.0003	2.5698	
6				
7				
8				
9	1.0000	0.0643	0.5174	
10				
11				
12				
13	1.0000	0.0346	0.2742	
14				
15				

No.	Sample Name	Measurement Date	PMT Voltage[V]	Temperature[C]	Optical Rotation Monitor	Specific O.R.	Path Length[mm]
16	PEROXIDEPRODUCT 1.0M-3	2015/10/29 下午 05:40	284	34.42	-0.0746	-12.6733	
17	PEROXIDEPRODUCT 1.0M				-0.0744	-12.6267	50
18	PEROXIDEPRODUCT 1.0M-1	2015/10/29 下午 05:42	285	34.43	-0.0743	-12.6133	
19	PEROXIDEPRODUCT 1.0M-2	2015/10/29 下午 05:42	284	34.44	-0.0743	-12.6133	
20	PEROXIDEPRODUCT 1.0M-3	2015/10/29 下午 05:43	284	34.45	-0.0745	-12.6533	

No.	Concentration[w/v%]	S.D.	C.V.	Comment
16				
17	1.0000	0.0231	0.1829	
18				
19				
20				

(*R*)-2-hydroperoxy-2-methyltetrahydrofuran (2v) (0.75 M in $CHCl_3$)

No.	Sample Name	Measurement Date	PMT Voltage[V]	Temperature[C]	Optical Rotation Monitor	Specific O.R.	Path Length[mm]
1	Blank				-0.0050		
2	Blank-1	2015/10/29 下午 03:54	283	33.48	-0.0048		

PMT Voltage[V]	Temperature[C]	Optical Rotation Monitor	Specific O.R.	Path Length[mm]
284	33.51	-0.0049		
284	33.53	-0.0052		
		-0.0057		
283	33.57	-0.0055		
283	33.59	-0.0057		
283	33.60	-0.0059		
		-0.0567	-13.6089	50
285	33.84	-0.0568	-13.6267	
285	33.87	-0.0566	-13.5733	
285	33.89	-0.0568	-13.6267	
		-0.0570	-13.6711	50
285	33.95	-0.0569	-13.6533	
284	33.96	-0.0567	-13.6000	

No.	Concentration[w/v%]	S.D.	C.V.	Comment
1		0.0004	2.2395	
2				
3				
4				
5		0.0004	2.4896	
6				
7				
8				
9	2.0000	0.0265	0.2590	
10				
11				
12				
13	2.0000	0.0115	0.1123	
14				
15				

No.	Concentration[w/v%]	S.D.	C.V.	Comment
1		0.0002	4.1913	
2				
3				
4				
5		0.0002	3.5088	
6				
7				
8				
9	0.7500	0.0308	0.2263	
10				
11				
12				
13	0.7500	0.0815	0.5959	
14				
15				

PMT Voltage[V]	Temperature[C]	Optical Rotation Monitor	Specific O.R.	Path Length[mm]
285	33.97	-0.0573	-13.7600	
		-0.0570	-13.6889	50
285	34.03	-0.0572	-13.7333	
285	34.04	-0.0570	-13.6800	
284	34.07	-0.0569	-13.6533	

13	2.0000	0.0115	0.1123	
14				
15				

No.	Concentration[w/v%]	S.D.	C.V.	Comment
16				
17	0.7500	0.0407	0.2976	
18				
19				
20				

(*R*)-2-hydroperoxy-2-methyltetrahydrofuran (2v) (0.25 M in $CHCl_3$)

No.	Sample Name	Measurement Date	PMT Voltage[V]	Temperature[C]	Optical Rotation Monitor	Specific O.R.	Path Length[mm]
1	Blank				-0.0036		
2	Blank-1	2015/10/29 下午 04:39	284	34.70	-0.0034		
3	Blank-2	2015/10/29 下午 04:39	284	34.72	-0.0036		
4	Blank-3	2015/10/29 下午 04:40	284	34.72	-0.0038		
5	Blank				-0.0050		
6	Blank-1	2015/10/29 下午 04:41	284	34.74	-0.0046		
7	Blank-2	2015/10/29 下午 04:42	285	34.75	-0.0050		
8	Blank-3	2015/10/29 下午 04:42	284	34.76	-0.0053		
9	peroxideproduct 0.25M				-0.0210	-12.8533	50
10	peroxideproduct 0.25M-1	2015/10/29 下午 04:46	285	34.72	-0.0205	-12.4267	
11	peroxideproduct 0.25M-2	2015/10/29 下午 04:47	286	34.73	-0.0207	-12.5867	
12	peroxideproduct 0.25M-3	2015/10/29 下午 04:47	285	34.74	-0.0219	-13.5467	
13	PEROXIDEPRODUCT-0.25M-1				-0.0225	-14.0000	50
14	PEROXIDEPRODUCT-0.25M-1-1	2015/10/29 下午 04:48	286	34.75	-0.0222	-13.7867	
15	PEROXIDEPRODUCT-0.25M-1-2	2015/10/29 下午 04:49	286	34.75	-0.0226	-14.1067	

No.	Concentration[w/v%]	S.D.	C.V.	Comment
1		0.0002	5.5556	
2				
3				
4				
5		0.0004	7.0709	
6				
7				
8				
9	0.2500	0.6058	4.7128	
10				
11				
12				
13	0.2500	0.1848	1.3197	
14				
15				

No.	Sample Name	Measurement Date	PMT Voltage[V]	Temperature[C]	Optical Rotation Monitor	Specific O.R.	Path Length[mm]
16	PEROXIDEPRODUCT-0.25M-1-3	2015/10/29 下午 04:49	286	34.75	-0.0226	-14.1067	
17	PEROXIDEPRODUCT-0.25M-2				-0.0237	-14.9600	50
18	PEROXIDEPRODUCT-0.25M-2-1	2015/10/29 下午 04:51	286	34.75	-0.0235	-14.8267	
19	PEROXIDEPRODUCT-0.25M-2-2	2015/10/29 下午 04:51	285	34.76	-0.0239	-15.1467	
20	PEROXIDEPRODUCT-0.25M-2-3	2015/10/29 下午 04:52	286	34.76	-0.0236	-14.9067	

No.	Concentration[w/v%]	S.D.	C.V.	Comment
16				
17	0.2500	0.1665	1.1132	
18				
19				
20				

(*R*)-2-hydroperoxy-2-methyltetrahydrofuran (2v) (2.0 M in $CHCl_3$)

No.	Sample Name	Measurement Date	PMT Voltage[V]	Temperature[C]	Optical Rotation Monitor	Specific O.R.	Path Length[mm]
1	Blank				-0.0161		
2	Blank-1	2015/10/29 下午 05:49	283	34.40	-0.0157		
3	Blank-2	2015/10/29 下午 05:49	283	34.41	-0.0162		
4	Blank-3	2015/10/29 下午 05:50	283	34.43	-0.0164		
5	Blank				-0.0162		
6	Blank-1	2015/10/29 下午 05:50	283	34.45	-0.0166		
7	Blank-2	2015/10/29 下午 05:51	283	34.44	-0.0163		
8	Blank-3	2015/10/29 下午 05:51	283	34.45	-0.0158		
9	proxide product 0.2M				-0.1184	-10.2167	50
10	proxide product 0.2M-1	2015/10/29 下午 05:57	285	34.45	-0.1182	-10.1967	
11	proxide product 0.2M-2	2015/10/29 下午 05:57	285	34.45	-0.1183	-10.2067	
12	proxide product 0.2M-3	2015/10/29 下午 05:58	285	34.45	-0.1187	-10.2467	
13	PEROXIDE PRODUCT-0.2M				-0.1191	-10.2833	50
14	PEROXIDE PRODUCT-0.2M-1	2015/10/29 下午 05:59	285	34.46	-0.1190	-10.2767	
15	PEROXIDE PRODUCT-0.2M-2	2015/10/29 下午 05:59	285	34.47	-0.1192	-10.2967	

No.	Concentration[w/v%]	S.D.	C.V.	Comment
1		0.0004	2.2395	
2				
3				
4				
5		0.0004	2.4896	
6				
7				
8				
9	2.0000	0.0265	0.2590	
10				
11				
12				
13	2.0000	0.0115	0.1123	
14				
15				

Supplementary Figure 31**.** Specific optical rotation (SOR)

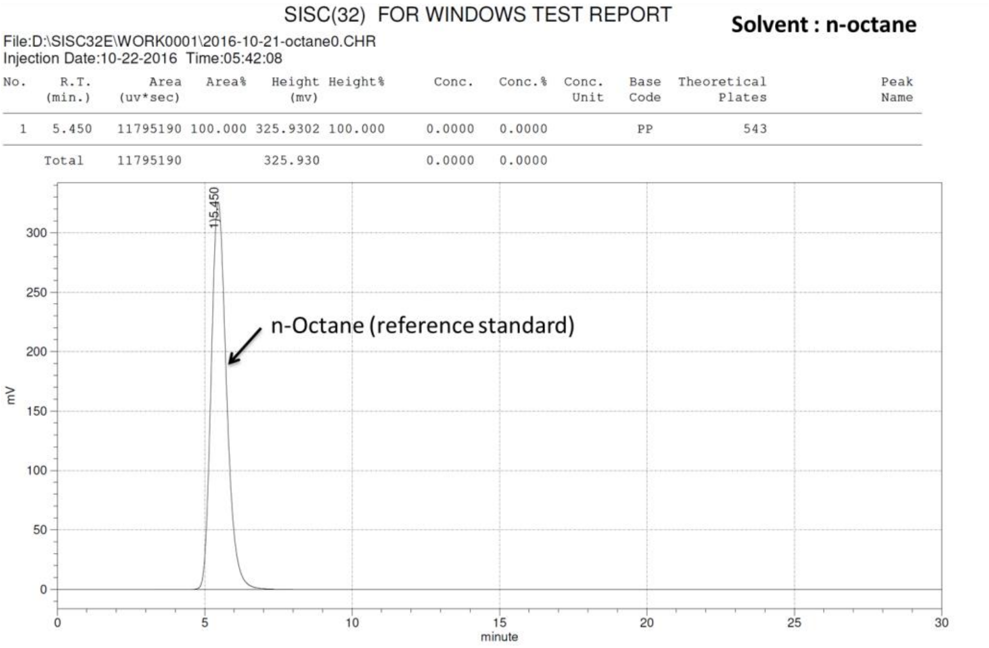

No.	R.T. (min.)	Area (uv*sec)	Area%	Height (mv)	Height%	Conc.	Conc.%	Conc. Unit	Base Code	Theoretical Plates	Peak Name
1	5.450	11795190	100.000	325.9302	100.000	0.0000	0.0000		PP	543	
	Total	11795190		325.930		0.0000	0.0000				

Supplementary Figure 32. GC chromatograph of n-octane (as a reference standard).

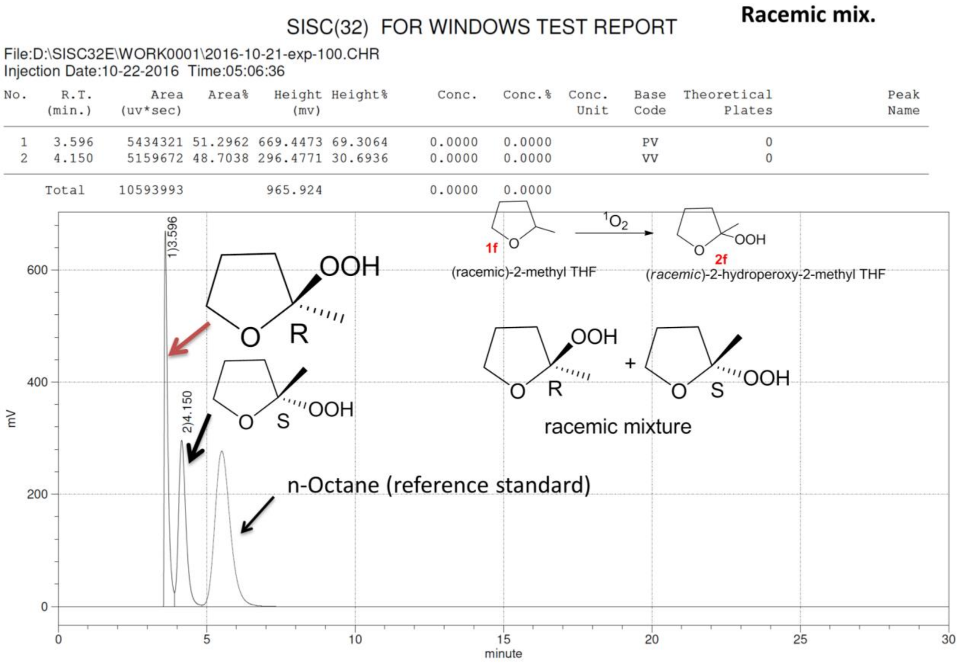

No.	R.T. (min.)	Area (uv*sec)	Area%	Height (mv)	Height%	Conc.	Conc.%	Conc. Unit	Base Code	Theoretical Plates	Peak Name
1	3.596	5434321	51.2962	669.4473	69.3064	0.0000	0.0000		PV	0	
2	4.150	5159672	48.7038	296.4771	30.6936	0.0000	0.0000		VV	0	
	Total	10593993		965.924		0.0000	0.0000				

Supplementary Figure 33. Chiral GC chromatograph of racemic 2-hydroperoxyl-2-methyl-THF (**2f**).

Chiral Product

SISC(32) FOR WINDOWS TEST REPORT

File:D:\SISC32E\WORK0001\2016-10-21-exp-50.CHR
Injection Date:10-22-2016 Time:01:28:38

No.	R.T. (min.)	Area (uv*sec)	Area%	Height (mv)	Height%	Conc.	Conc.%	Conc. Unit	Base Code	Theoretical Plates	Peak Name
1	3.537	4637049	95.5855	566.6483	97.8751	0.0000	0.0000		VV	0	
2	4.050	214157	4.4145	12.3021	2.1249	0.0000	0.0000		VV	0	
	Total	4851206		578.950		0.0000	0.0000				

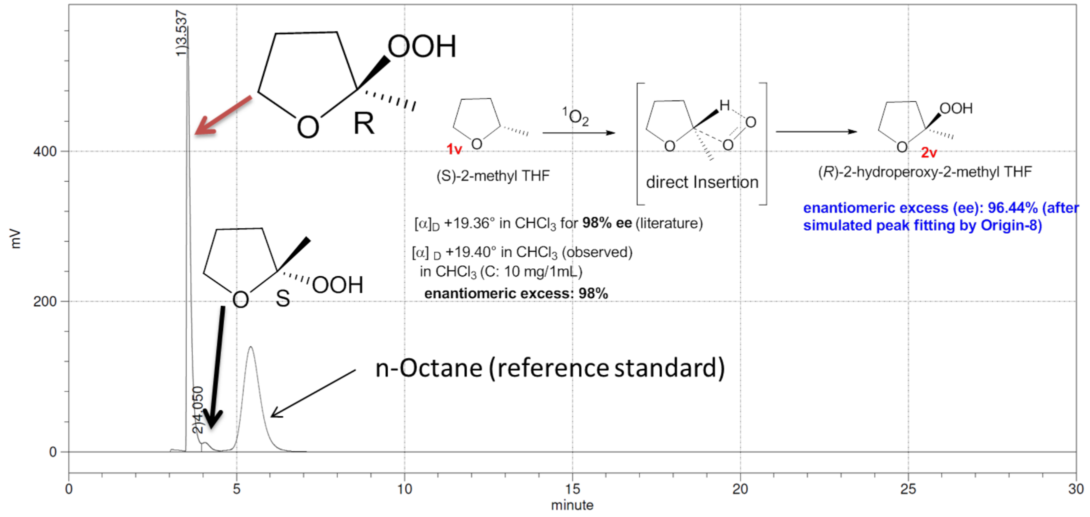

Supplementary Figure 34. Chiral GC chromatograph of (*R*)-2-hydroperoxyl-2-methyl-THF (**2v).**

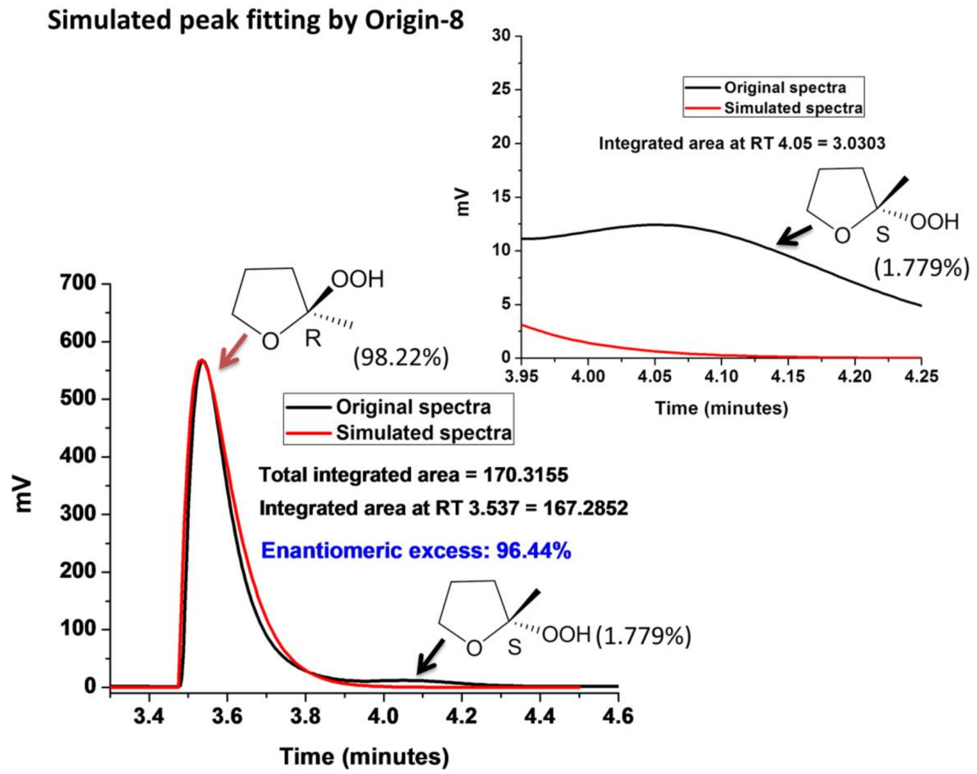

Supplementary Figure 35. Simulated peak fitting by Origin: Chiral GC chromatograph of (*R*)-2-hydroperoxyl-2-methyl-THF-peroxides.

Supplementary Discussion

Determination of singlet O_2 reaction rate with THF. To measure the chemical reaction rate of THF with singlet O_2, a competition reaction was carried out using 1-methylcyclohexene (MCH) as a competing reactant (Supplementary Figure 36a). 1-Methylcyclohexene is known to react with singlet O_2 to generate allyl peroxides with a reaction rate of 0.16 x 10^6 $M^{-1}s^{-1}$ [20]. In the presence of MCH, the amount of the THF product **2a** decreases gradually at higher MCH concentrations in accompany with the formation of MCH oxidation products **2u**, **2u'**, and **2u"**. The amounts of THF product, **2a**, and the MCH products, **2u**, **2u'**, and **2u"**, can be determined using ^{1}H NMR in the presence of known amount of an internal standard, namely, 1,4-dicyanobenzene (Supplementary Figure 36b for the ^{1}H NMR assignments of all products). From the slope of the Supplementary Figure 36c, the chemical reaction rate of singlet O_2 with THF was determined to be ~3.84 x 10^3 $M^{-1}s^{-1}$ (see detailed calculation procedure).

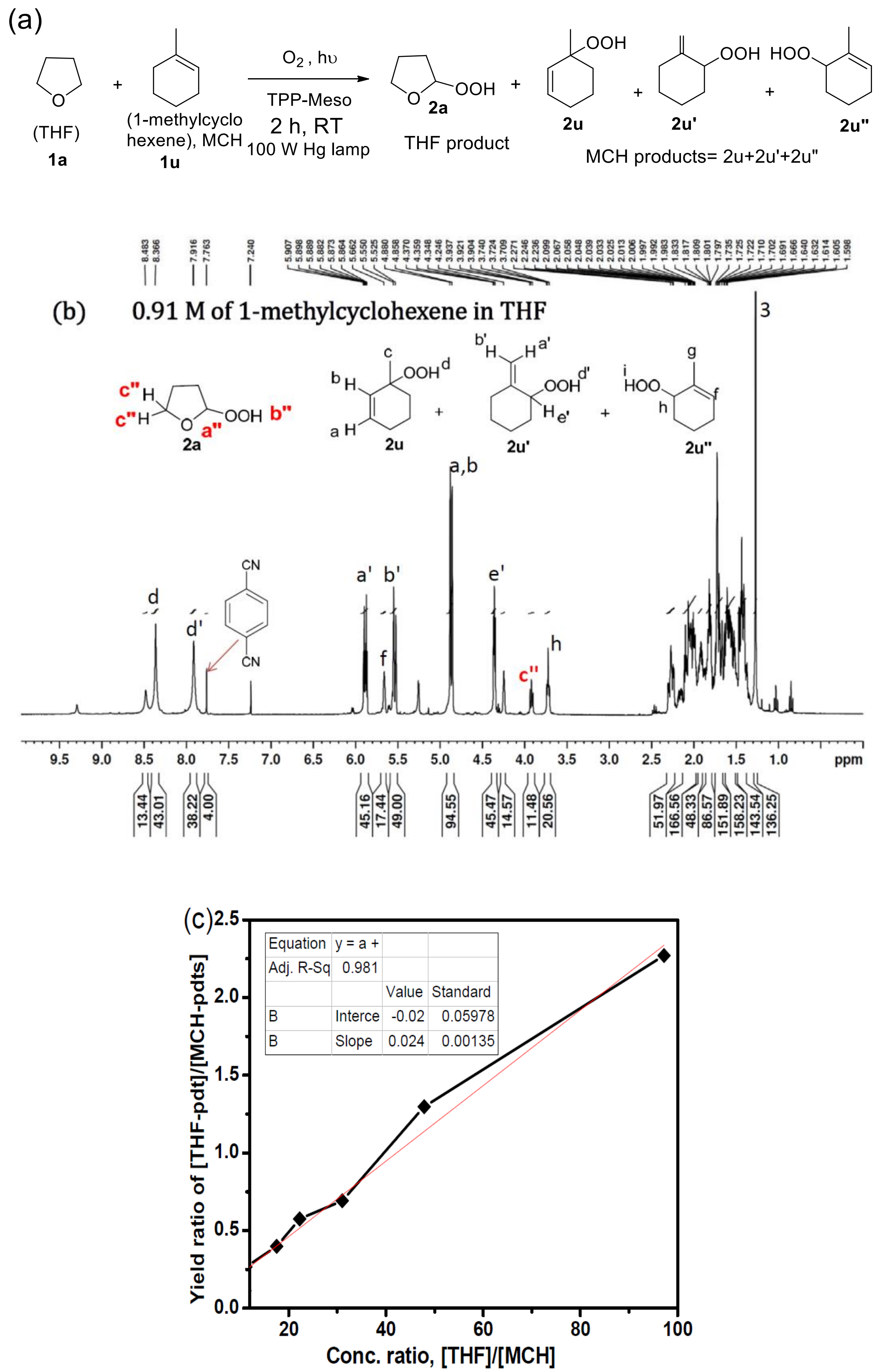

(a)
(THF)
1a
(1-methylcyclo hexene), MCH
1u
O2 , hυ
TPP-Meso
2 h, RT
100 W Hg lamp
OOH
2a
THF product
2u
2u'
2u"
MCH products= 2u+2u'+2u"
(b) 0.91 M of 1-methylcyclohexene in THF
2a
2u
2u'
2u"
a,b
a'
b'
e'
d
d'
f
c"
h
CN
ppm
(c)
Equation
y = a +
Adj. R-Sq
0.981
Value
Standard
B
Interce
-0.02
0.05978
Slope
0.024
0.00135
Yield ratio of [THF-pdt]/[MCH-pdts]
Conc. ratio, [THF]/[MCH]

Supplementary Figure 36. Competition of 1-methylcyclohexene (MCH) with THF to react with singlet O_2. (a) Photo-irradiation of a THF (12.36 M) solution containing different concentrations of MCH. (b) ^1H NMR spectrum and assignments of all reaction products of 1-methylcyclohexene (0.9 M) in THF with singlet O_2. (c) The plot of [THF-product]/[MCH-products] vs. [THF]/[MCH]. The slope is equal to k_1/k_2, where k_1 is the reaction rate of THF with singlet O_2, and k_2 the reaction rate of MCH with singlet O_2.

From Supplementary Figure 36(c), one can obtain a slope value of 0.024, which is equal to the k_1/k_2, where k_1 is the intrinsic chemical reaction rate of THF with singlet oxygen, and k_2 = $0.36 \times 10^6\ M^{-1}s^{-1}$, the intrinsic chemical reaction rate of 1-methylcyclohexene with THF (see the chemical equations derived below). Therefore, one can obtain the chemical reaction rate of THF with singlet O_2 to be $3.8 \times 10^3\ M^{-1}s^{-1}$. Consequently, the lifetime of singlet O_2 in neat THF is 21.06 μs, which is the reciprocal of the apparent rate in neat THF (12.36 M).

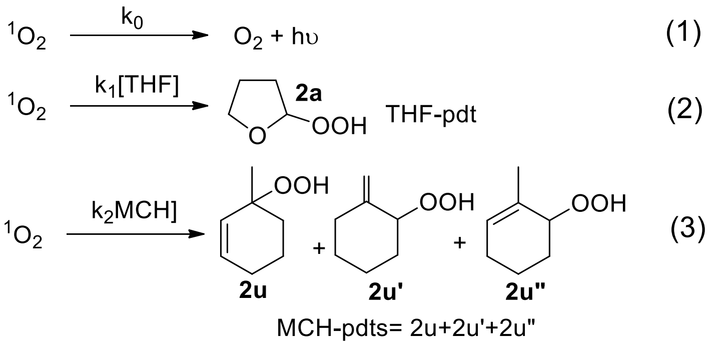

case **a**), in neat THF

$$k_{Total} = k_0 + k_1[THF]$$

$$\Phi_{THF\text{-}pdt} = \frac{k_1[THF]}{k_0 + k_1[THF]}$$

case **b**), in the presence of both THF and MCH

$$k'_{Total} = k_0 + k_1[THF] + k_2\,[MCH]$$

$$\Phi_{THF\text{-}pdt} = \frac{k_1[THF]}{k'_{Total}}$$

$$\Phi_{MCH\text{-}pdts} = \frac{k_2[MCH]}{k'_{Total}}$$

$$\frac{\Phi_{THF\text{-}pdt}}{\Phi_{MCH\text{-}pdts}} = \frac{\dfrac{k_1[THF]}{k'_{Total}}}{\dfrac{k_2[MCH]}{k'_{Total}}} = \frac{k_1[THF]}{k_2\,[MCH]}$$

plot: $\dfrac{\Phi_{THF\text{-}pdt}}{\Phi_{MCH\text{-}pdts}}$ vs. $\dfrac{[THF]}{[MCH]}$; slope= k_1/k_2

reaction rate k_2 for 1-methylcyclohexene= $0.16 \times 10^6\ M^{-1}s^{-1}$

(Ref. Frimer, A. A. Singlet O_2 CRC: Boca Raton, FL, 1985, p203)

from plot, one obtains a slope = 0.024 = $k_1/k_2 = k_1/(0.16 \times 10^6\ M^{-1}s^{-1})$

Therefore, reaction rate of THF with singlet O_2, k_1= = $3.84 \times 10^3\ M^{-1}\ s^{-1}$

$k_{obs.} = k_1 \times [THF]$= = $3.84 \times 10^3\ M^{-1}\ s^{-1} \times 12.36\ M$

= $47.4624 \times 10^3\ s^{-1}$

lifetime (τ)= $1/\ k_{obs.}$ = 21.06 μs (in neat THF)

Quantum yield measurement of singlet oxygen chemical reaction with THF. The chemical quantum yield of singlet O_2 in THF, by definition, can be obtained via two methods:either the ratio of “moles of THF-singlet oxygen reaction products” vs. “the moles of singlet oxygen formed”; or the observed apparent chemical reaction rate (k_1[THF]) of singlet oxygen with THF divided by the total rates ($k_0 + k_1$[THF]) of all physical (k_0) and chemical (k_1[THF]) deactivation channels (see the chemical equation shown below).

$$^{1}O_2 \xrightarrow{k_0} O_2 + h\upsilon \qquad (4)$$

$^{1}O_2 \xrightarrow{k_1[THF]}$ 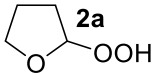 **2a** THF-pdt (5)

In neat THF

$$k_{Total} = k_0 + k_1[THF]$$

$$\text{Chemical quantum yield,}\Phi_{THF\text{-}pdt} = \frac{k_1[THF]}{k_0 + k_1[THF]} \qquad (6)$$

In CCl_4, the physical deactivation rate, k_0 value, is 1/(900 μs)=1.1 x 10^3 s^{-1}. In neat THF, the k_0 value is assumed to be (13/4= 3.25) times faster than for CCl_4, since THF has 13 (C-H, C-C or C-O) single bonds when compared to 4 C-Cl single bonds for CCl_4. To the first approximation, this assumption is valid since solvent nuclear vibrational motion is the main exit channel responsible for the physical deactivation (via electronic-vibrational coupling) of singlet O_2. Therefore, the k_0 value for THF is tentatively assumed to be approximately 1.1 x 10^3 s^{-1} x 3.25= 3.6 x 10^3 s^{-1}. From the competition reaction with 1-methylcyclohexene, we obtained the chemical reaction rate of singlet O_2 with THF to be 3.8 x 10^3 $M^{-1}s^{-1}$. Neat THF has a THF concentration of 12.35 M. Therefore, the chemical quantum yield of product formation for singlet O_2 chemical reaction with THF can be obtained by the Supplementary Equation (6):

$\Phi_{THF,pdts}$= k_1[THF]/ (k_0 + k_1[THF])

= (3.8 x 10^3 $M^{-1}s^{-1}$ x 12.35 M)/ (3.6 x 10^3 s^{-1} + 3.8 x 10^3 $M^{-1}s^{-1}$ x 12.35 M)

= 0.93

The physical deactivation rate of singlet O_2 in THF adopted in the above calculation may not be accurate. Nevertheless, the physical deactivation rate is at least one order slower than the chemical reaction rate of singlet O_2 in THF. Small variation in the physical deactivation rate will not change significantly the chemical quantum yield value (here, 0.93) of singlet O_2 in THF.

Supplementary Figure 37. Check CIF PDF files for compound 3p**.**

checkCIF/PLATON report

Structure factors have been supplied for datablock(s) I

THIS REPORT IS FOR GUIDANCE ONLY. IF USED AS PART OF A REVIEW PROCEDURE FOR PUBLICATION, IT SHOULD NOT REPLACE THE EXPERTISE OF AN EXPERIENCED CRYSTALLOGRAPHIC REFEREE.

No syntax errors found. CIF dictionary Interpreting this report

Datablock: I

```
Bond precision:   C-C = 0.0021 A          Wavelength=0.71073

Cell:              a=7.7471(7)    b=10.8420(11)       c=8.1277(9)
                   alpha=90       beta=112.208(4)     gamma=90
Temperature:       296 K

                  Calculated                Reported
Volume            632.04(11)                632.04(11)
Space group       P 21/c                    P 1 21/c 1
Hall group        -P 2ybc                   -P 2ybc
Moiety formula    C8 H6 O2                  ?
Sum formula       C8 H6 O2                  C8 H6 O2
Mr                134.13                    134.13
Dx,g cm-3         1.410                     1.410
Z                 4                         4
Mu (mm-1)         0.102                     0.102
F000              280.0                     280.0
F000'             280.16
h,k,lmax          9,13,10                   9,13,10
Nref              1301                      1293
Tmin,Tmax         0.980,0.985               0.836,0.949
Tmin'             0.980

Correction method= # Reported T Limits: Tmin=0.836 Tmax=0.949
AbsCorr = MULTI-SCAN

Data completeness= 0.994          Theta(max)= 26.430

R(reflections)= 0.0405( 947)      wR2(reflections)= 0.1431( 1293)

S = 1.180                  Npar= 91
```

The following ALERTS were generated. Each ALERT has the format
test-name_ALERT_alert-type_alert-level.
Click on the hyperlinks for more details of the test.

Supplementary Figure 38. Check CIF PDF file for compound 3s**.**

checkCIF/PLATON report

Structure factors have been supplied for datablock(s) I

THIS REPORT IS FOR GUIDANCE ONLY. IF USED AS PART OF A REVIEW PROCEDURE FOR PUBLICATION, IT SHOULD NOT REPLACE THE EXPERTISE OF AN EXPERIENCED CRYSTALLOGRAPHIC REFEREE.

No syntax errors found. CIF dictionary Interpreting this report

Datablock: I

```
Bond precision:   C-C = 0.0025 A          Wavelength=0.71073

Cell:             a=7.4988(5)     b=10.6861(7)        c=9.4162(7)
                  alpha=90        beta=110.507(3)     gamma=90
Temperature:      100 K

                Calculated                Reported
Volume          706.73(9)                 706.73(8)
Space group     P 21                      P 1 21 1
Hall group      P 2yb                     P 2yb
Moiety formula  C16 H26 O2                ?
Sum formula     C16 H26 O2                C16 H26 O2
Mr              250.37                    250.37
Dx,g cm-3       1.176                     1.177
Z               2                         2
Mu (mm-1)       0.075                     0.075
F000            276.0                     276.0
F000'           276.12
h,k,lmax        9,13,11                   9,13,11
Nref            2901[ 1533]               2329
Tmin,Tmax       0.997,0.998               0.714,0.949
Tmin'           0.989

Correction method= # Reported T Limits: Tmin=0.714 Tmax=0.949
AbsCorr = MULTI-SCAN

Data completeness= 1.52/0.80       Theta(max)= 26.400

R(reflections)= 0.0340( 2080)      wR2(reflections)= 0.0801( 2329)

S = 1.056                  Npar= 167
```

The following ALERTS were generated. Each ALERT has the format
test-name_ALERT_alert-type_alert-level.
Click on the hyperlinks for more details of the test.

Purification of starting materials. Starting materials were purchased at the highest commercial quality and further purification by distillation process or activated alumina column chromatography. Low boiling liquid starting materials (**1a-1k**) were purified by distillation process (distillation procedure followed by known literature methods). Aromatic Starting materials (**1l-1r**) were purified by passing through activated alumina column chromatography under N_2 atmosphere. As requested by one of the reviewer, we have attached the ^{1}H-NMR of purified starting materials (See Supplementary Figures 39-53).

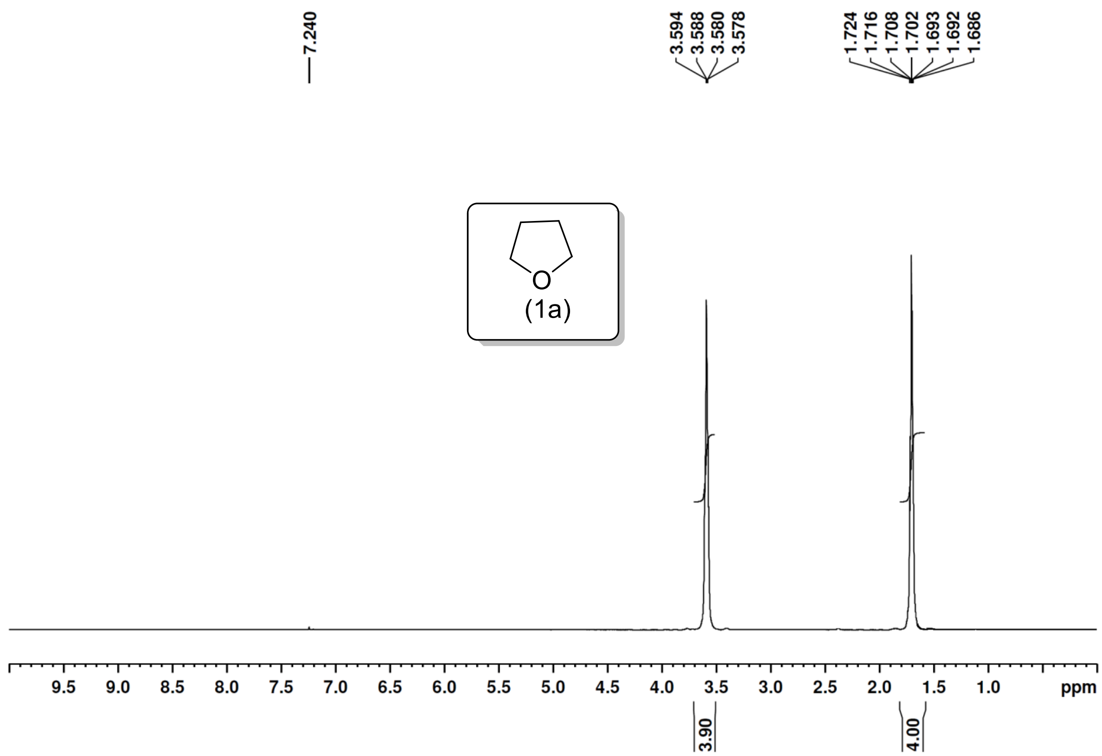

Supplementary Figure 39. ^{1}H NMR spectrum of compound **1a**.

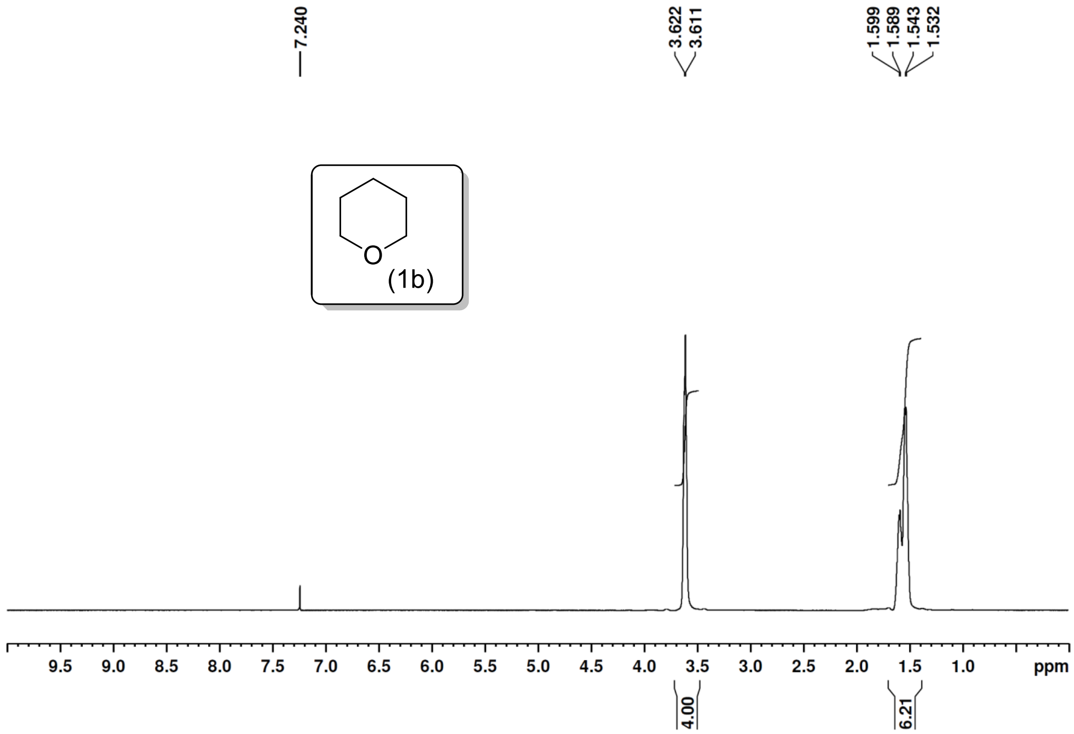

Supplementary Figure 40. ^{1}H NMR spectrum of compound **1b**.

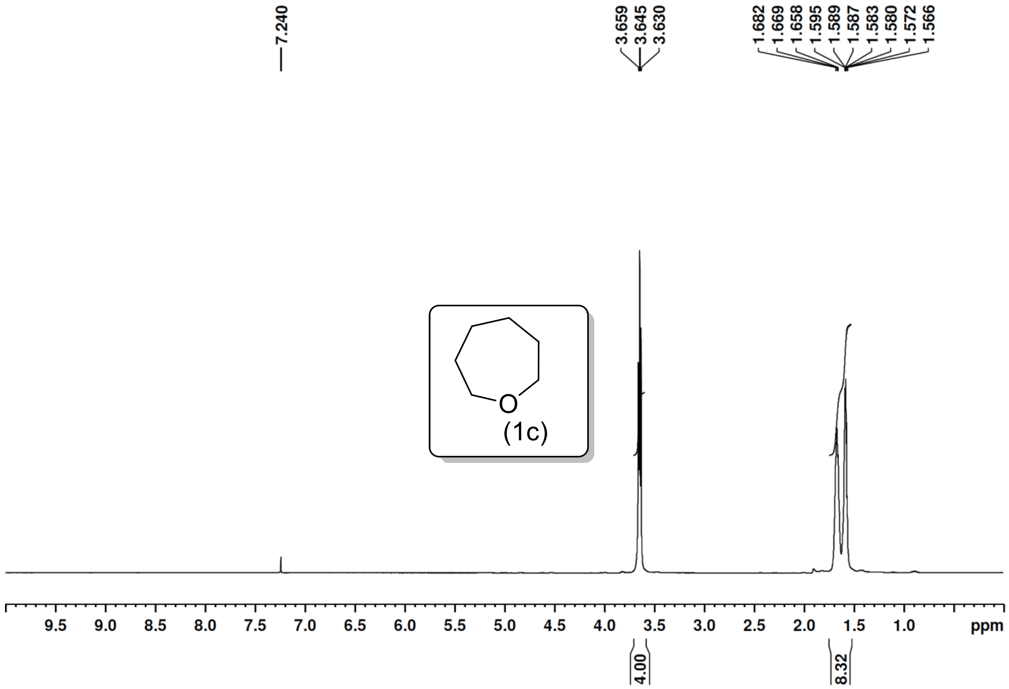

Supplementary Figure 41. ^{1}H NMR spectrum of compound **1c**

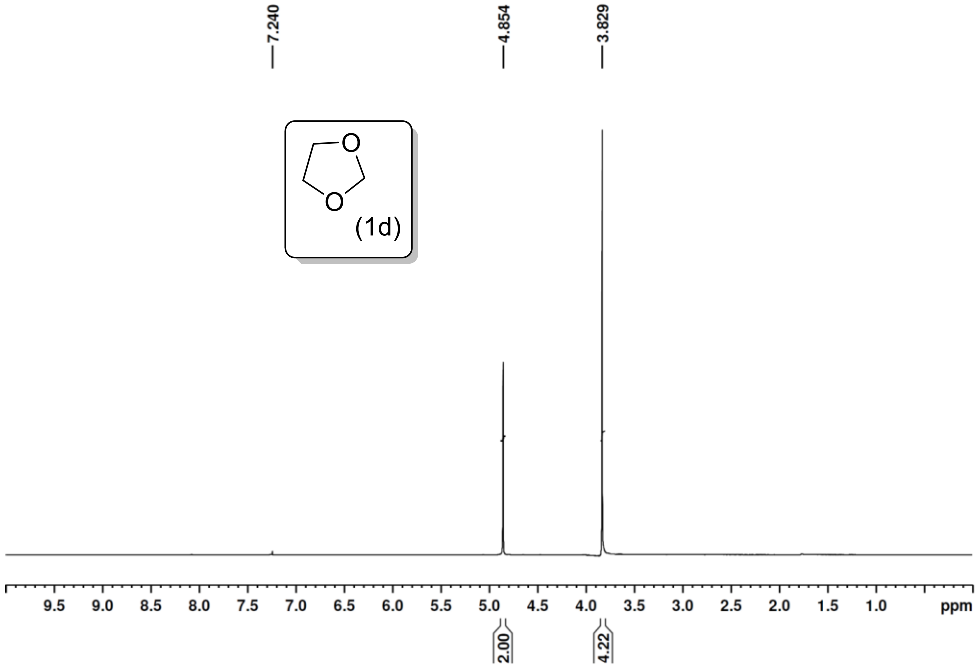

Supplementary Figure 42. ^{1}H NMR spectrum of compound **1d**

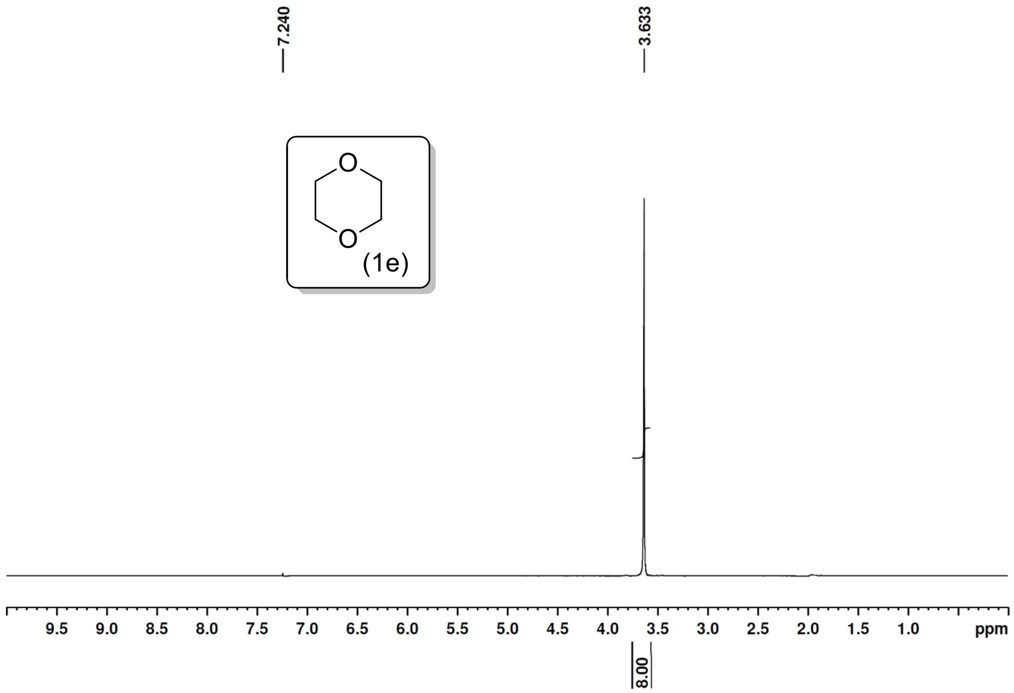

Supplementary Figure 43. ^{1}H NMR spectrum of compound **1e.**

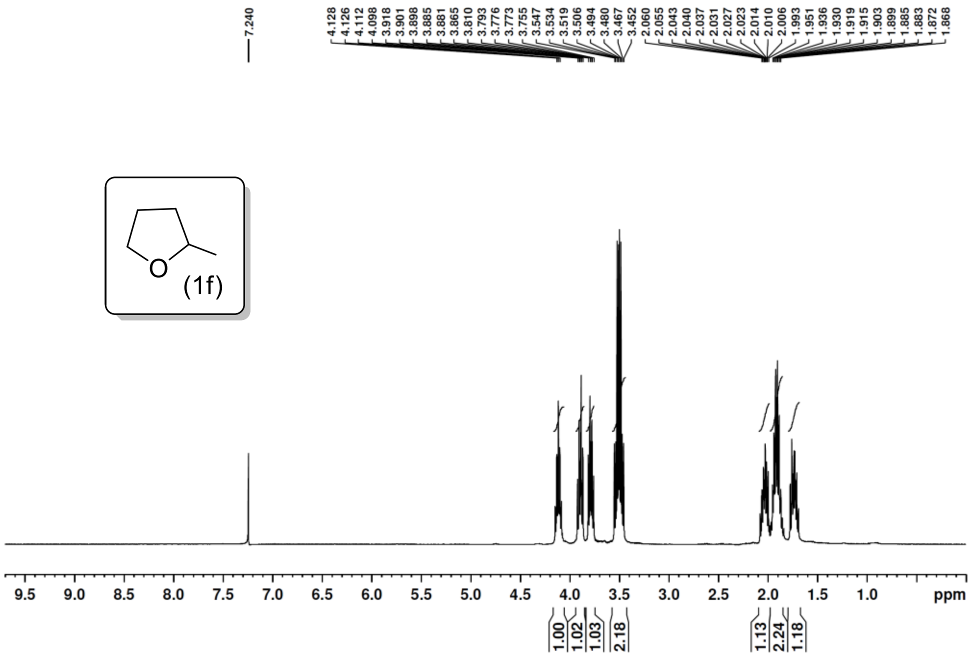

Supplementary Figure 44. ^{1}H NMR spectrum of compound **1f.**

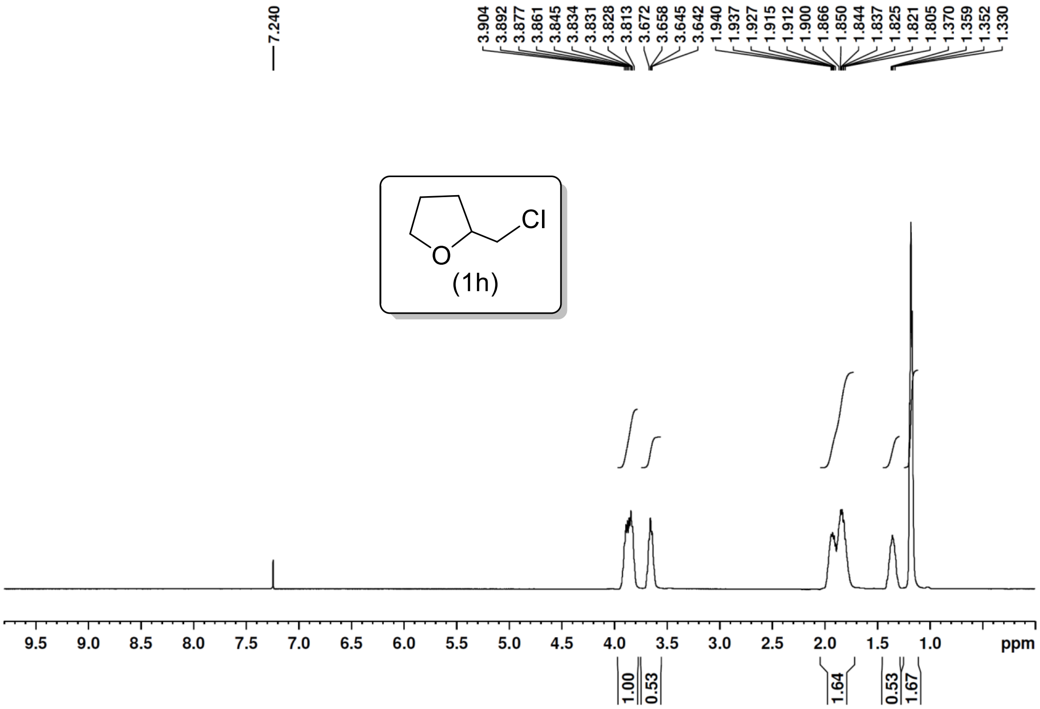

Supplementary Figure 45. ^{1}H NMR spectrum of compound **1h.**

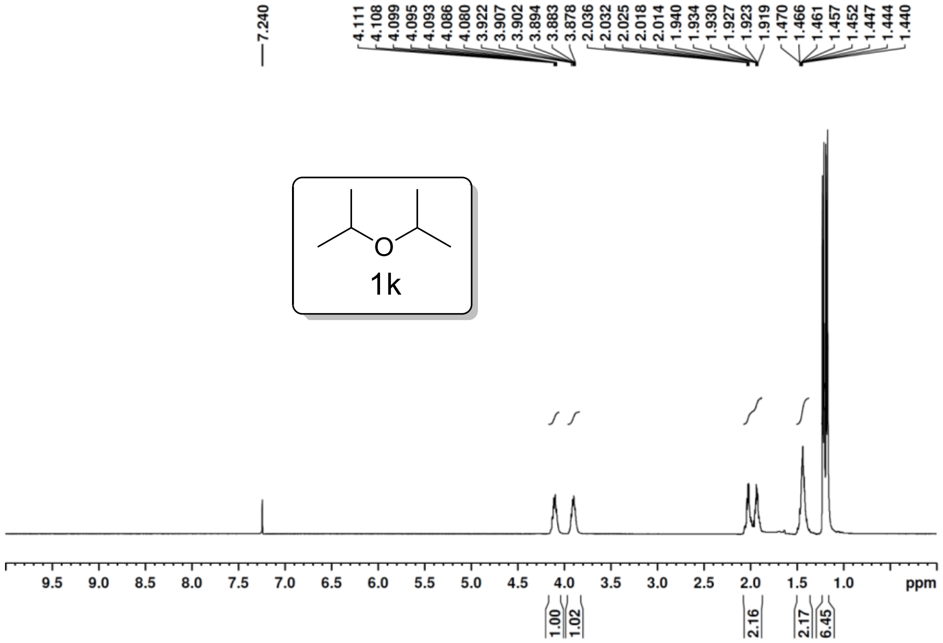

Supplementary Figure 46. ^{1}H NMR spectrum of compound **1k**

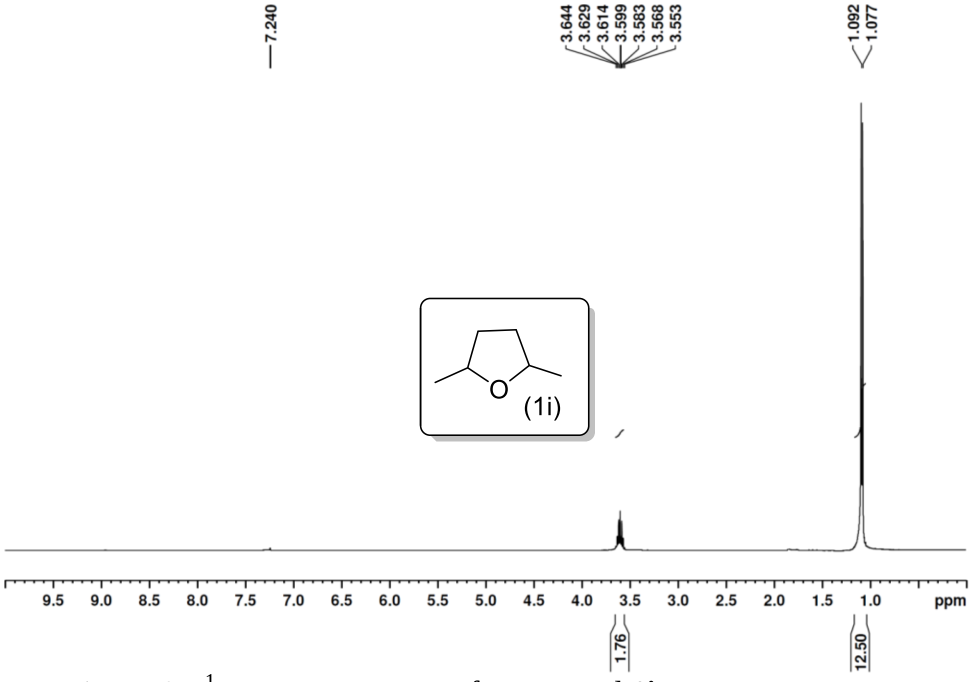

Supplementary Figure 47. ^{1}H NMR spectrum of compound **1i**

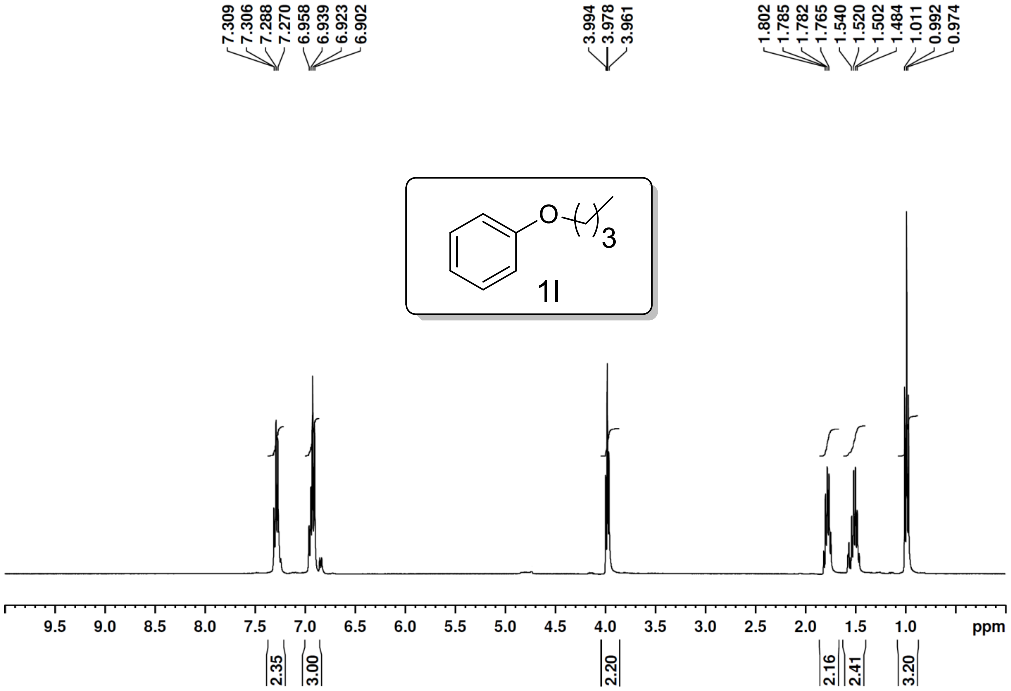

Supplementary Figure 48. ^{1}H NMR spectrum of compound **1j.**

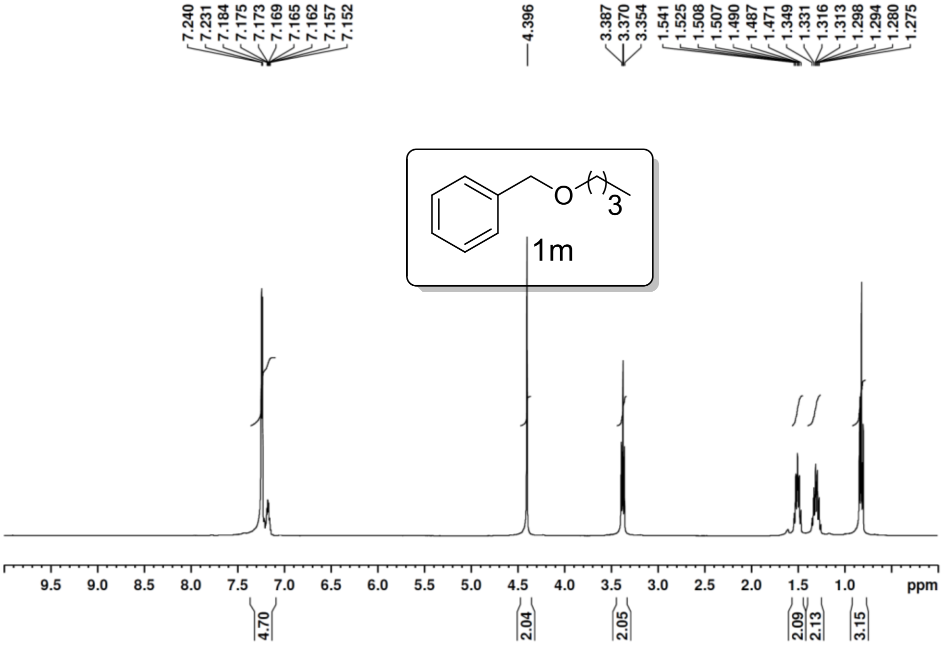

Supplementary Figure 49. ^{1}H NMR spectrum of compound **1m.**

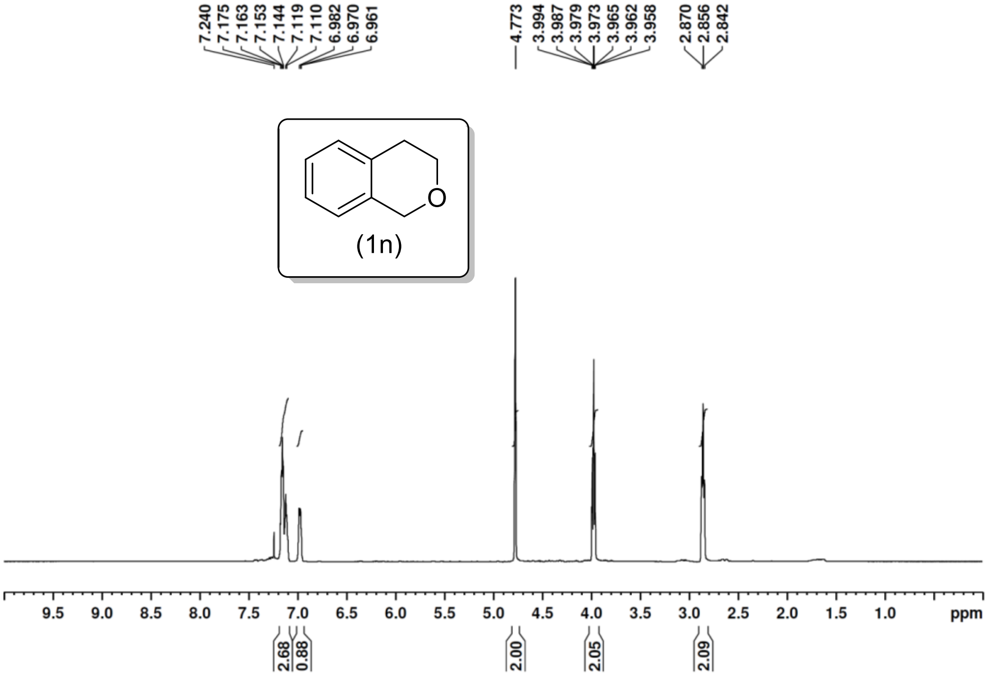

Supplementary Figure 50. ^{1}H NMR spectrum of compound **1n**

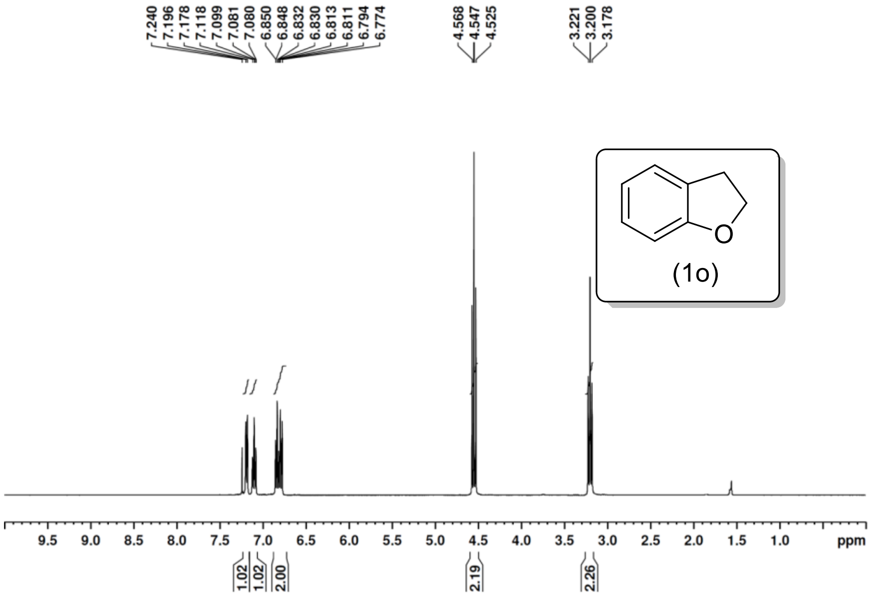

Supplementary Figure 51. ^{1}H NMR spectrum of compound **1o.**

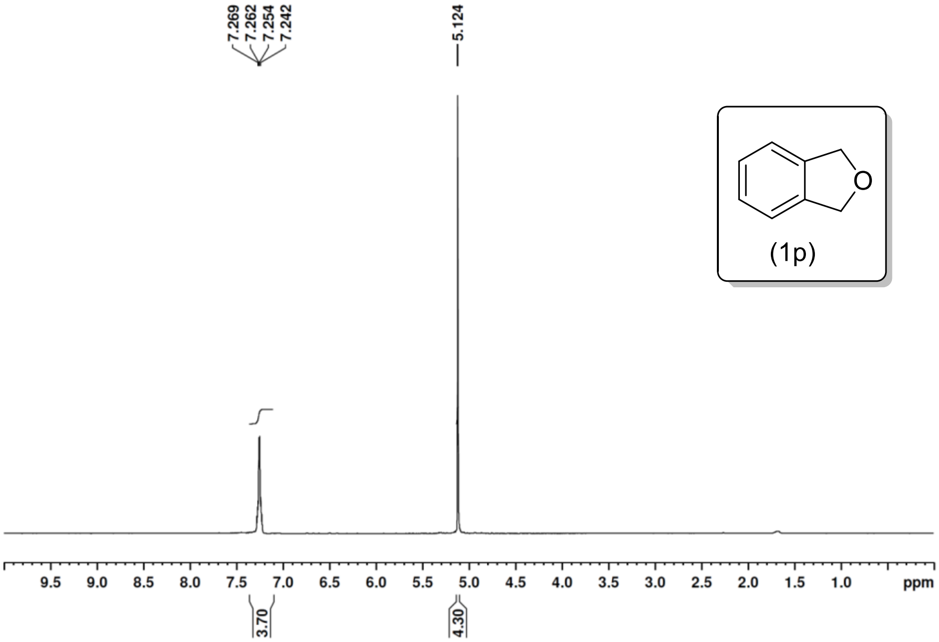

Supplementary Figure 52. ^{1}H NMR spectrum of compound **1p.**

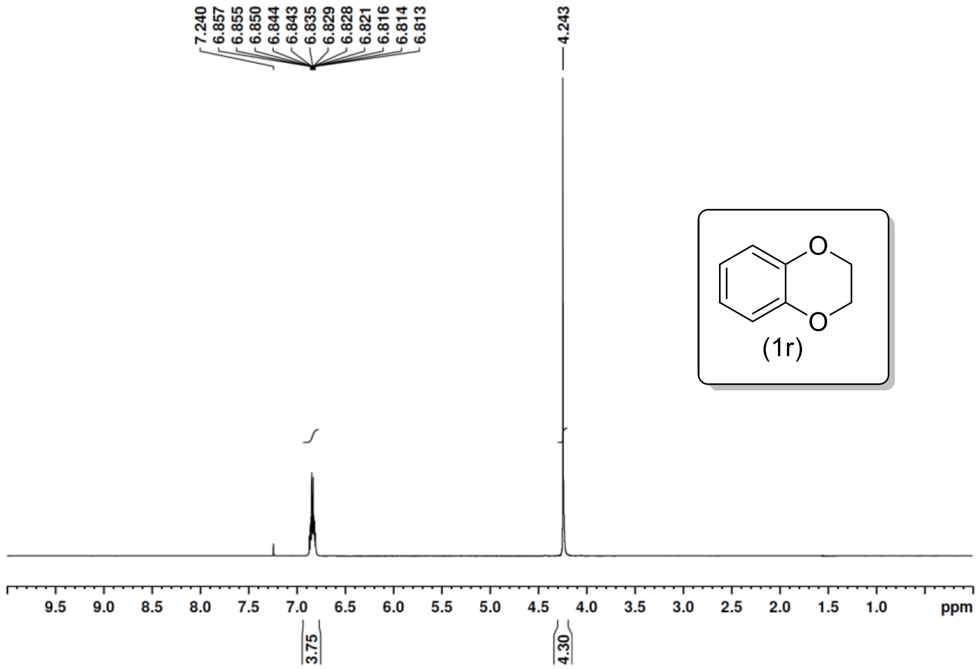

Supplementary Figure 53. ^{1}H NMR spectrum of compound **1r.**

Supplementary References:

1. N. Tada, T. Ishigami, L. Cui, K. Ban, T. Miura, A. Itoh, Calcium iodide catalyzed photooxidative oxylactonization of oxocarboxylic acids using molecular oxygen as terminal oxidant. *Tetrahedron Lett.* **54**, 256-258 (2013).

2. R. F. Moreira, E. Y. Tshuva, S. J. Lippard, Catalytic Oxidative Ring Opening of THF Promoted by a Carboxylate-Bridged Diiron Complex, Triarylphosphines, and Dioxygen. *Inorg. Chem.* **43**, 4427-4434 (2004).

3. F. Kovač, The formation of peroxides from vinyl ethers in the presence of silica-gel. *Tetrahedron Lett.* **42**, 5313-5314 (2001).

4. H. E. Seyfarth, A. Rieche, A. Hesse, Peroxygenierung von Acetalen, I. Peroxygenierung 2-substituierter 1.3-Dioxolane. *Chem. Ber.* **100**, 624-628 (1967).

5. K. Chung, S. M. Banik, A. G. De Crisci, D. M. Pearson, T. R. Blake, J. V. Olsson, A. J. Ingram, R. N. Zare, R. M. Waymouth, Chemoselective Pd-Catalyzed Oxidation of Polyols: Synthetic Scope and Mechanistic Studies. *J. Am. Chem. Soc.* **135**, 7593-7602 (2013).

6. Y. Ishii, S. Sakaguchi, A new strategy for alkane oxidation with O_2 using N-hydroxyphthalimide (NHPI) as a radical catalyst. *Catalysis Surveys from Asia* **3**, 27-35 (1999).

7. S. Sakaguchi, S. Kato, T. Iwahama, Y. Ishii, An Efficient Aerobic Oxidation of Isobutane to t-Butyl Alcohol by N-Hydroxyphthalimide Combined with Co(II) Species. *Bull. Chem. Soc. Jap.* **71**, 1237-1240 (1998).

8. R. Murashige, Y. Hayashi, S. Ohmori, A. Torii, Y. Aizu, Y. Muto, Y. Murai, Y. Oda, M. Hashimoto, Comparisons of O-acylation and Friedel–Crafts acylation of phenols and acyl chlorides and Fries rearrangement of phenyl esters in trifluoromethanesulfonic acid: effective synthesis of optically active homotyrosines. *Tetrahedron* **67**, 64-649 (2011).

9. R. V. Jagadeesh, H. Junge, M.-M. Pohl, J. Radnik, A. Brückner, M. Beller, Selective Oxidation of Alcohols to Esters Using Heterogeneous Co3O4–N@C Catalysts under Mild Conditions. *J. Am. Chem. Soc.* **135**, 10776-10782 (2013).

10. A. Rieche, E. Schmitz, Synthesen von Isochromanyl-Hydroperoxyd und Diisochromanyl-Peroxyden Neue Wege zu Ä therperoxyden. *Chem. Ber*. **90**, 1094-1099 (1957).

11. A.-R. Song, J. Yu, C. Zhang, A Simple and Effective Synthesis of Benzolactones and Benzolactams by Noncatalytic Benzylic Oxidation of Cyclic Benzylic Ethers and N-Protected Cyclic Benzylic Amines with Sodium Chlorite as an Oxidant. *Synthesis* **44**, 2903-2909 (2012).

12. K. Manjulatha, S. Srinivas, N. Mulakayala, D. Rambabu, M. Prabhakar, K. M. Arunasree, M. Alvala, M. V. Basaveswara Rao, M. Pal, Ethylenediamine diacetate (EDDA) mediated synthesis of aurones under ultrasound: Their evaluation as inhibitors of SIRT1. *Bioorg.Med. Chem. Lett.* **22**, 6160-6165 (2012).

13. A. Rieche, M. Schulz, D. Becker, Ozonisierung von Olefinalkoholen. Bildung von Hydroperoxyden cyclischer Ä ther. *Chem. Ber.* **98**, 3627-3631 (1965).

14. X. Xiao, S. Antony, Y. Pommier, M. Cushman, Total Synthesis and Biological Evaluation of 22-Hydroxyacuminatine. *J. Med. Chem.* **49**, 1408-1412 (2006).

15. M. S. Chen, M. C. White, Combined Effects on Selectivity in Fe-Catalyzed Methylene Oxidation. *Science* **327**, 566-571 (2010).

16. D. F. Wyss, A. Arasappan, M. M. Senior, Y.-S. Wang, B. M. Beyer, F. G. Njoroge, M. A. McCoy, Non-Peptidic Small-Molecule Inhibitors of the Single-Chain Hepatitis C Virus NS3 Protease/NS4A Cofactor Complex Discovered by Structure-Based NMR Screening. *J. Med. Chem.* **47**, 2486-2498 (2004).

17. E. Keinan, K. K. Seth, R. Lamed, Organic synthesis with enzymes. 3. TBADH-catalyzed reduction of chloro ketones. Total synthesis of (+)-(S,S)-(cis-6-methyltetrahydropyran-2-yl)acetic acid: a civet constituent. *J. Am. Chem. Soc.* **108**, 3474-3480 (1986).

18. E. J. Corey, S. Shibata, R. K. Bakshi, An efficient and catalytically enantioselective route to (S)-(-)-phenyloxirane. *J. Org. Chem.* **53**, 2861-2863 (1988).

19. V. Schurig, W. Biirkle**,** Extending the Scope of Enantiomer Resolution by Complexation Gas Chromatography. *J. Am. Chem. SOC.* **104**, 1513-1580 (1982).

20. Rodgers, M. J. A. Solvent-induced deactivation of singlet oxygen: additivity relationships in nonaromatic solvents. *J. Am. Chem. Soc*. **105**, 6201-6205 (1983).
