# Supplementary material for: Singlet oxygen-mediated selective C–H bond hydroperoxidation of ethereal hydrocarbons
Source: Nat Commun. 2017 Nov 27;8:1812. doi: 10.1038/s41467-017-01906-5 (PMC5703888; doi:10.1038/s41467-017-01906-5)
Supplement: Supplementary file 2 — Peer Review File [file 41467_2017_1906_MOESM2_ESM.pdf]

Reviewer #1 (Remarks to the Author):

In this manuscript, the authors reported a hydroperoxylation reaction of ethereal C-H bonds using singlet oxygen as oxidant. A series of simple substrates containing ethereal C-H bonds are examined in the manuscript under singlet oxygen/photo-sensitizer/Lewis acid conditions to give the corresponding hydroperoxidation/lactonization products at α-ethereal positions.

1) Using singlet oxygen to activate allylic C-H bonds has been extensively studied in literature. Ethereal C-H bonds are also highly activated (via hyperconjugation) and not "inert" as the authors claim. These types of bonds are highly suseptable to C-H oxidation with a wide range of oxidants, including electrophilic transition metal catalysts with H2O2 as well as organic oxidants such as DMDO. Moreover, it is well established that such active ethereal bonds in oxygen-containing solvents can be oxidized when exposed to air and light. Therefore, this transformation is not novel and can be performed easily with numerous other oxidants and with no advantages to this opertationally difficult system are apparent.

2) From a practical standpiont, the yields of this operationally difficult transformation are generally low and do not compete with the other state-of-the-art ways to do this. In Table 1 and Table 2, eight of the 17 reported yields are below 50%. It is hard to justify the "high yields" claimed by the authors in abstract. In most cases when a methylene ethereal site is oxidized by singlet oxygen, mixtures of hydroperoxylation and ester products are formed which further lower the yield of a single product and limit the synthetic utility of this transformation.

3) The mechanism study with chiral 2-methyl THF is not conclusive. The authors only present only the rotation of the hydroperoxylation product as evidence to conclude that the reaction proceed via stereoretentive direct insertion process. This a very irregular way to do this. These data are insufficient to draw such conclusion and comparison of the level of enantioenrichment of both the starting material (2-methyl THF) and the product (2-hydroperoxyl-2-methyl THF) is necessary. The enantiomeric excess of the product must be the same as that of the starting material to make the conclusion the authors are making.

4) The authors conclude that the site selectivity of singlet oxygen and "electrophilic organometallic catalyst/hydrogen peroxide systems" are orthogonal in the discussion section of the manuscript. In fact, the singlet oxygen/electrophilic organometallic catalyst systems give the exact same site of oxidation when ethereal substrates are employed. Cyclic ethers are oxidized to the corresponding lactones and (-)-ambroxide is oxidized to (+)-sclareolide in both systems.

In conclusion, the results reports in this manuscripts do not fit the novelty and impact required for manuscripts published in Nature Communications. I would suggest the authors make the changes listed above and look to publish in a more specialized chemistry journal.

Reviewer #2 (Remarks to the Author):

If the claims of the authors can be properly substantiated, this would indeed be a paper worthy of publication in a high profile journal. However, I found that many alternative explanations could be easily provided to account for their data. In short, the authors need to provide the results of more control experiments to establish credence in their claims (suggestions are listed below).

Moreover, the authors' credibility is easily questioned based solely on the fact that they refer to and cite dated material. They need to do a better search of the literature on singlet oxygen that has been published in the last 30 years, and present arguments that demonstrate a command and understanding of this material.

Some specific comments:

1. It was good to see the authors recognize that THF (and other ethers) readily form hydroperoxides. However, it is imperative that they demonstrate the complete absence of impurities in their reactants. Ethers are notoriously "dirty".

2. All of the systems investigated involve the presence of other molecules (in particular, sensitizers). The authors need to exclude the possibility that the singlet oxygen produced first reacts with the sensitizer which then, via a secondary process (e.g., decomposition of a sensitizer hydroperoxide), oxidizes the ether.

3. It was nice to see that the authors cited some early work on charge-transfer (CT) states. However, this direction needs to be more fully investigated. This includes CT states involving the sensitizers.

4. With respect to the material on the top of page 5, I have no doubt that singlet oxygen is formed. However, the five points listed by the authors are consistent with many different, and more believable, mechanisms.

5. Were the yields shown in Tables 1 and 2 independent of the sensitizer used? What happens if a radical trap is added to the solution?

6. Here is another meaningful experiment that could be (should be) done. It has recently been shown that irradiation at 765 nm of a neat (i.e., sensitizer-free) solvent that contains oxygen will generate singlet oxygen. This is a consequence of pumping a transition in oxygen itself. In this way, one cleanly generates singlet oxygen, and singlet oxygen alone. If the authors produce singlet oxygen in this way, and still see the oxidation of their ethers, I will be more inclined to look deeper into their claims.

In conclusion, I regret to report that this paper is not ready to be published.

Reviewer #3 (Remarks to the Author):

This work describes the selective oxidation of ethers at the alpha position to hydroperoxides and esters and provides good evidence that the reaction is singlet oxygen mediated. To the best of my knowledge, this is a novel reaction of singlet oxygen (assuming the authors are correct) and it is therefore of broad general interest.

The English could use some work in the editing process, but is basically clear enough to be read and understood.

I think the paper should be accepted after some revision. Here are some points to consider.

I do not think the introduction is very clear on what the authors think was anomalous about the singlet oxygen lifetime data in the literature and the general theory about vibrational deactivation in many solvents. I knew Chris Foote well, and I think I would have known if he thought THF was some weird case. Many lifetime discrepancies have been demonstrated to be impurity related because the impurities are so much more efficient quenchers of singlet oxygen than the main solvent. For example, Oliveros and Braun find a quenching rate constant of < 7 x 10^3 for methyl t-butyl ether (which is totally in line with these results), but note that lifetimes in that solvent vary with small amounts of alkene impurity. When I was active in singlet oxygen chemistry, impurity quenching was

the broad conventional wisdom for the lifetime in several solvents. The authors need to be more clear about whether they think ethers break the traditional vibrational model or fit right in...and hit it again in the discussion.

I was surprised the authors did not report an experiment using d8-THF, which (although expensive) is commercially available.

The reported rate constant should only be given to 2 places, especially given that the reference rate is only given to two. and an "about tilde" is used.

In my version of Table 2, there are some stray remarks and figures. This could be a pdf artifact, but please check.

Why these two particular Lewis Acids? Are these optimized or just two random choices?

Is there good evidence that the complexation of singlet oxygen by the Lewis Acid is not involved? The theoretical modeling does not include any Lewis Acid.

The authors need to be much clearer in the main text that the molar optical rotations of methylTHF and the hydroperoxide are known values, because that makes the retention of configuration argument much stronger. (This is very good evidence for a one step mechanism.)

Figure 3 should make clear what the computational method was. A couple different basis sets are noted in the text. I assume that they are the MP2-CASSCF, rather than just CASSCF.

The discussion is really unclear. I don't understand at all what is meant by "the selectivity of singlet O2 is most probably governed by the singlet spin multiplicity". By definition, singlet oxygen is a singlet, so there must be something more profound in here that I am missing. I think the authors would do well to give singlet oxygen chemists who have missed this reaction for decades a better physical interpretation of what they think is going on.

Finally, the authors quote a rate constant for "chemical reaction rate" of THF with singlet oxygen as 3.8 x 10^3. They should be very, very explicit about "chemical reaction" vs "deactivation". A number

of compounds physically deactivate singlet oxygen at a much higher rate than they "net" react with it. In other words the chemical efficiency of the quenching event is pretty low. The authors seem to be saying that is not the case here, in that they are saying that the "chemical reaction rate" is compatible with the lifetime in neat solvent. That, of course, implies a quantum yield of near 1 (after formation of singlet oxygen). That may be exactly what they mean, but it would be all the more remarkable that this reaction was missed for so long if it is the case. An explicit statement to that effect or measurement of the chemical quantum yield is definitely reasonable to expect.

Response to Referees' letter (**manuscript NCOMMS-16-05981)**

Reviewer #1 (Remarks to the Author):

In this manuscript, the authors reported a hydroperoxylation reaction of ethereal C-H bonds using singlet oxygen as oxidant. A series of simple substrates containing ethereal C-H bonds are examined in the manuscript under singlet oxygen/photo-sensitizer/Lewis acid conditions to give the corresponding hydroperoxidation/lactonization products at α-ethereal positions.

Q1) Using singlet oxygen to activate allylic C-H bonds has been extensively studied in literature. Ethereal C-H bonds are also highly activated (via hyperconjugation) and not "inert" as the authors claim. These types of bonds are highly suseptable to C-H oxidation with a wide range of oxidants, including electrophilic transition metal catalysts with H_2O_2 as well as organic oxidants such as DMDO. Moreover, it is well established that such active ethereal bonds in oxygen-containing solvents can be oxidized when exposed to air and light. Therefore, this transformation is not novel and can be performed easily with numerous other oxidants and with no advantages to this opertationally difficult system are apparent.

Our reply: We respectfully disagree with the comment "Using singlet oxygen to activate allylic C-H bonds has been extensively studied in literature". Singlet oxygen was never reported in the literature to be able to activate allylic C-H bond. As we stated in the manuscript, singlet oxygen was well known to react with olefin. The reaction mechanism of singlet O_2-ene reaction is well documented in the literature (see, J. Am. Chem. Soc. 1980, 102, 439-449; and Ace. ***Chem. Res.*** 1980, 13, 419-425). As shown in the scheme below that singlet oxygen attacks the C=C double bond to generate hydroperoxyl radical intermediates **20** and **21**, which then abstract allylic proton to generate allylic peroxides. Singlet oxygen molecule does not attack or activate directly at the allylic C-H bond position; it is the hydroperoxyl radical intermediate that abstracts the allylic proton. Overall, it looks that singlet O_2 activates the allylic C-H bond to generate allylic hydroperoxides, but it is NOT the case. Key features of such a singlet O_2-ene reaction are (a) hydroperoxide moiety locates at the original C=C double bond positions, and (b) C=C double bond shifts to the adjacent position to the original C=C double bond position. Therefore, one of our key claims in the manuscript that singlet O_2 was never reported in the literature to be able to activate C-H bonds, remains valid.

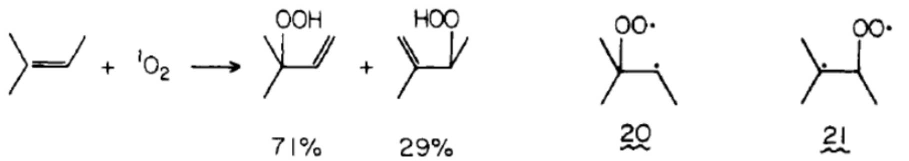

(reaction scheme adopted from J. Am. Chem. Soc. 1980, 102, 439-449; and **Acc. *Chem. Res.*** 1980, 13, 419-425).

Yes, we agree that alpha C-H bonds in ethereal compounds are not as inert as those in

pure hydrocarbons and many combinations of oxidants are able to oxidize and functionalize alpha ethereal C-H bonds. However, we are NOT trying to report a new method to activate/oxidize/functionalize alpha-ethereal C-H bonds. We are trying to report **unprecedented, totally new singlet oxygen chemistry**, that is, selective C-H bond functionalization of ethereal hydrocarbons by singlet oxygen, a non-organometallic reagent, at a very mild condition of room temperature and 1 atmosphere.

Yes, we agree that it is well established that such "not-so-inert" ethereal bonds in oxygen-containing solvents can be oxidized when exposed to air and light. We have discussed this point in the main text and cited related work as reference 18. As we discussed in the main text that it is commonly known that 6~12 months exposure of open-canned THF to air and room light will result in formation of THF-hydroperoxide (but not tetrahydrofuran-2(3H)-one),(*18*) which was once proposed, without experimental evidences, to be due to photo-excitation of a O_2-THF ground state charge transfer complex. It was never proposed or proved that the formation of hydroperoxide is related to singlet O_2.

Q2) From a practical standpiont, the yields of this operationally difficult transformation are generally low and do not compete with the other state-of-the-art ways to do this. In Table 1 and Table 2, eight of the 17 reported yields are below 50%. It is hard to justify the "high yields" claimed by the authors in abstract. In most cases when a methylene ethereal site is oxidized by singlet oxygen, mixtures of hydroperoxylation and ester products are formed which further lower the yield of a single product and limit the synthetic utility of this transformation.

Our reply: We are aware of many existing processes for preparation of hydroperoxides and lactone. As our reply to Q1, we are not trying to report a new way to prepare hydroperoxides and lactones from ethereal compounds using singlet oxygen, albeit the hydroperoxides generated from this unprecedented singlet oxygen reaction is chiral and is different from literature methods. One of the key novelties of our current work is the **discovery of an unprecedented, totally new singlet oxygen reaction**, that is, selective C-H bond functionalization of ethereal hydrocarbons by a non-organometallic reagent, namely, singlet oxygen. Implication of such a new discovery is that it might be possible that in addition to so-called type I and type II reaction processes, singlet oxygen mediated photodynamic therapy might involve direct oxidation of saturated hydrocarbons, saturated lipids/proteins/DNA to generate hydropoxides inside cancer cells. The hydroperoxides then further decompose to generate alkoxyl and hydroxyl radicals, leading to intensive oxidative stress and cancer cellular deaths.

Yes, we agree that many reactions listed in the Tables 1 and 2 have yields lower than 50%. Therefore, we have changed the word "high yield" in the abstract to "modest to high yield". In fact, the "low yields" can be further boosted up if given a longer reaction time. These yields listed in Tables 1 & 2 are not optimized. Our purpose is to demonstrate that singlet oxygen indeed can react with various ethereal compounds, via selective C-H functionalization, which can explain the 35 years pending literature

puzzle, that is, huge variation of singlet oxygen lifetimes in different solvents.

Q3) The mechanism study with chiral 2-methyl THF is not conclusive. The authors only present only the rotation of the hydroperoxylation product as evidence to conclude that the reaction proceed via stereo-retentive direct insertion process. This a very irregular way to do this. These data are insufficient to draw such conclusion and comparison of the level of enantioenrichment of both the starting material (2-methyl THF) and the product (2-hydroperoxyl-2-methyl THF) is necessary. The enantiomeric excess of the product must be the same as that of the starting material to make the conclusion the authors are making.

Our reply: Yes, we agree that the measurement of optical rotation value, without comparison with molar optical rotation value, is not conclusive to prove 100% conservation of stereo-configuration. The molar optical rotational angle value of the product, i.e., chiral 2-hydroperoxyl-2-methyl-THF, however, is not available in the literature. To resolve the problem, we have to determine the enantiomeric excess values for both the starting chiral (S)-2-methyl-THF and the product (R)-2-hydroperoxyl-2-methyl-THF. We followed a literature procedure (ref. 24) to prepare the chiral (S)-2-methyl-THF. The optical rotation value of our as-synthesized chiral (S)-2-methyl-THF is $[a]_D$ +19.40° in $CHCl_3$, observed, 10 mg/mL, which is nearly the same as that ($[a]_D$ +19.36° in $CHCl_3$, 10 mg/mL) of literature reported value for 98% enatiomeric excessed (S)-2-methyl-THF (ref. 23). Therefore, our starting chiral (S)-2-methyl-THF is 98% enatiomeric excess. To determine the enantiomeric excess of the (R)-2-hydroperoxyl-2-methyl-THF product, we followed a literature procedure (*J. Am. Chem. SOC.* **1982,** *104*,1513-1580) to prepare a chiral nickel(II) bis[(1R)-3-(hepta-fluorobutyryl)camphorates] complex-based GC column for separation of the R and S forms of racemic 2-hydroperoxyl-2-methyl-THF (**2f**) products. As shown in the GC spectra below, racemic 2-hydroperoxyl-2-methyl-THF can be well separated by the Ni complex-based chiral column. Details of procedure for preparation of chiral Ni-complex, coating of column, and instrumental analysis condition were discussed in revised supporting information (page no: S15 and S59). The GC spectrum of the hydroperoxides obtained from reaction of (S)-2-methyl THF (98% ee) with singlet oxygen shows the dominant presence of (R)-2-hydroperoxyl-2-methyl-THF (96.4% enantiomeric excess) with minor amount of (S)-2-hydroperoxyl-2-methyl-THF, which is very close to the enatiomeric excess (98% ee) value of the starting substrate. Therefore, the data strongly support our conclusion that singlet oxygen directly inserts into the alpha ethereal C-H bond in a one-step manner, leading to the conservation of stereo-configuration of the starting substrate. We have incorporated the above data to supplementary information as Figure S4~S7.

Scheme S1. Enatiomeric excess values of the starting (S)-2-methyl THF and its reaction products with singlet oxygen.

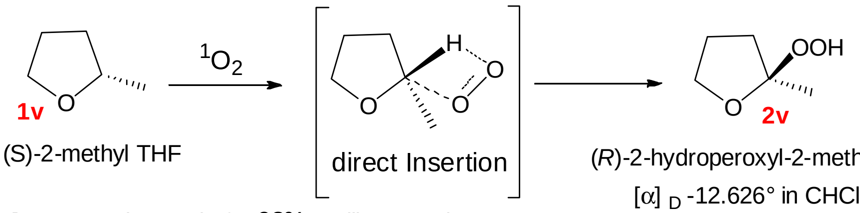

(1)

$[\alpha]_D$ -12.626° in $CHCl_3$ (C: 10 mg/mL)

$[\alpha]_D$ +19.36° in CHCl3 for **98% ee** (literature)

$[\alpha]_D$ +19.40° in $CHCl_3$ (observed)
in $CHCl_3$ (C: 10 mg/mL)

enantiomeric excess (ee): 98% (literature)

enantiomeric excess (ee): 98% (observed)

enantiomeric excess (ee): 96.44% after simulated peak fitting by Origin-8

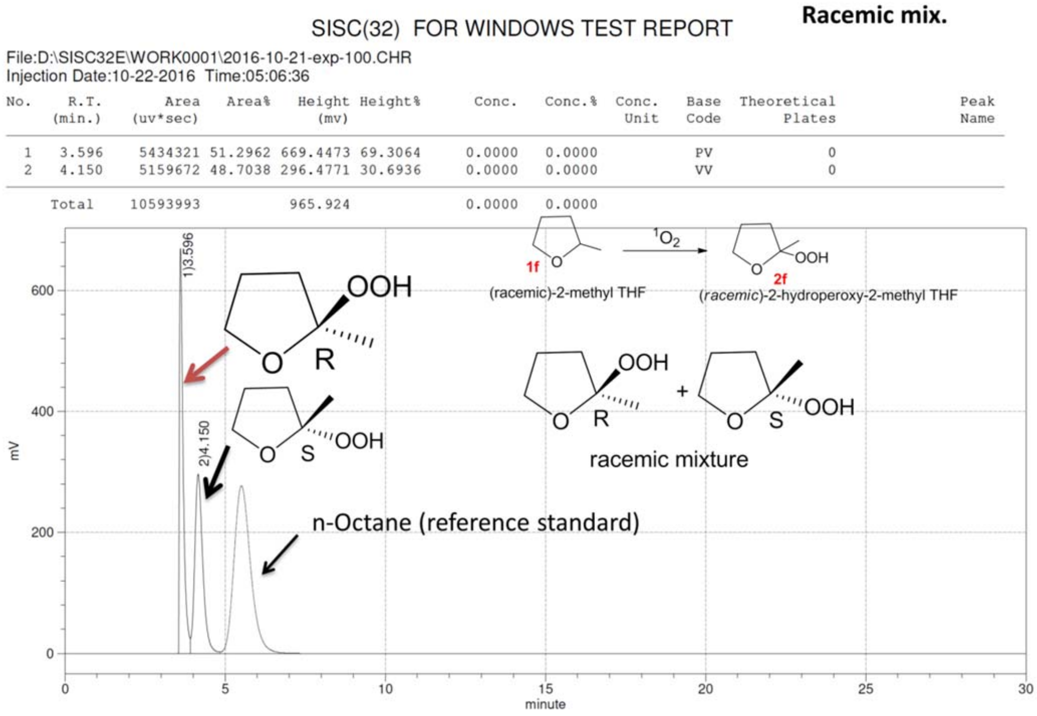
SISC(32) FOR WINDOWS TEST REPORT

Racemic mix.

File:D:\SISC32E\WORK0001\2016-10-21-exp-100.CHR
Injection Date:10-22-2016 Time:05:06:36

No.	R.T. (min.)	Area (uv*sec)	Area%	Height (mv)	Height%	Conc.	Conc.%	Conc. Unit	Base Code	Theoretical Plates	Peak Name
1	3.596	5434321	51.2962	669.4473	69.3064	0.0000	0.0000		PV	0	
2	4.150	5159672	48.7038	296.4771	30.6936	0.0000	0.0000		VV	0	
Total		10593993		965.924		0.0000	0.0000				

Figure S5. Chiral GC chromatograph of racemic 2-hydroperoxyl-2-methyl-THF (**2f**).

Chiral Product

SISC(32) FOR WINDOWS TEST REPORT

File:D:\SISC32E\WORK0001\2016-10-21-exp-50.CHR
Injection Date:10-22-2016 Time:01:28:38

No.	R.T. (min.)	Area (uv*sec)	Area%	Height (mv)	Height%	Conc.	Conc.%	Conc. Unit	Base Code	Theoretical Plates	Peak Name
1	3.537	4637049	95.5855	566.6483	97.8751	0.0000	0.0000		VV	0	
2	4.050	214157	4.4145	12.3021	2.1249	0.0000	0.0000		VV	0	
	Total	4851206		578.950		0.0000	0.0000				

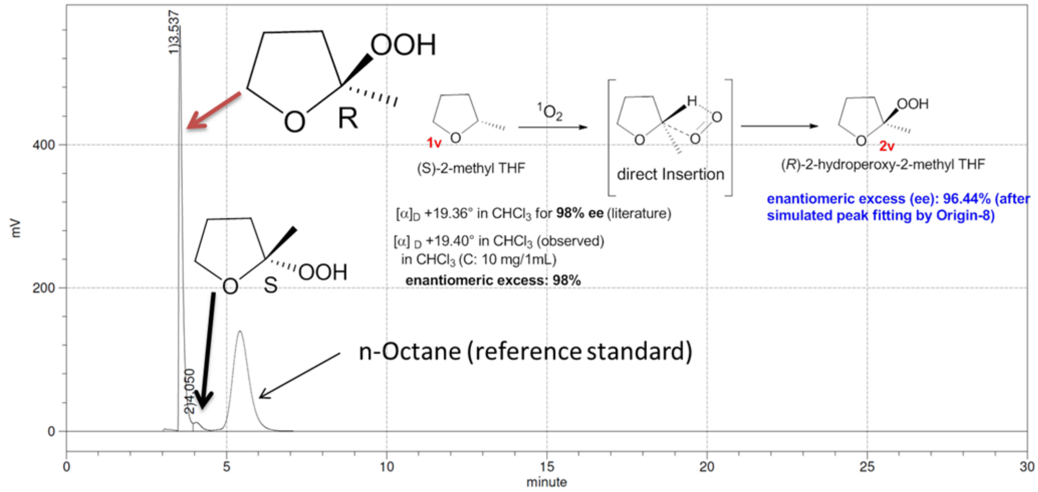

Figure S6. Chiral GC chromatograph of (R)-2-hydroperoxyl-2-methyl-THF (**2v).**

Q4) The authors conclude that the site selectivity of singlet oxygen and "electrophilic organometallic catalyst/hydrogen peroxide systems" are orthogonal in the discussion section of the manuscript. In fact, the singlet oxygen/electrophilic organometallic catalyst systems give the exact same site of oxidation when ethereal substrates are employed. Cyclic ethers are oxidized to the corresponding lactones and (-)-ambroxide is oxidized to (+)-sclareolide in both systems.

Our reply: Yes, we believe that singlet oxygen and electrophilic organometallic catalyst/H_2O_2 system belong to two different classifications, since singlet O_2 contains no metal ions and was consumed during the oxidation reaction, whereas organometallic catalyst contains a metal ion and was not incorporated into the final product. Although both systems act on the same alpha-ethereal C-H bond, they cannot be categorized into the same classification.

Reviewer #2 (Remarks to the Author):
If the claims of the authors can be properly substantiated, this would indeed be a paper worthy of publication in a high profile journal. However, I found that many alternative explanations could be easily provided to account for their data. In short, the authors need to provide the results of more control experiments to establish credence in their claims (suggestions are listed below).

Moreover, the authors' credibility is easily questioned based solely on the fact that they refer to and cite dated material. They need to do a better search of the literature on singlet oxygen that has been published in the last 30 years, and present arguments that demonstrate a command and understanding of this material.

Our reply: Thanks for the positive comments "If the claims of the authors can be properly substantiated, this would indeed be a paper worthy of publication in a high profile journal". As you can see in our replies to the 6 questions below, all questions/puzzles were well addressed and resolved.

If this reviewer finds any alternative explanations to our data, any mistakes or wrong description in our main text regarding the singlet oxygen literature work, please describe it (or them) explicitly so that we can provide our replies specifically to the questions.

Some specific comments:
Q1. It was good to see the authors recognize that THF (and other ethers) readily form hydroperoxides. However, it is imperative that they demonstrate the complete absence of impurities in their reactants. Ethers are notoriously "dirty".

Our reply: Yes, we are aware that ethers contains a lot of hydroperoxides once they are open-canned and exposed to air and room light for 6~12 months. All the ethers used in this study were freshly distillated so that no hydroperoxide is present in the starting substrates. We stated this experimental procedure in the main text of the original manuscript. For example, we have attached below selected ^{1}H NMR spectra of starting materials (after freshly distilled) and see, all starting materials ^{1}H NMR spectra (after purified) in supporting information (page: S69-S76) From the ^{1}H NMR spectrum of shown below, one can see that no any impurities, especially peroxide and lactone impurities, were present in our starting substrate THF. Even after 36 h reaction with 25 mol% KO_2 at 35 °C, no any other peroxide/lactones were present in the THF solution.

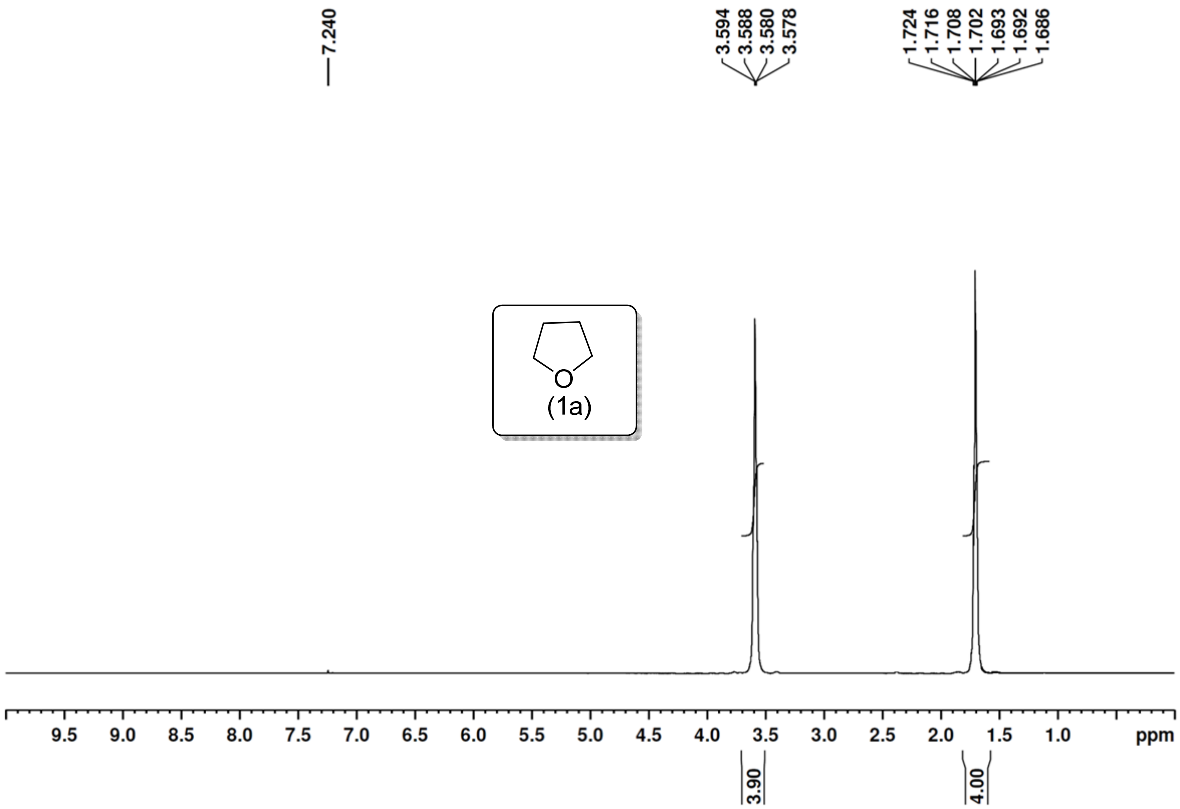
7.240
3.594
3.588
3.580
3.578
1.724
1.716
1.708
1.702
1.693
1.692
1.686
(1a)
9.5 9.0 8.5 8.0 7.5 7.0 6.5 6.0 5.5 5.0 4.5 4.0 3.5 3.0 2.5 2.0 1.5 1.0 ppm
3.90
4.00

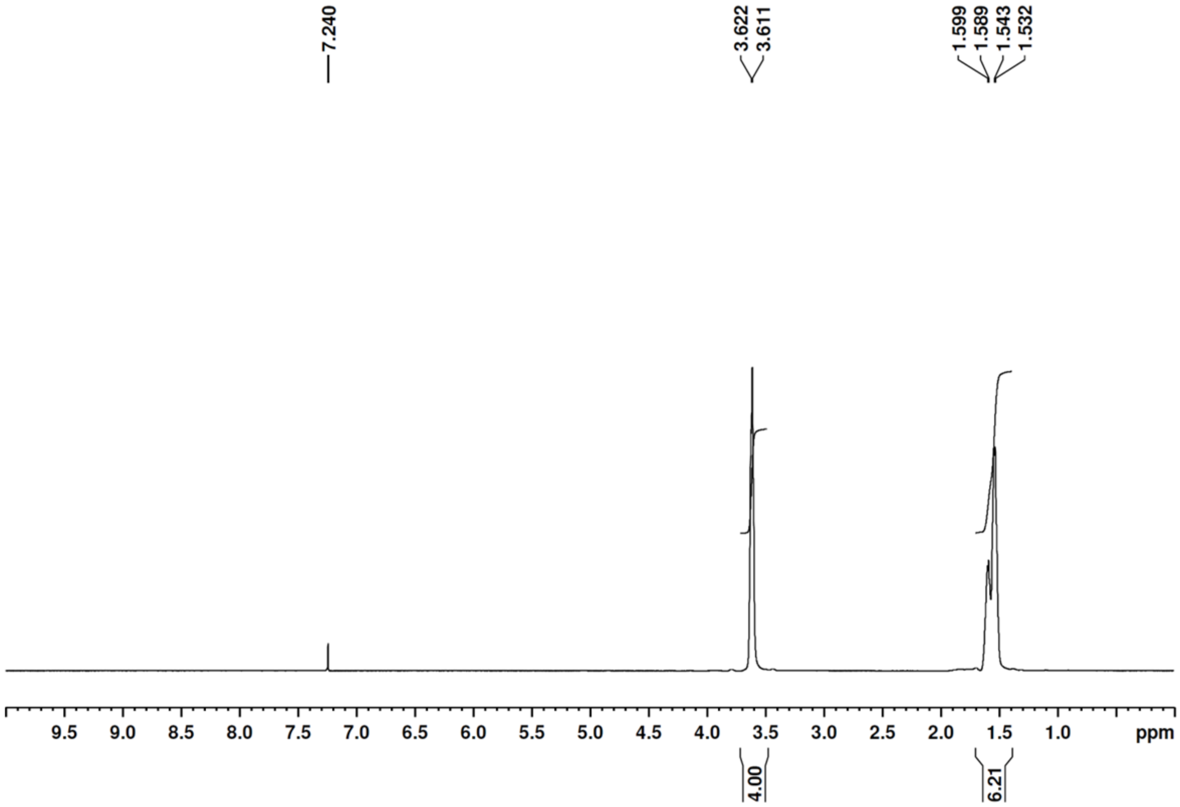
7.240
3.622
3.611
1.599
1.589
1.543
1.532
9.5 9.0 8.5 8.0 7.5 7.0 6.5 6.0 5.5 5.0 4.5 4.0 3.5 3.0 2.5 2.0 1.5 1.0 ppm
4.00
6.21

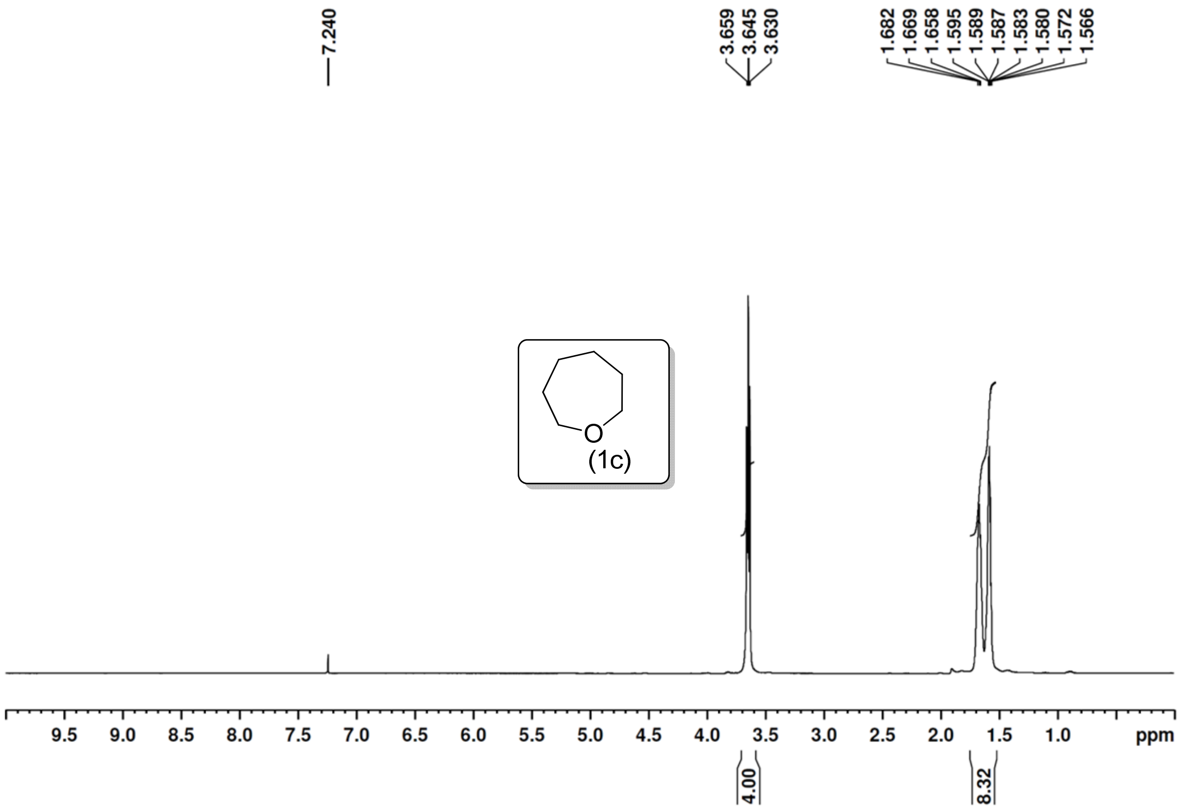
7.240
3.659
3.645
3.630
1.682
1.669
1.658
1.595
1.589
1.587
1.583
1.580
1.572
1.566
O
(1c)
9.5
9.0
8.5
8.0
7.5
7.0
6.5
6.0
5.5
5.0
4.5
4.0
3.5
3.0
2.5
2.0
1.5
1.0
ppm
4.00
8.32

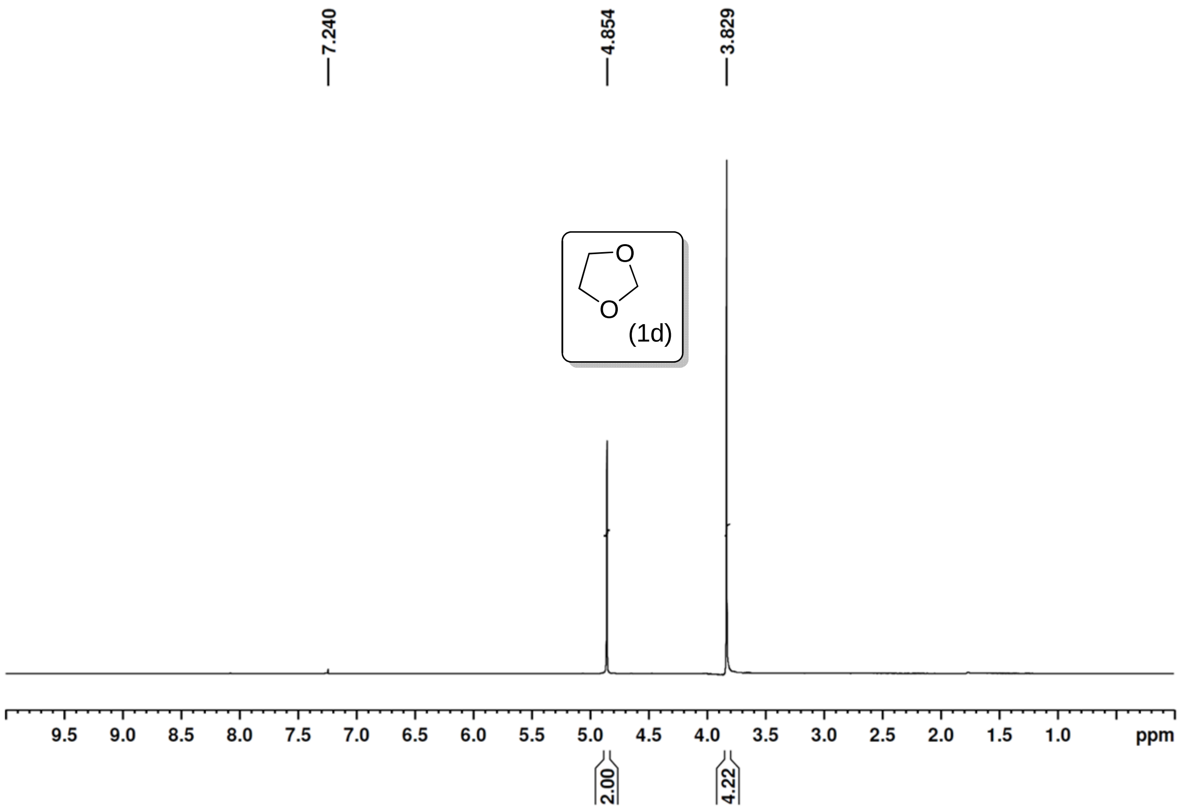
7.240
4.854
3.829
O
O
(1d)
9.5 9.0 8.5 8.0 7.5 7.0 6.5 6.0 5.5 5.0 4.5 4.0 3.5 3.0 2.5 2.0 1.5 1.0 ppm
2.00
4.22

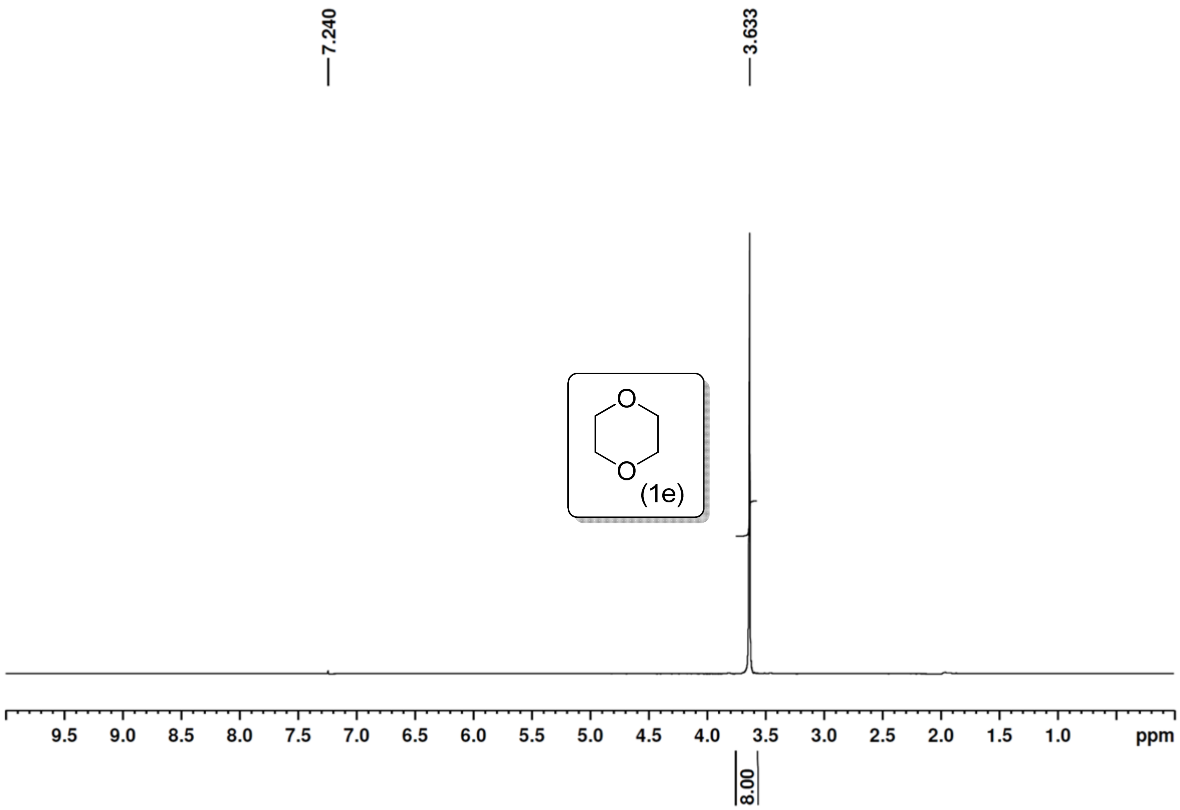
7.240
3.633
O
O
(1e)
9.5 9.0 8.5 8.0 7.5 7.0 6.5 6.0 5.5 5.0 4.5 4.0 3.5 3.0 2.5 2.0 1.5 1.0 ppm
8.00

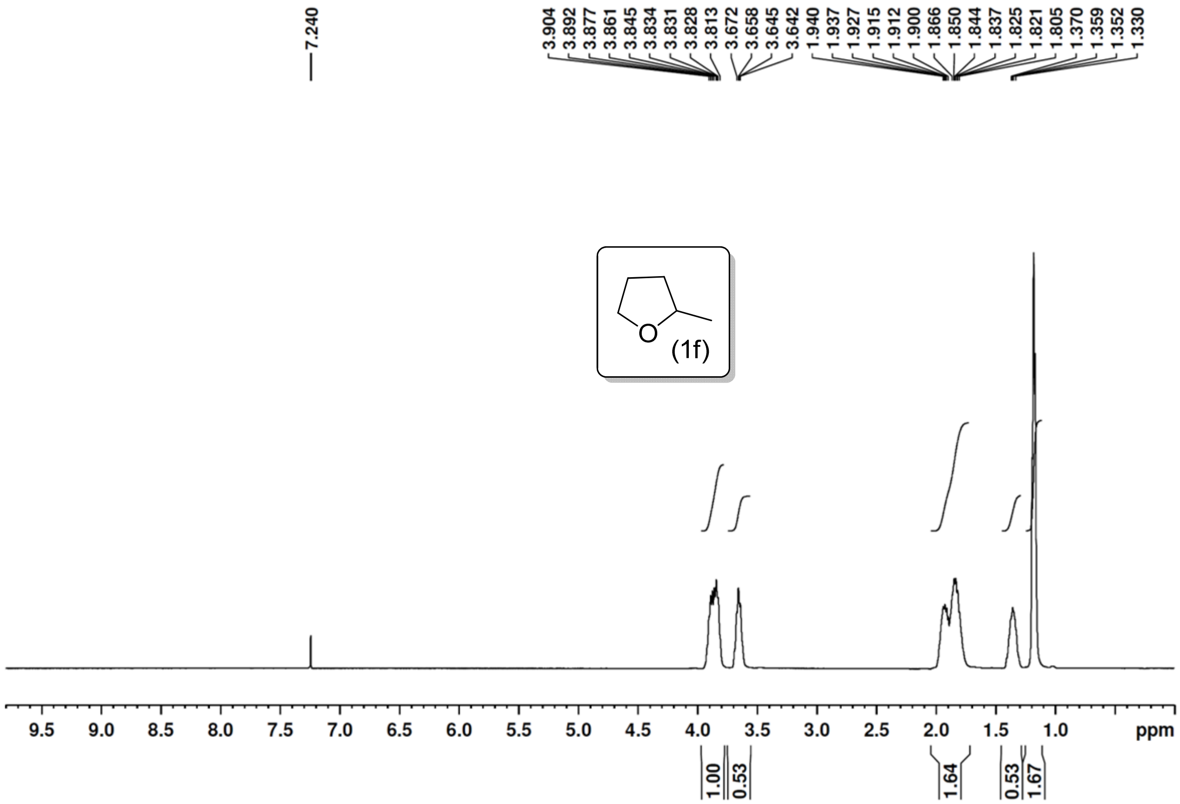

Q2. All of the systems investigated involve the presence of other molecules (in particular, sensitizers). The authors need to exclude the possibility that the singlet oxygen produced first reacts with the sensitizer which then, via a secondary process (e.g., decomposition of a sensitizer hydroperoxide), oxidizes the ether.

Our reply: Yes, there exists a possibility that singlet oxygen reacts with the photosensitizer to generate some reactive species, which then further oxidizes ether to generate hydroperoxide and lactones, albeit such a possibility is very slim since the concentration of a photosensitizer is usually 4~6 orders lower than THF in the system. Another more likely process is electron transfer from organic photosensitizer to generate superoxide ($O_2^{\cdot -}$), which might then further oxidize ether to hydroperoxides and lactones. To examine such a possibility, we mixed 25 mole percent of KO_2, a stable source of superoxide radical anion, with freshly distilled THF in the presence of O_2 for 36 h. However, no any trace amounts of either hydroperoxide or lactone was detected (see the ^{1}H NMR spectrum in Figure 1(e) attached below).

To exclude the possibility that the primary reaction intermediate(s) of singlet O_2 with photosensitizers further oxidizes ethers to generate hydroperoxide and lactones, we took the suggestion from this reviewer in Q6 to directly excite molecular oxygen to generate singlet O_2, and see whether hydroperoxide and/or lactones were formed. As shown in the ^{1}H NMR spectra of Figures 1(f) and (g) attached below, there are indeed some small

amounts (6~10%) of THF-hydroperoxide and THF-lactones being formed after 36 h irradiation by 100 W Hg lamp (900 mW/cm^2 at 765 nm) or by 780 nm laser (75 mW/cm^2, see also the data shown in Table 1 in our reply to Q5). The low yields of THF-hydroperoxide/lactone are understandable, since the quantum yield of singlet oxygen generation by direct excitation of molecular oxygen is very low, in the order of 10^{-5}~10^{-9}, depending the solvent and conditions (see, P. R. Ogilby, et al., J. Phys. Chem. B 2015, 119, 5422−5429). The data unambiguously demonstrate that singlet O_2 indeed can directly react with THF to generate oxidation products.

Previously, we reported that direct photo excitation of gold, silver and platinum metal nanoparticles can lead to sensitization of molecular oxygen and formation of singlet O_2 (see, *Angew. Chem. Int. Ed* .**2011**, *50*, 10640-10644), which has been confirmed by many other groups around the world. To avoid the use of organic photosensitizers, we also adopt silver nanoparticles to generate singlet oxygen in THF using the broad band output of a 100 W high pressure Hg lamp as the light source. As expected, formation of THF-hydroperoxide/lactone still can be observed (see the attached ^{1}H NMR spectrum shown in Figure 1(h) below).

All the above control experiments, in addition to 5 control experiments stated in our original manuscript, unambiguously demonstrate that singlet oxygen is in part, if not all, the active species which reacts with THF to generate THF-hydroperoxide and lactone.

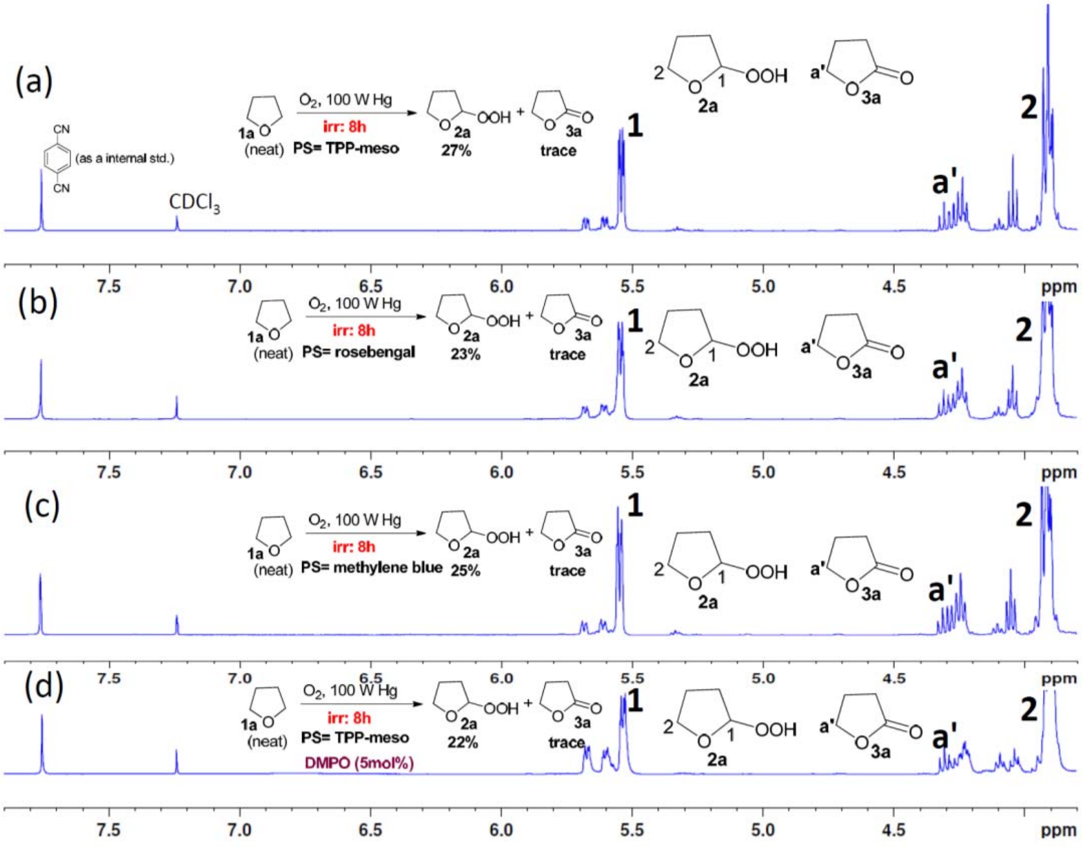

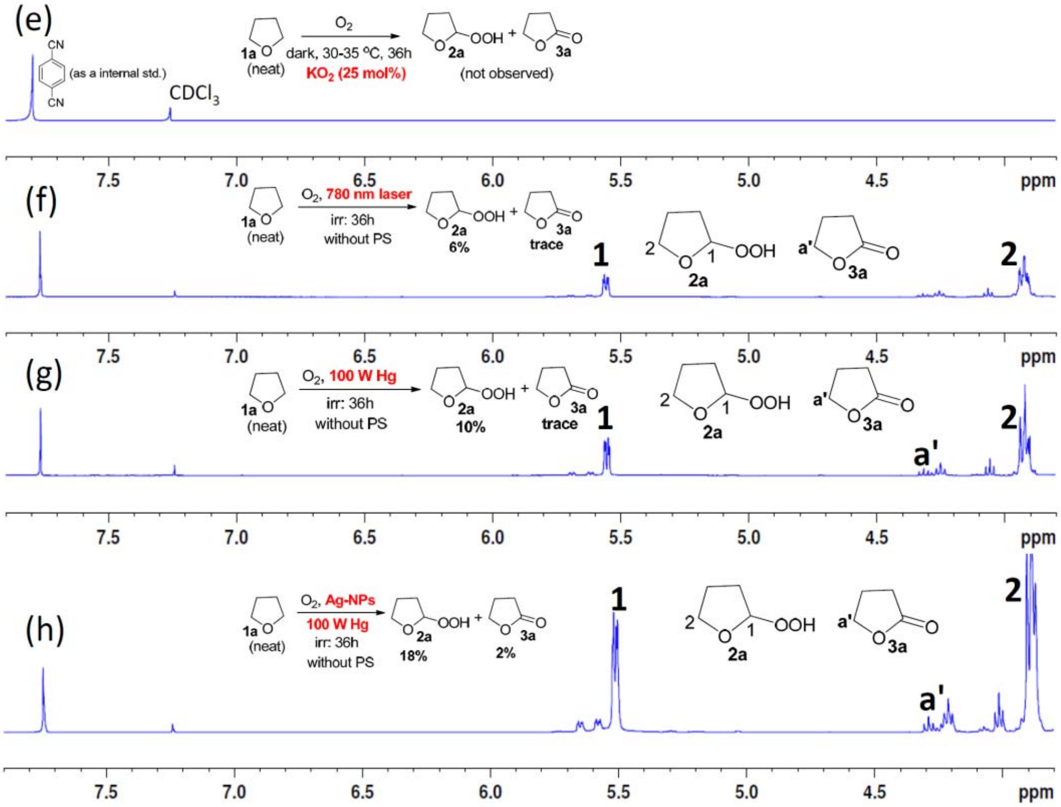

Figure 1. ^{1}H NMR spectra of THF reaction mixtures under different reaction conditions with singlet oxygen as labeled in the figures.

Q3. It was nice to see that the authors cited some early work on charge-transfer (CT) states. However, this direction needs to be more fully investigated. This includes CT states involving the sensitizers.

Our reply: Yes, as explained in our reply to Q2, there is such a possibility to generate superoxide via electron transfer from photo-excited organic photosensitizers to molecular oxygen. Whether or not superoxide is in part responsible for the formation of hydroperoxide and lactones should be examined. As shown in the attached ^{1}H NMR spectrum in Figure 1(e) in our reply to Q2, addition of 25 mole% KO_2, a stable source of superoxide, to THF in the presence of O_2 for 36 h in dark did not result in formation of any trace amounts of THF-hydroperoxide and lactone. Therefore, the participation of superoxide in reaction with THF to form oxidation products is unambiguously excluded. Overall, all our control experiments clearly demonstrated that it is the singlet oxygen which directly reacts with the alpha-ethereal C-H bond to form hydroperoxide and lactone. Such a reaction mechanism is also supported by a few other control experiments (such as, olefin competition reaction) as well as our CASSCF theoretical calculation which was described in the main text of the original manuscript.

Q4. With respect to the material on the top of page 5, I have no doubt that singlet oxygen is formed. However, the five points listed by the authors are consistent with many different, and more believable, mechanisms.

Our reply: We respectfully disagree with the comment "However, the five points listed by the authors are consistent with many different, and more believable, mechanisms". In the literature, it is well known that NaN_3 is a specific quencher for singlet O_2 (see, Luminescence. 2011; 26(6):685-8; P. R. Ogilby, et al., J. Phys. Chem. B 2015, 119, 5422−5429). In the presence of NaN_3, singlet O_2 was quenched and converted to superoxide. In our control experiment, the presence of NaN_3 dramatically decreases the amount of THF-hydroperoxide and lactone, which unambiguously indicates that singlet O_2 is in part, if not all, responsible for the formation of THF oxidation product. Our new control experiment using KO_2 as a source of superoxide does not result in formation of any THF oxidation product. Therefore, the possible involvement of superoxide is clearly excluded. We also generate singlet oxygen by a non-photochemical way via a well-known dark reaction of H_2O_2 with strong oxidants (such as, HOCl or $NaMoO_4$)(*19*) in THF, which also leads to formation of the products, **2a** and **3a**. This control experiment also unambiguously indicate that singlet O_2 can react with THF to generate corresponding hydroperoxide and lactone. In the case of olefin competition reaction, the presence of olefin, namely methylcyclohexene, leads to formation of well documented singlet O_2-ene reaction products (i.e., methylcyclohexene-hydroperoxides shown in the reaction equation S1 and Figure S4 in pages S56 & S57 in the supplementary materials, and also ref. 20) in accompany with the decrease in the yields of THF-hydroperoxide and lactone. This control experiment also unambiguously demonstrates that singlet O_2 is involved in the reaction with THF to generate THF-hydroperoxide and lactone. These 5 control experiments are very clear cut and are well accepted in the literature to be specific for singlet O_2. We did not see any other possible species or mechanisms being involved in these control experiments. If this reviewer thought there might be "many different, and more believable, mechanisms" being involved in our control experiments, please describe them explicitly so that we can further design experiments to prove or disprove these possible mechanisms.

In addition, as suggested by this reviewer in Q6, direct photo excitation of molecular oxygen to generate singlet oxygen in neat THF in the absence of any organic photosensitizers also leads to the formation of THF-hydroperoxide and lactone. Overall, so many different lines of evidences all ambiguously demonstrate that singlet O_2 can activate the alpha ethereal C-H bond of THF to generate oxidation products, possibly via direct C-H bond insertion, which was supported by our theoretical calculation as well as observation of chiral 1-methyl,1-hydroperoxyl THF (Figure 2 in the main text of the original manuscript).

Q5. Were the yields shown in Tables 1 and 2 independent of the sensitizer used? What happens if a radical trap is added to the solution?

Our reply: As shown in the Table 1 attached below, 3 different organic photosensitizers, namely, TPP-meso, rosebengal, and methylene blue, were used as photosensitizers for generation of singlet oxygen in THF under the same irradiation condition. The total yields of THF-hydroperoxide and lactone, are more or less similar, within 2% difference, indicating that the formation of THF oxidation products does not seem to be strongly affected by the choice of organic photosensitizers. Certainly, we are aware that light absorption and quantum yields of singlet O_2 sensitization of photosensitizers as well as the incident light intensities all play a role in affecting the final yields of THF oxidation products.

As suggested, we have also added a radical trapping reagent, namely, DMPO (5,5-Dimethyl-1-Pyrroline-N-Oxide), into the neat THF solution. The total yield of THF-hydroperoxide and lactone was only slightly affected by the presence of DMPO (see Figure 1(d) vs. 1(a) in reply to Q1 above and the entry 4 in the Table attached below), suggesting that very little (or no) amount of) THF-hydroperoxide/lactone was produced via a radical-associated pathway. Overall, our results unambiguously demonstrate that singlet O_2 is most probably the only one (or the dominant) species responsible for the formation of THF-hydroperoxide and lactone. Superoxide does not react with THF to give any products; and no (or negligible amount, if there is any) any radical species is involved in the reaction with THF to generate THF-hydroperoxide and lactone.

Table. Yields of THF-hydroperoxide and lactone using different organic photosensitizers and in the presence/absence of a radical trapping reagent, DMPO, as well as direct generation of singlet O_2 by direct photoexcitation of molecular oxygen by 780 nm laser light.

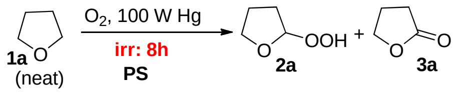

Entry	PS	Products (2a & 3a)		Yield[a] *%
1	TPP-meso	**2a (27%)**	**3a (trace)**	27
2	rosebengal	**2a (23%)**	**3a (trace)**	23
3	methylene blue	**2a (25%)**	**3a (trace)**	25
4	TPP-meso +<br>5 mol% DMPO	**2a (22%)**	**3a (trace)**	22
5	Ag-NPs (36 h)	**2a (18%)**	**3a (2%)**	20
6	absence of PS (36 h)<br>100 W Hg lamp<br>900 mW/cm2 at 765 nm	**2a (10%)**	**3a (trace)**	10
7	absence of PS (36 h)<br>irr.by 780 nm laser<br>75 mW/cm2	**2a (6%)**	**3a (trace)**	6

Q6. Here is another meaningful experiment that could be (should be) done. It has recently been shown that irradiation at 765 nm of a neat (i.e., sensitizer-free) solvent that contains oxygen will generate singlet oxygen. This is a consequence of pumping a transition in oxygen itself. In this way, one cleanly generates singlet oxygen, and singlet oxygen alone. If the authors produce singlet oxygen in this way, and still see the oxidation of their ethers, I will be more inclined to look deeper into their claims.
In conclusion, I regret to report that this paper is not ready to be published

Our reply: Yes, as suggested by this reviewer, we have done direct photo excitation of molecular oxygen to generate singlet oxygen in THF. As shown in the ^{1}H NMR spectra in our reply to Q2 (Figure 1(g) and 1(h) above), after a long time (36 h) irradiation by either 100 W high pressure Hg lamp (900 mW/cm^2 at 765 nm) or by 780 nm laser (75 mW/cm^2), indeed small amounts (5%) of THF-hydroperoxide and lactone were formed. The yields of THF-hydroperoxide and lactone are far less than those generated in the presence of organic photosensitizers at a much shorter (8 h) irradiation time. Such a low yield is reasonable and understandable, since the quantum yield of singlet O_2 formation by direct photo excitation of molecular oxygen is very low, in the order of 10^{-5}~10^{-9}, dependent on the solvent and conditions (see, P. R. Ogilby, et al., J. Phys. Chem. B 2015, 119, 5422−5429). This control experiment unambiguously illustrates that singlet O_2 can directly activate alpha-ethereal C-H bond to generate hydroperoxide and lactones. Our data clearly demonstrate that there is indeed some new singlet oxygen chemistry under the sun. We have added this new piece of data into the main text as another important supporting evidence for the direct chemical reaction of singlet oxygen with THF.

Reviewer #3 (Remarks to the Author):

This work describes the selective oxidation of ethers at the alpha position to hydroperoxides and esters and provides good evidence that the reaction is singlet oxygen mediated. To the best of my knowledge, this is a novel reaction of singlet oxygen (assuming the authors are correct) and it is therefore of broad general interest.
The English could use some work in the editing process, but is basically clear enough to be read and understood.

I think the paper should be accepted after some revision. Here are some points to consider. I do not think the introduction is very clear on what the authors think was anomalous about the singlet oxygen lifetime data in the literature and the general theory about vibrational deactivation in many solvents. I knew Chris Foote well, and I think I would have known if he thought THF was some weird case. Many lifetime discrepancies have been demonstrated to be impurity related because the impurities are so much more efficient quenchers of singlet oxygen than the main solvent. For example, Oliveros and Braun find a quenching rate constant of < 7 x 10^3 for methyl t-butyl ether (which is totally in line with these results), but note that lifetimes in that solvent vary with small amounts of alkene impurity. When I was active in singlet oxygen chemistry, impurity quenching was the broad conventional wisdom for the lifetime in several solvents. The authors need to be more clear about whether they think ethers break the traditional vibrational model or fit right in...and hit it again in the discussion.

Our reply: In the Oliveros and Braun's paper (*J. Photochem. Photobiol. A: Chem 1992, 68,343-351),*the authors observed that addition of different amounts of methyl tert-butyl ether (MTBE) into CH_3OD can shorten the lifetime of singlet O_2 in CD_3OD with a quenching rate of 7 x 10^3 $M^{-1}s^{-1}$. The authors also found that the olefinic impurity in MTBE, i.e., 2-methyl-2-butene (OL, a precursor for making MTBE), contributes significantly to the whole quenching process. By using Stern-Volmer plot as a function of the OL concentration, the authors were able to obtain a quenching rate of 9.4 x 10^5 $M^{-1}s^{-1}$ for OL, and a quenching rate of 2900 $M^{-1}s^{-1}$ for **pure** MTBE. Overall, the authors observed that in addition to the olefinic impurity quenching pathway, pure MTBE itself can also deactivate the singlet O_2 with a rate of 2.9 x 10^3 $M^{-1}s^{-1}$. Since the authors wrote that "the ethers are chemically inert to $^1O_2$, ….", they attributed the bimolecular quenching rate of 2.9 x 10^3 $M^{-1}s^{-1}$ to be **physical quenching** of singlet O_2 by pure MTBE, which is corresponding to a lifetime of 41 μs for $^1O_2$ in pure MTBE (8.4 M, density= 740 g/L). As compared to the 900 μs lifetime in CCl_4, the lifetime of 41 μs in pure MTBE is 220 times shorter. The authors, however, did not provide explanation to the commonly observed very short lifetimes of singlet O_2 in many other solvents, including non-ethereal solvents, where olefins were not used as precursors for making solvents and thus there is absence of olefinic impurities in solvents. Overall, olefinic impurity-mediated quenching is only part of overall quenching processes, and cannot be extended to many other non-ethereal solvent systems to explain the large variation of singlet O_2 lifetimes as well as solvent kinetic isotope effects in various types of solvents. Therefore, the significantly shorter lifetimes of singlet O_2 in various types of solvents is still an

unresolved mystery in the literature. Notice that the "physical quenching" rate of 2.9 x 10^3 $M^{-1}s^{-1}$ obtained by Oliveros and Braun is in the same order as what we obtained the chemical quenching rate of 3.8 x 10^3 $M^{-1}s^{-1}$ for freshly distilled pure THF toward singlet O_2. We have added the above discussion to both introduction section of our revised manuscript.

Q1. I was surprised the authors did not report an experiment using d_8-THF, which (although expensive) is commercially available.

Our reply: Yes, we did use d_8-THF to react with singlet oxygen. Under the same reaction condition, the yield (8%) of d_8-THF hydroperoxide is significant lower than that (35%) of the H_8-THF hydroperoxide, which is due to the stronger C-D bond than the C-H bond and is consistent with the expected kinetic isotope effect. We have described the experimental conditions in the supplementary information in page S3, and added the ^{1}D-NMR spectra of d_8-THF data to the supplementary materials in page S45~S46.

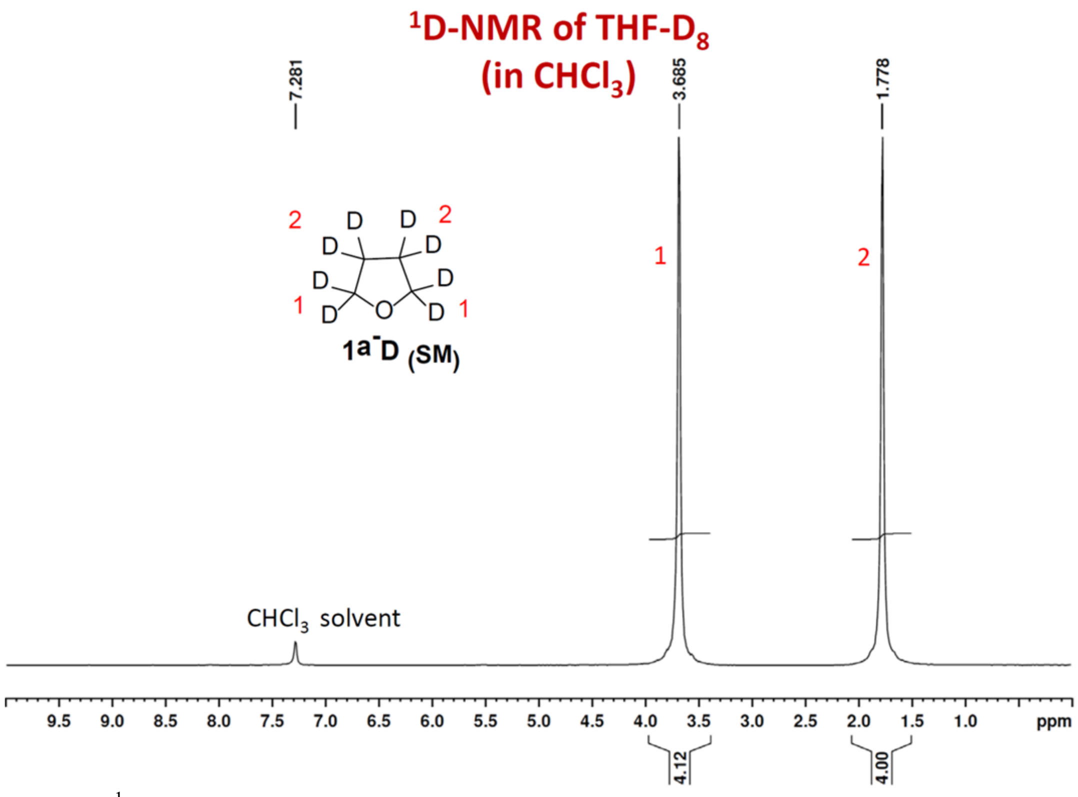

Figure. ^{1}D-NMR of d_8-THF in $CHCl_3$ (before irradiation)

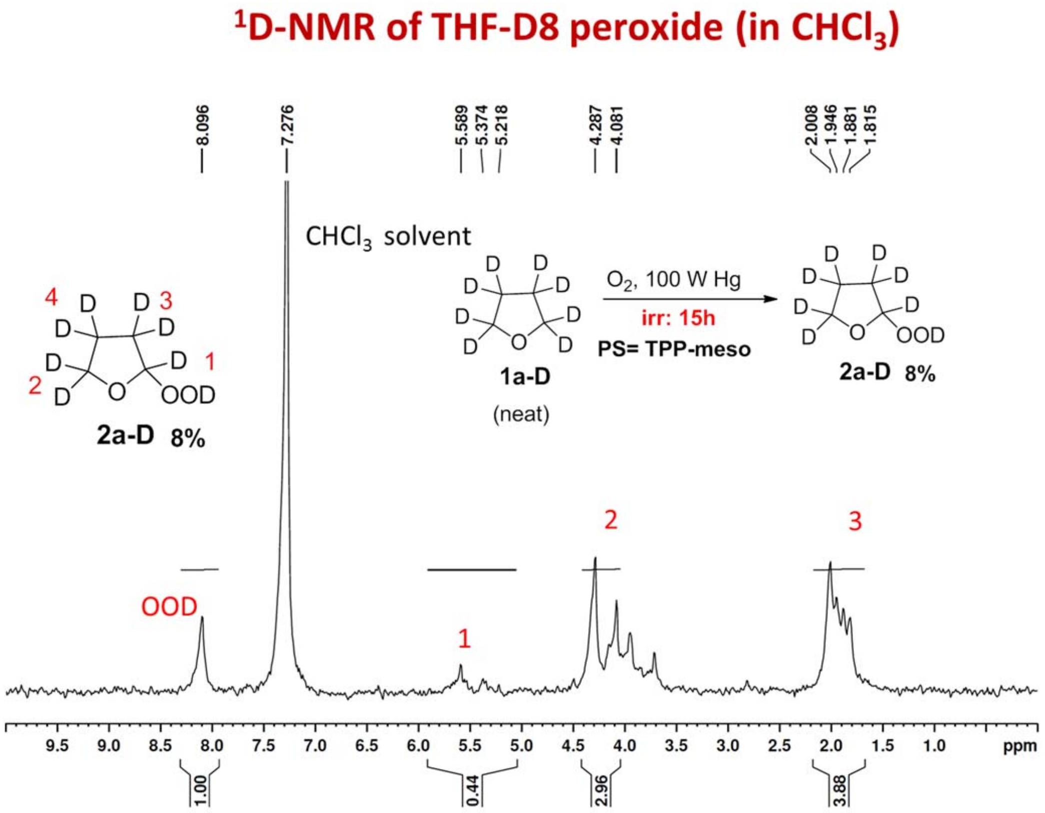

Figure. [1]D-NMR of d_8-THF hydroperoxide in $CHCl_3$

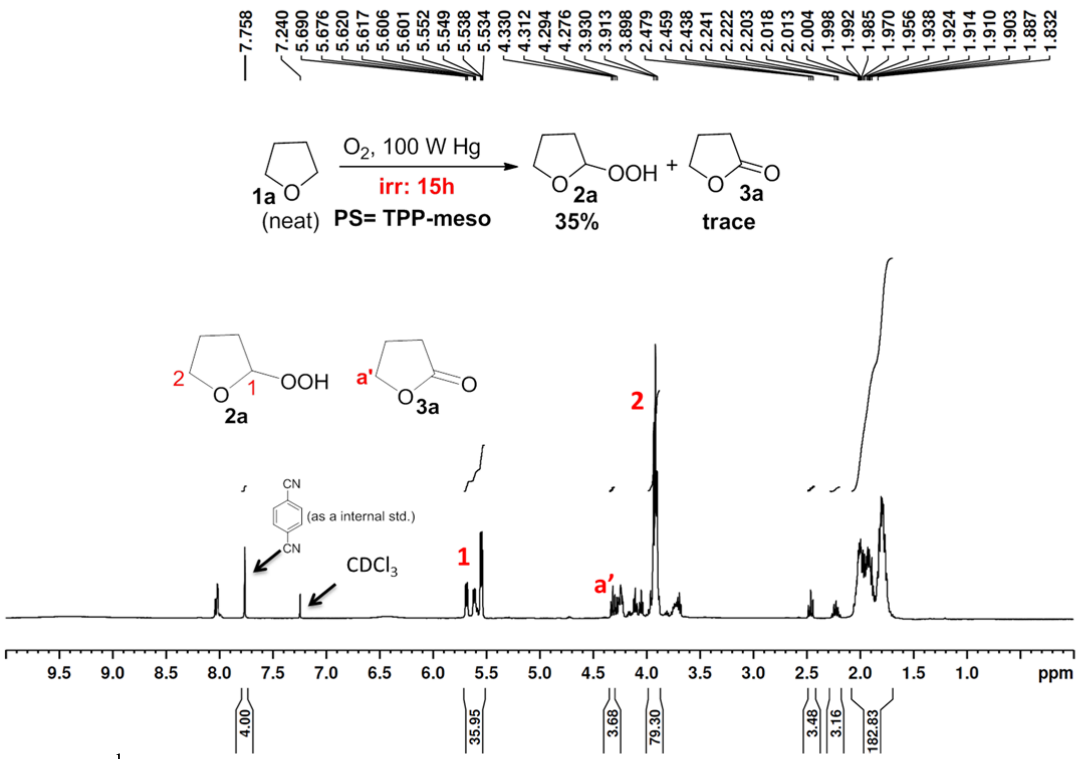

Figure. ^{1}H-NMR of THF-hydroperoxide (**2a**) and THF-lactone (**3a**) from the reaction of neat THF with singlet oxygen (15 hr irradiation)

Q2. The reported rate constant should only be given to 2 places, especially given that the reference rate is only given to two. and an "about tilde" is used.

Our reply: Thanks for the suggestion. We have changed the rate constant of singlet O_2 reaction with THF to be two digits, i.e., 3.8 x 10^3 $M^{-1}s^{-1}$ without the about tilde.

Q3. In my version of Table 2, there are some stray remarks and figures. This could be a pdf artifact, but please check.

Our reply: In the Table 2, the note on yield was mistakenly labeled as "[c]", which has been corrected as "[a]". For the product **3p**, we added the single crystal x-ray structure within the Table nearby the chemical draw structure **3p** to show that the chemical structure of **3p** was confirmed by single crystal x-ray measurement. If this is the one this reviewer mentioned, it is not a pdf artifact. In the Table legend, we did add two sentences to describe the experimental condition.

Q4. Why these two particular Lewis Acids? Are these optimized or just two random choices?

Our reply: As shown in the Table 2 attached below, we in fact tried many different Lewis acids, including $AlCl_3$, Et_3B, $(EtO)_3B$, $FeCl_3$, $SnCl_2$, and γ-Al_2O_3. All Lewis acids can enhance the total yields of THF-hydroperoxide and lactone, probably via coordination to the ethereal oxygen atom and subsequently weakening the alpha-ethereal C-H bond to favor direct C-H bond insertion by singlet O_2. Different Lewis acids have different extent of ability to help convert hydroperoxides to corresponding lactones.

Table 2. Effects of different Lewis acids on the yields of THF-hydroperoxide and lactone.

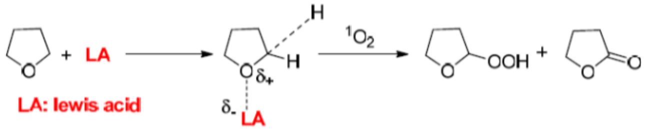

Entry	Lewis acids	Products (2 & 3)		Yield[b] %
1	Absence of lewis acid	OOH 2a (27%)	+ 3a (trace)	27
2	$AlCl_3$	2a (5%)	3a (40%)	45
3	Et_3B	2a (20%)	3a (23%)	43
4	$(EtO)_3B$	2a (20%)	3a (10%)	30
5	$FeCl_3$	2a (5%%)	3a (30%)	35
6	$SnCl_2$	2a (5%%)	3a (32%)	37
7	γAl_2O_3	2a (10%%)	3a (35%)	45

Q5. Is there good evidence that the complexation of singlet oxygen by the Lewis Acid is not involved? The theoretical modeling does not include any Lewis Acid.

Our reply: Yes, in the theoretical calculation part, we did not consider the involvement of the Lewis acid. The reason for this is simply because the active space in the originally computed system is 14-electron-in-10-orbital, which is so large that we cannot afford to consider the other Lewis acid. For instance, if our calculations consider the Lewis acid (such as $AlCl_3$), then its active space will be 36-electron-in-24-orbital, which makes the theoretical computations unlikely. Moreover, as seen in Figure 3, even without considering the Lewis acid, the barrier height from **2a** to **3a** was computed to be small (19 kcal/mol). One may thus imagine that the involvement of the Lewis acid (such as $AlCl_3$) should greatly reduce the activation energy (much less than 19 kcal/mol), which can make such chemical reactions quite efficient.

In the literature, it was never reported that singlet oxygen can form complexes with Lewis acids. In addition, singlet oxygen is an oxidant, electron deficient and short-lived. All these factors do not favor the complexation of singlet oxygen with electron-deficient Lewis acid. Therefore, it is quite unlikely that short-lived singlet oxygen is able to complete with high concentration of electron donating THF (12.3 M in neat THF) and form complexes with Lewis acid.

Q6. The authors need to be much clearer in the main text that the molar optical rotations of methyl-THF and the hydroperoxide are known values, because that makes the retention of configuration argument much stronger. (This is very good evidence for a one step mechanism.)

Our reply: Yes, we agree that the measurement of optical rotation value, without comparison with molar optical rotation value, is not conclusive to prove 100% conservation of stereo-configuration. The molar optical rotational angle value of the product, i.e., chiral 2-hydroperoxyl-2-methyl-THF, however, is not available in the literature. To resolve the problem, we have to determine the enantiomeric excess values for both the starting chiral (S)-2-methyl-THF and the product (R)-2-hydroperoxyl-2-methyl-THF. We followed a literature procedure (ref. 24) to prepare the chiral (S)-2-methyl-THF. The optical rotation value of our as-synthesized chiral (S)-2-methyl-THF is $[a]_D$ +19.40° in $CHCl_3$, observed, 10 mg/1 mL, which is nearly the same as that ($[a]_D$ +19.36° in $CHCl_3$, 10 mg/mL) of literature reported value for 98% enatiomeric excessed (S)-2-methyl-THF (ref. 23). To determine the enantiomeric excess of the (R)-2-hydroperoxyl-2-methyl-THF product, we followed a literature procedure (*J. Am. Chem. SOC.* **1982,** *104*,1513-1580) to prepare a chiral nickel(II) bis[(1R)-3-(hepta-fluorobutyryl)camphorates] complex-based GC column for separation of the R and S forms of racemic 2-hydroperoxyl-2-methyl-THF (**2f**) products. As shown in the GC spectra below, racemic 2-hydroperoxyl-2-methyl-THF can be well separated by the Ni complex-based chiral column. Details of procedure for preparation

of chiral Ni-complex, coating of column, and instrumental analysis condition were discussed in revised supporting information (page no: S15 and S59). The GC spectrum of the hydroperoxides obtained from reaction of (S)-2-methyl THF (98% ee) with singlet oxygen shows the dominant presence of (R)-2-hydroperoxyl-2-methyl-THF (96.4% enantiomeric excess) with minor amount of (S)-2-hydroperoxyl-2-methyl-THF, which is very close to the enatiomeric excess (98% ee) value of the starting substrate. Therefore, the data strongly support our conclusion that singlet oxygen directly inserts into the alpha ethereal C-H bond in a one-step manner, leading to the conservation of stereo-configuration of the starting substrate. We have incorporated the above data to supplementary information as Figure S4~S7.

Scheme S1. Enatiomeric excess values of the starting (S)-2-methyl THF and its reaction products with singlet oxygen.

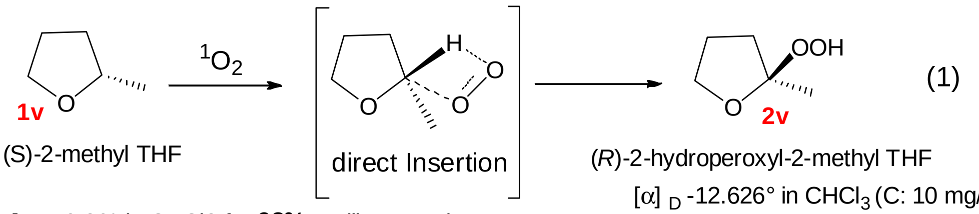

$[\alpha]_D$ +19.36° in CHCl3 for **98% ee** (literature)

$[\alpha]_D$ +19.40° in $CHCl_3$ (observed)
in $CHCl_3$ (C: 10 mg/mL)

enantiomeric excess (ee): 98% (literature)

enantiomeric excess (ee): 98% (observed)

$[\alpha]_D$ -12.626° in $CHCl_3$ (C: 10 mg/mL)

enantiomeric excess (ee): 96.44% after simulated peak fitting by Origin-8

SISC(32) FOR WINDOWS TEST REPORT

Racemic mix.

File:D:\SISC32E\WORK0001\2016-10-21-exp-100.CHR
Injection Date:10-22-2016 Time:05:06:36

No.	R.T. (min.)	Area (uv*sec)	Area%	Height (mv)	Height%	Conc.	Conc.%	Conc. Unit	Base Code	Theoretical Plates	Peak Name
1	3.596	5434321	51.2962	669.4473	69.3064	0.0000	0.0000		PV	0	
2	4.150	5159672	48.7038	296.4771	30.6936	0.0000	0.0000		VV	0	
	Total	10593993		965.924		0.0000	0.0000				

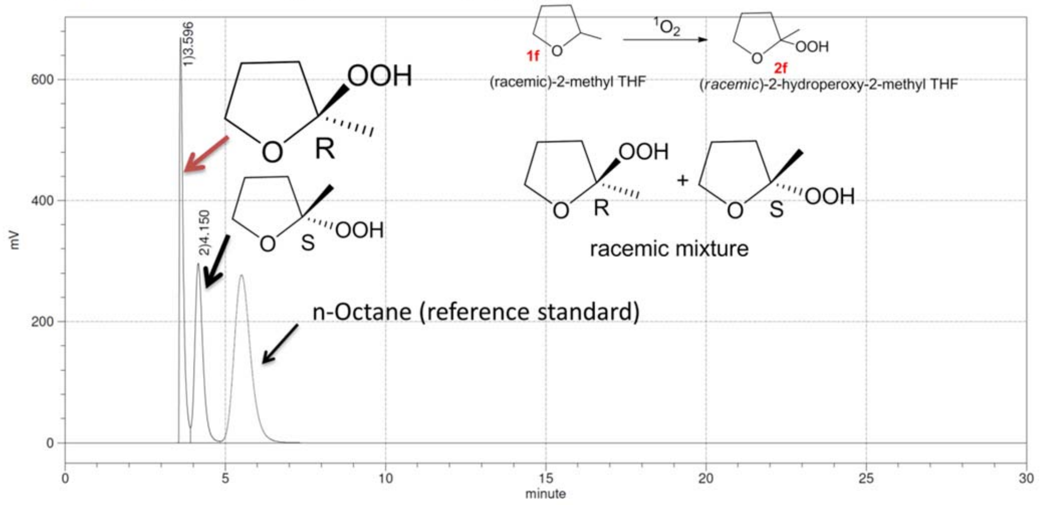

Figure S5. Chiral GC chromatograph of racemic -2-hydroperoxyl-2-methyl-THF (**2f**).

SISC(32) FOR WINDOWS TEST REPORT

Chiral Product

File:D:\SISC32E\WORK0001\2016-10-21-exp-50.CHR
Injection Date:10-22-2016 Time:01:28:38

No.	R.T. (min.)	Area (uv*sec)	Area%	Height (mv)	Height%	Conc.	Conc.%	Conc. Unit	Base Code	Theoretical Plates	Peak Name
1	3.537	4637049	95.5855	566.6483	97.8751	0.0000	0.0000		VV	0	
2	4.050	214157	4.4145	12.3021	2.1249	0.0000	0.0000		VV	0	
	Total	4851206		578.950		0.0000	0.0000				

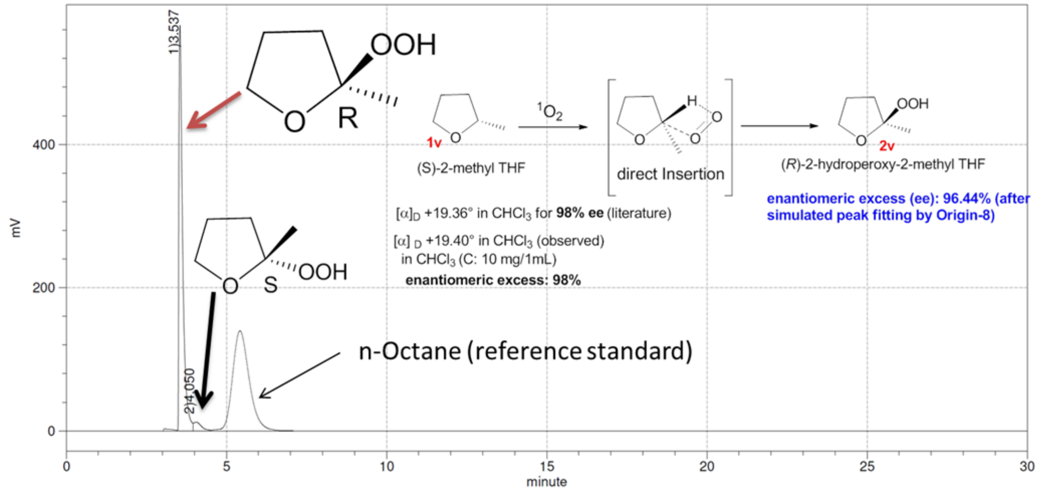

Figure S6. Chiral GC chromatograph of (R)-2-hydroperoxyl-2-methyl-THF (**2v**).

Q7. Figure 3 should make clear what the computational method was. A couple different basis sets are noted in the text. I assume that they are the MP2-CASSCF, rather than just CASSCF.

Our reply: The computational method used in this work is at the CAS-MP2(20,14)/6-311G(p)//CAS(20,14)/6-31G(p) level of theory. We apologize for the confusion we made.

Q8. The discussion is really unclear. I don't understand at all what is meant by "the selectivity of singlet O_2 is most probably governed by the singlet spin multiplicity". By definition, singlet oxygen is a singlet, so there must be something more profound in here that I am missing. I think the authors would do well to give singlet oxygen chemists who have missed this reaction for decades a better physical interpretation of what they think is going on.

Our reply: What we mean "the selectivity of singlet O_2 is most probably governed by the singlet spin multiplicity" is that direct C-H bond insertion reaction is only possible or allowed for reactive species having singlet spin multiplicity, but not possible for those with triplet spin multiplicity. In the literature, it was well documented that singlet carbene undergoes direct one step C-H insertion reaction, whereas triplet carbene undergoes a three-step reaction, i.e., hydrogen atom abstraction, intersystem crossing of a triplet radical pair to singlet radical pair, and then singlet radical pair coupling to form a covalent bond (see, Chem. Rev. 2000, 100, 39−91; *J. Am. Chem. Sot.* **1984,** *106,* 283-287). Reactive species with triplet spin multiplicity cannot proceed with one step direct C-H bond insertion reaction, since all covalent bond has a singlet ground state character in nature. Likewise, singlet oxygen undergoes one step, direct C-H bond insertion to alpha-ethereal C-H bond, which is dominantly controlled by the spin multiplicity of the "singlet O_2". We have revised the above the sentence to be "the selectivity of singlet O_2 is most probably governed by its spin multiplicity as well as the C-H bond strength of the alpha-ethereal C-H bond" in the revised manuscript.

Q9. Finally, the authors quote a rate constant for "chemical reaction rate" of THF with singlet oxygen as 3.8 x 10^3. They should be very, very explicit about "chemical reaction" vs "deactivation". A number of compounds physically deactivate singlet oxygen at a much higher rate than they "net" react with it. In other words the chemical efficiency of the quenching event is pretty low. The authors seem to be saying that is not the case here, in that they are saying that the "chemical reaction rate" is compatible with the lifetime in neat solvent. That, of course, implies a quantum yield of near 1 (after formation of singlet oxygen). That may be exactly what they mean, but it would be all the more remarkable that this reaction was missed for so long if it is the case. An explicit

statement to that effect or measurement of the chemical quantum yield is definitely reasonable to expect.

Our reply: Yes, this reviewer is correct that we are trying to report that chemical reaction of singlet oxygen with solvent molecules, rather than other physical deactivation processes, is the main deactivation channel responsible for the short lifetime (20 μs) of singlet O_2 in THF, as compared to the very long lifetime (900 μs) of singlet O_2 in CCl_4. The chemical quantum yield of singlet O_2 in THF, by definition, can be obtained via two methods: (a) the ratio of "moles of THF-singlet oxygen reaction products" vs. "the moles of singlet oxygen formed"; or (b) the observed apparent chemical reaction rate (k_1[THF]) of singlet oxygen with THF divided by the total rates (k_0 + k_1[THF]) of all physical (k_0) and chemical (k_1[THF]) deactivation channels (see the chemical equation shown below).

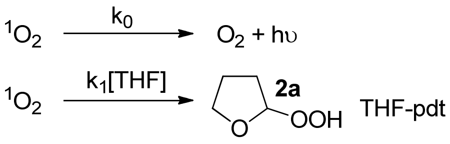

(S2a)

(S2b)

In neat THF

$$k_{Total} = k_0 + k_1[THF]$$

$$\text{Chemical quantum yield,}\Phi_{THF\text{-}pdt} = \frac{k_1[THF]}{k_0 + k_1[THF]} \qquad (3)$$

In CCl_4, the physical deactivation rate, k_0 value, is 1/(900 μs)=1.1 x 10^3 s^{-1}. In neat THF, the k_0 value is assumed to be (13/4= 3.25) times faster than that for CCl_4, since THF has 13 (C-H, C-C or C-O) single bonds as compared to 4 C-Cl single bonds for CCl_4. To the first approximation, this assumption is valid since solvent nuclear vibrational motion is the main exit channel responsible for the physical deactivation (via electronic-vibrational coupling) of singlet O_2. Therefore, the k_0 value for THF is tentatively assumed to be approximately 1.1 x 1.1 x 10^3 s^{-1} x 3.25= 3.6 x 10^3 s^{-1}. From the competition reaction with 1-methylcyclohexene, we obtained the chemical reaction rate of singlet O_2 with THF to be 3.8 x 10^3 $M^{-1}s^{-1}$. Neat THF has a THF concentration of 12.35 M. Therefore, the chemical quantum yield of product formation for singlet O_2 chemical reaction with THF can be obtained by the equation (3):

$$\Phi_{THF,pdts} = k_1[THF]/ (k_0 + k_1[THF])$$

$$= (3.8 \text{ x } 10^3 \text{ M}^{-1}\text{s}^{-1} \text{ x } 12.35 \text{ M})/ (3.6 \text{ x } 10^3 \text{ s}^{-1} + 3.8 \text{ x } 10^3 \text{ M}^{-1}\text{s}^{-1} \text{ x } 12.35 \text{ M})$$

$$= 0.93$$

The physical deactivation rate of singlet O_2 in THF adopted in the above calculation may not be accurate. Nevertheless, the physical deactivation rate is at least one order slower than the chemical reaction rate of singlet O_2 in THF. Small variation in the physical

deactivation rate will not change significantly the chemical quantum yield value (here, 0.93) of singlet O_2 in THF.

We have explicitly incorporated the above calculation and the chemical quantum yield value into the main text of revised manuscript. As per request by this reviewer, we have also explicitly stated in the revised manuscript that chemical reaction of singlet O_2 with THF solvent molecules is the dominant deactivation channel responsible for the short lifetime (20 μs) of singlet O_2 in THF, as compared to the very long lifetime (900 μs) of singlet O_2 in CCl_4. Such a chemical deactivation channel was never considered or recognized before in the past 35 years to account for short lifetimes of singlet oxygen in various solvents.

Reviewer #2 (Remarks to the Author):

I regret to report that the authors' revised manuscript and rebuttal have not convinced me that this work is ready to be published (in Nature Communications or any other journal).

Rather, the documents submitted this second time around strongly suggest to me that the authors still make some rather exorbitant claims that are not substantiated.

Listed below are selected points and examples that contributed to my opinion.

1. In my first review, I suggested that the authors make singlet oxygen using an approach that does not involve the addition of other molecules to the ether solution (e.g., sensitizers, H2O2, HOCl). I suggested they exploit the fact that singlet oxygen can be made upon direct irradiation of oxygen at 765 nm, and I am pleased to see that the authors took my advice in this regard. However, as best as I can tell from the information provided, rather than providing data to support their claims, the experiments performed by the authors support my contention that something else is happening in this system. Specifically, the authors indicate that, to directly excite oxygen, they irradiated the solution at 780 nm. However, as can be seen in the pertinent paper on this subject (their reference 21), oxygen does not absorb at this wavelength. Thus, unless they used a laser with a very broad spectral output (not indicated in the paper), something else must be absorbing the light that, in turn, triggers the reactions they observe. In short, they should see a pronounced difference in the reactivity of the ethers upon irradiation at 780 nm and 765 nm, respectively.

2. To make global conclusive statements throughout the manuscript about how their data from selected ethers solve the "35-year old mystery" regarding the effect of solvent on the singlet oxygen lifetime is not justified. Furthermore, to support such statements with a vague and unsubstantiated footnote (their reference 31) about experiments with other solvents ("data not shown") is not acceptable. With respect to the latter, I am particularly interested in seeing what the authors claim is happening in water.

3. If the authors choose to go beyond the "simple" report that singlet oxygen reacts with ethers and, instead, make global statements about the mechanism(s) for the general effect of solvent on the singlet oxygen lifetime, then a more systematic and detailed study needs to be presented. In such a work, the authors would need to properly address the existing work they claim is wrong, and show exactly why they think that work is incorrect. In such studies, it is indeed generally assumed that the singlet oxygen lifetime is determined by physical rather than reactive quenching. Thus, for the appropriate proof of their claims, the authors would need to identify the solvents that do not

behave according to a given model of physical deactivation, and show that everything fits properly when the given lifetime is adjusted for the observed component of reactive quenching.

4. As stated in my first report, I do not doubt that the authors are making singlet oxygen in their sensitized experiments. What they fail to do is show, in a convincing way, that their chemistry is the result of a direct reaction between singlet oxygen and the ether. In short, they still have not properly excluded other reaction possibilities. Again, as indicated in my first report, I find it most interesting that the authors' case in built upon a solvent system (ethers) that is notoriously "dirty" and that could support a host of reactions (e.g., free radical and electron transfer). The other solvent systems that they cite in their defense (reference 31, "data not shown"), likewise are notorious for supporting a host of reactions.

Reviewer #3 (Remarks to the Author):

This manuscript is much improved from the earlier version and is probably suitable for publication after a few more minor revisions. I think it is an excellent example of the peer-review process working because it is so much better than before. The addition of several control experiments and the chiral chromatography are big plusses.

The major flaw in the paper is that some of the language in it feels like a stretch to the knowledgable chemist involved in singlet oxygen and other physical organic chemistry. I feel obliged to be fussy on such points for a paper submitted to Nature.

For example, the authors still maintain in the introduction language that seems to equate the further oxidation of this ethereal carbon to oxidation of "unactivated" methane or internal methylenes. This is a pre-activated carbon, one that is easier to oxidized by several mechanisms (apparently including this new one) than others. If I were writing this paper, I would completely delete all discussion of CH activation and focus on the singlet oxygen novelty, as the authors do in their extensive "response to authors" section. The abstract and introduction are still misleading

My other major complaint is that the reported yields are so arbitrary. I assume that RSM stands for "remaining starting material", but the authors make no attempt to actually maximize yields, while simultaneously talking about their synthetic utility. If the vast majority of material is starting material plus products after 12 hours, why haven't the authors (in at least a few cases) actually taken the

reaction out to conclusion, either by increasing the light intensity or by extending the time of photolysis? These “moderate yields” are of no particular meaning if they are only moderate because they stopped the reaction before starting material was consumed or other secondary reactions started happening. You kind of have to choose: are you going to focus on the singlet oxygen novelty, or are you ALSO going to talk about the synthetic utility. If the latter, you need to at least make an effort to optimize a few yields.

The discussion of hydroperoxide vs lactone is still not satisfactory. I don’t even see a single mention of the possibility/probability that all of the singlet oxygen chemistry generates hydroperoxide, and the dehydration to lactone is simply catalyzed by the Lewis Acid. The computations can’t really address this at all, since there are such dramatic effects of charge separation in gas phase calculations…and to do anything of “chemical accuracy” would become enormously expensive on top of the current MP2-CAS computations.

Another silly point to continue to emphasize (because it is a straw man) is that the singlet oxygen system is different than hydroperoxide and organometallic catalysts. I fail to see the point.

A mention of two control experiments outlined in the response (KO2 and spin trapping) should be made in the main text, with reference to details in the supporting material.

The authors over-react to the olefinic quenching in MTBE, in the sense that olefins are not the only thing that can act as “impurity quenchers”. For example, in THF, water will affect the lifetime of singlet oxygen if it gets to high enough concentration. (This may make the full conversion of the ethers to product a little more difficult!)

It should be made clear relative to what the yelds are being measured earlier in the first Results paragraph. Eventually, we figure out that they mean relative to complete conversion, but that isn’t obvious at first and is confusing.

In the first paragraph of page 8, “Interestingly” is misused. According to the previously reported results, that should be “As expected,” or “In line with the model compounds,”.

In the discussion of the stereochemistry on page 9, the authors state “The chiral [product] has the same steric configuration as, but and opposite chirality from (due to onversion of light C-H to heavy OOH group), the starting substrate…” It would be less clumsy and convey just the same information to say that “the oxidation of CH to COOH proceeds with at least nearly complete retention.”

A conical intersection cannot be an intermediate (which is by definition a thermodynamic minimum). Please clarify. Also, please note the irrelevance of the computations to a normal acid-catalyzed dehydration mechanism.

The discussion of "site selectivity of singlet O2 [being] most probably governed by its spin multiplicity" still makes no sense. I understand that they are saying that ground state triplet oxygen and singlet oxygen are different, but this sentence reads much more as talking about which CH bond the singlet oxygen is going to react with….and in that context it makes no sense. The authors should focus their discussion on the observation that they think they have almost complete chemical quenching (as opposed to physical quenching) and what they think the CH selectivity is due to. Speculation about PDT is also reasonable, but of course, there's no reason to think that people will change what they are doing with PDT, but it might give them something else to look for in the chemical outcomes of PDT.

In reference 31, it's "dioxane" not "dioxerane", at least to the best of my knowledge.

Replies to reviewers' comments (NCOMMS-16-05981A-Z).R2

Reviewer #2 (Remarks to the Author):

I regret to report that the authors' revised manuscript and rebuttal have not convinced me that this work is ready to be published (in Nature Communications or any other journal). Rather, the documents submitted this second time around strongly suggest to me that the authors still make some rather exorbitant claims that are not substantiated.

Listed below are selected points and examples that contributed to my opinion.

Q1. In my first review, I suggested that the authors make singlet oxygen using an approach that does not involve the addition of other molecules to the ether solution (e.g., sensitizers, H2O2, HOCl). I suggested they exploit the fact that singlet oxygen can be made upon direct irradiation of oxygen at 765 nm, and I am pleased to see that the authors took my advice in this regard. However, as best as I can tell from the information provided, rather than providing data to support their claims, the experiments performed by the authors support my contention that something else is happening in this system. Specifically, the authors indicate that, to directly excite oxygen, they irradiated the solution at 780 nm. However, as can be seen in the pertinent paper on this subject (their reference 21), oxygen does not absorb at this wavelength. Thus, unless they used a laser with a very broad spectral output (not indicated in the paper), something else must be absorbing the light that, in turn, triggers the reactions they observe. In short, they should see a pronounced difference in the reactivity of the ethers upon irradiation at 780 nm and 765 nm, respectively.

Our reply: We respectfully disagree with the comment that "as can be seen in the pertinent paper on this subject (their reference 21), oxygen does not absorb at this [780 nm] wavelength". As reported by P. R. Ogilby and coworkers (ref. 21 in original manuscript, or ref. 13 in the revised manuscript) that the absorption coefficient of molecular oxygen at 780 nm is NOT zero, but is 13% of that at 765 nm (see the Figure 3a shown below, which is adopted from Figure 3a of the ref. 21). In our 780 nm light irradiation experiment, the diode laser we used has a maximum output wavelength of 780 ±5 nm. Therefore, molecular oxygen still has a significant amount of light absorption at 780 nm. Irradiation of 780 nm laser light leads to direct excitation of molecular oxygen to form singlet oxygen and subsequent singlet oxygen-THF reaction products. Since this reviewer insists to see results using 765 nm light excitation, we accept the suggestion and carried out 765 nm light excitation of a THF-O_2 experiments. In our lab, we do not have a Ti:sapphire laser to provide 765 nm laser light. We carried out this 765 nm light experiments in one of my colleagues, professor Po-Yuan Cheng's lab (Department of Chemistry, National Tsing Hua University, Hsinchu, Taiwan) using the output of a Ti:sapphire laser (Spectra Physics, Tsunami) at 765 nm (with the laser set in the non-self mode lock quasi CW mode: 375 mW with a light spot of 0.5 cm diameter) to excite molecular oxygen directly. One can find the

description of the Ti-saphire laser in the following reference: Hsueh-Ying Chen, I-Ren Lee, and Po-Yuan Cheng, Rev. Sci. Instrum. 2006, **77**, 076105; doi: 10.1063/1.2221545. The experimental setup is shown below in the Figure Y. The THF is freshly distilled prior to the experiments. As shown in the Figure 1(a), 6.5 h direct 765 nm light irradiation of a THF-O_2 balloon solution results in the formation of THF-hydroperoxide and THF lactone, of which the products are precisely the same as those obtained using 780 (± 5 nm) laser light irradiation (see Figure 1(c) below). A control experiment shows that when O_2 balloon was replaced by Ar gas, no any product was detected under the same 765 nm light irradiation condition (see Figure 1(b)).

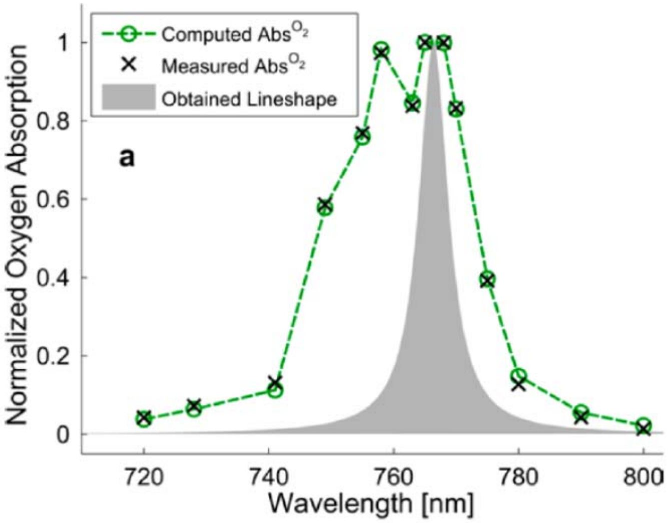

Figure 3a The computed and experimentally measured absorption of molecular oxygen as a function of wavelengths (adopted from Figure 3a of ref. 13; P. R. Ogilby, et. al. J. Phys. Chem. B 2015, 119, 5422-5429). The absorption coefficient of molecular oxygen at 780 nm is NOT zero, but is 13% of that at 765 nm.

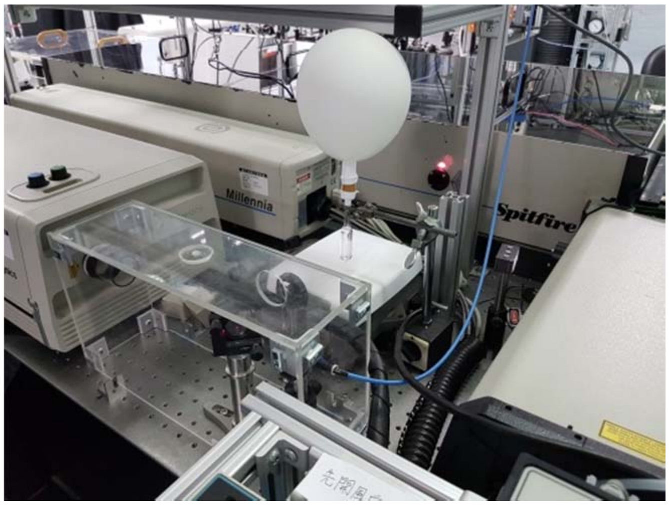

Figure Y. The setup for direct 765 nm laser irradiation of a THF-molecular oxygen solution (the glass vessel with a white O_2 bloom). The 765 nm laser light was from the output of a Ti:sapphire laser (Spectra Physics, Tsunami) under a non-self mode lock quasi CW mode with an output power of 375 mW and a laser spot (see the white-red spot) of ~ 0.5 cm in diameter. The THF is freshly distilled prior to experiments, and the irradiation time was 6.5 h. These results unambiguously demonstrates that the singlet oxygen, generated via direct photo-excitation of molecular oxygen by 765 nm light, can directly react with THF to generate THF-hydroperoxide, most probably via one step direct insertion into the alpha ethereal C-H bond.

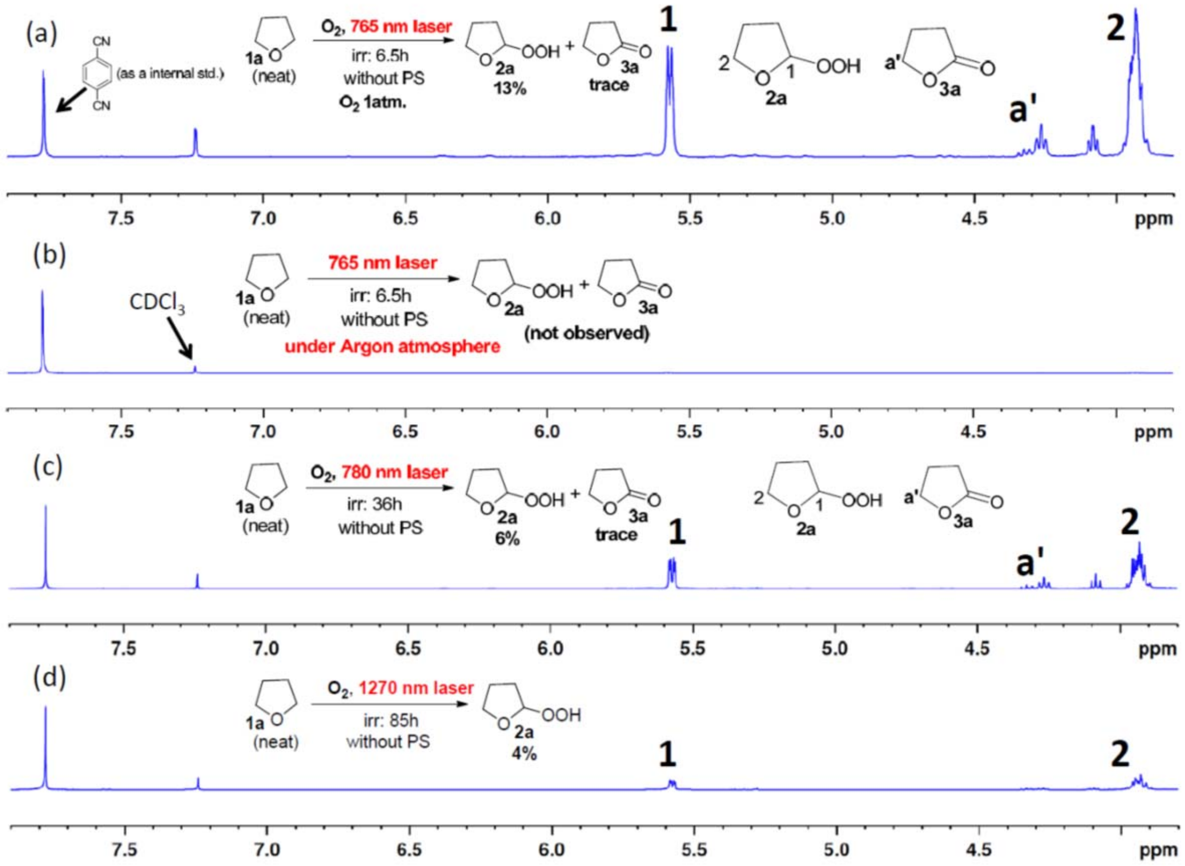

Figure 1. Photo irradiation of a freshly distilled neat THF solution under different conditions: (a) O_2 atmosphere, 765 nm, 375 mW/(0.5 cm diameter spot), CW mode, 6.5 h; (b) Ar atmosphere, 765 nm, 375 mW/(0.5 cm diameter spot), CW mode, 6.5 h; (c) O_2 atmosphere, 780 (± 5) nm, 75 mW/cm^2, CW mode, 36 h; and (d) O_2 atmosphere, 1270 nm, 2.5 mW/(0.5 cm diameter spot), CW mode, 85 h.

In the literature, it was reported by the late professor Nick J Turro (this author's Ph. D. advisor at Columbia Univ) that singlet oxygen can also be formed by direct 1270 nm photo-excitation of molecular oxygen (see, N. J. Turro et al., *Photochem. Photobiol. Sci.*, 2008, ***7***, 235–239). Therefore, we also carried out an experiment using 1270 nm CW diode laser light (2.5 mW with a laser spot diameter of 0.5 cm, Light Source, Model No.:S20012009999, Liverage Technology Inc, Hsinchu, Taiwan) to irradiate a THF-O_2 balloon solution (see the optical picture below for experimental setup). After 85 h irradiation, one could clearly detect the formation of THF-hydroperoxide by ^{1}H NMR spectroscopy (see the Figure 1(d) below). The requirement of a long irradiation time and a low yield (4% using a known amount of 1,4-dicyanobenzene as an external standard in ^{1}H NMR measurements) of THF-hydroperoxide is understandable, since our 1270 nm light intensity is very weak (2.5 mW with a laser spot diameter of 0.5 cm).

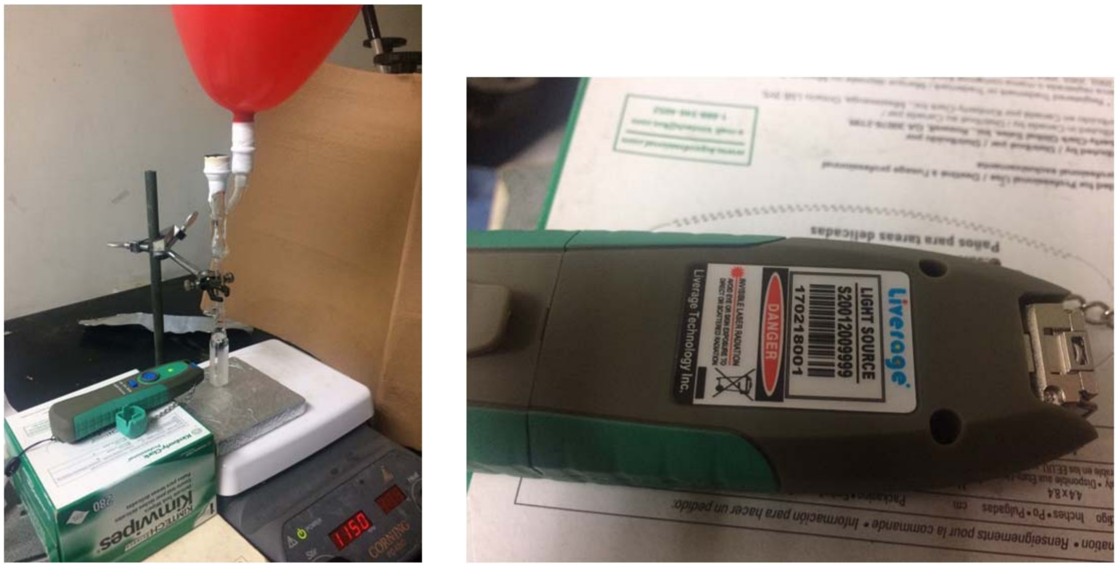

Figure Z. The experimental setup of 1270 nm light irradiation of a neat THF-O_2 solution. The light source is from the output of a CW diode laser light (2.5 mW with a laser spot diameter of 0.5 cm, Light Source, Model No.:S20012009999, Liverage Technology Inc, Hsinchu, Taiwan).

Overall, we have presented 6 different independent lines of experimental evidences to support the chemical reaction of singlet oxygen, including (a) phosphorescence of singlet O_2 at ~1270 nm was observed; (b) In the absence of photosensitizers, no any products and singlet O_2 phosphorescence could be observed under the same condition; (c) The presence of NaN_3, a well-known singlet O_2 quencher, dramatically suppresses the yields of **2a** and **3a**; (d) generation of singlet O_2 via a dark reaction of H_2O_2 with strong oxidants (such as, HOCl or $NaMoO_4$) in THF also leads to formation of the THF-hydroperoxide and lactone products, (e) addition of olefins to the THF-meso TPP solution results in the formation of expected singlet O_2-ene reaction products and decreases in the product yields of THF-hydroperoxide and lactone. In the previous run of review, this reviewer did not disagree that that evidences (a)~(e) are well accepted in the literature to be specific for the involvement of singlet oxygen in the reaction. Now, we have presented two additional supporting evidences using 765 and 1270 nm light to excite molecular oxygen directly to generate singlet oxygen in THF. The products are all the same when using 765, 780, and 1270 nm light excitation of a simple THF-O_2 solution. All the above mentioned experiments unambiguously demonstrate that singlet oxygen is the key and the only species, which reacts with THF to generate THF-hydroperoxide and subsequent decomposition/dehydration product, THF-lactone. We have added these new results into the revised manuscript as Figure 1.

Q2. To make global conclusive statements throughout the manuscript about how their data from selected ethers solve the "35-year old mystery" regarding the effect of solvent on the singlet oxygen lifetime is not justified. Furthermore, to support such statements with a vague and unsubstantiated footnote (their reference 31) about experiments with other solvents ("data not shown") is not acceptable. With respect to the latter, I am particularly interested in seeing what the authors claim is happening in water.

Our reply: Yes, we agree with this reviewer that"…….. solve the "35-year old mystery" regarding the effect of solvent on the singlet oxygen lifetime is not justified", since we only presented chemical reaction of singlet oxygen with two solvents, namely, THF and 1,4-dioxane, in the manuscript. It is not possible to resolve a long standing literature problem in one paper, and to avoid complication, we have removed the word "solve 35-year old mystery", and re-stated as "This study provides important clues to the 35-year old literature mystery regarding the huge lifetime variation of singlet oxygen in various solvents by taking into account the chemical reaction pathway of singlet oxygen with weakly activated solvent C-H bonds.". We will organize our singlet oxygen reactions with other solvents in a follow-up paper.

Q3. If the authors choose to go beyond the "simple" report that singlet oxygen reacts with ethers and, instead, make global statements about the mechanism(s) for the general effect of solvent on the singlet oxygen lifetime, then a more systematic and detailed study needs to be presented. In such a work, the authors would need to properly address the existing work they claim is wrong, and show exactly why they think that work is incorrect. In such studies, it is indeed generally assumed that the singlet oxygen lifetime is determined by physical rather than reactive quenching. Thus, for the appropriate proof of their claims, the authors would need to identify the solvents that do not behave according to a given model of physical deactivation, and show that everything fits properly when the given lifetime is adjusted for the observed component of reactive quenching.

As stated in my first report, I do not doubt that the authors are making singlet oxygen in their sensitized experiments. What they fail to do is show, in a convincing way, that their chemistry is the result of a direct reaction between singlet oxygen and the ether. In short, they still have not properly excluded other reaction possibilities. Again, as indicated in my first report, I find it most interesting that the authors' case in built upon a solvent system (ethers) that is notoriously "dirty" and that could support a host of reactions (e.g., free radical and electron transfer). The other solvent systems that they cite in their defense (reference 31, "data not shown"), likewise are notorious for supporting a host of reactions.

Our reply: As we stated in our reply to Q2, it is not possible to clarify all issues involved in a long standing literature problem in one paper. Therefore, we have removed

the word "resolve 35-year old literature problem". We have re-stated in the revised manuscript that "This study provides important clues to the 35-year old literature mystery regarding the huge lifetime variation of singlet oxygen in various solvents by taking into account the chemical reaction pathway of singlet oxygen with weakly activated solvent C-H bonds." We will address the reaction of singlet oxygen with other solvents in a follow up paper.

Although it is known that THF can easily form hydroperoxides once its container is open and exposed to air and room light for 6~12 months, we use freshly distillated THF in all experiments. As we can see from ^{1}H NMR that freshly distillated THF does not contain any hydroperoxide, lactone, or other stabilizers. We have run many control experiments, as stated above in our replies to Q1 and replies in the 2nd run of replies to reviewers comments. We have also run the control experiment suggested by this reviewer, that is, direct photo excitation of molecular oxygen, by either 765 nm (375 mW/(0.5 cm diameter spot), CW mode) or 1270 nm, to generate singlet oxygen in a freshly distillated neat THF. The system is the simplest one without any other reagents or high laser power. The possibility of involvement of radical species and electron transfer process were excluded by the control experiments that the product yield and distribution are negligibly affected by the presence of a radical scavenger, DMPO, and the absence of product formation by dark reaction of KO_2 (a stable source of superoxide, O_2^-) with THF. All results and products are consistent with each other and all support the conclusion that singlet oxygen is the species which reacts with THF to give the THF-hydroperoxide and lactone products. We have added the results regarding KO_2 and radical scavenger, DMPO, experiments into the main text in the revised manuscript (page 9, the 2nd paragraph in the Reaction Mechanism section).

Reviewer #3 (Remarks to the Author):

This manuscript is much improved from the earlier version and is probably suitable for publication after a few more minor revisions. I think it is an excellent example of the peer-review process working because it is so much better than before. The addition of several control experiments and the chiral chromatography are big plusses.

Our reply: Thanks for all the positive comments and suggestion of acceptance of our work after a few minor revisions.

Q1. The major flaw in the paper is that some of the language in it feels like a stretch to the knowledgable chemist involved in singlet oxygen and other physical organic chemistry. I feel obliged to be fussy on such points for a paper submitted to Nature. For example, the authors still maintain in the introduction language that seems to equate the further oxidation of this ethereal carbon to oxidation of “unactivated” methane or internal methylenes. This is a pre-activated carbon, one that is easier to oxidized by several mechanisms (apparently including this new one) than others. If I were writing this paper, I would completely delete all discussion of CH activation and focus on the singlet oxygen novelty, as the authors do in their extensive “response to authors” section. The abstract and introduction are still misleading.

Our reply: Thanks for your editorial-like suggestions. We have removed the part related to C-H functionalization of unactivated C-H bonds in the introduction section, since the alpha-ethereal C-H bonds in THF is actually partially activated. We have focused major emphasis on the singlet oxygen novelty in the revised manuscript.

Q2. My other major complaint is that the reported yields are so arbitrary. I assume that RSM stands for “remaining starting material”, but the authors make no attempt to actually maximize yields, while simultaneously talking about their synthetic utility. If the vast majority of material is starting material plus products after 12 hours, why haven’t the authors (in at least a few cases) actually taken the reaction out to conclusion, either by increasing the light intensity or by extending the time of photolysis? These “moderate yields” are of no particular meaning if they are only moderate because they stopped the reaction before starting material was consumed or other secondary reactions started happening. You kind of have to choose: are you going to focus on the singlet oxygen novelty, or are you ALSO going to talk about the synthetic utility. If the latter, you need to at least make an effort to optimize a few yields.

Our reply: The major emphasis of our manuscript was put more on the physical organic view of singlet oxygen novelty that it was never reported to be able to react with weakly activated alpha-ethereal C-H bonds, which provides an important clue to the 35 pending literature mystery about the huge variation of singlet oxygen lifetimes in different solvents.

Certainly, chemical reactivity of singlet oxygen towards partially activated C-H bonds will have some synthetic applications. In the current work, we indeed did not intend to optimize the yields of all reactions. The yields we reported here range from moderate to good, which, from some organic chemists' point of view, are acceptable. Presentation of some "moderate" yields of singlet oxygen reactions is to illustrate the wide substrate scope of this previously unrecognized singlet oxygen reaction. Some organic chemists might find this novel singlet oxygen chemical reaction pattern interesting and will probably develop some applications in the future. Synthetic utility of this singlet oxygen reaction is the secondary selling point in our manuscript. A good paper should not contain only a single emphasis. Besides, a new chemical reaction always has its own physical as well as organic aspects. They are related, but do not conflict with each other. Yes, "rsm" represents "remaining starting material". We have added to full term of "rsm" to the manuscript at its first appearance.

Q3. The discussion of hydroperoxide vs lactone is still not satisfactory. I don't even see a single mention of the possibility/probability that all of the singlet oxygen chemistry generates hydroperoxide, and the dehydration to lactone is simply catalyzed by the Lewis Acid. The computations can't really address this at all, since there are such dramatic effects of charge separation in gas phase calculations and to do anything of "chemical accuracy" would become enormously expensive on top of the current MP2-CAS computations.

Our reply: We agree with this reviewer that it is too expensive, on top of the current MP2-CAS computations, to calculate the catalytical conversion of THF-hydroperoxide to lactone by Lewis acid or other metal ion catalysts. In the theoretical calculation section, we have pointed out that due to the high excess energy of 51 kcal/mol, resulting from the **CI-1** to THF-hydroperoxide **2a**, the barrier of 48 kcal/mol from the THF-hydroperoxide **2a** to a more stable lactone product **3a** can be easily overcome in the absence of any catalysts (see the theoretical calculation section in the revised manuscript). .

Alternatively, from bond strength point of view, the O-O bond strength (~ 35 kcal/mol) of THF-hydroperoxide is very weak, and decomposes easily to form free O centered radical, which then undergoes H atom abstraction, via breakage of a C-H bond (~98 kcal/mol), to form a strong O-H bond (~ 110 kcal/mol) and a pi-bond in >C=O (~88 kcal/mol), leading to the formation of corresponding lactone. Overall, such an O-O bond cleavage in hydroperoxide or hydroperoxy acetals is exothermic and thermodynamically allowed. Rearrangement of hydroperoxide (via the Hock rearrangement mechanism) or hydroperoxy acetals to form corresponding ketones, esters, or lactones, respectively, are well documented in the literature (Tetrahedron Letters 2010, 51, 5615–5617; *Beilstein J. Org. Chem.* **2016,** *12,* 1647–1748). These rearrangements can also be catalyzed by metal ions or Lewis acids.

Rapid decomposition of hydroperoxides and subsequent explosion often occur by mistakenly adding metal ions-containing waste water into hdyroperoxides containing-organic lab. wastes. We have added a few sentences and cite the above references to describe the rearrangement of our THF-hydroperoxide to lactone in the revised manuscript (page 5, the first paragraph).

Q4. Another silly point to continue to emphasize (because it is a straw man) is that the singlet oxygen system is different than hydroperoxide and organometallic catalysts. I fail to see the point. A mention of two control experiments outlined in the response (KO_2 and spin trapping) should be made in the main text, with reference to details in the supporting material.

Our reply: Singlet oxygen is different from hydroperoxides and organometallic catalysts when it chemically reacts with THF to form peroxide products. The key difference is that gaseous singlet oxygen was consumed without forming any unwanted side products, whereas solid/liquid organic hydroperoxide and organometallic oxidants will generate stoichiometric wastes (organic alcohol and metal oxides, respectively).

Yes, as per this reviewer's suggestion, we have added the KO_2 and spin trapping results into the main text in the revised manuscript in page 9, second paragraph.

Q5. The authors over-react to the olefinic quenching in MTBE, in the sense that olefins are not the only thing that can act as "impurity quenchers". For example, in THF, water will affect the lifetime of singlet oxygen if it gets to high enough concentration. (This may make the full conversion of the ethers to product a little more difficult!).

Our reply: Yes, we are aware that water is also a potential chemical quencher of singlet oxygen. To avoid complication, the THF used in all our experiments are freshly distillated to remove and water and other impurities prior to experiments.

Q6. It should be made clear relative to what the yields are being measured earlier in the first Results paragraph. Eventually, we figure out that they mean relative to complete conversion, but that isn't obvious at first and is confusing. In the first paragraph of page 8, "Interestingly" is misused. According to the previously reported results, that should be "As expected," or "In line with the model compounds,". In the discussion of the stereochemistry on page 9, the authors state "The chiral [product] has the same steric configuration as, but an opposite chirality from (due to conversion of light C-H to heavy OOH group), the starting substrate…" It would be less clumsy and convey just the same information to say that "the oxidation of CH to COOH proceeds with at least nearly complete retention."

Our reply: As suggested, we have defined the product yield in the footnote of Table 1 as

below: “% of yield was expressed with respect to the starting materials upon 100% conversion”.

The word “Interestingly” in the *Synthetic Application* section has be replaced by “As expected”. Thanks for the editorial suggestion.

In the discussion of the stereochemistry on page 9, we have also replaced the statement “The chiral (R)-2-hydroperoxyl-2-methyl THF has the same steric configuration as, but an opposite chirality from (due to conversion of light C-H to heavy OOH group), the starting substrate…” by the statement “The chiral (R)-2-hydroperoxyl-2-methyl THF has the same steric configuration as the starting substrate ($[\alpha]_D$ +19.40°, see SI).(*22*). The oxidation of C-H to COOH proceeds with at least nearly complete retention.”, as suggested by the reviewer.

Thanks for editorial comments and suggestions. We have taken the suggestions and revised our text writing accordingly. In addition, we have had our manuscript corrected/checked by a native English speaking, professional editor.

Q7. A conical intersection cannot be an intermediate (which is by definition a thermodynamic minimum). Please clarify. Also, please note the irrelevance of the computations to a normal acid-catalyzed dehydration mechanism.

Our reply: Yes, we agree with this reviewer that a conical intersection cannot be an intermediate, which is by definition a thermodynamic minimum. We have removed the “intermediate structure” term. We have also added a few sentences at the end of theoretical calculation section to state that the calculation shown in Figure 4 is under the condition in the absence of any catalysts. It has been reported in the literature that metal ions/Lewis acid can catalyze dehydration of hydroperoxide to lactone (*17*). The presence of catalysts may find other pathways with lower activation energies. However, involvement of catalysts in theoretical calculation is too complicated, and is beyond the scope of this study.

Q8. The discussion of “site selectivity of singlet O_2 [being] most probably governed by its spin multiplicity” still makes no sense. I understand that they are saying that ground state triplet oxygen and singlet oxygen are different, but this sentence reads much more as talking about which CH bond the singlet oxygen is going to react with….and in that context it makes no sense.

Our reply: Thanks for pointing out the confusion point in our manuscript. Now, we have changed the statement to be “The site selectivity of singlet oxygen reaction with THF is governed by the C-H bond strength and activation energy. From theoretical calculation, it is clear that both the alpha-ethereal C-H bond strength and the energy barrier for direct singlet oxygen C-H insertion reaction are much lower than those for the beta-ethereal C-H bonds. Therefore, direct insertion of singlet oxygen occurs selectively at the alpha-ethereal

C-H bonds". We have added the above discussion to the first paragraph of the Discussion section.

Q9. The authors should focus their discussion on the observation that they think they have almost complete chemical quenching (as opposed to physical quenching) and what they think the CH selectivity is due to. Speculation about PDT is also reasonable, but of course, there's no reason to think that people will change what they are doing with PDT, but it might give them something else to look for in the chemical outcomes of PDT.

Our reply: Thanks for your editorial suggestions. Yes, we have emphasized more on the complete chemical quenching of singlet oxygen by C-H bonds of various solvents, as compared to physical quenching, in the revised manuscript.

Q10. In reference 31, it's "dioxane" not "dioxerane", at least to the best of my knowledge.

Our reply: Thanks for pointing out our mistake. It should be 1,4-dioxane. We have corrected the error. Now, the ref. 31 has been removed. We will prepare a follow-up paper to discuss about singlet oxygen reactions with other solvents.

Reviewer #3 (Remarks to the Author):

This paper is improved over the previous version and I recommend publication after minor revisions. I have been asked to consider the comments of both previous referees and whether their concerns have been addressed. I have reviewed them carefully and I find the responses generally quite satisfactory. Both referees might quibble here and there, but the authors have gone a long way in addressing the major concerns.

Below are my specific remarks over this version:

The introduction would be improved with a sentence or two that *specifically* gives an example of inconsistency with the well-known physical quenching model. Most people familiar with singlet oxygen will not be acquainted with the problem they purport to solve.

Furthermore, a qualitative view here is that they are not actually showing the physical quenching model to be wrong, but instead they are showing that there is a previously unknown chemical quenching path (which presumably explains an anomalously short lifetime, but I do not know the old data well enough).

THF (or any ether) is not considered an "inert" solvent (Results, second line) by anyone involved in radical chemistry. In that same line, "solvent molecules" is written in such a way as to make this seem overly broad. It's a pretty specific set of activated CH bonds we are talking about.

The definition of yield is still very ambiguous On page 3, I believe it's just being used in the sense of how many molecules are obtained relative to the original amount of THF. The difference between conversion and yield needs to be kept in mind. A ~50% yield (Table 1) should mean that there is another 50% of the material decomposed into unidentifiable products, not remaining starting material that could be converted with enough patience.

The authors should clarify if they are using air saturated or oxygen saturated solutions in the main text.

The statement about reference 11 in lines 66-67 may be important, but it is a non sequitur in that paragraph and interrupts the flow.

The authors are very vague about the purpose of the alumina. I don't believe any mechanistic explanation of how they improve yields is given, but maybe I missed it. This is also important in that acid-catalyzed dehydration of the hydroperoxide can also lead to the furanone product.

The authors seem to postulate that formation of the furanone is due to O-O hemolysis of the hydroperoxide. This is only weakly supported by the results, if at all. They need to give a reference for the stated value of the O-O BDE. They should also address why there are not multiple products derived from hydrogen abstraction by the very non-specific HO radial. All it has to do is escape from the primary cage, and it will surely react with more THF. In the presence of O2, that could lead to autoxidation to the observed products! The authors seem to be vague on whether they think the hemolysis is thermal or photochemical.

A quantum yield for chemical conversion of the singlet oxygen would be extremely useful in knowing whether this chemical deactivation of 1O2 is kinetically important. If the quantum yield from 1O2 is near 100%, it obviously is; if it is 1% or something, then it's quite possible that the lifetimes are not going to be affected much.

The authors should do something to clarify that Figure 3 is not based on an actual computed geometry of the insertion Transitionsstate

I find the argument in the discussion very puzzling, with respect to the effect of the alumina. The CH bond in THF is *activated* by the adjacent lone pairs of electrons. How does making a Lewis Acid

The authors should narrow the breadth of the claim in the last couple of lines.

Point to point replies to reviewers' comments (**NCOMMS-16-05981B-Z**)

Reviewer #3 (Remarks to the Author):

This paper is improved over the previous version and I recommend publication after minor revisions. I have been asked to consider the comments of both previous referees and whether their concerns have been addressed. I have reviewed them carefully and I find the responses generally quite satisfactory. Both referees might quibble here and there, but the authors have gone a long way in addressing the major concerns.

Our reply: Thanks for all the positive words and fair evaluation regarding our submission.

Below are my specific remarks over this version:

Q1. The introduction would be improved with a sentence or two that *specifically* gives an example of inconsistency with the well-known physical quenching model. Most people familiar with singlet oxygen will not be acquainted with the problem they purport to solve. Furthermore, a qualitative view here is that they are not actually showing the physical quenching model to be wrong, but instead they are showing that there is a previously unknown chemical quenching path (which presumably explains an anomalously short lifetime, but I do not know the old data well enough).

Our reply: As per requested by this reviewer, we have specifically pointed out two examples in the section of "35-years literature mystery" (page 2 in the revised manuscript), where those theoretically predicated values using solvent C-H bond vibration-induced physical perturbation models are inconsistent with experimentally determined singlet oxygen lifetimes in different solvents, especially in cases where the vibrational frequencies of solvent C-H bonds were tuned via either replacing H by D (references 6, 8) or replacing unstrained hydrocarbons by strained hydrocarbons (ref. 9).

Q2. THF (or any ether) is not considered an "inert" solvent (Results, second line) by anyone involved in radical chemistry. In that same line, "solvent molecules" is written in such a way as to make this seem overly broad. It's a pretty specific set of activated C-H bonds we are talking about.

Our reply: Yes, THF and many other ethers are not inert solvents. We have removed the words "commonly thought "inert"". To be more precise, we have also restricted the solvent molecules to be THF and some cyclic/linear ethers. The revised sentence is quoted below:

In this study, we demonstrate that chemical reaction between singlet O_2 and ~~commonly thought "inert"~~ C-H bonds of solvent molecules is the major excited state deactivation channel for singlet O_2 in THF and some cyclic as well as linear ethers (Tables 1 and 2), which was never considered before in the literature as one of the possible

deactivation channels.

Q3. The definition of yield is still very ambiguous On page 3, I believe it's just being used in the sense of how many molecules are obtained relative to the original amount of THF. The difference between conversion and yield needs to be kept in mind. A ~50% yield (Table 1) should mean that there is another 50% of the material decomposed into unidentifiable products, not remaining starting material that could be converted with enough patience. The authors should clarify if they are using air saturated or oxygen saturated solutions in the main text.

Our reply: We report the product yields by following the same way as that commonly adopted in the field of organic synthesis; that is (the amount of product divided by original amount of the major reactant) x 100%. To be more precise, on has to describe the rest of materials, either unidentified products or remaining unreacted starting materials. In our case, the rest of material is unreacted starting materials. We have added an additional column "conversion %" to both Tables 1 and 2. Meanwhile, we have also stated in the "footnote a" of Tables 1 and 2 that besides the listed products, the rest of materials are unreacted starting substrate. We have also specified the experimental condition of "~1 atm O_2 balloon" to the legends of Tables 1 and 2. Thanks for all the editorial suggestions.

Q4. The statement about reference 11 in lines 66-67 may be important, but it is a non sequitur in that paragraph and interrupts the flow.

Our reply: As per your suggestion, we have removed the sentence about reference 11 from the first paragraph of the Results section, and relocate this sentence to the end of the Discussion section.

Q5. The authors are very vague about the purpose of the alumina. I don't believe any mechanistic explanation of how they improve yields is given, but maybe I missed it. This is also important in that acid-catalyzed dehydration of the hydroperoxide can also lead to the furanone product.

Our reply: In the first paragraph of the Discussion section, we have mentioned that coordination of Lewis acid to the ethereal oxygen can weaken the α-ethereal C-H bonds, which favors the attack of α-ethereal C-H bonds by singlet oxygen and thus the product yields. In the Theoretical calculation section, we have also mentioned that "It has been reported in the literature that metal ions/Lewis acid can catalyze dehydration of hydroperoxide to lactone (18). The presence of catalysts may find other pathways with lower activation energies. However, involvement of catalysts in theoretical calculation is too complicated, and is beyond the scope of this study."

Q6. The authors seem to postulate that formation of the furanone is due to O-O hemolysis of the hydroperoxide. This is only weakly supported by the results, if at all. They need to give a reference for the stated value of the O-O BDE. They should also address why there are not multiple products derived from hydrogen abstraction by the very non-specific HO radical. All it has to do is escape from the primary cage, and it will surely react with more THF. In the presence of O_2, that could lead to autoxidation to the observed products! The authors seem to be vague on whether they think the hemolysis is thermal or photochemical.

Our reply: The weak O-O bond strength (34~45 kcal/mol) and its easy decomposition to form hydroxyl and alkoxyl radicals are well documented in the literature (*J. Am. Chem. Soc.* **1996,** *118,* 12758-12765). As per this reviewer's request, we have added the above JACS 1996 paper as a reference to the main text.

Regarding the hydrogen atom abstraction reaction by hydroxyl radical (.OH), results in many literature papers have clearly shown that it is NOT non-specific or random site selectivity, especially in **organic solvent** systems. For example, rearrangement or decomposition of hydroperoxide (via the Hock rearrangement mechanism) or hydroperoxy acetals to form corresponding ketones, esters, or lactones, respectively, are well documented in the literature.(*11,17*) Hydrogen atom abstraction from the weakest germinal C-H bond by hydroxyl radical is easiest, exothermic and most favorable, which leads to formation of ketones, esters or lactones, as reported in the references 11 and 17. The reason for such kind of excellent site selectivity (and thus simple product distribution) is probably due to slow chemical reaction rate (or high energy barrier) of hydroxyl radical in **aprotic organic solvent** systems. It has been reported that the hydrogen atom abstraction rate of hydroxyl radical is diffusion controlled in **aqueous** solutions, but becomes 2 orders slower in aprotic organic solvents, which is due to unfavorable formation of polar transition state in nonpolar aprotic organic solvents (see, J. Am. Chem. Soc **2010**, *132*, 2907–2913). Slower hydrogen atom abstraction rate leads to excellent site selectivity and simple product distribution.

If hydroxyl radical can escape from solvent cages and further exert autooxidation chain reaction of THF in the presence of O_2, the amounts of THF-hydroperoxide products will be dramatically enhanced, leading to the quantum yield of THF oxidation products to be much larger than 1.0. As shown in our reply to Q7, the quantum yield of THF oxidation products relative to the formation of singlet oxygen is 0.93, less than 1.0. Therefore, our experimental result does not support the possibility of hydroxyl radical solvent cage escape-THF autooxidation process.

In the references 11 and 17, the decomposition of the weak O-O bond occurs thermally. In addition, the O-O single bond has weak absorption in the short uv region. Therefore, we do not expect visible light can induce cleavage of the O-O bond in THF-hydroperoxide. We have added "thermal decomposition occurs easily" to the main text in the revised

manuscript.

Q7. A quantum yield for chemical conversion of the singlet oxygen would be extremely useful in knowing whether this chemical deactivation of $^1O_2$ is kinetically important. If the quantum yield from $^1O_2$ is near 100%, it obviously is; if it is 1% or something, then it's quite possible that the lifetimes are not going to be affected much.

Our reply: The chemical quantum yield of singlet O_2 in THF, by definition, can be obtained via two methods: (a) the ratio of "moles of THF-singlet oxygen reaction products" vs. "the moles of singlet oxygen formed"; or (b) the observed apparent chemical reaction rate (k_1[THF]) of singlet oxygen with THF divided by the total rates ($k_0 + k_1$[THF]) of all physical (k_0) and chemical (k_1[THF]) deactivation channels (see the chemical equation shown below).

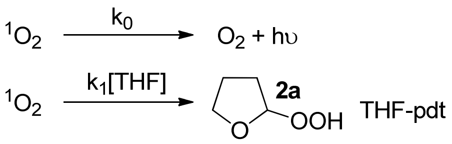

(S2a)

(S2b)

In neat THF

$$k_{Total} = k_0 + k_1[THF]$$

$$\text{Chemical quantum yield,}\Phi_{THF\text{-}pdt} = \frac{k_1[THF]}{k_0 + k_1[THF]} \quad (3)$$

In CCl_4, the physical deactivation rate, k_0 value, is 1/(900 μs)=1.1 x 10^3 s^{-1}. In neat THF, the k_0 value is assumed to be (13/4= 3.25) times faster than that for CCl_4, since THF has 13 (C-H, C-C or C-O) single bonds as compared to 4 C-Cl single bonds for CCl_4. To the first approximation, this assumption is valid since solvent nuclear vibrational motion is the main exit channel responsible for the physical deactivation (via electronic-vibrational coupling) of singlet O_2. Therefore, the k_0 value for THF is tentatively assumed to be approximately 1.1 x 10^3 s^{-1} x 3.25= 3.6 x 10^3 s^{-1}. From the competition reaction with 1-methylcyclohexene, we obtained the chemical reaction rate of singlet O_2 with THF to be 3.8 x 10^3 $M^{-1}s^{-1}$. Neat THF has a THF concentration of 12.35 M. Therefore, the chemical quantum yield of product formation for singlet O_2 chemical reaction with THF can be obtained by the equation (3):

$$\Phi_{THF,pdts} = k_1[THF]/\ (k_0 + k_1[THF])$$

$$= (3.8 \times 10^3\ M^{-1}s^{-1} \times 12.35\ M)/\ (3.6 \times 10^3\ s^{-1} + 3.8 \times 10^3\ M^{-1}s^{-1} \times 12.35\ M)$$

$$= 0.93$$

The physical deactivation rate of singlet O_2 in THF adopted in the above calculation may not be accurate. Nevertheless, the physical deactivation rate is at least one order slower than the chemical reaction rate of singlet O_2 in THF. Small variation in the physical

deactivation rate will not change significantly the chemical quantum yield value (here, 0.93) of singlet O_2 in THF.

We have explicitly incorporated the above calculation in the supplementary information and stated the chemical quantum yield value into the main text of revised manuscript (page 6, first paragraph). We have also explicitly stated in the revised manuscript that chemical reaction of singlet O_2 with THF solvent molecules is the dominant deactivation channel responsible for the short lifetime (20 μs) of singlet O_2 in THF, as compared to the very long lifetime (900 μs) of singlet O_2 in CCl_4. Such a chemical deactivation channel was never considered or recognized before in the past 35 years to account for short lifetimes of singlet oxygen in various solvents.

Q8. The authors should do something to clarify that Figure 3 is not based on an actual computed geometry of the insertion Transitions state. I find the argument in the discussion very puzzling, with respect to the effect of the alumina. The C-H bond in THF is *activated* by the adjacent lone pairs of electrons. How does making a Lewis Acid

Our reply: We have added a sentence to the legend of Figure 3 to explain that "The optical rotation values and ee values are based experimental measurements (see Supplementary information for details)". We agree with this reviewer that "The C-H bond in THF is *activated* by the adjacent lone pairs of electrons". We have also stated in the main text that the alpha-C-H bond adjacent to the ethereal oxygen is weakly activated. Coordination of Lewis acid to the ethereal oxygen can further weaken the alpha C-H bonds, rendering the direct C-H bond insertion by singlet oxygen easier and thus higher yields of the final products (more favorable for synthetic applications).

Q9. The authors should narrow the breadth of the claim in the last couple of lines.

Our reply: At the very end of the manuscript, we wrote that "We believe that such a new type of singlet O_2 reaction will soon find its wide applications in synthetic chemistry and important roles in photodynamic therapy destruction of cancer cells. This study provides important clues to the 35-year old literature mystery regarding the huge lifetime variation of singlet oxygen in various solvents by taking into account the chemical reaction pathway of singlet oxygen with weakly activated solvent C-H bonds". These are not really claims, but to point out the potential influence and applications of our current discovery regarding the new chemical reaction patterns of singlet oxygen. In the 2nd run review process, the reviewer #3 commented that "Speculation about PDT is also reasonable, but of course, there's no reason to think that people will change what they are doing with PDT, but it might give them something else to look for in the chemical outcomes of PDT". Since these implications are not really claims, we prefer to leave them there, unless this reviewer insists to make changes.

Reviewer #3 (Remarks to the Author):

I believe this paper is a very good example of the anonymous review process at work. The paper is significant and now answers the questions that a broader audience than originally intended will want to know. The authors have done a great job of listening to feedback of the various referees (including me) and providing thoughtful responses and changes to the manuscript.

The paper is ready for publication and should make a significant impact.

Replies to editorial comments (NCOMMS-16-05981C)

Editorial questions regarding main text.

Q1. The English would benefit from careful grammar editing. Please have an English collegue to check the manuscript and SI.

Our reply: Yes, we have had our manuscript corrected by a professional English editing service company (see attached proof for the English editing service).

Q2. Could you please approve the draft summary below or provide us with a suitably edited version.

"Singlet oxygen is known to react with carbon-carbon double bonds. Here, the authors show the unusual functionalization of ethereal alpha C-H bonds mediated by singlet oxygen under mild conditions to afford lactones and hydroperoxide products."

Our reply: Yes, the draft summary is good and appropriate.

Q3. To comply with our article templates, the text should be split into:

- Introduction (<1000 words), which must include the background and rationale for the work. The final paragraph should be a brief summary of the major results and conclusions. The results of the current study should only be discussed in this final paragraph. The Introduction should contain no references to figures or tables.
- Results, which must be split into subheaded sections, ensuring that the subheadings are no longer than 60 characters including spaces. Subheadings should contain no punctuation.
- Discussion (optional), without subheadings.
- Methods, which must be split into subheaded sections, ensuring that the subheadings are no longer than 60 characters including spaces.

Our reply: Yes, we have revised our manuscript according to the format of Nature Communications.

Q4. The choice of title is largely yours, however we do encourage to maximize the clarity by avoiding symbology even if appropriate.

Suggested change: Singlet oxygen-mediated selective C-H bond hydroperoxydation of ethereal hydrocarbons.

Our reply: Yes, we have changed the title to the suggested one. Thanks for the suggestion.

Q5. The results of the current study should only be discussed in the final paragraph of the introduction. Please move the highlighted text to the final paragraph of the

introduction.

Our reply: Yes, we have re-located the highlighted part to the final paragraph of the introduction section.

Q6. In Figure 1, in order to improve the clarity don´t use numbers for both compounds and hydrogens. Please label hydrogens with letters instead (a, a´, b, b´, etc.).

Our reply: Yes, as suggested, we have re-labeled hydrogen atoms using letters, such as a, b, etc., in Figure 1 as well as supplementary figures.

Q7. Please replace general citations to the SI (e.g. "See Supplementary Information") with specific citations (e.g. "See Supplementary Figure 1/Supplementary Table 1/Supplementary Methods/etc.").

Our reply: Yes, we have replaced general SI citation to more specific citations, such as, Supplementary Methods, Supplementary Information “Determination of singlet O_2 reaction rate with THF” section, etc.

Q8. In Figure 2, chemical structures that appear in figures in the manuscript (or as part of the single .cdx file mentioned below), should be drawn using the Nature Chemistry template (available at: http://www.nature.com/nchem/authors/submit/NatureChemistry.cds) or using the settings from this template - structures should, however, be scaled in proportion to fit our figure dimensions - if you do this though, please only scale atom labels and bond lengths, please do not reduce (or increase) the bond thickness. Also please comply with our chemical structure guide (http://www.nature.com/nchem/authors/submit/nchemstyleguide.pdf).

Our reply: Since the webpage for Nature Chemistry template (at http://www.nature.com/nchem/authors/submit/NatureChemistry.cds) does not exist anymore (when try to connect to the above website, “No page found” message always appears), we cannot use the Nature Chemistry template to prepare our chemical structures. However, we have followed the “Style guide for chemical structures” available in Nature Chemistry home page to prepare all our chemical structures.

Q9. The Methods section must be split into subheaded sections. Subheadings must be no longer than 60 characters including spaces and should not contain punctuation.

Our reply: Yes, we have followed the instruction in preparation of the Methods section.

==

Editorial questions regarding Supplementary Information.

Q1. The Supplementary Information can contain only the following sections:

- Supplementary Figures, labeled and referred to as Supplementary Figure X throughout both the Supplementary Information and the main text
- Supplementary Tables, labeled and referred to as Supplementary Table X throughout both the Supplementary Information and the main text
- Supplementary Notes, numbered Supplementary Note 1, Supplementary Note 2, etc.
- Supplementary Discussion
- Supplementary Methods
- Supplementary References, which should be self-contained. That is, references mentioned in both the main text and the Supplementary Information should be part of both reference lists so that the Supplementary Information does not refer to the reference list in the main paper and vice versa. References cited in the Supplementary Information should be numbered sequentially from 1. They should be formatted in the same style as the main paper.

Our reply: Yes, we have reorganized our Supplementary Information file to fit the format listed in Q1.

Q2. The SI does not require a title page, table of contents or author list – please remove.

Our reply: Yes, we have removed the title page, TOC, and the author list.
